# Silylimidazolium Hexafluorophosphate Salts as Synthetic Precursors to N-Heterocyclic Carbene Pentafluorophosphorus Adducts

**DOI:** 10.1021/acs.orglett.4c01549

**Published:** 2024-05-25

**Authors:** Rylan A. Rowsey, Jeremy D. Hilgar, Nathan A. Romero

**Affiliations:** Department of Chemistry & Biochemistry, University of California San Diego, La Jolla, California 92093, United States

## Abstract

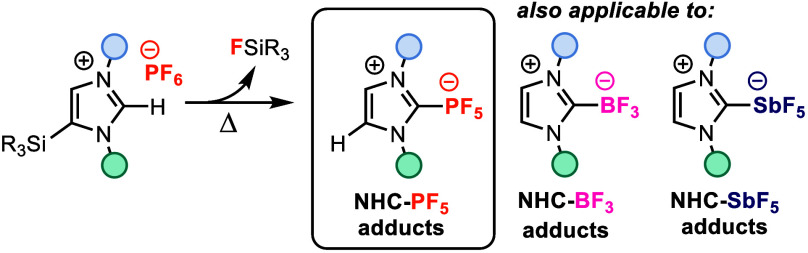

N-Heterocyclic carbene pentafluorophosphorus (NHC-PF_5_) adducts are six-coordinate phosphorus(v) compounds with emerging applications but poor synthetic accessibility. We have developed a simple and high yielding protocol for synthesizing imidazolylidene NHC-PF_5_ adducts from silylimidazolium hexafluorophosphate salts. Using this methodology, we have prepared a series of NHC-PF_5_ adducts in high yields, including new NHC-PF_5_ building blocks amenable to subsequent synthetic diversification. We also demonstrate that a similar approach enables access to analogous, synthetically elusive NHC-BF_3_ and NHC-SbF_5_ adducts.

N-Heterocyclic carbene-pentafluorophosphorus (NHC-PF_5_) adducts are neutral, six-coordinate phosphorus(v) compounds possessing high thermal and hydrolytic stability^1−4^ owing to the strong σ-donating properties of NHCs^5,6^ and the high fluoride ion affinity of P(v) centers.^7,8^ Although the scope of NHC-PF_5_ adducts reported to date is presently restricted to imidazolyl-PF_5_ species, these fluorine-rich compounds have garnered interest for applications in nuclear medicine^2^ and battery materials,^9^ and as potential liquid crystal mesogens.^10^ However, broader applications of NHC-PF_5_-containing molecules and materials remain limited by a dearth of suitable methodologies to access the NHC-PF_5_ functionality. Arduengo and co-workers were the first to report the synthesis of an NHC-PF_5_ adduct,^11^ which was prepared by treating a pregenerated imidazolylidene NHC with PF_5_ (Scheme 1a). Saturated imidazolylidene analogues can be constructed by reacting difluoroimidazolidines with PF_3_ (Scheme 1b),^1,10,12^ but the toxicity of PF_3_ poses practical limitations to this methodology. Using simple imidazolium hexafluorophosphate salts, Borzov and co-workers found that high vacuum pyrolysis at 300–400 °C produced NHC-PF_5_ adducts in high yields (Scheme 1c).^3,13^ However, hydrogen fluoride (HF) is the stoichiometric byproduct of this reaction, which putatively proceeds via thermal decomposition of PF_6_^–^ to F^–^ and PF_5_, followed by F^–^-mediated deprotonation of the imidazolium. As the only available methodologies in the literature to date, these examples reveal a need for safer and more general synthetic technologies to expand access to NHC-PF_5_ compounds.

**Scheme 1:**
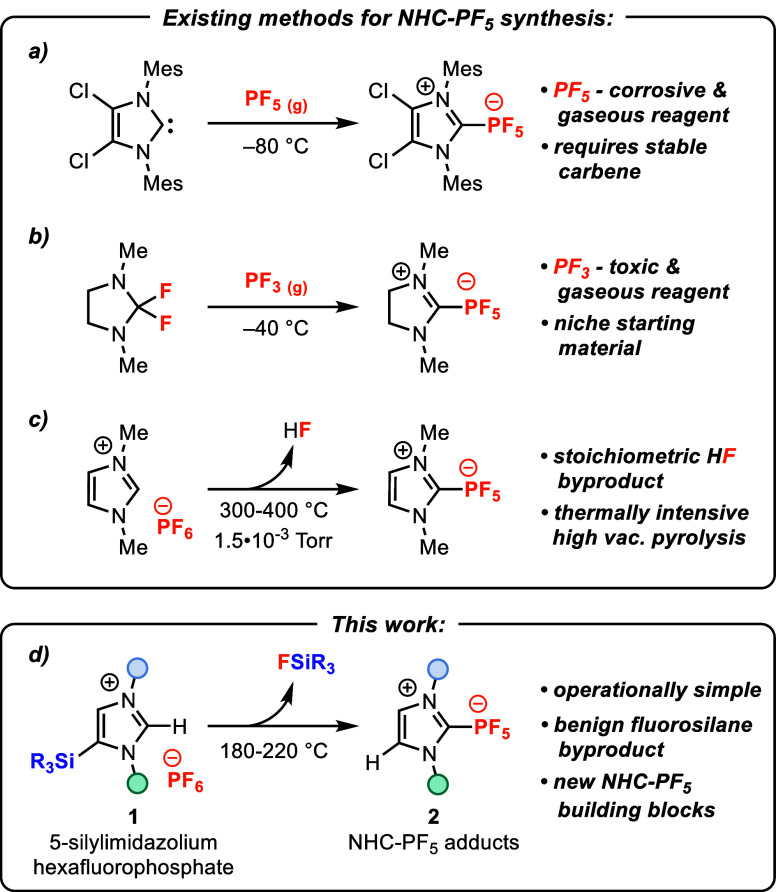
(a–c) Existing Synthetic Preparations of NHC-PF_5_ Adducts and (d) the Methodology Described in This Work

Here, we describe an operationally simple and high-yielding method for the preparation of NHC-PF_5_ adducts from 5-silylimidazolium hexafluorophosphate salts **1** (Scheme 1d). An advantage of the method reported by Borzov is that it obviates the need to handle PF_5_ (or PF_3_) by generating PF_5_ in situ from PF_6_^–^, although a critical limitation of this system is the extreme temperatures presumably required for PF_6_^–^ decomposition. Trends in the thermal decomposition of alkali metal PF_6_^–^ salts (e.g., *T*_decomp._ (LiPF_6_) = 97 °C; *T*_decomp._ (NaPF_6_) = 128 °C; *T*_decomp._ (KPF_6_) = 460 °C)^14^ suggested to us that Lewis acidic cations could facilitate defluorination of PF_6_^–^ at lower temperatures. Inspired by prior work in which PF_6_^–^ decomposition was observed in the presence of electrophilic silanes (Figure 1a),^15−19^ we envisaged that the Si-centers of silylimidazolium cations **3** may be sufficiently Lewis acidic to elicit defluorinative decomposition of PF_6_^–^ to PF_5_ (Figure 1b). Desilylation of a 2-silylimidazolium would deliver the requisite NHC **4** and PF_5_, similar to the mechanism proposed by Borzov, but with a comparatively inert fluorosilane as the byproduct, rather than HF. 2-Silylimidazolium salts **3** are indeed known to be highly electrophilic;^20^ however, 2-silylimidazolium salts and their synthetic precursors—2-silylimidazoles—are hydrolytically unstable,^21−23^ which would restrict the utility of 2-silylimidazolium hexafluorophosphate salts **3** as substrates for NHC-PF_5_ adduct synthesis. On the other hand, we considered 5-silylimidazolium salts **1** as alternative precursors, reasoning that the imidazol-5-ylidene **5** (an “abnormal NHC”^24,25^) initially generated upon desilylation could equilibrate to the more stable “normal NHC”^26,27^ in the presence of a catalytic weak Brønsted acid like **1** (Figure 1b) or **7** (see Figure 2). 5-Silylimidazolium salts **1** can be readily synthesized by alkylation or arylation of 5-silylimidazoles,^22,23,28^ or by lithiation-silylation of imidazol-2-ylidenes^29−33^ (Figure 1c).

**Figure 1:**
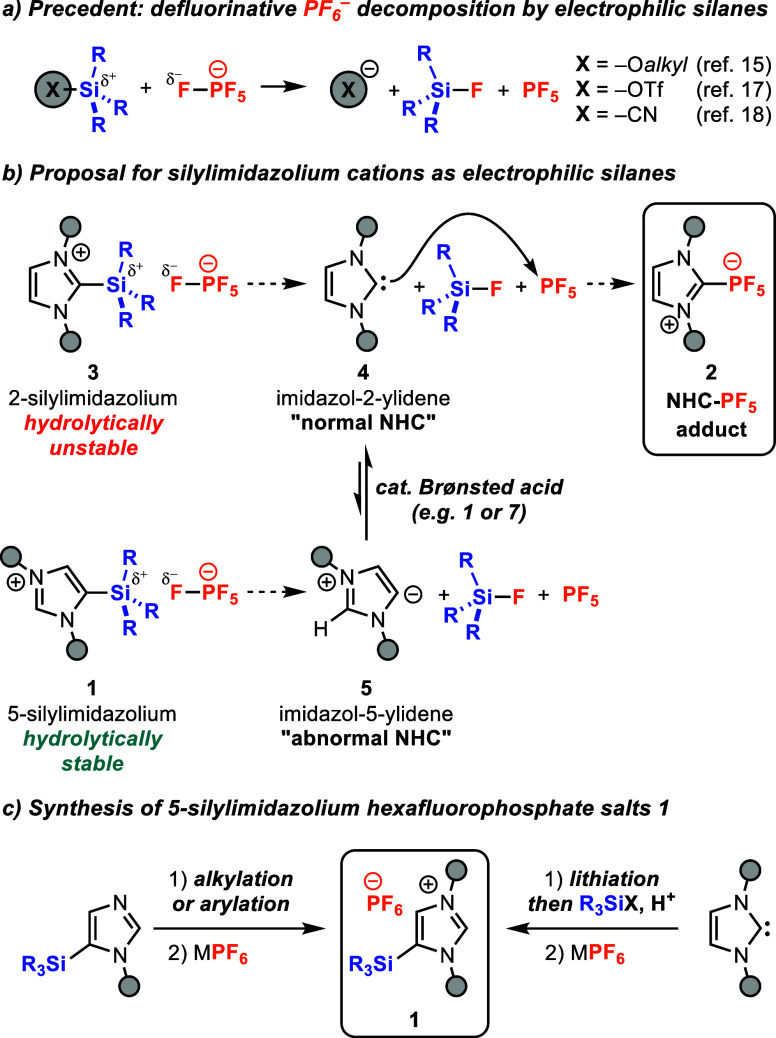
(a) Prior reports of defluorinative decomposition of PF_6_^–^ induced by electrophilic silanes. (b) Our design strategy for using silylimidazolium hexafluorophosphate salts to induce concomitant formation of PF_5_ and an NHC. (c) Synthetic routes to 5-silylimidazolium salts **1**.

**Figure 2:**
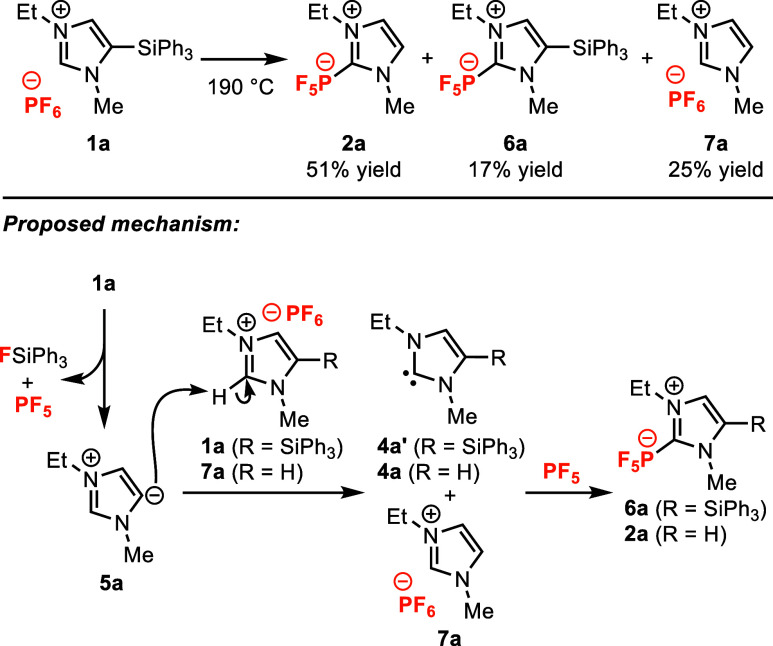
Initial results demonstrating the successful formation of NHC-adduct **2a** and mechanistic rationale to account for byproducts **6a** and **7a**.

We began our investigation by probing the thermal reactivity of 5-silylimidazolium hexafluorophosphate salt **1a** under solvent-free conditions (Figure 2). Upon heating at 190 °C, silylimidazolium **1a** underwent complete conversion and generated the desired NHC-PF_5_ adduct in a moderate yield (51%), along with another NHC-PF_5_ adduct, **6a**, and imidazolium hexafluorophosphate salt **7a** as byproducts in 17% and 25% yield, respectively. The identities of **2a** and **6a** were confirmed by X-ray diffraction analysis (Figure S12 and S13). Byproducts **6a** and **7a** are consistent with a mechanism involving bimolecular proton transfer between imidazol-5-ylidene **5a** and silylimidazolium **1a**, with silyl-NHC **4a’** and imidazolium **7a** as the resultant intermediates (Figure 2). Subsequent addition of silyl-NHC **4a’** to PF_5_ would produce the observed silyl-NHC-PF_5_ adduct **6a**. Adduct **6a** is more likely to form in the early stages of reaction, when silylimidazolium **1a** is more abundant than imidazolium **7a**. Ultimately, deprotonation of imidazolium **7a**, either by imidazol-5-ylidene **5a** or by imidazol-2-ylidene **4a’**, would generate the NHC **4a** that goes on to form the major product, NHC-PF_5_ adduct **2a**.

Building on these initial results, we used imidazolium hexafluorophosphate **1b** as a model substrate in our efforts to optimize the reaction, which primarily sought to minimize byproducts **6b** and **7b** (Table 1, Tables S1–S4). Heating **1b** to 200 °C with no solvent or additives produced the desired NHC-PF_5_ product **2b**, but in lower yield (17% yield) and lower conversion (58% conversion) than in the reaction of **1a** (Table 1, entry 1). When screening additives to improve conversion, we found that catalytic quantities of LiPF_6_ (0.1 equiv) led to a drastic improvement in the yield of **2b** at 71%, with only trace amounts of byproduct **6b** observed (Table 1, entry 2; Table S1). Use of other hexafluorophosphate salts as additives showed inferior performance (Table 1, entries 3–4; Table S2, entries 6–8), suggesting that the Li^+^ cation was uniquely responsible for the beneficial effect observed with LiPF_6_. LiCl also furnished improved yields of **2b** (73% yield), while other lithium salts (e.g., LiBr and LiOTf) were problematic (Table S2, entries 2–5).

**Table 1:**
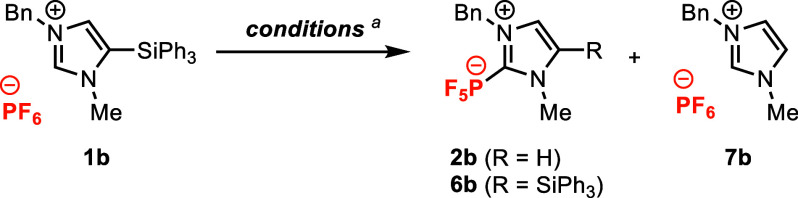
Summary of Reaction Optimization Using 5-Silylimidazolium Salt **1b**

entry	additives	temp. (°C)	% yield **2b**^b^	% yield **6b**^b^	% yield **7b**^b^
1	—	200	17^c^	5	28
2	0.1 eq. LiPF_6_	200	71	1	14
3	0.1 eq. KPF_6_	200	16	13	37
4	0.1 eq. AgPF_6_	200	39	6	32
5	0.1 eq. AlCl_3_	200	73	1	17
6	0.1 eq. ZnCl_2_	200	60	2	10
7	Ph_2_O	200	74	1	14
8	0.1 eq. LiPF_6_, Ph_2_O	200	80	0	12
9	—	220	47	3	32
10	0.1 eq. LiPF_6_	220	49	0	33
11	Ph_2_O	220	79	0	12
12	0.1 eq. LiPF_6_, Ph_2_O	220	74	0	21

a Reactions conducted at a 0.090 mmol scale according to the General Procedure described in the Supporting Information.

b % yield was determined from ^1^H NMR spectra of the crude reaction mixture relative to dimethylsulfone as an internal standard.

c % conversion was 58% for entry 1. For all other entries in this table, % conversion was 100%.

Considering the possibility that Li^+^ acts as a Lewis acid to promote PF_6_^–^ decomposition, we screened various Lewis acids, which gave high conversion of starting material in all cases (Table 1, entries 4–6; Table S2, entries 8–13). ZnCl_2_ and AlCl_3_ showed the highest yields of the desired product **2b** in this series at 60% and 73% yield, respectively, which are comparable to LiPF_6_. We did not obtain synthetically useful yields at reaction temperatures lower than 180 °C, and LiPF_6_ was necessary for productive reactivity under these conditions (e.g., Table S3, entry 17 vs entry 18). Use of high-boiling solvents generally led to improved yields (Table S3), with diphenyl ether (Ph_2_O) delivering the best results (Table 1, entry 7). There is marginal benefit to using LiPF_6_ in Ph_2_O at 200 °C (Table 1, entry 7 vs entry 8), and no benefit to using LiPF_6_ in Ph_2_O at 220 °C (Table 1, entry 11 vs entry 12), which is consistent with high background PF_6_^–^ decomposition at these elevated temperatures (see Supporting Information for additional discussion on the effect of Li^+^ and other Lewis acids).

We briefly surveyed the influence of the site and identity of the silyl group by reacting silylimidazolium salts **1b-b′′′** under the optimized reaction conditions (Figure 3). In stark contrast to silylimidazolium **1b**, its regioisomer **1b′** yielded no observable product **2b** at full conversion. It is possible that the imidazol-4-ylidene produced upon desilylation decomposes via a Sommelet-Hauser-type [2,3]-sigmatropic rearrangement (Figure S10),^34^ although we have not been able to identify any products that would support this conjecture. Under the same conditions, trimethylsilylimidazolium **1b′′** and triisopropylsilylimidazolium **1b′′′** both provided lower but serviceable yields of **2b**. Incomplete conversion in the reaction of **1b′′′** implies somewhat attenuated reactivity for this sterically hindered silane. Nonetheless, these results demonstrate that 5-silylimidazolium salts with different steric environments at the silicon center still support productive reactivity.

**Figure 3:**
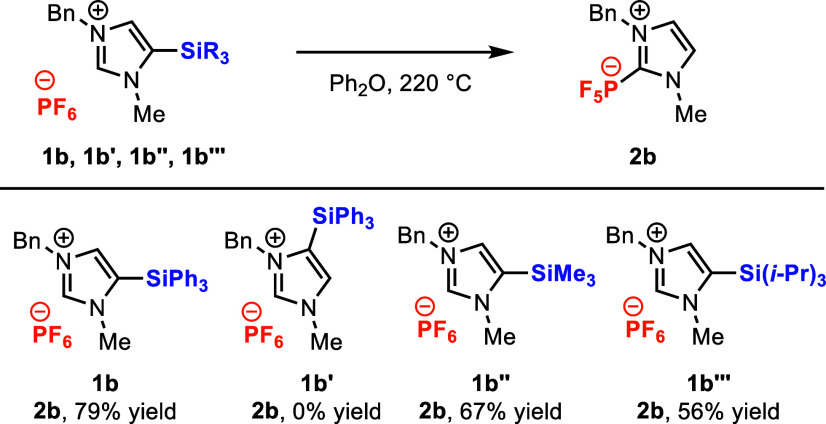
Evaluation of regioisomer and silane substituent effects in the reactions of **1b**–**1b′′′**

Having identified conditions that produce NHC-PF_5_ adduct **2b** in high yields, we further evaluated the substrate scope of this transformation with silylimidazolium salts **1a**–**m** (Figure 4). A majority of these substrates were prepared from 5-(triphenylsilyl)imidazole **S1**, which afforded easily tractable, crystalline silylimidazolium salts **1a**–**j** upon alkylation or arylation. Excellent isolated yields were obtained for NHC-PF_5_ adducts with *N*,*N-*dialkyl substitution (**2a,c,d**) and for benzyl substituted NHC-PF_5_ products **2b** and **2e**. Benzyl NHC-PF_5_ product **2b** was isolated in high yield when the reaction was conducted on a 1 mmol scale (63%). Reasonable isolated yields obtained for *N*-phenyl NHC-PF_5_ product **2f** (48% yield) shows that *N-*arylsilylimidazolium salts are also suitable substrates. Chloroethyl imidazolium **1h** gave lower isolated yields (17%) of NHC-PF_5_ adduct **2h** due to competing elimination of the alkyl chloride to an *N*-vinyl NHC-PF_5_ adduct (Figure S2). Substrates with moderately acidic C–H bonds (e.g., propargylimidazolium **1i**) and Lewis basic sites (e.g., nitrile **1j**) have thus far proven recalcitrant, and generally produced significant amounts of intractable material.

**Figure 4:**
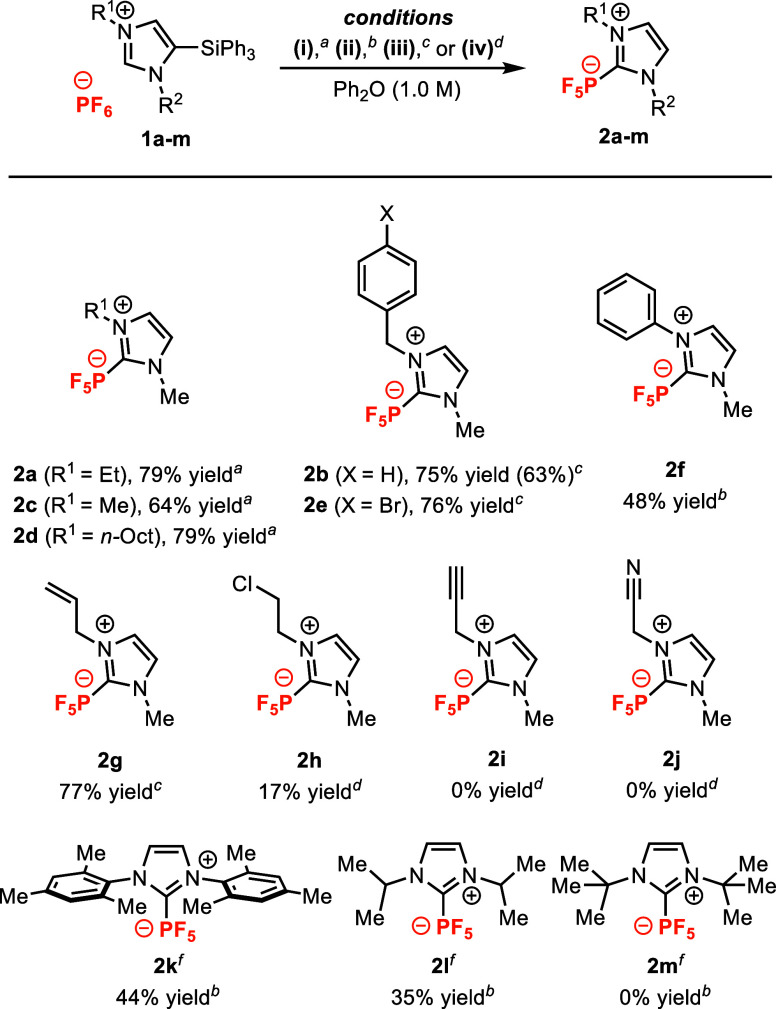
Substrate scope for the preparation of NHC-PF_5_ adducts **2a**–**m**. Reactions performed according to the General Procedure described in the Supporting Information. Reaction conditions: ^*a*^(i) 220 °C. ^*b*^(ii) 200 °C. ^*c*^(iii) 0.1 equiv of LiPF_6_, 200 °C. ^*d*^(iv) 0.1 equiv of LiPF_6_, 180 °C. ^*e*^% yield in parentheses at a 1.0 mmol scale. ^*f*^For **1k**–**1m**, a trimethylsilyl imidazolium salt was used instead of a triphenylsilyl imidazolium.

Informed by previous reports where 5-silyl-NHCs were generated by lithiation-silylation of imidazol-2-ylidenes,^29−31^ we prepared 5-silylimidazolium salts **1k**–**1m** directly from symmetrical imidazolium salts (see Supporting Information, Section 2d) and screened these sterically bulky substrates for reactivity. Dimesitylsilylimidazolium salt **1k** and diisopropylimidazolium salt **1l** afforded moderate yields of the corresponding NHC-PF_5_ adducts **2k** and **2l** (41% and 35%, respectively), establishing a two-step route to NHC-PF_5_ adducts from commercially available imidazolium salts. Unlike **1l**, di-*tert*-butylimidazolium salt **1m** did not produce the expected NHC-PF_5_ adduct **2m**, and instead formed *N*-PF_5_ adduct **2m′** (see Supporting Information, Section 3g).

Prior NHC-PF_5_ syntheses have largely failed to produce NHC-PF_5_ compounds with functional handles amenable to subsequent modification. Among the products accessible by our methodology, NHC-PF_5_ adducts **2e** and **2g** stand out as versatile synthetic intermediates (Figure 5). Using standard Suzuki-Miyaura-type cross-coupling conditions, adduct **2e** was smoothly transformed to biaryl **8** (90% yield). Biaryl **8** and similar NHC-PF_5_-containing structures are candidates of interest for new mesogenic materials, which we are currently investigating in our lab. The NHC-PF_5_ moiety is also stable to free radical conditions, which enabled successful preparation of the novel non-natural amino acid derivative **9** in 82% yield from allyl NHC-PF_5_ adduct **2g** through a thiol–ene reaction. These examples demonstrate that the NHC-PF_5_ functionality is inert to additional synthetic manipulations, and they illustrate how our methodology serves as an entryway to more complex NHC-PF_5_-bearing molecules.

**Figure 5:**
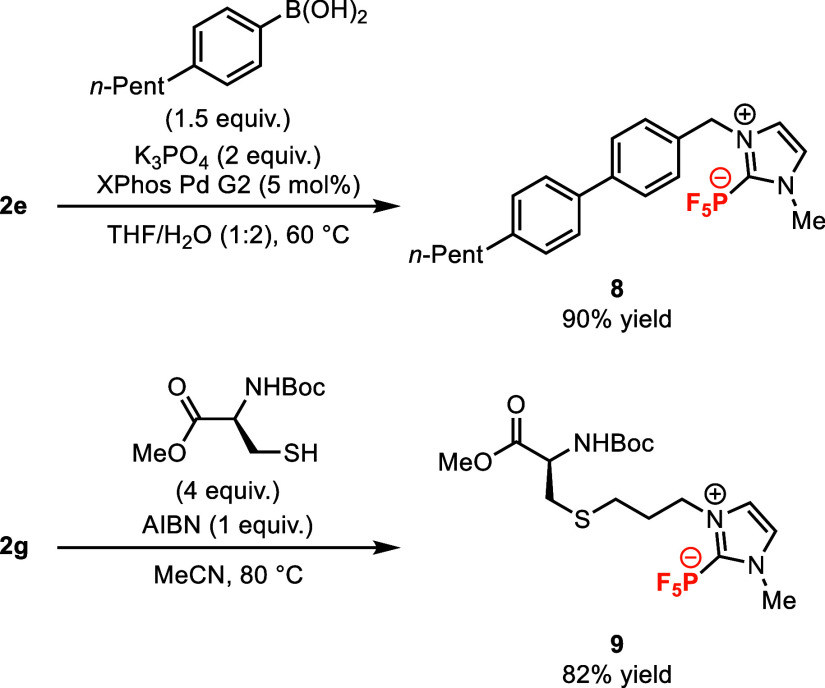
Synthetic diversification of NHC-PF_5_ adducts **2e** and **2g**.

Finally, we extended this synthetic approach to prepare NHC adducts of other fluorinated main group element centers (Figure 6). Under similar conditions used for the synthesis of NHC-PF_5_ adducts, NHC-BF_3_ adduct **11** was isolated in 72% yield from tetrafluoroborate salt **10**, offering a route to NHC-BF_3_ adducts that is complementary to existing methods.^3,11,35^ NHC-SbF_5_ adducts are comparatively elusive, with a singular literature precedent that was synthesized by reacting a stable NHC with SbF_5_.^11^ We successfully synthesized NHC-SbF_5_ adduct **13** in 37% yield simply by heating silylimidazolium hexafluoroantimonate salt **12** at 180 °C, which enabled isolation and structural characterization of NHC-SbF_5_ adduct **13** by XRD (Figure S14). This unoptimized result nevertheless circumvents the use of SbF_5_ as in the prior report^11^ and permits expansion of this rare class of molecules beyond NHC-SbF_5_ adducts derived from isolable NHCs.

**Figure 6:**
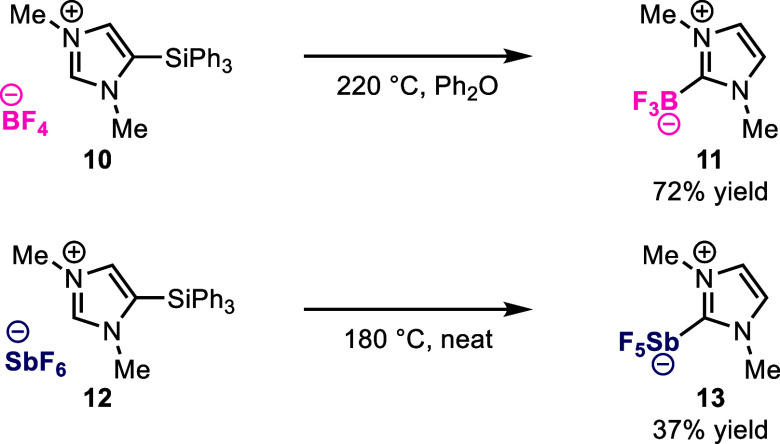
Preparation of NHC-BF_3_ and NHC-SbF_5_ adducts from the corresponding silylimidazolium tetrafluoroborate salt **10** and hexafluoroantimonate salt **12**.

In summary, we have developed a simple method for generating imidazolyl NHC-PF_5_ adducts in good to moderate yields from 5-silylimidazolium hexafluorophosphate salts. This transformation likely proceeds via in situ formation of PF_5_ from PF_6_^–^ with a benign fluorosilane as the primary byproduct. As such, this protocol obviates the use of unstable and toxic reagents (e.g., PF_5_ and PF_3_) or byproducts (e.g., HF) that have rendered existing methodologies impractical. Our methodology affords key synthetic lynchpins for divergent functionalization, which we anticipate will enable broader deployment of the NHC-PF_5_ functionality across synthetic disciplines. Furthermore, as evidenced by the isolation of NHC-BF_3_ and NHC-SbF_5_ adducts through a similar methodology, this work establishes the utility of silylimidazolium salts in accessing novel NHC-main group element adducts.

## Data Availability

The data underlying this study are available in the published article and its Supporting Information.

## Supporting Information Available

Synthetic procedures, characterization data, and supplementary figures (PDF)
