# Supplementary material for: Silylimidazolium Hexafluorophosphate Salts as Synthetic Precursors to N-Heterocyclic Carbene Pentafluorophosphorus Adducts
Source: Org Lett. 2024 May 25;26(22):4750–5. doi: 10.1021/acs.orglett.4c01549 (PMC11165577; doi:10.1021/acs.orglett.4c01549)
Supplement: Supplementary file 1 — ol4c01549_si_001.pdf [file ol4c01549_si_001.pdf]

Supporting Information

# Silylimidazolium Hexafluorophosphate Salts as Synthetic Precursors to N-Heterocyclic Carbene Pentafluorophosphorus Adducts

Rylan A. Rowsey, Jeremy D. Hilgar, Nathan A. Romero*

*Department of Chemistry & Biochemistry, University of California, San Diego, La Jolla, CA 92093, USA*

*naromero@ucsd.edu*

1. General Information ... 2
2. Synthesis and Characterization of Starting Materials ... 3
   a) Synthesis of 5-silylimidazoles ... 3
   b) Synthesis of silylimidazolium hexafluorophosphate salts ... 5
   c) Synthesis of silylimidazolium tetrafluoroborate 10 and silylimidazolium hexafluoroantimonate 12 ... 12
   d) Synthesis of 5-silylimidazolium salts by lithiation-silylation of symmetrical imidazolium salts ... 13
3. Synthesis and Characterization of NHC-$PF_5$ Adducts ... 15
   a) General procedure the preparation of NHC-PF5 adducts from silylimidazolium salts ... 15
   b) Preparation of NHC-$PF_5$ Adducts 2a-2h, 2k, and 2l ... 16
   c) Unsuccessful substrates ... 21
4. Derivatization of NHC-$PF_5$ adducts 2e and 2g ... 24
5. Additional Optimization Data and Mechanistic Discussion ... 26
   a) Table S1. $LiPF_6$ equivalents screen ... 26
   b) Table S2. Additive screen ... 26
   c) Table S3. Solvent screen ... 27
   d) Table S4. Evaluation of air and moisture tolerance ... 28
   e) Additional Control Experiments ... 28
   f) Additional mechanistic discussion ... 29
6. X-ray Crystallography ... 32
   a) Crystallization of compounds 2a, 6a, and 13 ... 32
   b) X-ray Crystallography Experimental Details for compounds 2a, 6a, and 13 ... 32
7. NMR Spectra ... 36
8. References ... 104

## 1. General Information

Unless otherwise stated, synthetic manipulations were conducted under ambient atmosphere with no precautions taken to exclude air or moisture. With the exception of THF, which was purified by passage through two columns of alumina using a JC Meyer solvent purification system, all other chemicals were of reagent grade and used as received unless otherwise specified.

$^{1}H$, $^{13}C$, $^{31}P$, $^{19}F$, and $^{11}B$ NMR spectra were recorded on a 400 Varian Mercury Plus spectrometer or a 300 Bruker AVA spectrometer. Chemical shifts (δ) are reported in parts per million (ppm) using the residual solvent signals as the internal standard for proton and carbon spectra (Chloroform-*d*: $\delta_H$ 7.26 ppm, $\delta_C$ 77.16 ppm; Acetone-$d_6$: $\delta_H$ 2.05 ppm, $\delta_C$ 29.84 ppm).[1] $^{19}F$ NMR chemical shifts are reported in ppm relative to $CFCl_3$ as an external standard. ($\delta_F$ 0 ppm). $^{31}P$ NMR chemical shifts are reported in ppm relative to $H_3PO_4$ as an external standard ($\delta_P$ 0 ppm). $^{11}B$ NMR chemical shifts are reported in ppm relative to $BF_3$•$OEt_2$ as an external standard ($\delta_B$ 0 ppm). NMR spectral data are reported as follows: chemical shift (δ, ppm), multiplicity (s = singlet, d = doublet, t = triplet, q = quartet, and m = multiplet, coupling constant(s) in Hz, integration).

High resolution mass spectra (HRMS) were obtained using an Agilent 6230 Accurate-Mass TOFMS. Prior to sample analysis, the instrument was calibrated by using Agilent ESL-L Low Concentration Tuning Mix (Part number G1969-85000, Agilent Technologies). In addition, internal mass calibration using Agilent Reference Mass Solution (Part number G1969-85001, Agilent Technologies) was enabled during HRMS data acquisition.

## 2. Synthesis and Characterization of Starting Materials

### a) Synthesis of 5-silylimidazoles

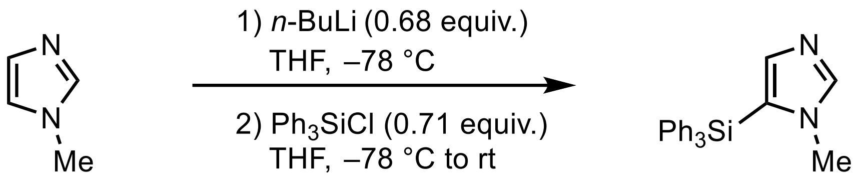


**S1**

**1-methyl-5-(triphenylsilyl)-1*H*-imidazole (S1).** To a flame-dried Schlenk flask 1-methylimidazole (1.2 mL, 15 mmol, 1.0 equiv.) and THF (31 mL) were added, and the solution was sparged with nitrogen for 20 minutes while cooling to -78 °C. A solution of *n*-BuLi (4.8 mL, 11 mmol, 0.71 equiv.) in hexanes was added dropwise with stirring. After 1 hour at -78 °C, chlorotriphenylsilane (3.0 g, 10 mmol, 0.68 equiv.) was added and the solution stirred overnight while slowly warming to ambient temperature. The reaction mixture was quenched by addition of saturated aqueous $NH_4Cl$. The layers were separated and the aqueous phase was extracted three times with ethyl acetate. The combined organic extracts were washed with brine, dried over $MgSO_4$, and concentrated in vacuo. The crude residue was purified via silica gel chromatography (100% ethyl acetate) to obtain the title compound **S1** in 2.4 g (47% yield with respect to 1-methylimidazole).

*Note: In our attempts to improve the isolated yields of **S1**, we found that using greater than 1 equivalent of n-BuLi led to lower yields, with the best results obtained with ca. 0.7 equiv. n-BuLi. A "silicon dance" reaction similar to that observed by Shapiro and Marzi*[2] *is likely occurring in this reaction to afford the 5-silylimidazole **S1** over the 2-silylimidazole regioisomer.*

$^{1}$H NMR (400 MHz, Chloroform-*d*) δ 7.67 (s, 1H), 7.60 (d, *J* = 6.5 Hz, 6H), 7.46 (t, *J* = 7.3 Hz, 3H), 7.40 (t, *J* = 7.4 Hz, 6H), 7.12 (s, 1H), 3.35 (s, 3H).
$^{13}$C NMR (101 MHz, Chloroform-*d*) δ 143.9, 143.1, 136.1, 133.1, 130.2, 128.3, 125.7, 34.7.
HRMS (ESI-TOFMS): $[M+H]^+$ calculated for $[C_{22}H_{21}N_2Si]^+$ = 341.1469, found = 341.1467

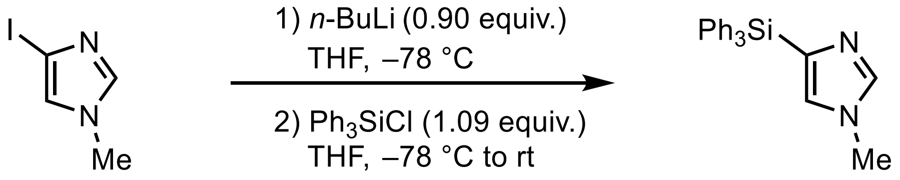


**S2**

**1-methyl-4-(triphenylsilyl)-1*H*-imidazole (S2**). To a flame-dried Schlenk flask 4-Iodo-1-methylimidazole (0.911 g, 4.38 mmol, 1.0 equiv.) and THF (13 mL) were added, and the solution was sparged with nitrogen for 20 minutes while cooling to -78 °C. A solution of *n*-BuLi (1.75 mL, 3.94 mmol, 0.90 equiv.) in hexanes was added dropwise with stirring. After 1 hour at -78 °C, chlorotriphenylsilane (1.40 g, 4.76 mmol, 1.09 equiv.) was added and the solution stirred overnight while slowly warming to ambient temperature. The reaction mixture was quenched by addition of saturated aqueous $NH_4Cl$. The layers were separated and the aqueous phase was extracted three times with ethyl acetate. The combined organic extracts were washed with brine, dried over $MgSO_4$, and concentrated in vacuo. The crude residue was purified via silica gel chromatography (100% ethyl acetate) to obtain the title compound **S2** in 93.2 mg (6% yield).

$^{1}$H NMR (400 MHz, Chloroform-*d*) δ 7.72 (s, 1H), 7.67 – 7.63 (m, 6H), 7.45 – 7.33 (m, 9H), 6.93 (d, *J* = 1.3 Hz, 1H), 3.65 (s, 3H).
$^{13}$C NMR (101 MHz, Chloroform-*d*) δ 141.2, 137.1, 136.2, 134.8, 131.5, 129.6, 127.9, 33.1.
HRMS (ESI-TOFMS): $[M+H]^+$ calculated for $[C_{22}H_{21}N_2Si]^+$ = 341.1469, found = 341.1466

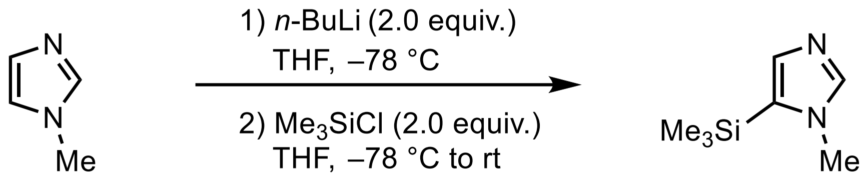


**S3**

**1-methyl-5-(trimethylsilyl)-1*H*-imidazole (S3).** To a flame-dried Schlenk flask 1-methylimidazole (1.5 mL, 19 mmol, 1.0 equiv.) and THF (55 mL) were added, and the solution was sparged with nitrogen for 20 minutes while cooling to -78 °C. A solution of *n*-BuLi (17 mL, 39 mmol, 2.0 equiv.) in hexanes was added dropwise with stirring. After 1 hour at -78 °C, chlorotrimethylsilane (4.8 mL, 38 mmol, 2.0 equiv.) was added and the solution stirred overnight while slowly warming to ambient temperature. The reaction mixture was quenched by addition of saturated aqueous $NH_4Cl$. The layers were separated and the aqueous phase was extracted three times with ethyl acetate. The combined organic extracts were washed with brine, dried over $MgSO_4$, and concentrated in vacuo. The crude residue was purified via silica gel chromatography (100% ethyl acetate) and subsequent vacuum distillation to obtain the title compound **S3** in 0.18 g (6% yield). $^1$H NMR spectra match the data reported in the literature.[3]

$^1$H NMR (400 MHz, Chloroform-*d*) δ 7.55 (s, 1H), 7.12 (s, 1H), 3.71 (s, 3H), 0.31 (s, 9H).

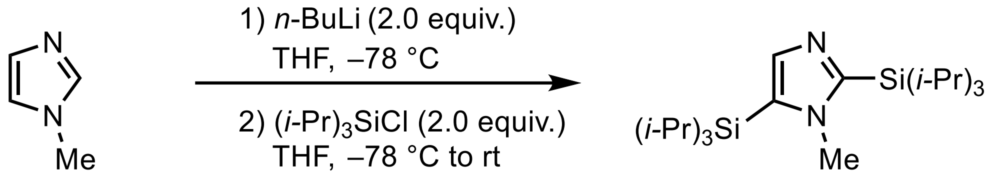


**S4**

**1-methyl-2,5-bis(triisopropylsilyl)-1H-imidazole (S4).** To a flame-dried Schlenk flask 1-methylimidazole (1.0 mL, 13 mmol, 1.0 equiv.) and THF (37 mL) were added, and the solution was sparged with nitrogen for 20 minutes while cooling to -78 °C. A solution of *n*-BuLi (11 mL, 25 mmol, 2.0 equiv.) in hexanes was added dropwise with stirring. After 1 hour at -78 °C, chlorotriisopropylsilane (5.5 mL, 26 mmol, 2.0 equiv.) was added to the solution under a counterflow of nitrogen and the solution stirred overnight while slowly warming to ambient temperature. The reaction mixture was quenched by addition of saturated aqueous $NH_4Cl$. The layers were separated and the aqueous phase was extracted three times with ethyl acetate. The combined organic extracts were washed with brine, dried over $MgSO_4$, and concentrated in vacuo. The crude residue was purified by recrystallization from hot MeCN to obtain the title compound **S4** in 1.3 g (26% yield).

*Note: In the synthesis of the other 5-silylimidazoles used in this study (i.e., **S1-S3**), the crude reaction mixtures likely contain some fraction of 2-silylimidazoles, which undergo protodesilylation during aqueous workup. However, 2-triisopropylsilylimidazoles appear to be more stable to hydrolysis under neutral conditions, as evidenced by the isolation of **S4**. Protodesilylation of the 2-triisopropylsilyl group occurs adventitiously during the alkylation of **S4** to **1b'''** as shown below.*

$^1$H NMR (400 MHz, Acetone-$d_6$) δ 7.29 (s, 1H), 3.84 (s, 3H), 1.52 (hept, $J$ = 7.5 Hz, 2H), 1.42 (hept, $J$ = 7.4 Hz, 1H), 1.12 (app t, $J$ = 7.3 Hz, 36H).
$^{13}$C NMR (101 MHz, Acetone-$d_6$) δ 154.0, 143.0, 128.9, 36.2, 19.3 (d, $J$ = 2.4 Hz), 12.9 (d, $J$ = 39.5 Hz).
HRMS (ESI-TOFMS): $[M+H]^+$ calculated for $[C_{22}H_{47}N_2Si_2]^+$ = 395.3272, found = 395.3269

## b) Synthesis of silylimidazolium hexafluorophosphate salts

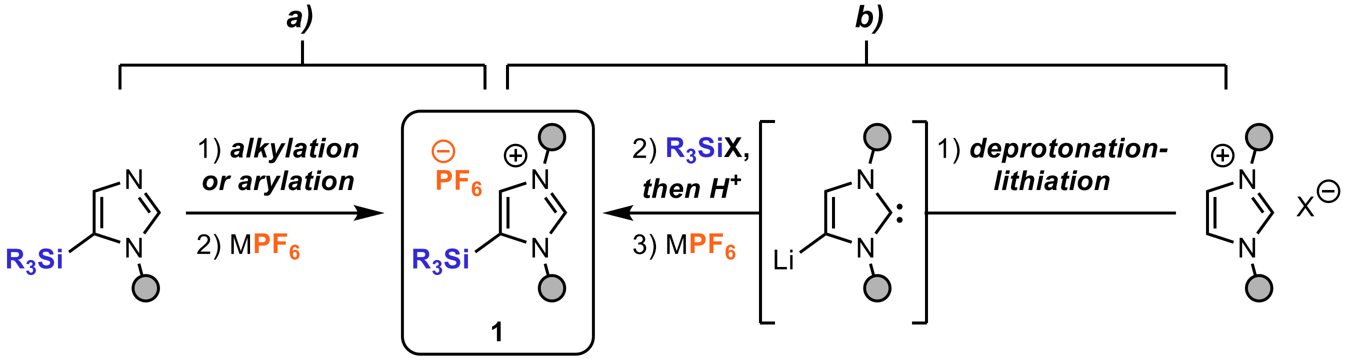


**Figure S1.** General synthetic routes used to prepare silylimidazolium hexafluorophosphate salts **1**: **a)** alkylation or arylation of 5-silylimidazoles (i.e., **S1-S4**), followed by anion metathesis. **b)** deprotonation-lithiation of symmetrical imidazolium salts, followed by trapping the organolithium with a silyl electrophile ($R_3SiX$) then quenching with acid and anion metathesis.

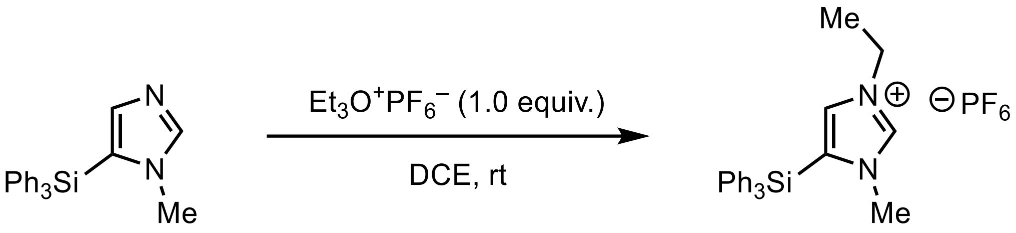


**3-ethyl-1-methyl-5-(triphenylsilyl)-1*H*-imidazol-3-ium hexafluorophosphate (1a).** To a flame-dried Schlenk flask was added 1-methyl-5-(triphenylsilyl)-1*H*-imidazole **S1** (500 mg, 1.47 mmol, 1.00 equiv.) and triethyloxonium hexafluorophosphate (364 mg, 1.47 mmol, 1.00 equiv.), and the vessel was subjected to three cycles of evacuation and purging with nitrogen. Anhydrous 1,2-dichloroethane (10.0 mL) was added, and the mixture was stirred at ambient temperature for 16 hours under nitrogen. A white precipitate was collected by vacuum filtration, affording the title compound **1a** in 703 mg (1.37 mmol, 93% yield).

$^{1}$H NMR (400 MHz, Acetone-$d_6$) δ 9.27 (s, 1H), 7.70 – 7.67 (m, 6H), 7.65 (d, $J$ = 1.6 Hz, 1H), 7.63 – 7.57 (m, 3H), 7.55 – 7.49 (m, 6H), 4.43 (q, $J$ = 7.3 Hz, 2H), 3.67 (s, 3H), 1.56 (t, $J$ = 7.3 Hz, 3H).
$^{13}$C NMR (101 MHz, Acetone-$d_6$) δ 141.9, 136.7, 134.4, 131.9, 131.7, 131.2, 129.6, 45.7, 37.9, 15.6.
$^{19}$F NMR (376 MHz, Acetone-$d_6$) δ -72.49 (d, $J$ = 707.6 Hz).
$^{31}$P NMR (162 MHz, Acetone-$d_6$) δ -143.67 (hept, $J$ = 712.2 Hz).

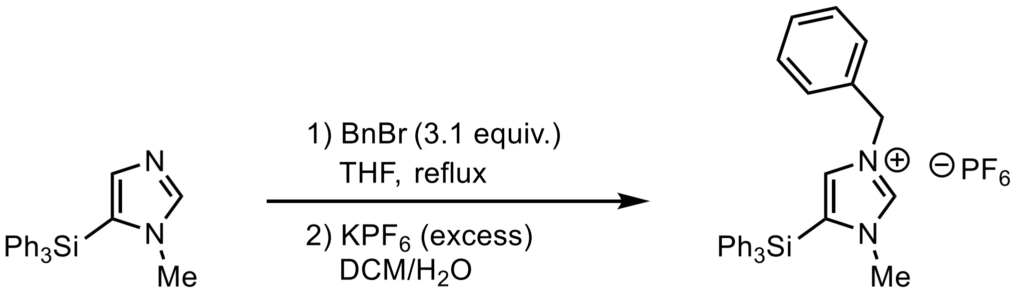


**3-benzyl-1-methyl-5-(triphenylsilyl)-1*H*-imidazol-3-ium (1b).** Benzyl bromide (0.27 mL, 2.3 mmol, 3.1 equiv.) was slowly added to a stirred solution of **S1** (0.25 g, 0.74 mmol, 1.0 equiv.) in THF (11 mL). The reaction mixture was then heated at reflux for 21 hours and cooled to room temperature. A white precipitate was collected by vacuum

filtration, then taken up in DCM and washed three times with a saturated aqueous solution of $KPF_6$. The organic phase was washed with deionized water, dried with $MgSO_4$, filtered, and concentrated in vacuo. The residue was recrystallized from a mixture of hot acetone/$H_2O$, yielding the title compound **1b** in 0.30 g (70% yield).

$^1$H NMR (400 MHz, Acetone-*d*$_6$) δ 9.27 (s, 1H), 7.71 – 7.65 (m, 7H), 7.64 – 7.54 (m, 3H), 7.55 – 7.46 (m, 8H), 7.49 – 7.38 (m, 3H), 5.59 (s, 2H), 3.67 (s, 3H).
$^{13}$C NMR (101 MHz, Acetone-*d*$_6$) δ 142.4, 136.7, 135.0, 134.6, 132.0, 131.9, 131.1, 130.0, 129.9, 129.6, 129.5, 53.5, 38.0.
$^{19}$F NMR (376 MHz, Acetone-*d*$_6$) δ -72.36 (d, *J* = 707.8 Hz).
$^{31}$P NMR (162 MHz, Acetone-*d*$_6$) δ -143.63 (hept, *J* = 707.9 Hz).
HRMS (ESI-TOFMS): $[M]^+$ calculated for $[C_{29}H_{27}N_2Si]^+$ = 431.1938, found = 431.1936

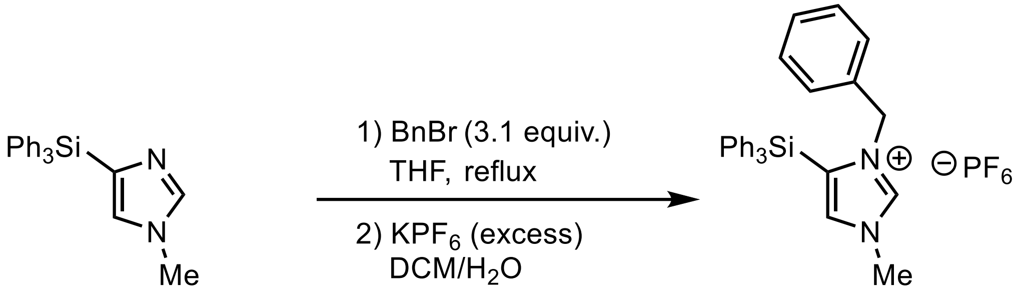


**S2** **1b'**

**3-benzyl-1-methyl-4-(triphenylsilyl)-1*H*-imidazol-3-ium hexafluorophosphate (1b').** The title compound was prepared by benzylation of 1-methyl-4-(triphenylsilyl)-1*H*-imidazole **S2** (93 mg, 270 µmol, 1.0 equiv.) with benzyl bromide (0.10 mL, 3.1 mmol, 3.1 equiv.) following a similar procedure as described for **1b** above. The title compound was isolated in 79 mg (0.14 mmol, 50% yield) after recrystallization from hot EtOH/MeCN.

$^1$H NMR (400 MHz, Acetone-*d*$_6$) δ 9.09 (s, 1H), 7.74 – 7.68 (m, 6H), 7.64 (s, 1H), 7.62 – 7.56 (m, 3H), 7.54 – 7.46 (m, 6H), 7.36 – 7.29 (m, 1H), 7.29 – 7.22 (m, 2H), 6.97 (s, 1H), 6.95 (s, 1H), 5.16 (s, 2H), 4.07 (s, 4H).
$^{13}$C NMR (101 MHz, Acetone-*d*$_6$) δ 142.3, 136.8, 136.1, 134.0, 132.0, 131.6, 131.1, 129.9, 129.9, 129.6, 129.3, 54.5, 36.6.
$^{19}$F NMR (376 MHz, Acetone-*d*$_6$) δ -72.52 (d, *J* = 707.5 Hz).
$^{31}$P NMR (162 MHz, Acetone-*d*$_6$) δ -143.66 (hept, *J* = 704.9 Hz).

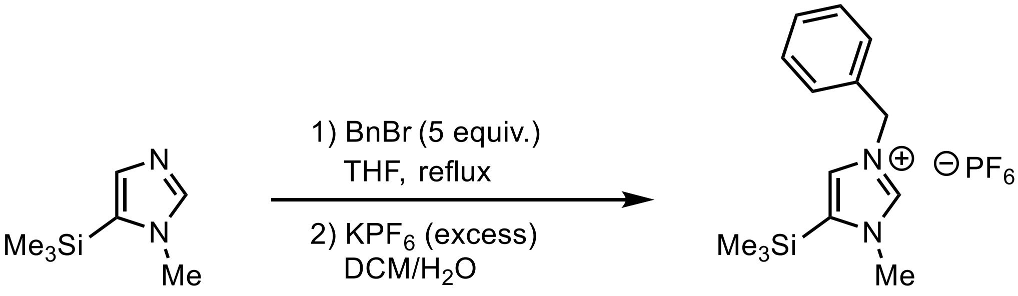


**S1** **1b''**

**3-benzyl-1-methyl-5-(trimethylsilyl)-1*H*-imidazol-3-ium (1b'').** The title compound was prepared by alkylation of 1-methyl-5-(triphenylsilyl)-1*H*-imidazole **S1** (150 mg, 0.97 mmol, 1.00 equiv.) with benzyl bromide (0.58 mL, 4.9 mmol, 5.0 equiv.) following a similar procedure as described for **1c** below. The title compound was isolated in 375 mg (0.96 mmol, 99% yield) after recrystallization from hot EtOH/MeCN.

$^1$H NMR (400 MHz, Acetone-*d*$_6$) δ 9.17 (s, 1H), 7.83 (s, 1H), 7.52 – 7.48 (m, 2H), 7.46 – 7.41 (m, 3H), 5.54 (s, 2H), 4.09 (s, 3H), 0.43 (s, 9H).
$^{13}$C NMR (101 MHz, Acetone-*d*$_6$) δ 140.6, 136.7, 135.3, 130.9, 130.0, 129.9, 129.5, 53.3, 37.4, -1.6.
$^{19}$F NMR (376 MHz, Acetone-*d*$_6$) δ -72.34 (d, *J* = 707.8 Hz).
$^{31}$P NMR (162 MHz, Acetone-*d*$_6$) δ -143.64 (hept, *J* = 707.7 Hz).

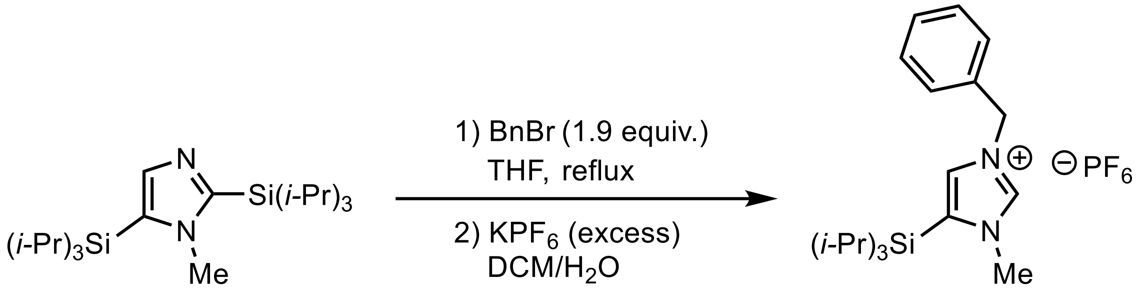


**3-benzyl-1-methyl-5-(triisopropylsilyl)-1*H*-imidazol-3-ium hexafluorophosphate (1b’’’).** The title compound was prepared by benzylation of 1-methyl-5-(triisopropylsilyl)-1*H*-imidazole **S4** (0.72 g, 1.8 mmol, 1.0 equiv.) with benzyl bromide (0.41 mL, 3.4 mmol, 1.9 equiv.) following a similar procedure as described for **1b** above. The title compound was isolated in 0.44 g (0.93 mmol, 52% yield) after recrystallization from hot EtOH.

$^{1}$H NMR (400 MHz, Acetone-$d_6$) δ 9.20 (s, 1H), 7.95 (s, 1H), 7.58 – 7.34 (m, 5H), 5.59 (s, 2H), 4.09 (d, *J* = 2.5 Hz, 3H), 1.54 (hept, *J* = 7.5 Hz, 3H), 1.14 (d, *J* = 7.6 Hz, 18H).
$^{13}$C NMR (101 MHz, Acetone-$d_6$) δ 141.6, 135.3, 132.8, 132.4, 130.0, 129.9, 129.3, 53.4, 38.2, 18.7, 12.1.
$^{19}$F NMR (376 MHz, Acetone-$d_6$) δ -72.36 (d, *J* = 707.8 Hz).
$^{31}$P NMR (162 MHz, Acetone-$d_6$) δ -143.64 (hept, *J* = 710.2 Hz).).

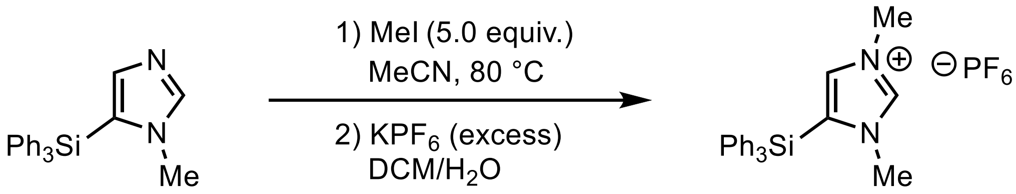


**1,3-dimethyl-5-(triphenylsilyl)-1*H*-imidazol-3-ium hexafluorophosphate (1c).** In a 2 dram vial, 1-methyl-5-(triphenylsilyl)-1*H*-imidazole **S1** (200 mg, 0.59 mmol, 1.00 equiv.) was dissolved in acetonitrile (2 mL). Iodomethane (0.19 mL, 2.94 mmol, 5.00 equiv.) was added, and the vial was sealed with a screw cap and heated at 80 °C for 20 hours. After cooling to room temperature, volatiles were removed, and the solid residue was washed with several portions of diethyl ether. The residue was redissolved in DCM (30 mL) and washed three times with a saturated aqueous solution of $KPF_6$. The organic phase was filtered through a pad of celite, concentrated in vacuo, and the resulting residue was recrystallized from boiling methanol to give the title compound **1c** in 198 mg (0.40 mmol, 67% yield).

$^{1}$H NMR (400 MHz, Acetone-$d_6$) δ 9.23 (s, 1H), 7.71 – 7.66 (m, 6H), 7.63 – 7.57 (m, 3H), 7.57 – 7.49 (m, 7H), 4.08 (s, 3H), 3.67 (s, 3H).
$^{13}$C NMR (101 MHz, Acetone-$d_6$) δ 142.77, 136.7, 135.9, 131.6, 131.2, 129.7, 37.8, 36.4.
$^{19}$F NMR (376 MHz, Acetone-$d_6$) δ -72.52 (d, *J* = 707.6 Hz).
$^{31}$P NMR (162 MHz, Acetone-$d_6$) δ -143.78 (hept, *J* = 703.7 Hz).

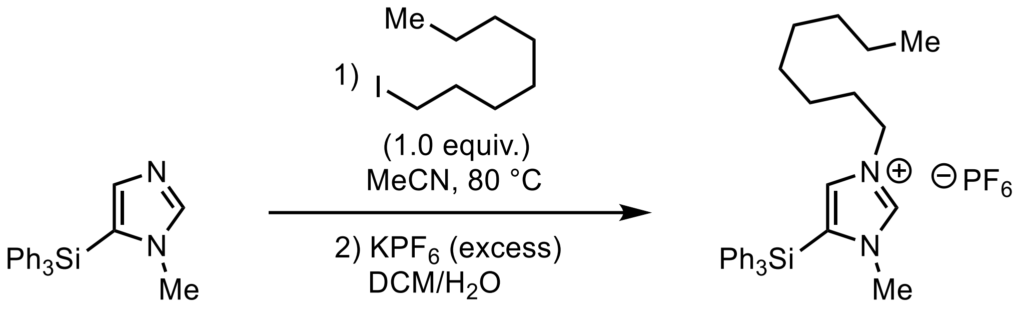


**S1** **1d**

**1-methyl-3-octyl-5-(triphenylsilyl)-1*H*-imidazol-3-ium hexafluorophosphate (1d).** The title compound was prepared by alkylation of 1-methyl-5-(triphenylsilyl)-1*H*-imidazole **S1** (400 mg, 1.17 mmol, 1.00 equiv.) with 1-iodooctane (282 mg, 1.17 mmol, 1.00 equiv.) following a similar procedure as described for **1c** above. The title compound was isolated in 193 mg (0.32 mmol, 27% yield) after recrystallization from hot EtOH/MeCN (8:1).

$^{1}$H NMR (400 MHz, Acetone-$d_6$) δ 9.27 (s, 1H), 7.69 (d, *J* = 6.6 Hz, 6H), 7.65 (s, 1H), 7.62 – 7.57 (m, 3H), 7.55 – 7.49 (m, 6H), 4.39 (t, *J* = 7.4 Hz, 2H), 3.67 (s, 3H), 1.94 (p, 2H), 1.42 – 1.17 (m, 10H), 0.88 – 0.77 (m, 3H).
$^{13}$C NMR (101 MHz, Acetone-$d_6$) δ 142.1, 136.7, 134.7, 131.9, 131.7, 131.2, 129.6, 50.4, 37.9, 32.4, 30.9, 29.8, 29.6, 26.8, 23.2, 14.3.
$^{19}$F NMR (376 MHz, Acetone-$d_6$) δ -72.45 (d, *J* = 707.8 Hz).
$^{31}$P NMR (162 MHz, Acetone-$d_6$) δ -143.68 (hept, *J* = 711.0 Hz).

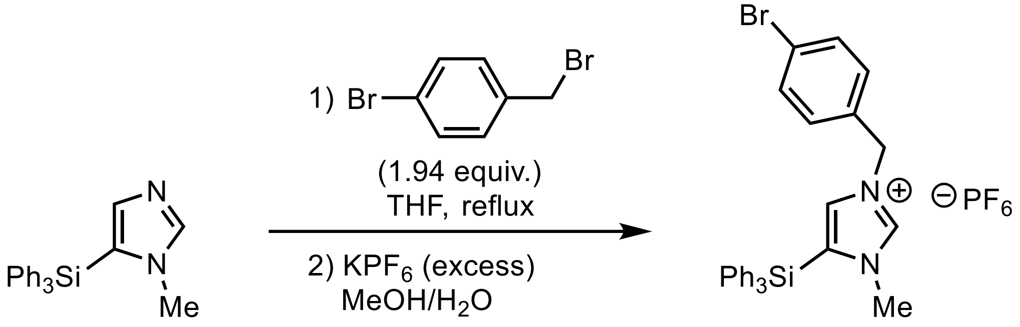


**S1** **1e**

**3-(4-bromobenzyl)-1-methyl-5-(triphenylsilyl)-1*H*-imidazol-3-ium (1e).** The title compound was prepared by benzylation of 1-methyl-5-(triphenylsilyl)-1*H*-imidazole **S1** (1.30 g, 3.83 mmol, 1.0 equiv.) with benzyl bromide (1.86 mL, 7.44 mmol, 1.94 equiv.) following a similar procedure as described for **1b** above. The title compound was isolated in 1.63 g (2.49 mmol, 65% yield) after recrystallization from hot EtOH/MeCN (9:1).

$^{1}$H NMR (400 MHz, Acetone-$d_6$) δ 9.32 (s, 1H), 7.72 (s, 1H), 7.70 – 7.65 (m, 6H), 7.64 – 7.56 (m, 5H), 7.51 (t, *J* = 7.2 Hz, 6H), 7.46 (d, *J* = 8.6 Hz, 2H), 5.61 (s, 2H), 3.67 (s, 3H).
$^{13}$C NMR (101 MHz, Acetone-$d_6$) δ 142.5, 136.7, 134.7, 134.4, 133.1, 132.2, 132.0, 131.7, 131.1, 129.6, 123.6, 52.8, 38.1.
$^{19}$F NMR (376 MHz, Acetone-$d_6$) δ -72.42 (d, *J* = 707.8 Hz).
$^{31}$P NMR (162 MHz, Acetone-$d_6$) -143.65 (hept, *J* = 705.6 Hz).

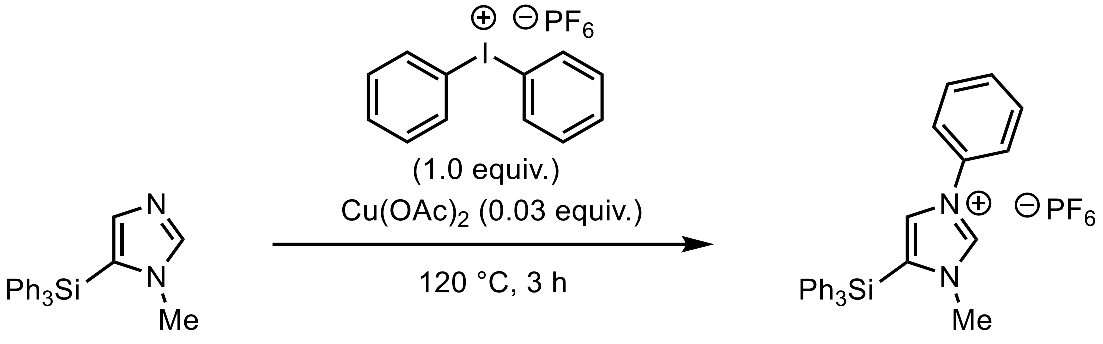

**S1** **1f**

**1-methyl-3-phenyl-5-(triphenylsilyl)-1*H*-imidazol-3-ium (1f).** To a flame-dried 2 dram vial were added 1-methyl-5-(triphenylsilyl)-1*H*-imidazole **S1** (300 mg, 0.88 mmol, 1.00 equiv.), diphenyliodonium hexafluorophosphate (375 mg, 0.88 mmol, 1.00 equiv.), and copper diacetate (4.8 mg, 0.026 mmol, 0.03 equiv.). The vial was sealed with a septum cap, then subjected to 5 cycles of evacuation and purging with nitrogen. Under counterflow of nitrogen, the perforated septum cap was replaced with a new PTFE-lined screw cap. The reaction mixture was heated at 120 °C for 3 hours, then cooled to ambient temperature. The residue was dissolved in a 1:1 mixture of DCM/MeCN (4 mL) and precipitated by addition of 50 mL diethyl ether. The precipitate was collected by vacuum filtration, then recrystallized from a boiling mixture of MeCN/EtOH (6:10) to yield the title compound in 180 mg (0.32 mmol, 36% yield).

$^{1}$H NMR (400 MHz, Acetone-$d_6$) δ 9.75 (s, 1H), 7.98 (s, 1H), 7.81 – 7.74 (m, 8H), 7.62 (dd, *J* = 15.1, 7.5 Hz, 6H), 7.54 (t, *J* = 7.0 Hz, 6H), 3.78 (s, 6H).
$^{13}$C NMR (101 MHz, Acetone-$d_6$) δ 141.4, 136.8, 135.8, 133.2, 132.7, 132.0, 131.1, 131.1, 131.0, 129.7, 38.3.
$^{19}$F NMR (376 MHz, Acetone-$d_6$) δ -72.44 (d, *J* = 707.8 Hz).
$^{31}$P NMR (162 MHz, Acetone-$d_6$) δ -143.66 (hept, *J* = 708.6 Hz).

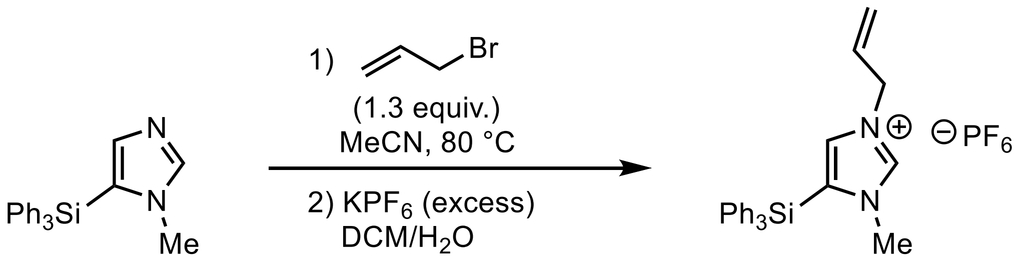

**S1** **1g**

**3-allyl-1-methyl-5-(triphenylsilyl)-1*H*-imidazol-3-ium hexafluorophosphate (1g).** The title compound was prepared by allylation of 1-methyl-5-(triphenylsilyl)-1*H*-imidazole **S1** (400 mg, 1.17 mmol, 1.00 equiv.) with allyl bromide (0.13 mL, 1.53 mmol, 1.30 equiv.) following a similar procedure as described for **1c** above. The title compound was isolated in 444 mg (0.84 mmol, 72% yield) after recrystallization from hot EtOH/MeOH (3:1).

$^{1}$H NMR (400 MHz, Acetone-$d_6$) δ 9.26 (s, 1H), 7.72 – 7.67 (m, 6H), 7.63 – 7.56 (m, 4H), 7.56 – 7.50 (m, 6H), 6.20 – 6.07 (m, 1H), 5.48 – 5.37 (m, 2H), 5.03 (d, *J* = 6.2 Hz, 2H), 3.69 (s, 3H).
$^{13}$C NMR (100 MHz, Acetone-$d_6$) δ 142.3, 136.7, 134.6, 132.0, 132.0 (d, *J* = 2.4 Hz), 131.2, 129.7, 121.5, 52.3, 38.0.
$^{19}$F NMR (376 MHz, Acetone-$d_6$) δ -72.47 (d, *J* = 707.7 Hz).
$^{31}$P NMR (162 MHz, Acetone-$d_6$) δ -143.66 (hept, *J* = 709.4 Hz).

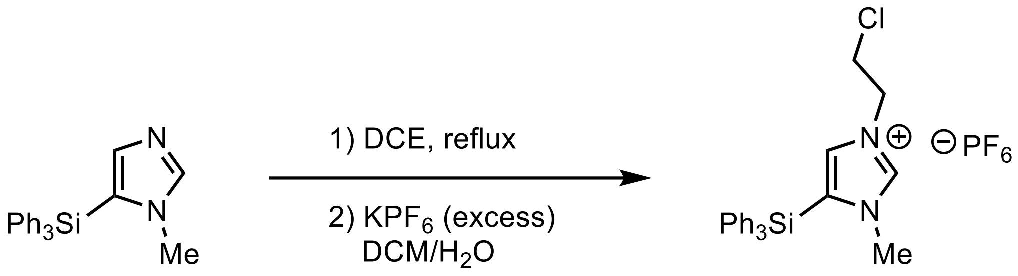


**S1** **1h**

**3-(2-chloroethyl)-1-methyl-5-(triphenylsilyl)-1*H*-imidazol-3-ium (1h).** In a 100 mL round bottom flask, 1-methyl-5-(triphenylsilyl)-1*H*-imidazole **S1** (400 mg, 1.17 mmol, 1.00 equiv.) was dissolved in 1,2-dichloroethane (20 mL) and heated at reflux for 20 hours. After cooling to room temperature, volatiles were removed, and the solid residue was washed with several portions of diethyl ether. The residue was redissolved in DCM (100 mL) and washed three times with a saturated aqueous solution of $KPF_6$. The organic phase was filtered through a pad of celite, concentrated in vacuo, and the resulting residue was recrystallized from boiling methanol to give the title compound in 250 mg (0.46 mmol, 39% yield).

$^1$H NMR (400 MHz, Acetone-$d_6$) δ 9.37 (s, 1H), 7.74 (d, *J* = 1.5 Hz, 1H), 7.72 – 7.67 (m, 6H), 7.64 – 7.58 (m, 3H), 7.56 – 7.51 (m, 6H), 4.81 (t, *J* = 5.6 Hz, 3H), 4.13 (t, *J* = 5.7 Hz, 4H), 3.72 (s, 3H).
$^{13}$C NMR (101 MHz, Acetone-$d_6$) δ 142.7, 136.7, 134.9, 132.0, 131.9, 131.1, 129.7, 51.7, 43.6, 38.1.
$^{19}$F NMR (376 MHz, Acetone-$d_6$) δ -72.46 (d, *J* = 707.8 Hz).
$^{31}$P NMR (162 MHz, Acetone-$d_6$) δ -143.68 (hept, *J* = 707.7 Hz).

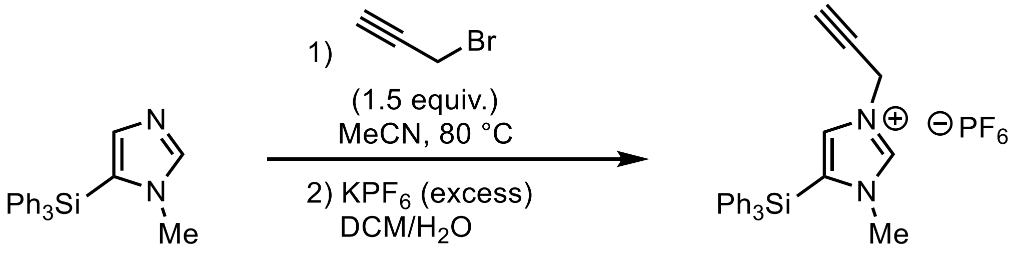


**S1** **1i**

**1-methyl-3-(prop-2-yn-1-yl)-5-(triphenylsilyl)-1*H*-imidazol-3-ium (1i).** The title compound was prepared by allylation of 1-methyl-5-(triphenylsilyl)-1*H*-imidazole **S1** (400 mg, 1.17 mmol, 1.00 equiv.) with propargyl bromide (0.19 mL, 1.76 mmol, 1.50 equiv.) following a similar procedure as described for **1c** above. The title compound was isolated in 464 mg (0.88 mmol, 75% yield) after recrystallization from hot EtOH/MeCN (3:1).

$^1$H NMR (400 MHz, Acetone-$d_6$) δ 9.40 (s, 1H), 7.74 – 7.67 (m, 6H), 7.64 – 7.58 (m, 4H), 7.56 – 7.48 (m, 6H), 5.31 (d, *J* = 2.6 Hz, 2H), 3.72 (s, 3H), 3.39 (t, *J* = 2.6 Hz, 1H).
$^{13}$C NMR (101 MHz, Acetone-$d_6$) δ 142.2, 136.7, 134.2, 132.3, 132.0, 131.0, 129.7, 78.8, 75.7, 39.7, 38.1.
$^{19}$F NMR (376 MHz, Acetone-$d_6$) δ -72.47 (d, *J* = 707.8 Hz).
$^{31}$P NMR (162 MHz, Acetone-$d_6$) δ -143.68 (hept, *J* = 707.7 Hz).

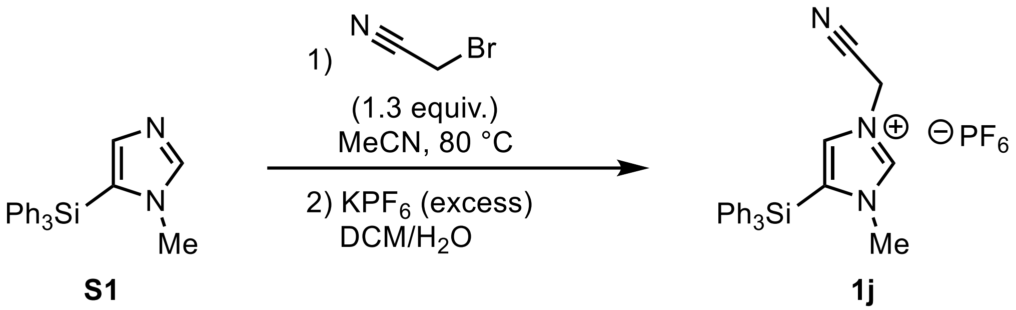

**1-methyl-3-(prop-2-yn-1-yl)-5-(triphenylsilyl)-1*H*-imidazol-3-ium (1j).** The title compound was prepared by allylation of 1-methyl-5-(triphenylsilyl)-1*H*-imidazole **S1** (400 mg, 1.17 mmol, 1.00 equiv.) with 2-bromoacetonitrile (0.11 mL, 1.53 mmol, 1.30 equiv.) following a similar procedure as described for **1c** above. The title compound was isolated in 464 mg (0.88 mmol, 75% yield) after recrystallization from hot EtOH/MeOH (3:1).

$^{1}$H NMR (400 MHz, Acetone-$d_6$) δ 9.52 (s, 1H), 7.80 (d, $J$ = 1.6 Hz, 1H), 7.75 – 7.67 (m, 6H), 7.65 – 7.58 (m, 3H), 7.57 – 7.51 (m, 6H), 5.71 (s, 2H), 3.75 (s, 3H).
$^{13}$C NMR (101 MHz, Acetone-$d_6$) δ 143.3, 136.7, 134.8, 132.8, 132.1, 130.8, 129.7, 114.5, 38.3, 37.7.
$^{19}$F NMR (376 MHz, Acetone-$d_6$) δ -72.48 (d, $J$ = 707.7 Hz).
$^{31}$P NMR (162 MHz, Acetone-$d_6$) δ -143.69 (hept, $J$ = 709.1 Hz).

## c) Synthesis of silylimidazolium tetrafluoroborate 10 and silylimidazolium hexafluoroantimonate 12

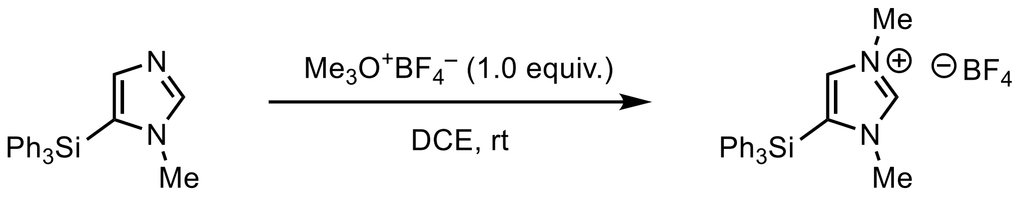


**S1** **10**

**1,3-dimethyl-5-(triphenylsilyl)-1H-imidazol-3-ium tetrafluoroborate (10).** To a flame-dried 16 mL vial was added 1-methyl-5-(triphenylsilyl)-1*H*-imidazole **S1** (200 mg, 0.59 mmol, 1.00 equiv.) and trimethyloxonium tetrafluoroborate (87 mg, 1.47 mmol, 1.00 equiv.), and the vial was purged with nitrogen. Anhydrous 1,2-dichloroethane (2.0 mL) was added, and the mixture was stirred at ambient temperature for 20 hours under nitrogen. Diethyl ether was layered on the crude reaction mixture, resulting in crystallization of a white solid, which was collected by vacuum filtration to afford the title compound **10** in 218 mg (0.49 mmol, 84% yield).

$^{1}$H NMR (400 MHz, Acetone-$d_6$) δ 9.18 (s, 1H), 7.73 – 7.65 (m, 6H), 7.65 – 7.51 (m, 3H), 7.57 – 7.47 (m, 7H), 4.05 (s, 3H), 3.65 (s, 3H).
$^{13}$C NMR (101 MHz, Acetone-$d_6$) δ 143.0, 136.7, 135.8, 131.9, 131.4, 131.3, 129.6, 37.7, 36.3.
$^{19}$F NMR (376 MHz, Acetone-$d_6$) δ -151.27 – -151.34 (m).
$^{11}$B {$^{1}$H, $^{13}$C} NMR (96 MHz, Acetone-$d_6$) δ -0.94.

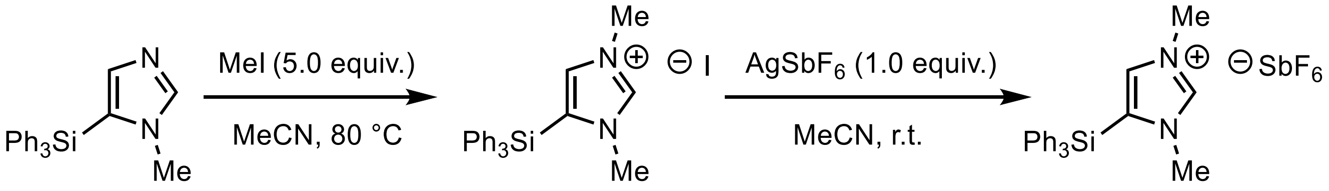


**S1** **12**

**1,3-dimethyl-5-(triphenylsilyl)-1*H*-imidazol-3-ium hexafluorostibate (12).** In a 2 dram vial, 1-methyl-5-(triphenylsilyl)-1*H*-imidazole **S1** (200 mg, 0.59 mmol, 1.00 equiv.) was dissolved in acetonitrile (2 mL). Iodomethane (0.19 mL, 2.94 mmol, 5.00 equiv.) was added, and the vial was sealed with a screw cap and heated at 80 °C for 20 hours. After cooling to room temperature, volatiles were removed, and the solid residue was washed with diethyl ether. A portion of the crude iodide salt (252 mg, 0.52 mmol) was dissolved in acetonitrile (20 mL), to which was added a solution of silver hexafluoroantimonate (179 mg, 0.52 mmol) in 20 mL acetonitrile, inducing precipitation of a faint yellow solid. After stirring for 30 minutes, the solid was removed by filtration through a pad of celite. The filtrate was concentrated in vacuo, taken up in boiling ethanol (40 ml), then quickly filtered to remove insoluble particulates. A white crystalline solid formed upon cooling the filtrate, which was collected by vacuum filtration to afford the title compound **12** in 216 mg (0.37 mmol, 63% yield over two steps).

$^{1}$H NMR (400 MHz, Acetone-$d_6$) δ 9.22 (s, 1H), 7.73 – 7.67 (m, 6H), 7.60 (t, $J$ = 7.4 Hz, 3H), 7.56 – 7.49 (m, 7H), 4.07 (s, 3H), 3.67 (s, 3H).
$^{13}$C NMR (101 MHz, Acetone-$d_6$) δ 142.7, 136.7, 135.9, 131.9, 131.6, 131.2, 129.6, 37.8, 36.4.
$^{19}$F NMR (376 MHz, Acetone-$d_6$) δ -101.91 – -141.11 (m).

## d) Synthesis of 5-silylimidazolium salts by lithiation-silylation of symmetrical imidazolium salts

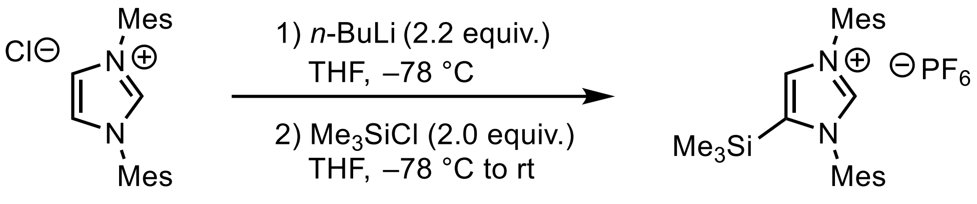

**1k**

**1,3-dimesityl-5-(trimethylsilyl)-1*H*-imidazol-3-ium hexafluorophosphate (1k).** To a flame-dried Schlenk flask 1,3-dimesityl-1H-imidazol-3-ium chloride (0.21 g, 0.62 mmol, 1.0 equiv.) and THF (6 mL) were added and the solution was sparged with nitrogen for 20 minutes while cooling to -78 °C. A solution of *n*-BuLi (0.60 mL, 1.3 mmol, 2.2 equiv.) in hexanes was added dropwise with stirring. After 30 minutes the reaction was allowed to warm to room temperature to increase solubility. After an additional 30 minutes, the reaction was cooled to -78 °C and chlorotrimethylsilane (0.16 mL, 1.3 mmol, 2.0 equiv.) was added and the solution stirred overnight while slowly warming to ambient temperature. The reaction mixture was quenched by addition of aqueous $NH_4PF_6$ in excess. The layers were separated and the aqueous phase was extracted three times with ethyl acetate. The combined organic extracts were washed with aqueous $KPF_6$, dried over $MgSO_4$, and concentrated in vacuo. The crude residue was purified via recrystallization from EtOH/MeCN to obtain the title compound 1k in 0.16 g (49% yield).

$^1$H NMR (400 MHz, Acetone-$d_6$) δ 9.46 (d, $J$ = 1.5 Hz, 1H), 8.20 (d, $J$ = 1.5 Hz, 1H), 7.26 (s, 2H), 7.20 (s, 2H), 2.41 (s, 3H), 2.38 (s, 3H), 2.21 (s, 8H), 2.20 (s, 7H), 0.22 (s, 8H).
$^{13}$C NMR (101 MHz, Acetone-$d_6$) δ 142.4 (d, $J$ = 53.4 Hz), 141.5, 137.9, 135.8 (d, $J$ = 70.8 Hz), 133.5, 132.2 (d, $J$ = 57.8 Hz), 130.5 (d, $J$ = 7.7 Hz), 21.1 (d, $J$ = 10.1 Hz), 17.5 (d, $J$ = 18.8 Hz), -1.7.
$^{19}$F NMR (376 MHz, Acetone-$d_6$) δ -72.43 (d, $J$ = 707.6 Hz).
$^{31}$P NMR (162 MHz, Acetone-$d_6$) δ -143.66 (hept, $J$ = 707.5 Hz).

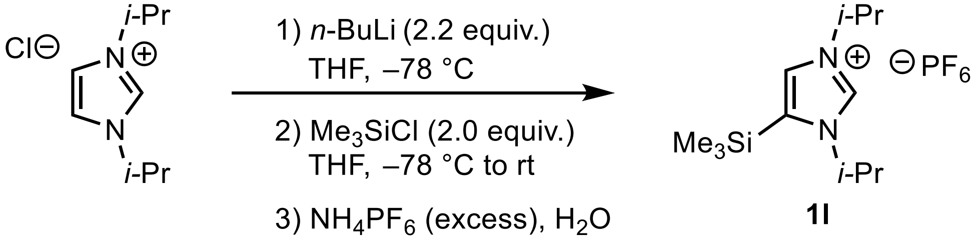

**1l**

**1,3-diisopropyl-5-(trimethylsilyl)-1H-imidazol-3-ium hexafluorophosphate (1l).** The title compound was prepared by silylation of 1,3-diisopropyl-1*H*-imidazol-3-ium chloride (0.54 g, 2.8 mmol, 1.0 equiv.) using *n*-BuLi (2.8 mL, 6.3 mmol, 2.2 equiv.) in hexanes, THF (29 mL) and chlorotrimethylsilane (0.72 mL, 5.7 mmol, 2.0 equiv.) following a similar procedure as described for **1k** above. The title compound was isolated in 33 mg (90 mmol, 3% yield) after recrystallization from EtOH/MeCN.

*Note: **1l** was isolated in insufficient quantity for full analytical characterization. $^1$H and $^{19}$F NMR spectral data are provided as confirmation of the identity and purity of **1l.***

$^1$H NMR (400 MHz, Acetone-$d_6$) δ 9.39 (s, 1H), 7.87 (s, 1H), 4.88 – 4.69 (m, 2H), 1.76 – 1.53 (m, 6H), 0.46 (s, 9H).
$^{19}$F NMR (376 MHz, Acetone-$d_6$) δ -72.47 (d, $J$ = 707.4 Hz).

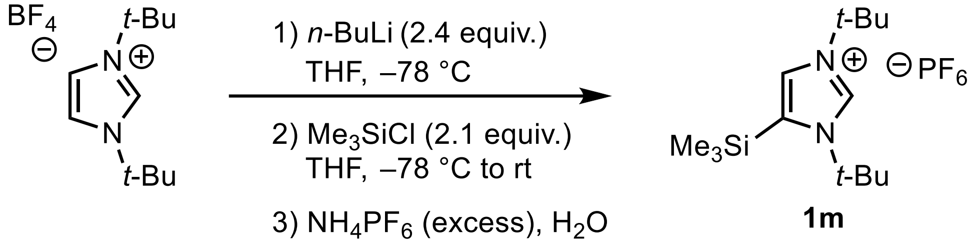

**1,3-di-tert-butyl-5-(trimethylsilyl)-1*H*-imidazol-3-ium hexafluorophosphate (1m).** The title compound was prepared by silylation of 1,3-di-tert-butyl-1*H*-imidazol-3-ium tetrafluoroborate (0.30 g,1.1 mmol, 1.0 equiv.) using *n*-BuLi (1.2 mL, 2.7 mmol, 2.4 equiv.) in hexanes, THF (11 mL) and chlorotrimethylsilane (0.30 mL, 2.4 mmol, 2.1 equiv.) following a similar procedure as described for **1k** above. The title compound was isolated in 0.25 g (0.62 mmol, 54% yield) after recrystallization from EtOH/MeCN.

$^{1}$H NMR (400 MHz, Acetone-$d_6$) δ 9.09 (s, 1H), 7.93 (s, 1H), 1.79 (s, 9H), 1.73 (s, 9H), 0.52 (s, 9H).
$^{13}$C NMR (101 MHz, Acetone-$d_6$) δ 136.8, 134.8, 130.9, 62.1, 60.9, 30.6, 29.5, 1.2.
$^{19}$F NMR (376 MHz, Acetone-$d_6$) δ -72.34 (d, $J$ = 707.7 Hz).
$^{31}$P NMR (162 MHz, Acetone-$d_6$) δ -143.67 (hept, $J$ = 707.6 Hz).

## 3. Synthesis and Characterization of NHC-$PF_5$ Adducts

### a) General procedure the preparation of NHC-PF5 adducts from silylimidazolium salts

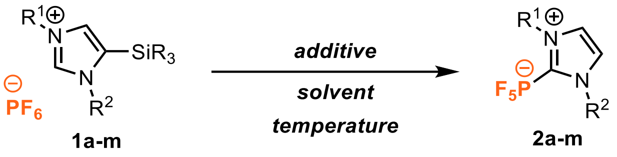

**General procedure for reaction setup:**

*Note: All optimization reactions were conducted on a 0.090 mmol scale and NMR yields were determined using dimethylsulfone as an internal standard. Preparative reactions were typically carried out with 0.15-0.20 mmol substrate.*

To an oven-dried screw cap test tube containing a PTFE-coated stir bar was added silylimidazolium hexafluorophosphate salt **1**. *If an additive was used, it was also added at this stage*. The test tube was then sealed with a PTFE-lined septum screw cap and evacuated by inserting a needle through the septum cap to connect to the vacuum manifold. The tube was submerged in a pre-heated oil bath set to 120 °C and subjected to five cycles of successive evacuation and refilling with $N_2$ to remove adventitious moisture. The tube was removed from the oil bath and cooled to room temperature. *If a solvent was used, it was added at this stage, then the tube was resubjected to three cycles of evacuation and refilling with $N_2$ at room temperature*. Under counterflow of $N_2$, the septum cap was replaced with a solid PTFE-lined screw cap, and the tube was submerged in an oil bath set to the specified reaction temperature (i.e., 160 °C, 180 °C, 190 °C, 200 °C, or 220 °C). After heating the reaction vessel for 24 hours, it was cooled to room temperature and unsealed in a fume hood under ambient atmosphere. The crude mixture was taken up in acetone, transferred to a separate vessel, then concentrated in vacuo for NMR analysis or purification by column chromatography.

**General procedure for purification:**

NHC-$PF_5$ adducts were isolated by silica gel chromatography using a Teledyne-Isco CombiFlash NextGen 300+ system with a gradient eluent from 100% hexanes to 100% EtOAc with UV-vis detection at 250 nm and 280 nm. Most NHC-$PF_5$ adducts in this study exhibit extremely low extinction coefficients at wavelengths greater than ca. 220 nm, rendering isolation from the eluted fractions challenging. We have found that the NHC-$PF_5$ adducts typically elute at or near an eluent strength of 75-100% EtOAc. We do not report $R_f$ values because simple NHC-$PF_5$ adducts could not be visualized by TLC using fluorescence quenching or staining with $KMnO_4$, anisaldehyde, or CAM stains. Staining of TLC plates with $I_2$ vapor revealed faint traces of the NHC-$PF_5$ compounds only after extended periods of staining (>3 hours).

In some cases, NHC-$PF_5$ adducts co-eluted with minor amounts of an inorganic hexafluorophosphate salt, as observed $^{19}F$ spectra. The source of $PF_6^-$ may be from **7** present in the crude reaction mixture, or it may result from slow degradation of the NHC-$PF_5$ adducts on silica gel. We believe the cation is either residual $Li^+$ when $LiPF_6$ was used as an additive in the reaction, or $Na^+$ present in the silica gel. The products for which we observed $PF_6^-$ in $^{19}F$ spectra are noted below. The $PF_6^-$ impurities are present in less than 5 mol%, unless otherwise noted. In these cases, the isolated yields reported in Figure 4 have been adjusted to account for the trace $PF_6^-$ impurities, assuming $NaPF_6$ as the contaminant. We found that simply washing with $H_2O$ is sufficient to completely remove $PF_6^-$ contamination, and the select examples where this was performed prior to collecting characterization data are noted below.

## b) **Preparation of NHC-$PF_5$ Adducts 2a-2h, 2k, and 2l**

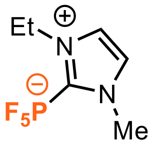

**(3-ethyl-1-methyl-1*H*-imidazol-3-ium-2-yl)pentafluorophosphate (2a).** Following the General Procedure with $Ph_2O$ as a solvent and heating to 220 °C, the title compound was obtained in 79% yield after purification by silica gel chromatography (hexanes/ethyl acetate = 100/0% to 0/100%). This isolated yield has been adjusted to account for trace hexafluorophosphate impurity (<5 mol%) observed in the fluorine NMR spectrum.

Samples of **2a** and **6a** for X-ray crystallography were obtained from the reaction of **1a** following the General Procedure with heating at 190 °C. Details regarding the purification and crystallization of **2a** and **6a** can be found in Section 6a) below.

$^{1}$H NMR (400 MHz, Acetone-$d_6$) δ 7.54 (t, 1H), 7.47 (t, 1H), 4.44 (q, *J* = 7.2 Hz, 2H), 3.97 (s, 3H), 1.42 (t, *J* = 7.2 Hz, 3H).
$^{13}$C NMR (101 MHz, Acetone-$d_6$) δ 124.4 (d, *J* = 9.9 Hz), 121.6 (d, *J* = 9.7 Hz), 46.0 (p, *J* = 4.0), 38.4 (p, *J* = 4.6 Hz), 34.8, 23.0, 16.5, 14.3. C(2) not observed above baseline noise.
$^{19}$F NMR (376 MHz, Acetone-$d_6$) δ -54.07 (dd, *J* = 777.2, 49.7 Hz), -73.84 (dp, *J* = 760.1, 49.7 Hz).
$^{31}$P NMR (162 MHz, Acetone-$d_6$) δ -150.47 (pd, *J* = 777.1, 759.9 Hz).
HRMS (ESI-TOFMS): $[M+Na]^+$ calculated for $[C_6H_{10}F_5N_2PNa]^+$ = 259.0394, found = 259.0395

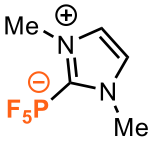

**(1,3-dimethyl-1H-imidazol-3-ium-2-yl)pentafluorophosphate (2c).** Following the General Procedure, with 0.1 equiv. $LiPF_6$, $Ph_2O$ as a solvent, and heating to 200 °C, the title compound was obtained in 64% yield after purification by silica gel chromatography (hexanes/ethyl acetate = 100/0% to 0/100%). This isolated yield has been adjusted to account for trace hexafluorophosphate impurity (<5 mol%) observed in the fluorine NMR spectrum. The NMR spectra of **2c** below were collected after the material isolated by chromatography was washed with water.

$^{1}$H NMR (400 MHz, Acetone-$d_6$) δ 7.43 (d, *J* = 3.0 Hz, 2H), 3.97 (s, 6H).
$^{13}$C NMR (101 MHz, Acetone-$d_6$) δ 123.7 (d, *J* = 9.6 Hz), 38.4 (p, *J* = 4.4 Hz). C(2) not observed above baseline noise.
$^{19}$F NMR (376 MHz, Acetone-$d_6$) δ -55.07 (dd, *J* = 775.5, 49.6 Hz), -74.07 (dp, *J* = 761.1, 49.6 Hz).
$^{31}$P NMR (162 MHz, Acetone-$d_6$) δ -150.74 (pd, *J* = 775.5, 761.1 Hz).
HRMS (ESI-TOFMS): $[M+Na]^+$ calculated for $[C_5H_8F_5N_2PNa]^+$ = 245.0237, found = 245.0237

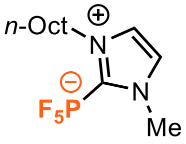

**(1-methyl-3-octyl-1*H*-imidazol-3-ium-2-yl)pentafluorophosphate (2d).** Following the General Procedure with $Ph_2O$ as a solvent and heating to 220 °C, the title compound was obtained in 79% yield after purification by silica gel chromatography (hexanes/ethyl acetate = 100/0% to 0/100%).

$^{1}$H NMR (400 MHz, Acetone-$d_6$) δ 7.54 (t, 1H), 7.46 (t, 1H), 4.37 (t, 2H), 3.97 (s, 3H), 1.84 (p, *J* = 7.9 Hz, 2H), 1.44 – 1.19 (m, 10H), 0.87 (t, 3H).

$^{13}$C NMR (101 MHz, Acetone-$d_6$) δ 158.9 – 152.8 (m, C(2) not resolved above baseline), 124.3 (d, $J$ = 9.9 Hz), 121.9 (d, $J$ = 9.6 Hz), 50.8 (p, $J$ = 3.9 Hz), 38.4 (p, $J$ = 4.1 Hz), 32.5, 31.7, 29.8, 27.2, 23.3, 14.3.
$^{19}$F NMR (376 MHz, Acetone-$d_6$) δ -53.81 (dd, $J$ = 777.9, 49.9 Hz), -73.72 (dp, $J$ = 760.1, 49.9 Hz).
$^{31}$P NMR (162 MHz, Acetone-$d_6$) δ -150.48 (pd, $J$ = 777.9, 760.1 Hz).
HRMS (ESI-TOFMS): $[M+Na]^+$ calculated for $[C_{12}H_{22}F_5N_2PNa]^+$ = 343.1333, found = 343.1329

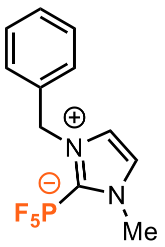


**(3-benzyl-1-methyl-1H-imidazol-3-ium-2-yl)pentafluorophosphate (2b).** When conducted on a 0.2 mmol scale: following the General Procedure with $Ph_2O$ as a solvent and heating to 220 °C, the title compound was obtained in 75% yield after purification by silica gel chromatography (hexanes/ethyl acetate = 100/0% to 0/100%).

When conducted on a 1 mmol scale: Following the General Procedure, using **1b** (0.577 g, 1.0 mmol, 1.0 equiv.) and $Ph_2O$ (1.077 g) as a solvent with heating to 220 °C, the title compound was isolated in 0.189 g (0.633 mmol, 63% yield) after purification by silica gel chromatography (hexanes/ethyl acetate = 100/0% to 0/100%).

$^{1}$H NMR (400 MHz, Chloroform-$d$) δ 7.39 (d, $J$ = 4.8 Hz, 3H), 7.34 – 7.29 (m, 2H), 6.88 (s, 1H), 6.72 (s, 1H), 5.53 (s, 2H), 4.01 (s, 3H).
$^{13}$C NMR (101 MHz, Acetone-$d_6$) δ 155.9 (dp, $J$ = 324.6, 66.1 Hz), 136.7, 129.7, 129.1, 128.9, 124.6 (d, $J$ = 9.2 Hz), 122.1 (d, $J$ = 9.2 Hz), 53.8 (p, $J$ = 3.9 Hz), 38.6 (p, $J$ = 4.6 Hz).
$^{19}$F NMR (376 MHz, Chloroform-$d$) δ -53.38 (dd, $J$ = 778.9, 52.2 Hz), -74.57 (dp).
$^{31}$P NMR (162 MHz, Chloroform-$d$) δ -150.75 (dp, $J$ = 778.9, 766.7 Hz).
HRMS (ESI-TOFMS): $[M+Na]^+$ calculated for $[C_{11}H_{12}F_5N_2PNa]^+$ = 321.0550, found = 321.0553

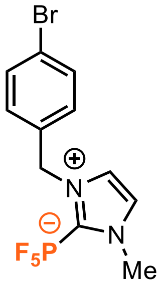


**(3-(4-bromobenzyl)-1-methyl-1H-imidazol-3-ium-2-yl)pentafluorophosphate (2e).** Following the General Procedure, with 0.1 equiv. $LiPF_6$, $Ph_2O$ as a solvent, and heating to 200 °C, the title compound was obtained in 76% yield after purification by silica gel chromatography (hexanes/ethyl acetate = 100/0% to 0/100%).

$^{1}$H NMR (400 MHz, Acetone-$d_6$) δ 7.58 (dt, $J$ = 4.5, 2.5 Hz, 2H), 7.51 (t, $J$ = 2.3 Hz, 1H), 7.34 (t, $J$ = 2.3 Hz, 1H), 7.27 (d, $J$ = 8.7 Hz, 2H), 5.61 (s, 2H), 4.03 (s, 3H).
$^{13}$C NMR (101 MHz, Acetone-$d_6$) δ 136.3, 132.7, 130.9, 124.8 (d, $J$ = 10.2 Hz), 122.6, 122.2 (d, $J$ = 9.3 Hz), 53.1 (p, $J$ = 4.1 Hz), 38.6 (p, $J$ = 4.4 Hz). C(2) not observed above baseline noise.
$^{19}$F NMR (376 MHz, Acetone-$d_6$) δ -53.15 (dd, $J$ = 777.1, 50.4 Hz), -74.01 (dp, $J$ = 763.7, 50.3 Hz).
$^{31}$P NMR (162 MHz, Acetone-$d_6$) δ -150.68 (pd, $J$ = 777.1, 763.6 Hz).
HRMS (ESI-TOFMS): $[M+Na]^+$ calculated for $[C_{11}H_{11}BrF_5N_2PNa]^+$ = 398.9656, found = 398.9655

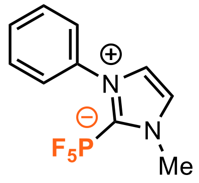

**(1-methyl-3-phenyl-1H-imidazol-3-ium-2-yl)pentafluorophosphate (2f).** Following the General Procedure with $Ph_2O$ as a solvent and heating to 200 °C, the title compound was obtained in 48% yield after purification by silica gel chromatography (hexanes/ethyl acetate = 100/0% to 0/100%). This isolated yield has been adjusted to account for ~45% hexafluorophosphate impurity observed in the fluorine NMR spectrum. The NMR spectra of **2f** below were collected after the material isolated by chromatography was washed with water.

$^1$H NMR (400 MHz, Acetone-*d*$_6$) δ 7.63 (t, 1H), 7.56 – 7.45 (m, 4H), 7.41 (d, *J* = 7.3 Hz, 2H), 4.10 (s, 3H).
$^{13}$C NMR (101 MHz, Acetone-*d*$_6$) δ 139.7, 130.3, 129.2, 128.5, 124.6 (d, *J* = 8.8 Hz), 124.0 (d, *J* = 9.3 Hz), 38.8 (t, *J* = 4.4 Hz), 23.3, 14.4. C(2) not observed above baseline noise.
$^{19}$F NMR (376 MHz, Acetone-*d*$_6$) δ -51.34 (dd, *J* = 779.3, 51.7 Hz), -74.70 (dp, *J* = 760.3, 51.7 Hz).
$^{31}$P NMR (162 MHz, Acetone-*d*$_6$) δ -150.69 (pd, *J* = 779.2, 760.0 Hz).
HRMS (ESI-TOFMS): $[M+Na]^+$ calculated for $[C_{10}H_{10}F_5N_2PNa]^+$ = 307.0394, found = 307.0392

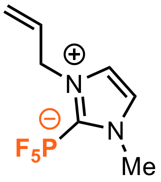

**(3-allyl-1-methyl-1H-imidazol-3-ium-2-yl)pentafluorophosphate (2g).** Following the General Procedure, with 0.1 equiv. $LiPF_6$, $Ph_2O$ as a solvent, and heating to 200 °C, the title compound was obtained in 77% yield after purification by silica gel chromatography (hexanes/ethyl acetate = 100/0% to 0/100%).

$^1$H NMR (400 MHz, Acetone-*d*$_6$) δ 7.48 (t, *J* = 2.5 Hz, 1H), 7.43 (t, *J* = 2.6 Hz, 1H), 6.02 (ddt, *J* = 16.7, 10.1, 6.2 Hz, 1H), 5.33 (dd, *J* = 32.1, 1.3 Hz, 1H), 5.32 (dd, *J* = 4.7, 1.3 Hz, 1H), 5.01 (d, *J* = 5.7 Hz, 2H), 3.99 (s, 3H).
$^{13}$C NMR (101 MHz, Acetone-*d*$_6$) δ 158.9 – 152.5 (m, C(2) not resolved above baseline), 133.7, 124.4 (d, *J* = 9.9 Hz), 122.0 (d, *J* = 9.3 Hz), 120.2, 53.1 (p, *J* = 4.2 Hz), 38.5 (p, *J* = 4.3 Hz).
$^{19}$F NMR (376 MHz, Acetone-*d*$_6$) δ -53.71 (dd, *J* = 776.9, 50.1 Hz), -73.86 (dp, *J* = 761.7, 50.1 Hz).
$^{31}$P NMR (162 MHz, Acetone-*d*$_6$) δ -150.60 (pd, *J* = 776.9, 761.5 Hz).
HRMS (ESI-TOFMS): $[M+Na]^+$ calculated for $[C_7H_{10}F_5N_2PNa]^+$ = 271.0394, found = 271.0394

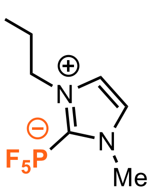

**(3-(2-chloroethyl)-1-methyl-1H-imidazol-3-ium-2-yl)pentafluorophosphate (2h).** Following the General Procedure, with 0.1 equiv. $LiPF_6$, $Ph_2O$ as a solvent, and heating to 180 °C, the title compound was obtained in pure form in 17% yield after purification by silica gel chromatography (hexanes/ethyl acetate = 100/0% to 0/100%). The first elution contained **2h** with trace hexafluorophosphate impurity (<5 mol%) observed in the fluorine NMR spectrum that have been accounted for in the 17% isolated yield. The second elution contained a mixture of **2h** and **2h'** in a ratio of approximately 1:4 (see Figure S2 below).

*Characterization data for **2h**:*
$^1$H NMR (400 MHz, Acetone-*d*$_6$) δ 7.63 (t, *J* = 3.0 Hz, 1H), 7.52 (t, *J* = 2.4 Hz, 1H), 4.77 (t, *J* = 6.3 Hz, 2H), 4.01 (s, 3H), 3.99 (t, *J* = 6.2 Hz, 2H).

$^{13}$C NMR (101 MHz, Acetone-$d_6$) δ 124.1 (d, $J$ = 8.5 Hz), 123.1 (d, $J$ = 8.8 Hz), 52.0 – 51.8 (m), 43.4, 38.8 – 38.5 (m). C(2) not observed above baseline noise.
$^{19}$F NMR (376 MHz, Acetone-$d_6$) δ -53.73 (dd, $J$ = 776.9, 49.8 Hz), -74.25 (dp, $J$ = 764.0, 49.8 Hz).
$^{31}$P NMR (162 MHz, Acetone-$d_6$) δ -150.64 (pd, $J$ = 777.1, 12.3 Hz).
HRMS (ESI-TOFMS): $[M+Na]^+$ calculated for $[C_6H_9ClF_5N_2PNa]^+$ = 293.0004, found = 293.0000

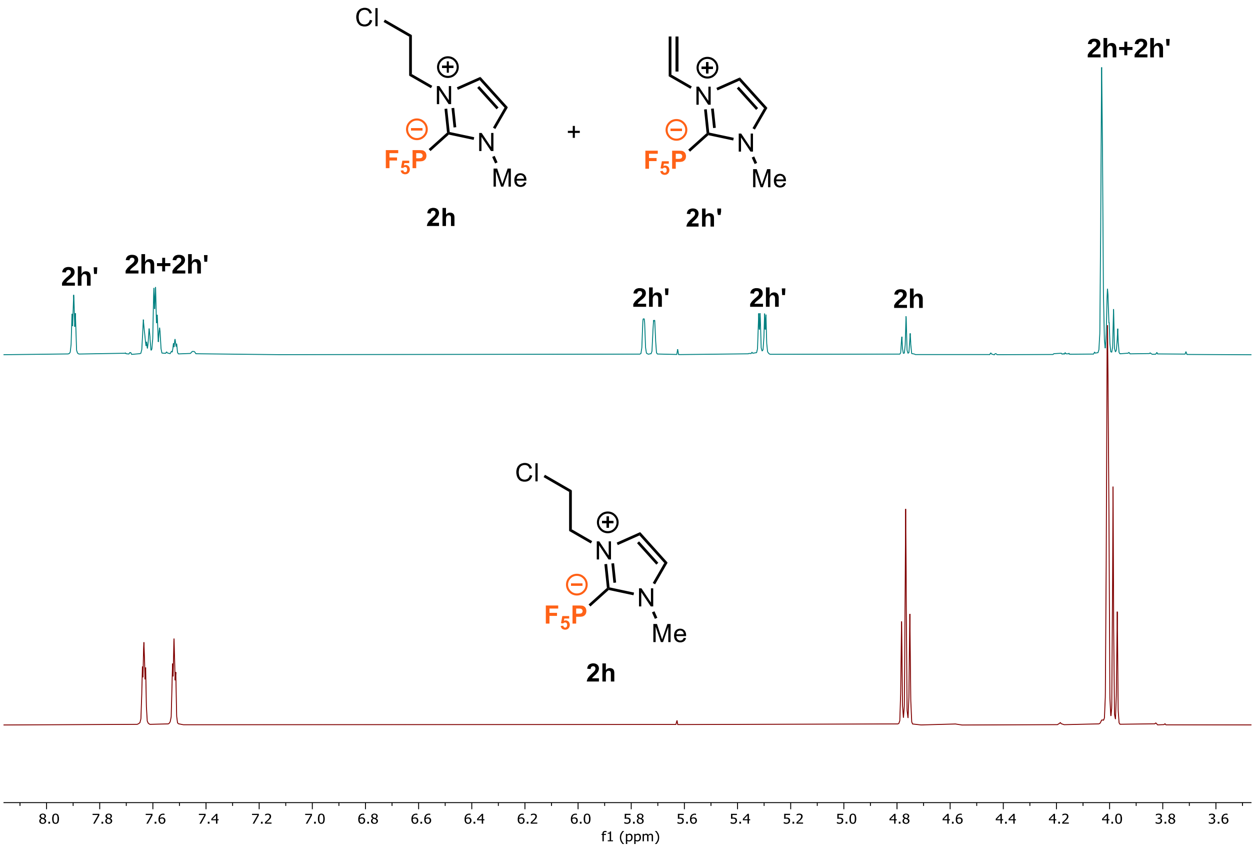


**Figure S2.** $^1$H NMR spectrum (400 MHz) in acetone-$d_6$ showing **2h** as a pure isolate obtained from column chromatography (bottom, red trace) along with a mixed fraction containing **2h** and vinyl NHC-$PF_5$ adduct **2h'** (top, green trace). The chemical shifts for **2h'** match reported NMR spectral data in the literature.[4] Based on relative integrations, the ratio of **2h:2h'** in the mixed fraction is ~1:4.

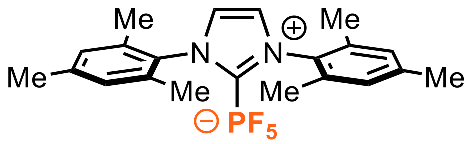


**(1,3-dimesityl-1H-imidazol-3-ium-2-yl)pentafluorophosphate (2k).** Following the General Procedure with $Ph_2O$ as a solvent and heating to 200 °C, the title compound was obtained in 44% yield after purification by silica gel chromatography (hexanes/ethyl acetate = 100/0% to 0/100%). Trace hexafluorophosphate impurity (<1 mol%) was observed in the fluorine NMR spectrum, which does not impact the isolated yield.

$^1$H NMR (400 MHz, Acetone-$d_6$) δ 7.62 (d, $J$ = 2.5 Hz, 2H), 7.03 (s, 4H), 2.34 (s, 6H), 2.08 (s, 12H).
$^{13}$C NMR (101 MHz, Acetone-$d_6$) δ 140.0, 136.0, 135.8, 129.4, 124.7 (d, $J$ = 9.6 Hz), 21.0, 17.6 (t, $J$ = 1.7 Hz).
$^{19}$F NMR (376 MHz, Acetone-$d_6$) δ -53.94 (dd, $J$ = 787.1, 52.7 Hz), -76.11 (dp, $J$ = 754.2, 52.7 Hz).
$^{31}$P NMR (162 MHz, Acetone-$d_6$) δ -151.58 (pd, $J$ = 787.0, 754.1 Hz). C(2) not observed above baseline noise.
HRMS (ESI-TOFMS): $[M+Na]^+$ calculated for $[C_{21}H_{24}F_5N_2PNa]^+$ = 453.1489, found = 453.1485

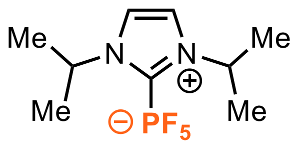

**(1,3-diisopropyl-1H-imidazol-3-ium-2-yl)pentafluorophosphate (2l).** Following the General Procedure with $Ph_2O$ as a solvent and heating to 200 °C, the title compound was obtained in 35% yield after purification by silica gel chromatography (hexanes/ethyl acetate = 100/0% to 0/100%).

$^1H$ NMR (400 MHz, Acetone-$d_6$) δ 7.77 (d, $J$ = 3.2 Hz, 2H), 5.37 (hept, $J$ = 6.7 Hz, 2H), 1.47 (s, 6H), 1.46 (s, 6H).
$^{13}C$ NMR (101 MHz, Acetone-$d_6$) δ 119.3 (d, $J$ = 9.6 Hz), 52.4 (p, $J$ = 4.6 Hz), 23.2.
$^{19}F$ NMR (376 MHz, Acetone-$d_6$) δ -52.87 (dd, $J$ = 780.5, 49.9 Hz), -73.33 (dp, $J$ = 759.6, 49.9 Hz).
$^{31}P$ NMR (162 MHz, Acetone-$d_6$) δ -149.86 (pd, $J$ = 780.6, 759.6 Hz). C(2) not observed above baseline noise.
HRMS (ESI-TOFMS): $[M+Na]^+$ calculated for $[C_9H_{16}F_5N_2PNa]^+$ = 301.0863, found = 301.0858

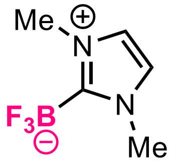

**(1,3-dimethyl-1*H*-imidazol-3-ium-2-yl)trifluoroborate (11).** Using a similar protocol to the General Procedure described above, compound **10** was heated at 200 °C for 24 hours with $Ph_2O$ as a solvent, and the title compound was obtained in 72% yield after purification by silica gel chromatography (hexanes/ethyl acetate = 100/0% to 0/100%). Trace tetrafluoroborate impurity (<1 mol%) was observed in the fluorine NMR spectrum, which does not impact the isolated yield. The NMR spectra of **11** below were collected after the material isolated by chromatography was washed with water.

$^1H$ NMR (400 MHz, Acetone-$d_6$) δ 7.34 (s, 2H), 3.89 (s, 6H).
$^{13}C$ NMR (101 MHz, Acetone-$d_6$) δ 123.2, 36.4. C(2) not observed above baseline noise.
$^{19}F$ NMR (376 MHz, Acetone-$d_6$) δ -139.21 (q, $J$ = 35.8 Hz).
$^{11}B$ {$^1H$, $^{13}C$} NMR (96 MHz, Acetone-$d_6$) δ -0.26 (q, $J$ = 36.1 Hz).
HRMS (ESI-TOFMS): $[M+Na]^+$ calculated for $[C_5H_8BF_3N_2Na]^+$ = 187.0626, found = 187.0625

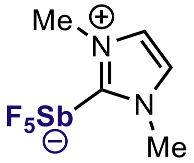

**(1,3-dimethyl-1H-imidazol-3-ium-2-yl)pentafluorostibate (13).** Using a similar protocol to the General Procedure described above, compound **12** was heated neat at 180 °C for 24 hours, and the title compound was obtained in 37% yield after purification by silica gel chromatography (hexanes/ethyl acetate = 100/0% to 0/100%). X-ray crystallography studies are described in Section 6.

*Caution: $SbF_5$, which is highly toxic, may be formed in this reaction. To quench any residual $SbF_5$, $CaCO_3$ was added after dissolving the crude reaction mixture in acetone, then removed by filtration prior to purification by column chromatography.*

$^1H$ NMR (400 MHz, Acetone-$d_6$) δ 7.80 (s, 2H), 4.14 (s, 6H).
$^{13}C$ NMR (101 MHz, Acetone-$d_6$) δ 126.9, 38.5. C(2) not observed above baseline noise.
$^{19}F$ NMR (376 MHz, Acetone-$d_6$) δ -101.68, -118.21.
HRMS (ESI-TOFMS): $[M+Na]^+$ calculated for $[C_5H_8F_5N_2SbNa]^+$ = 334.9538, found = 334.9538

## c) Unsuccessful substrates

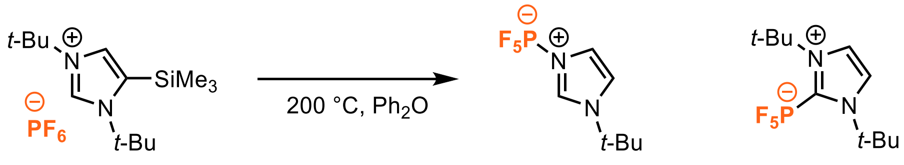


**1m** **2m'** 67% yield **2m** *not observed*

**(2-tert-butyl-1*H*-imidazole-κ$N^3$)pentafluorophosphorus (2m').** Following the General Procedure with $Ph_2O$ as a solvent and heating to 200 °C, compound **2m'** was obtained in 67% yield after purification by silica gel chromatography (hexanes/ethyl acetate = 100/0% to 0/100%). We did not observe evidence of **2m** in crude NMR spectra.

$^1$H NMR (400 MHz, Acetone-$d_6$) δ 8.61 (d, $J$ = 1.9 Hz, 1H), 7.70 (s, 1H), 7.49 (d, $J$ = 2.1 Hz, 1H), 1.75 (s, 9H).
$^{13}$C NMR (101 MHz, Acetone-$d_6$) δ 134.7, 131.8 (d, $J$ = 2.8 Hz), 129.7 (d, $J$ = 6.8 Hz), 129.3 (d, $J$ = 3.3 Hz), 124.1 (d, $J$ = 2.9 Hz), 118.8 (d, $J$ = 8.2 Hz), 59.8, 29.8.
$^{19}$F NMR (376 MHz, Acetone-$d_6$) δ -60.28 (dd, $J$ = 759.3, 56.1 Hz), -80.04 (dp, $J$ = 755.2, 56.1 Hz).
$^{31}$P NMR (162 MHz, Acetone-$d_6$) δ -149.10 (h, $J$ = 751.3 Hz).
HRMS (ESI-TOFMS): $[M+Na]^+$ calculated for $[C_7H_{12}F_5N_2PNa]^+$ = 273.0550, found = 273.0547

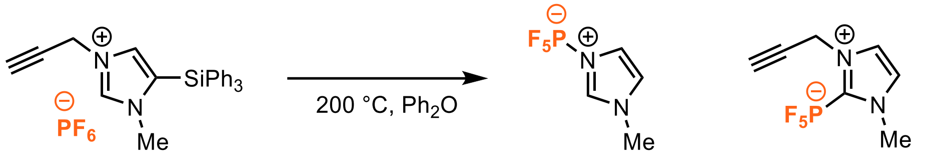


**1i**

**2i'**
*possible product*

**2i**
*not observed*

Compound **1i** was reacted following the General Procedure, with 0.1 equiv. $LiPF_6$, $Ph_2O$ as a solvent, and heating to 180 °C. The crude reaction mixture showed no evidence of the desired product **2i** by NMR spectral analysis. The $^{19}F$ NMR spectrum is shown in Figure S3 below.

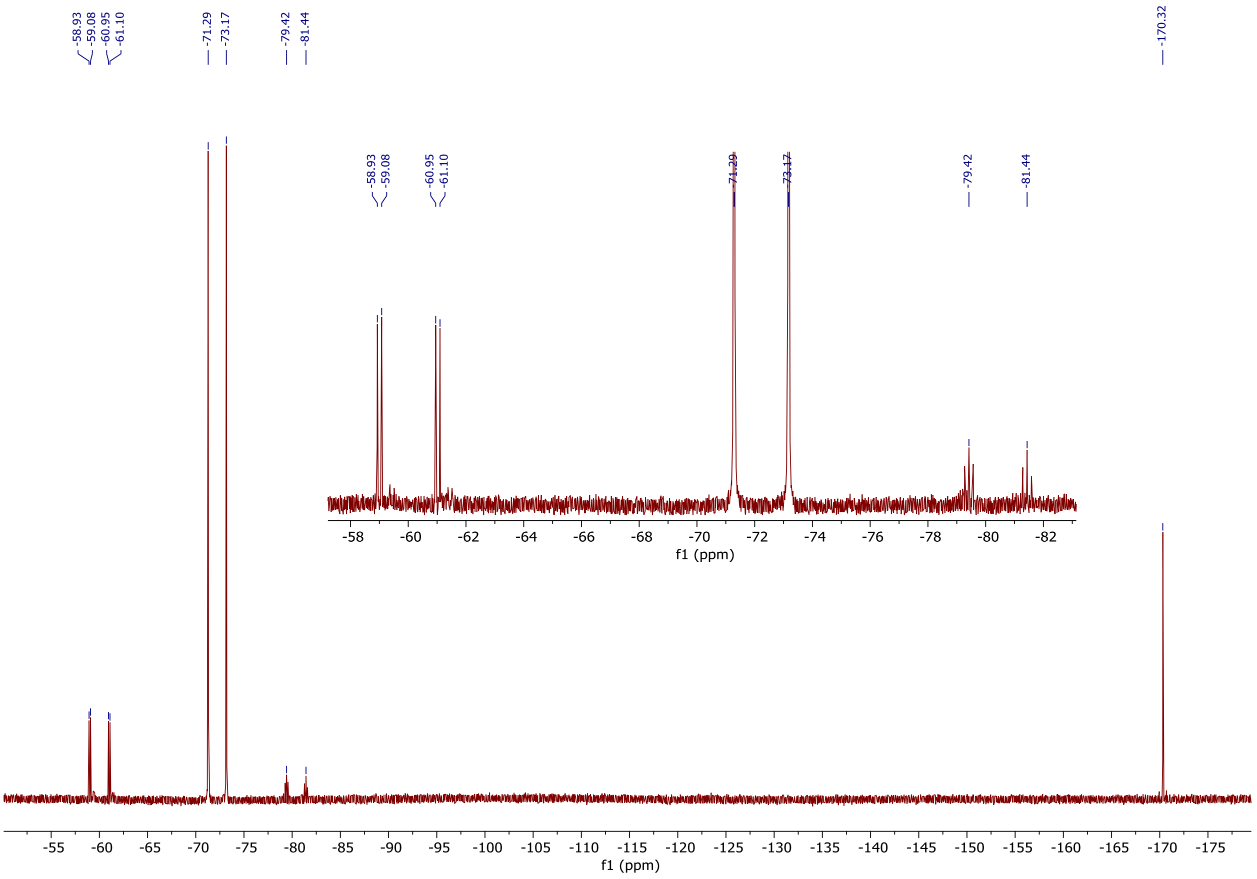


**Figure S3.** $^{19}F$ NMR spectrum (376 MHz) in $CDCl_3$ of the crude reaction mixture of **1i**. The signals at -60.02 (dd, $J$ = 760, 56.3 Hz) and -80.43 (dp, $J$ = 758, 56.6 Hz) are similar to those observed for the isolated *N*-$PF_5$ adduct **2m’**, suggesting **2i’** as a possible product. The peak at -170.32 ppm corresponds to $Ph_3SiF$.

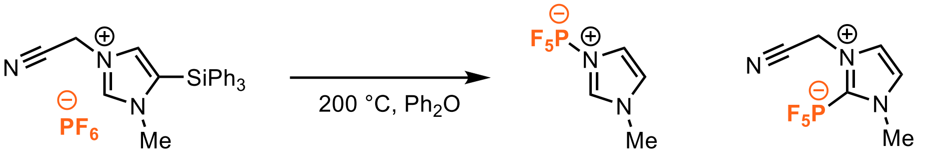


**1j** **2j'** *possible product* **2j** *not observed*

Compound **1j** was reacted following the General Procedure, with 0.1 equiv. $LiPF_6$, $Ph_2O$ as a solvent, and heating to 180 °C. The crude reaction mixture showed no evidence of the desired product **2j** by NMR spectral analysis. The $^{19}F$ NMR spectrum is shown in Figure S4 below.

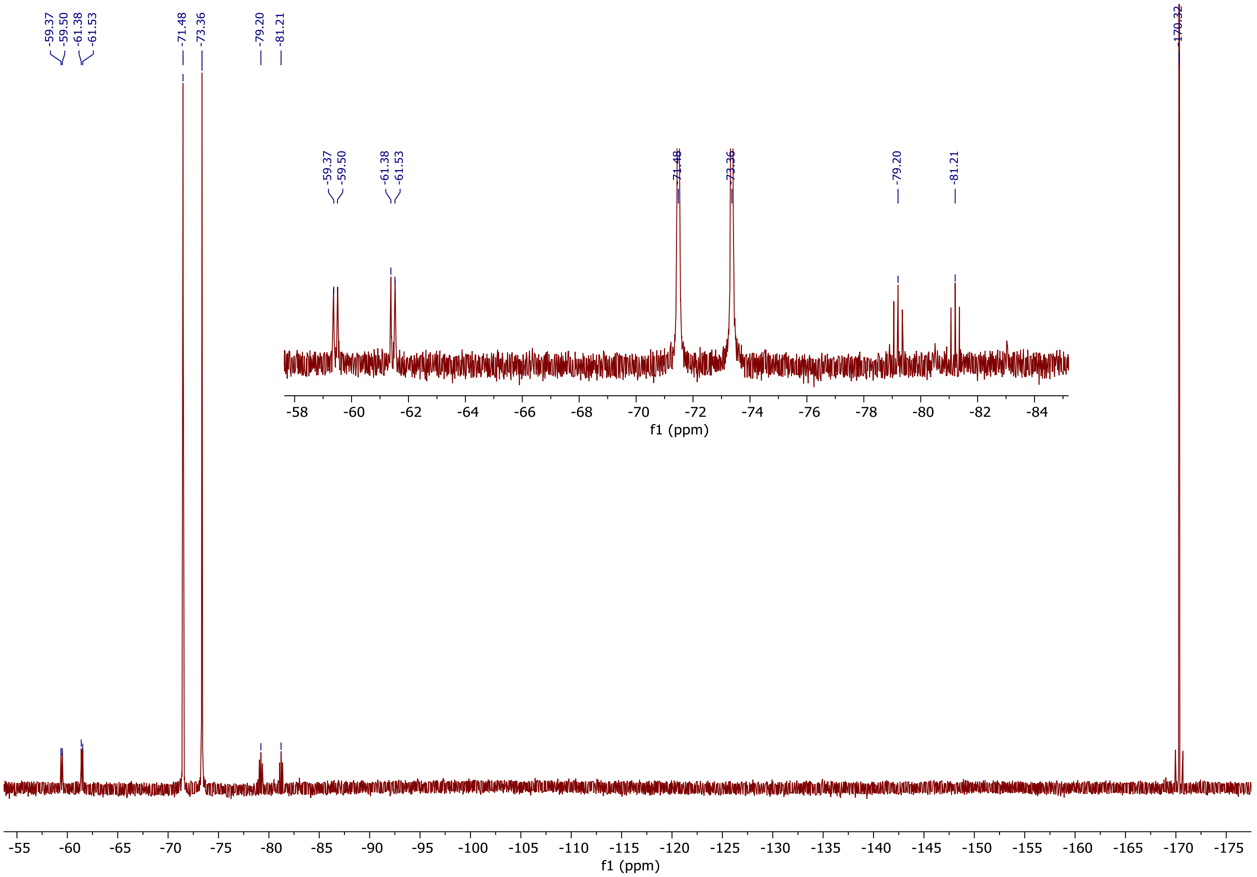


**Figure S4.** $^{19}F$ NMR spectrum (376 MHz) in $CDCl_3$ of the crude reaction mixture of **1j**. The signals at -60.45 (dd, $J$ = 758, 51.9 Hz) and -80.20 (dp, $J$ = 755, 56.2 Hz) are similar to those observed for the isolated *N*-$PF_5$ adduct **2m'**, suggesting **2j'** as a possible product, albeit in very minor amounts. The peak at -170.32 ppm corresponds to $Ph_3SiF$.

## 4. Derivatization of NHC-$PF_5$ adducts 2e and 2g

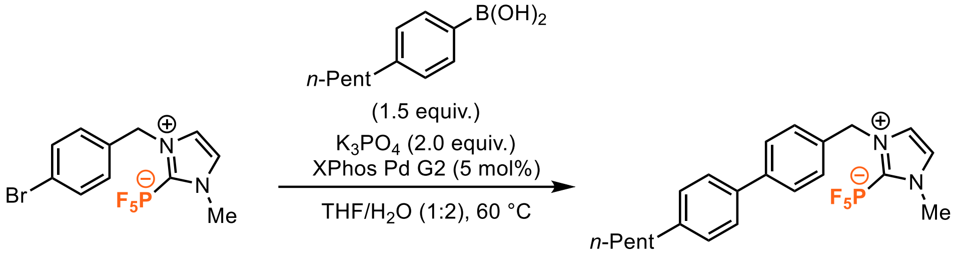


**2e** **8**

**Pentafluoro(1-methyl-3-((4'-pentyl-[1,1'-biphenyl]-4-yl)methyl)-1H-imidazol-3-ium-2-yl)phosphate(V) (8).** To a Schlenk tube was added NHC-$PF_5$ adduct **2e** (200 mg, 0.53 mmol, 1.00 equiv.), 4-pentylphenylboronic acid (153 mg, 0.80 mmol, 1.50 equiv.), and XPhos Pd G2 (chloro(2-dicyclohexylphosphino-2',4',6'-tri-iso-propyl-1,1'-biphenyl)(2'-amino-1,1'-biphenyl-2-yl) palladium(II), 21 mg, 27 mmol, 0.05 equiv.), and the vessel was subjected to three cycles of evacuation and purging with $N_2$. Pre-sparged THF (1.1 mL) was added, followed by a pre-sparged aqueous solution of 0.5 M $K_3PO_4$ (2.1 mL, 1.1 mmol, 2 equiv.). The reaction mixture was stirred with heating at 60 °C under $N_2$ for 16 hours. After cooling to room temperature, the reaction mixture was diluted with 15 mL water and 30 mL EtOAc, then passed through a short pad of celite. The layers were separated, and the aqueous phase was extracted two times with EtOAc (30 mL). The combined extracts were dried with $MgSO_4$, filtered, and concentrated in vacuo. The title compound was obtained in 213 mg (90% yield) as a white solid after purification by column chromatography on silica gel ($R_f$ = 0.3 at 50% hexanes/EtOAc).

$^1$H NMR (400 MHz, Acetone-$d_6$) δ 7.68 (d, $J$ = 8.4 Hz, 2H), 7.58 (d, $J$ = 8.2 Hz, 2H), 7.49 (t, $J$ = 2.5 Hz, 1H), 7.41 (d, $J$ = 8.2 Hz, 2H), 7.34 – 7.25 (m, 3H), 5.66 (s, 2H), 4.04 (s, 3H), 2.65 (t, $J$ = 7.7 Hz, 2H), 1.64 (p, 2H), 1.43 – 1.25 (m, 4H), 0.88 (t, 3H).
$^{13}$C NMR (101 MHz, Acetone-$d_6$) δ 159.1 – 152.8 (m, C(2) not resolved above baseline), 143.2, 141.8, 138.4, 135.5, 129.8, 129.5, 127.9, 127.6, 124.6 (d, $J$ = 9.8 Hz), 122.1 (d, $J$ = 9.2 Hz), 53.5 (p, $J$ = 4.2 Hz), 38.6 (p, $J$ = 4.2 Hz), 36.0, 32.2, 32.0, 23.2, 14.3.
$^{19}$F NMR (376 MHz, Acetone-$d_6$) δ -53.08 (dd, $J$ = 777.5, 50.3 Hz), -73.87 (dp, $J$ = 762.9, 50.2 Hz).
$^{31}$P NMR (162 MHz, Acetone-$d_6$) δ -148.87 (dp).
HRMS (ESI-TOFMS): $[M+Na]^+$ calculated for $[C_{22}H_{26}F_5N_2PNa]^+$ = 467.1646, found = 467.1643

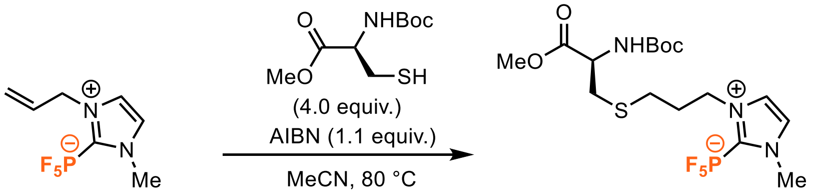


**2g** **9**

**(R)-(3-(3-((2-((tert-butoxycarbonyl)amino)-3-methoxy-3-oxopropyl)thio)propyl)-1-methyl-1H-imidazol-3-ium-2-yl)pentafluorophosphate (9).** To an oven-dried screw cap test tube containing a PTFE-coated stir bar was added (3-allyl-1-methyl-1H-imidazol-3-ium-2-yl)pentafluorophosphate **2g** (31 mg, 0.12 mmol, 1.00 equiv.) and AIBN (11 mg, 69 μmol, 0.55 equiv.). The tube was then sealed with a PTFE-lined septum screw cap and evacuated by inserting a needle through the septum cap to connect to the vacuum manifold. The tube was subjected to five cycles of successive evacuation and refilling with $N_2$. Methyl (*tert*-butoxycarbonyl)-L-cysteinate (0.11 mL, 0.53 mmol, 4.3 equiv.) and 1,2-dichloroethane (0.50 mL) were then added via syringe through the septum cap. The solution was sparged with $N_2$ for 15 minutes, with a DCE-filled pre-bubbler to saturate the $N_2$ stream with DCE and prevent solvent loss. Under counterflow of $N_2$, the septum cap was replaced with a solid PTFE-lined screw cap, and the tube was submerged in an oil bath set to 80 °C. After heating the reaction vessel for 18 hours, a second portion of AIBN (11 mg, 69 μmol, 0.55 equiv.) under counterflow of nitrogen. The vial was resealed and heating continued

for an additional 27 hours. The vial was cooled to room temperature and unsealed in a fume hood under ambient atmosphere. The title compound was obtained in 50 mg (82% yield) after purification by silica gel chromatography (hexanes/ethyl acetate = 100/0% to 0/100%).

$^{1}$H NMR (400 MHz, Acetone-$d_6$) δ 7.58 (t, *J* = 2.3 Hz, 1H), 7.49 (t, *J* = 2.5 Hz, 1H), 6.32 (d, *J* = 8.6 Hz, 1H), 4.48 (t, *J* = 7.8 Hz, 2H), 4.38 (td, *J* = 8.2, 5.2 Hz, 1H), 3.98 (s, 3H), 3.70 (s, 3H), 3.06 – 2.99 (m, 1H), 2.94 – 2.89 (m, 1H), 2.67 (t, *J* = 7.3 Hz, 2H), 2.20 – 2.09 (m, 2H), 1.40 (s, 9H).
$^{13}$C NMR (101 MHz, Acetone-$d_6$) δ 172.3, 156.2, 157.8 – 153.3 (m, C(2) not resolved above baseline), 124.4 (d, *J* = 10.0 Hz), 122.1 (d, *J* = 9.4 Hz), 120.2, 79.6, 55.4, 54.5, 52.5, 50.6 – 47.9 (m), 38.5 (t, *J* = 4.5 Hz), 34.2, 31.4, 29.6, 21.8, 15.0, 14.5.
$^{19}$F NMR (376 MHz, Acetone-$d_6$) δ -53.75 (dd, *J* = 777.6, 49.9 Hz), -73.85 (dp, *J* = 761.3, 49.9 Hz).
$^{31}$P NMR (162 MHz, Acetone-$d_6$) δ -150.54 (pd, *J* = 777.6, 761.4 Hz).
HRMS (ESI-TOFMS): $[M+Na]^+$ calculated for $[C_{16}H_{27}F_5N_3O_4PSNa]^+$ = 506.1272, found = 506.1273

## 5. Additional Optimization Data and Mechanistic Discussion

### a) **Table S1.** $LiPF_6$ equivalents screen

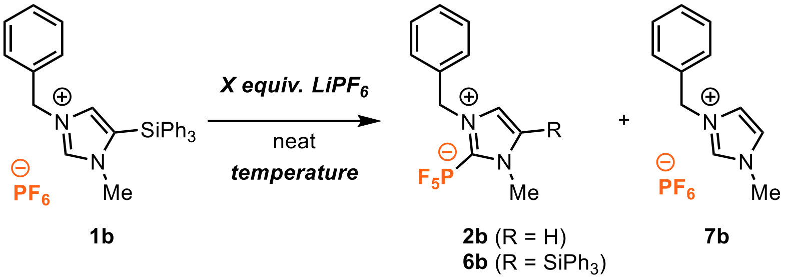


| entry [a] | equiv. $LiPF_6$ | temp. (°C) | % conv. | % yield 2b | % yield 6b | % yield 7b |
|---|---|---|---|---|---|---|
| **1** | 0 | 200 | 58 | 17 | 5 | 28 |
| **2** | 0.10 | 200 | 100 | 71 | 1 | 14 |
| **3** | 0.50 | 200 | 99 | 48 | 9 | 25 |
| **4** | 1.00 | 200 | 16 | 0 | 0 | 6 |
| **5** | 0 | 220 | 99 | 47 | 3 | 32 |
| **6** | 0.10 | 220 | 100 | 49 | 0 | 33 |
| **7** | 0.50 | 220 | 100 | 50 | 0 | 32 |
| **8** | 1.00 | 220 | 97 | 58 | 5 | 22 |

*[a] Reactions conducted according to the General Procedure described in Section 3a) at a 0.090 mmol scale. % Conversion and % yields were determined from $^{1}H$ NMR spectra by integration relative to dimethylsulfone, which was added to the crude reaction mixtures in a known amount after dissolving in acetone.*

### b) **Table S2.** Additive screen

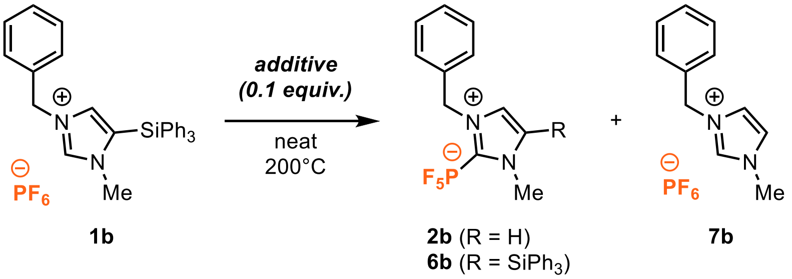


| entry [a] | additive (0.1 equiv.) | % conv. | % yield 2b | % yield 6b | % yield 7b |
|---|---|---|---|---|---|
| **1** | - | 58 | 17 | 5 | 28 |
| **2** | **$LiPF_6$** | **100** | **71** | **1** | **14** |
| **3** | LiOTf | 95 | 40 | 18 | 26 |
| **4** | **LiCl** | **100** | **73** | **2** | **10** |
| **5** | LiBr | 100 | 39 | 0 | 19 |
| **6** | $KPF_6$ | 80 | 16 | 13 | 37 |
| **7** | $TBAPF_6$ | 69 | 13 | 15 | 34 |
| **8** | $AgPF_6$ | 99 | 39 | 6 | 32 |
| **9** | $AlCl_3$ | 100 | 60 | 2 | 10 |
| **10** | **$ZnCl_2$** | **100** | **73** | **1** | **17** |
| **11** | $BCl_3$ | 97 | 44 | 11 | 17 |
| **12** | DBTDL | 100 | 9 | 0 | 59 |
| **13** | $BF_3•OEt_2$ | 100 | 3 | 0 | 0 [b] |

*[a] Reactions conducted according to the General Procedure described in Section 3a) at a 0.090 mmol scale. % Conversion and % yields were determined from $^{1}H$ NMR spectra by integration relative to dimethylsulfone, which was added to the crude reaction mixtures in a known amount after dissolving in acetone. [b] An imidazolium tetrafluoroborate salt was obtained as a major byproduct.*

## c) **Table S3.** Solvent screen

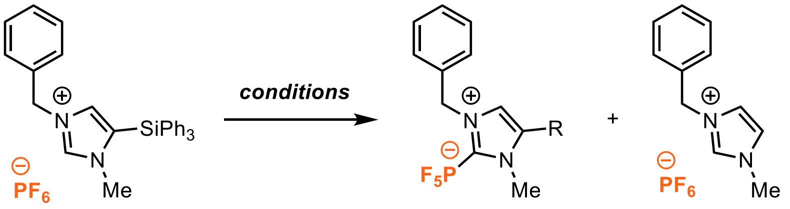

**1b** | **2b** (R = H), **6b** (R = $SiPh_3$) | **7b**

| entry[a] | solvent[b] | additive[c] | temp. (°C) | % conv. | % yield 2b | % yield 6b | % yield 7b |
|---|---|---|---|---|---|---|---|
| **1** | **$Ph_2O$** | **-** | **220** | **100** | **79** | **0** | **12** |
| **2** | **$Ph_2O$** | **$LiPF_6$** | **220** | **100** | **74** | **0** | **21** |
| **3** | Diglyme | - | 220 | 100 | 40 | 0 | 35 |
| **4** | Diglyme | $LiPF_6$ | 220 | 100 | 37 | 0 | 37 |
| **5** | PhOMe | - | 220 | 100 | 68 | 0 | 15 |
| **6** | PhOMe | $LiPF_6$ | 220 | 100 | 47 | 0 | 27 |
| **7** | 1,2-DCB[d] | - | 220 | 98 | 67 | 7 | 13 |
| **8** | 1,2-DCB[d] | $LiPF_6$ | 220 | 100 | 47 | 0 | 26 |
| **9** | $Ph_2O$ | - | 200 | 100 | 74 | 1 | 14 |
| **10** | **$Ph_2O$** | **$LiPF_6$** | **200** | **100** | **80** | **0** | **12** |
| **11** | Diglyme | - | 200 | 100 | 53 | 0 | 35 |
| **12** | Diglyme | $LiPF_6$ | 200 | 100 | 43 | 0 | 38 |
| **13** | PhOMe | - | 200 | 98 | 57 | 11 | 21 |
| **14** | PhOMe | $LiPF_6$ | 200 | 100 | 70 | 0 | 12 |
| **15** | 1,2-DCB[d] | - | 200 | 100 | 79 | 3 | 11 |
| **16** | 1,2-DCB[d] | $LiPF_6$ | 200 | 100 | 70 | 0 | 14 |
| **17** | $Ph_2O$ | - | 180 | 35 | 1 | 7 | 20 |
| **18** | $Ph_2O$ | $LiPF_6$ | 180 | 98 | 45 | 17 | 29 |
| **19** | 1,2-DCB[d] | - | 180 | 24 | 0 | 8 | 15 |
| **20** | 1,2-DCB[d] | $LiPF_6$ | 180 | 99 | 38 | 21 | 30 |
| **21** | $Ph_2O$ | - | 160 | 16 | 0 | 2 | 9 |
| **22** | $Ph_2O$ | $LiPF_6$ | 160 | 46 | 5 | 17 | 22 |
| **23** | 1,2-DCB[d] | - | 160 | 14 | 0 | 2 | 6 |
| **24** | 1,2-DCB[d] | $LiPF_6$ | 160 | 84 | 12 | 32 | 37 |

*[a] Reactions conducted according to the General Procedure described in Section 3a) at a 0.090 mmol scale. % Conversion and % yields were determined from $^1$H NMR spectra by integration relative to dimethylsulfone, which was added to the crude reaction mixtures in a known amount after dissolving in acetone. [b] Reactions performed at a substrate concentration of 1.0 M.*
*[c] 0.10 equiv. additive relative to 1b. [d] 1,2-DCB = 1,2-dichlorobenzene.*

## d) **Table S4.** Evaluation of air and moisture tolerance

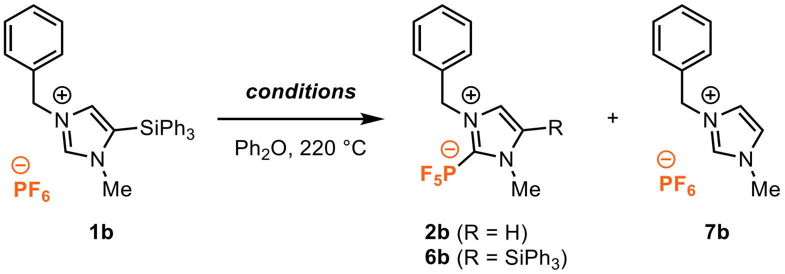


| entry [a] | Modifications to General Procedure[a] | % conv. | % yield 2b | % yield 3b | % yield 4b |
|---|---|---|---|---|---|
| 1 | no change | 100 | 79 | 0 | 12 |
| 2 | evacuation/purge at r.t. instead of 120 °C | 100 | 60 | 0 | 22 |
| 3 | no evacuation/purge | 100 | 6 | 0 | 66 |

*[a] Reactions conducted according to the General Procedure described in Section 3a) at a 0.090 mmol scale. % Conversion and % yields were determined from $^1$H NMR spectra by integration relative to dimethylsulfone, which was added to the crude reaction mixtures in a known amount after dissolving in acetone.*

## e) Additional Control Experiments

Control reactions using imidazolium hexafluorophosphate salt **7b** as a substrate showed no evidence of NHC-$PF_5$ adduct formation (Figure S5), even in the presence of an exogenous arylsilane (i.e. $PhSiMe_3$; see Figure S6), confirming that silylimidazolium hexafluorophosphate salts are uniquely required for productive reactivity at these temperatures. Experiments probing whether adduct **6** can undergo protodesilylation under the reaction conditions showed only decomposition (Figure S7), implying that **6** is not a source of **2** and is, instead, a mechanistic dead-end. Since the formation of **6** is unavoidable according to the proposed mechanism by which imidazol-5-ylidene **5** is converted to imidazole-2-ylidene **4**, this observation is consistent with the plateau in yield observed for **2b** at ca. 80%.

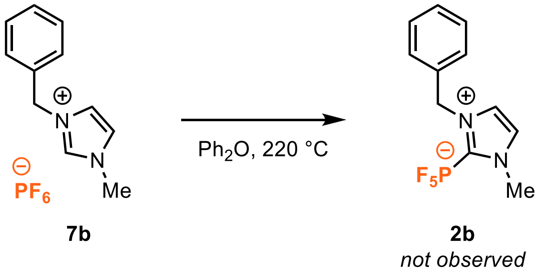


**Figure S5.** Control experiment with imidazolium hexafluorophosphate salt **7b**. Product **2b** was not observed in $^1$H and $^{19}$F NMR spectra of the crude reaction mixture.

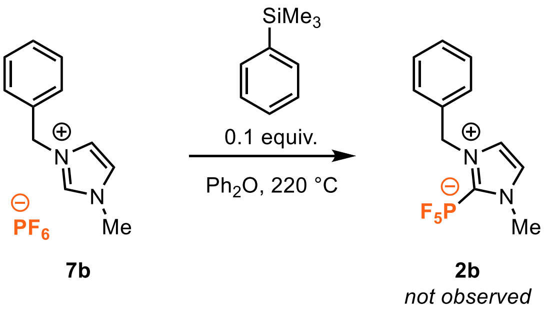


**Figure S6.** Control experiment with imidazolium hexafluorophosphate salt **7b** and phenyltrimethylsilane as an additive. Product **2b** was not observed in $^1$H and $^{19}$F NMR spectra of the crude reaction mixture, demonstrating that an exogenous silane additive is not effective in promoting NHC-$PF_5$ formation.

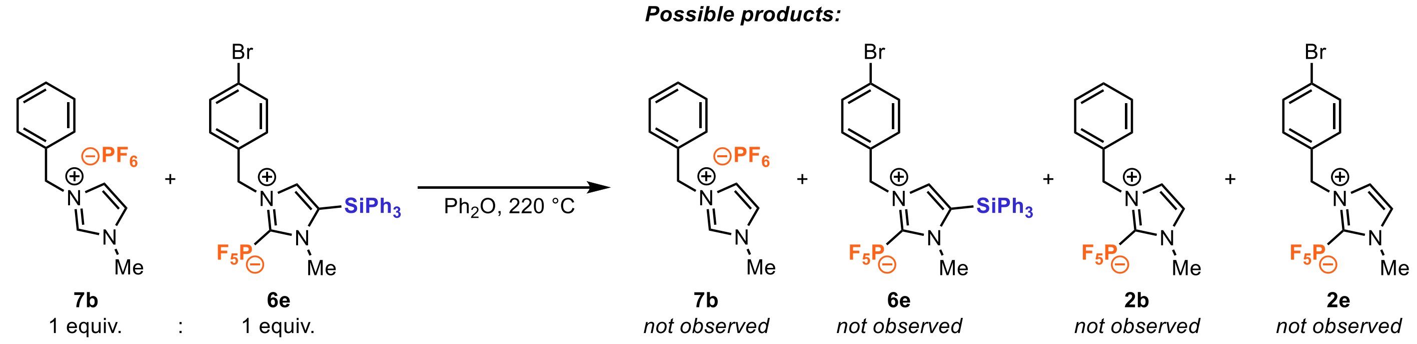


**Figure S7.** Control experiment testing whether a silylated NHC-$PF_5$ (**6e)** can undergo protodesilylation to the corresponding NHC-$PF_5$ **2b** in the presence of an imidazolium hexafluorophosphate salt (**7b**). $^{19}$F NMR spectra of the crude reaction mixture showed no signals for either $PF_6^-$ or $PF_5$. Note: a pure sample of **6e** was obtained by column chromatography from a 1 mmol scale reaction of **1e** which showed higher ratios of **6e:2e** than typical reactions. We did not obtain full characterization data on **6e** due to limited quantities.

## f) Additional mechanistic discussion

We have considered 3 plausible mechanisms for the formation of imidazol-5-ylidene **5** from silylimidazolium **1** (Figure S8): **a)** Stepwise dissociation of $PF_6^-$ to $PF_5$ and $F^-$, followed by $F^-$-induced desilylation of the 5-silylimidazolium cation. **b)** Concomitant defluorination of $PF_6^-$ to $PF_5$ and desilylation of the 5-silylimidazolium cation., and **c)** Defluorination of $PF_6^-$ to $PF_5$ via Lewis acid (LA) abstraction of a $F^-$, followed by nucleophilic desilylation of the 5-silylimidazolium cation by the LA-$F^-$ complex. Our optimization data with $LiPF_6$ and other Lewis acidic additives is consistent with LA-assisted defluorination of $PF_6^-$ (i.e., Mechanism C in Figure S8). The data in Table S3 shows that $LiPF_6$ is crucial to promoting the conversion of starting material at lower temperatures by LA-assisted defluorination of $PF_6^-$, but this beneficial effect is superfluous at elevated temperatures when background decomposition of $PF_6^-$ is high. While catalytic $LiPF_6$ affords reasonable yields of **2b** with or without a solvent, all reactions performed at 220 °C showed lower yields and poorer mass balance when a solvent was not used (Table 1 of main text, entries 9 & 10 vs. entries 11 & 12).

In addition to promoting $PF_6^-$ decomposition, it is possible that $Li^+$ and other Lewis acidic additives attenuate unproductive decomposition by reversible adduction with the putative imidazolylidene intermediates (Figure S9). Similar processes are known for $Li^+$ adducts of imidazolyl NHCs.[5]

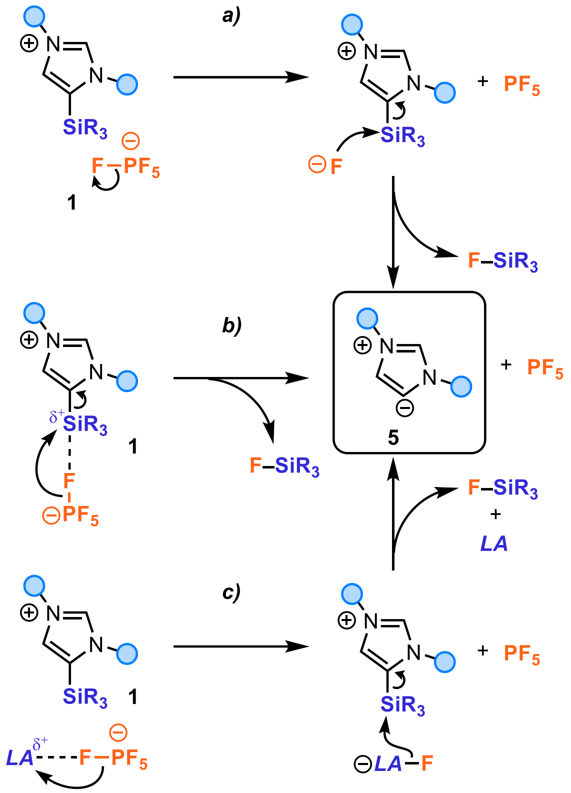


**Figure S8.** Possible mechanisms for the defluorinative decomposition of $PF_6^-$ to $PF_5$ and the formation of imidazol-5-ylidene **5**: **a)** Stepwise dissociation of $PF_6^-$ to $PF_5$ and $F^-$, followed by nucleophilic desilylation of the 5-silylimidazolium cation. **b)** Concomitant defluorination of $PF_6^-$ to $PF_5$ and desilylation of the 5-silylimidazolium cation. In this mechanism, the electrophilic silane acts as a Lewis acid and abstracts a fluoride from $PF_6^-$. **c)** Defluorination of $PF_6^-$ to $PF_5$ via Lewis acid (LA) abstraction of a $F^-$, followed by nucleophilic desilylation of the 5-silylimidazolium cation by the LA-$F^-$ complex.

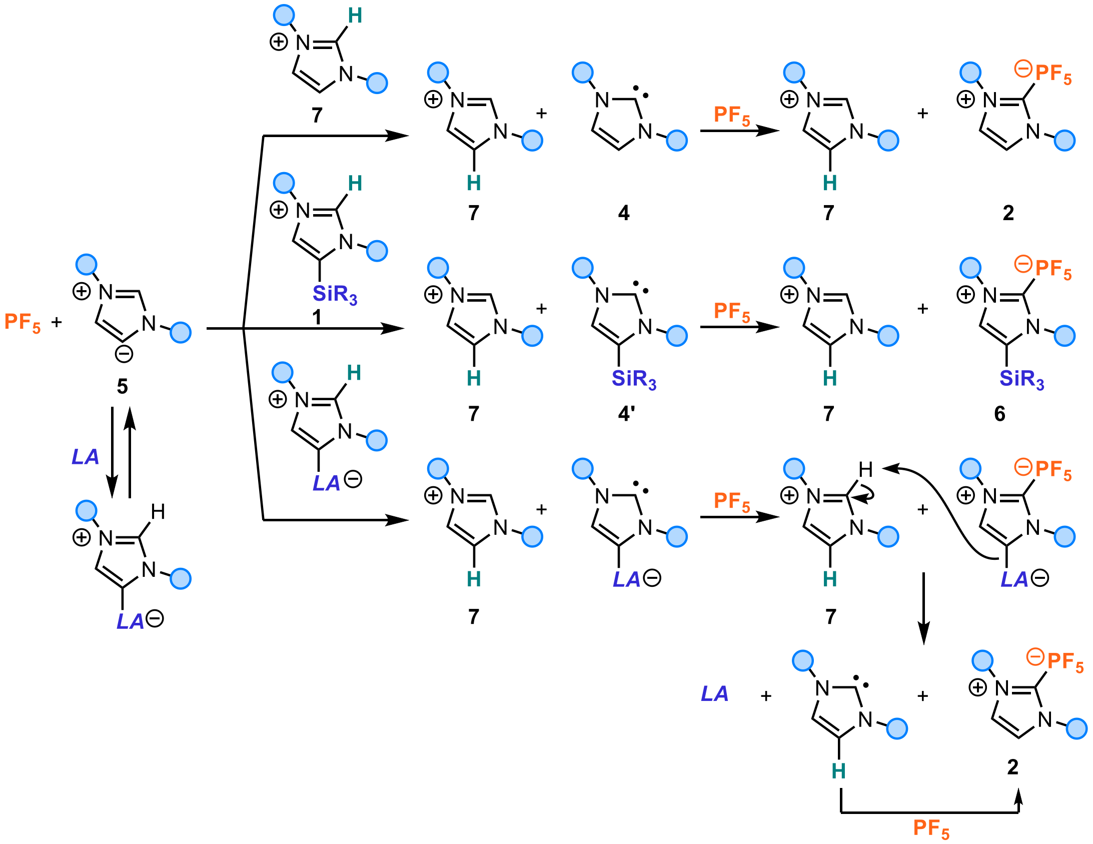


**Figure S9.** Beginning with imidazol-5-ylidene **5** (formed via the mechanisms shown in **Figure S8**), possible mechanistic pathways involved in the formation of NHC-$PF_5$ adducts **2** and **6** and imidazolium salt **7.**

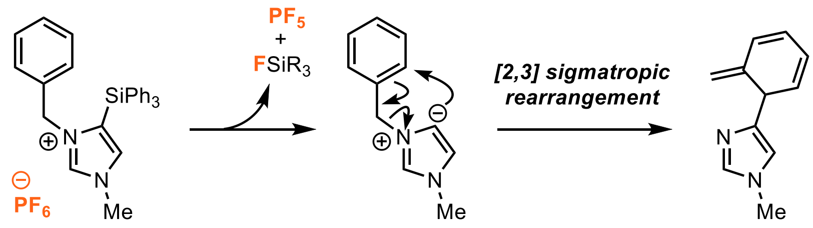


**1b'**

**Figure S10.** Possible decomposition pathway for silylimidazolium **1b'.**

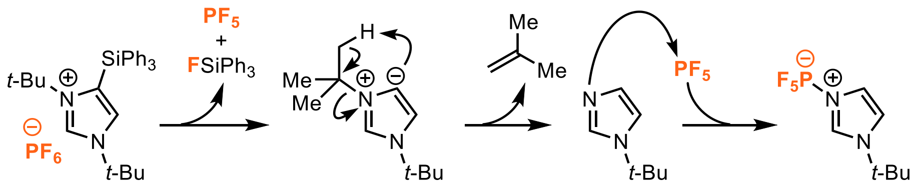


**1m** **2m'**

**Figure S11.** Possible mechanism for the formation of *N*-$PF_5$ adduct **2m'**

## 6. X-ray Crystallography

### a) Crystallization of compounds 2a, 6a, and 13

Crystalline samples of compounds **2a** and **6a** were isolated from the reaction of silylimidazolium **1a** following the General Procedure (Section 3b). The crude reaction mixture was subjected to column chromatography with a gradient eluent from 100% hexanes to 100% EtOAc, whereupon compounds **2a** and **6a** eluted as an inseparable mixture in a molar ratio of 3.0:1.0 (**2a:6a**) as determined by $^1$H NMR spectroscopy. This mixture was dissolved in $CHCl_3$/MeOH (1:1 v/v), and needle-like crystals formed upon slow evaporation of the solvent. Single crystals of differing morphologies were picked for XRD analysis, and the corresponding structures **2a** and **6a** were determined as described below.

Crystals of compound **13** suitable for XRD analysis were grown by slow diffusion of $Et_2O$ into a solution of **13** in MeCN.

### b) X-ray Crystallography Experimental Details for compounds 2a, 6a, and 13

Single crystal X-ray diffraction data for **2a**, **6a**, and **13** were collected at 100 K on diffractometers equipped with Mo rotating anodes and either a Bruker ApexII-Ultra CCD or a Bruker D8-Venture Photon3 detector. The data were integrated using SAINT and post-integration scaling and absorption corrections were carried out with SADABS.[6] Each structure was solved with SHELXT[7] using direct methods and the anisotropic displacement parameters of these solutions were subsequently refined against $F^2$ using SHELXL[8] and Olex2.[9] The positions of hydrogen atoms were set using a riding model. These crystallographic data are available from the Cambridge Crystallographic Data Center (CCDC) under their respective deposition IDs.

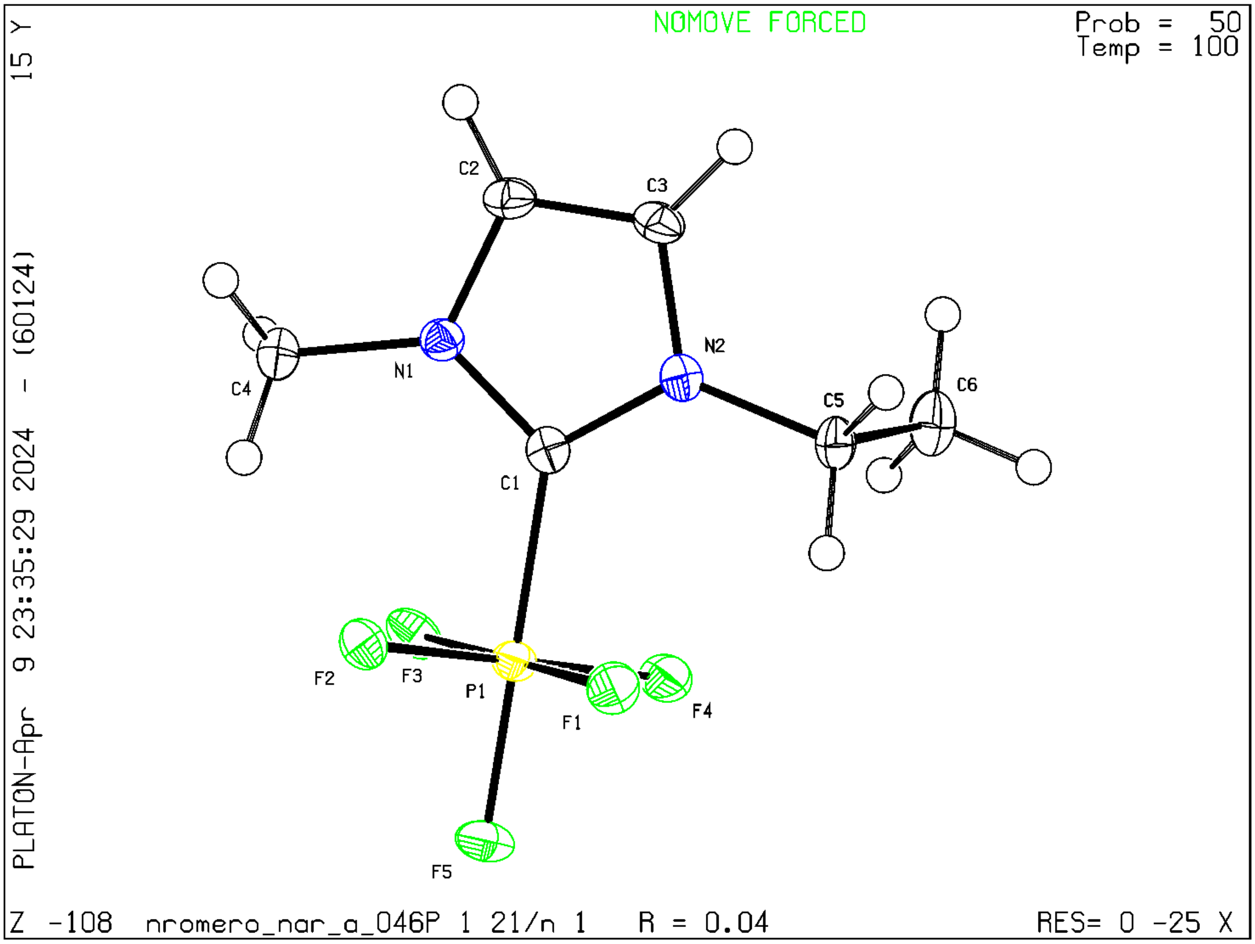


**Figure S12.** ORTEP of **2a** with C (white), N (blue), F (green), and P (yellow) represented at 50% probability levels and H (white) depicted as spheres. CCDC deposition ID: 2347336.

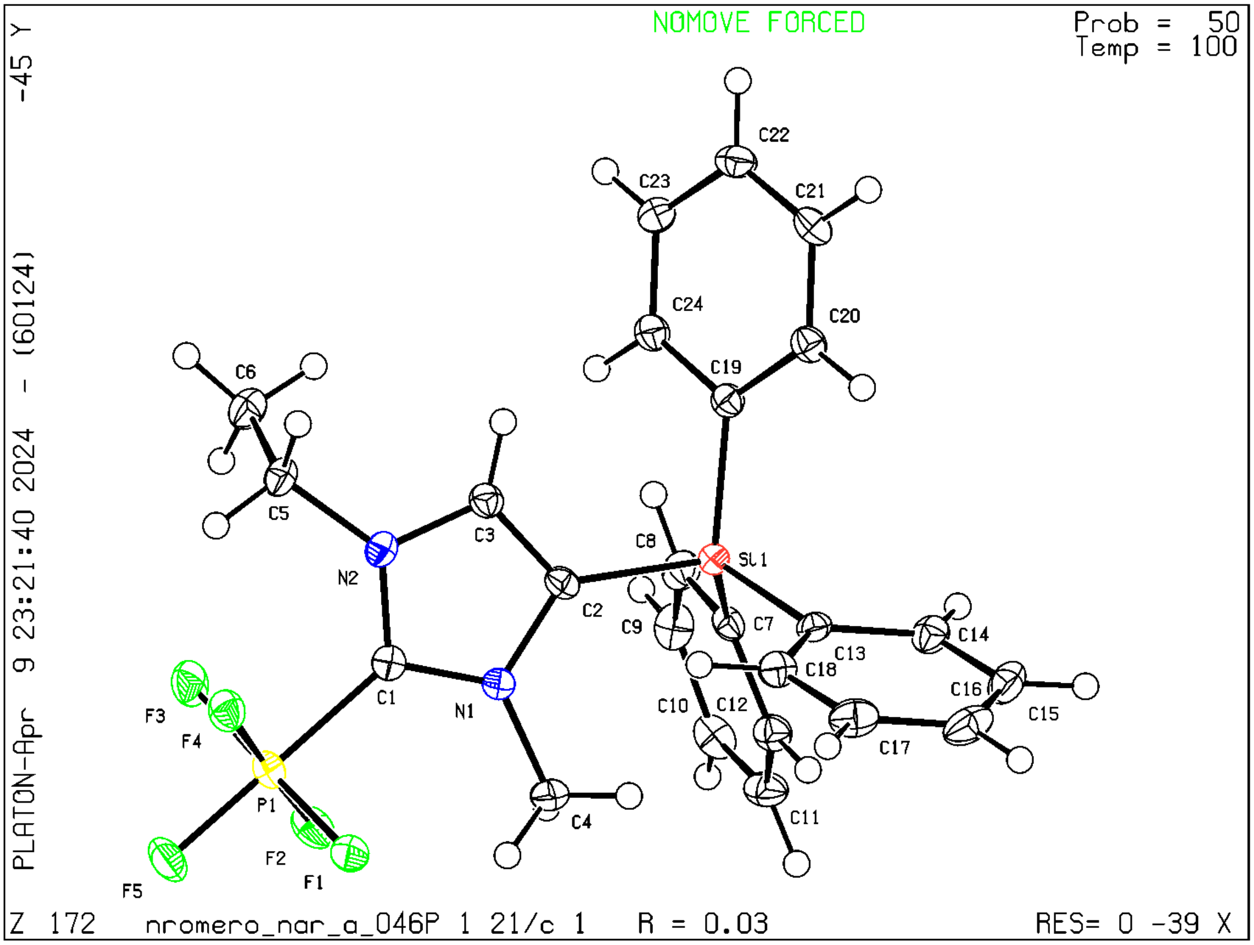


**Figure S13.** ORTEP of **6a** with C (white), N (blue), F (green), Si (red), and P (yellow) represented at 50% probability levels and H (white) depicted as spheres. CCDC deposition ID: 2347334.

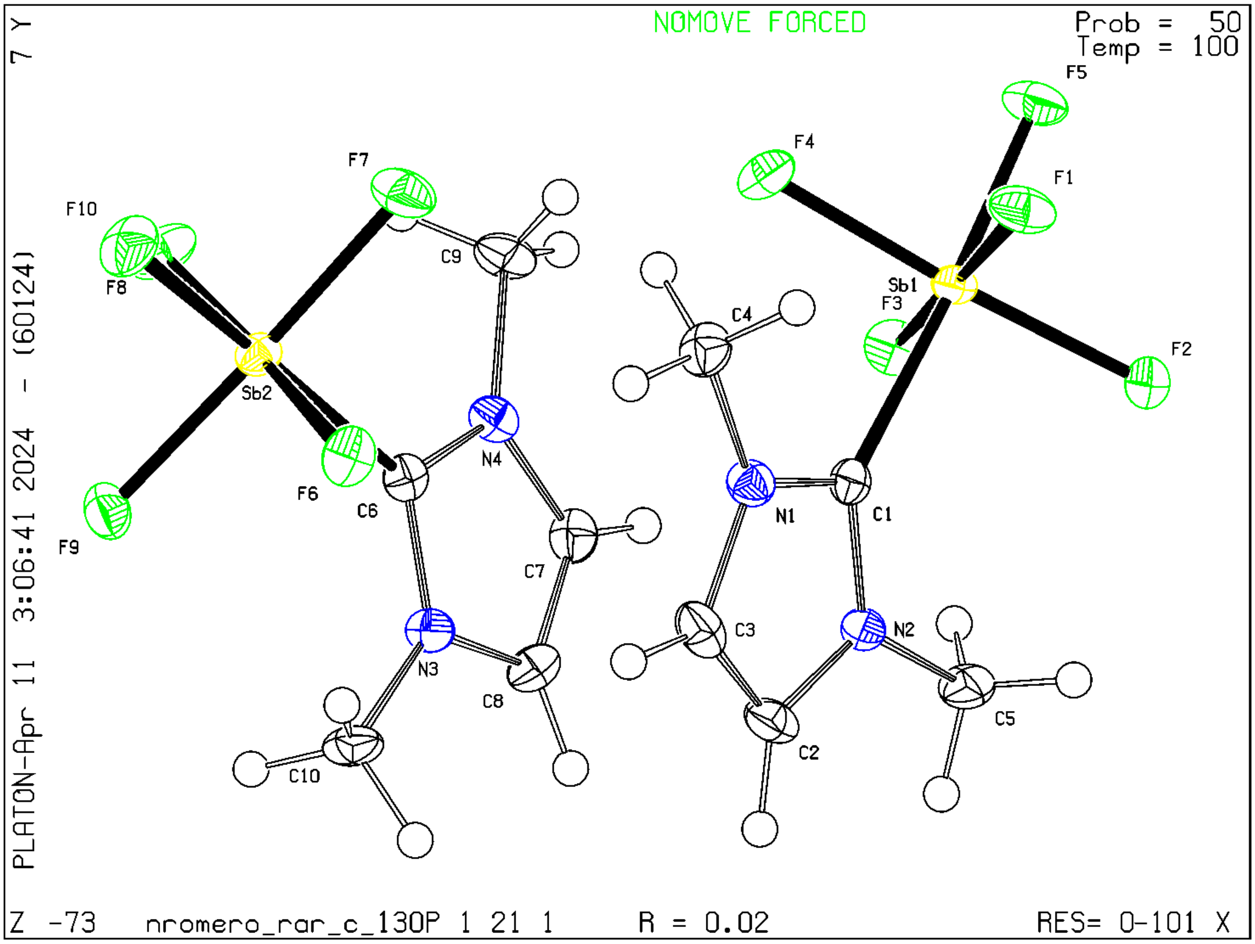


**Figure S14.** ORTEP of **13** with C (white), N (blue), F (green), and Sb (yellow) represented at 50% probability levels and H (white) depicted as spheres. Two molecules of **13** were observed per unit cell. CCDC deposition ID: 2348030.

# 7. NMR Spectra

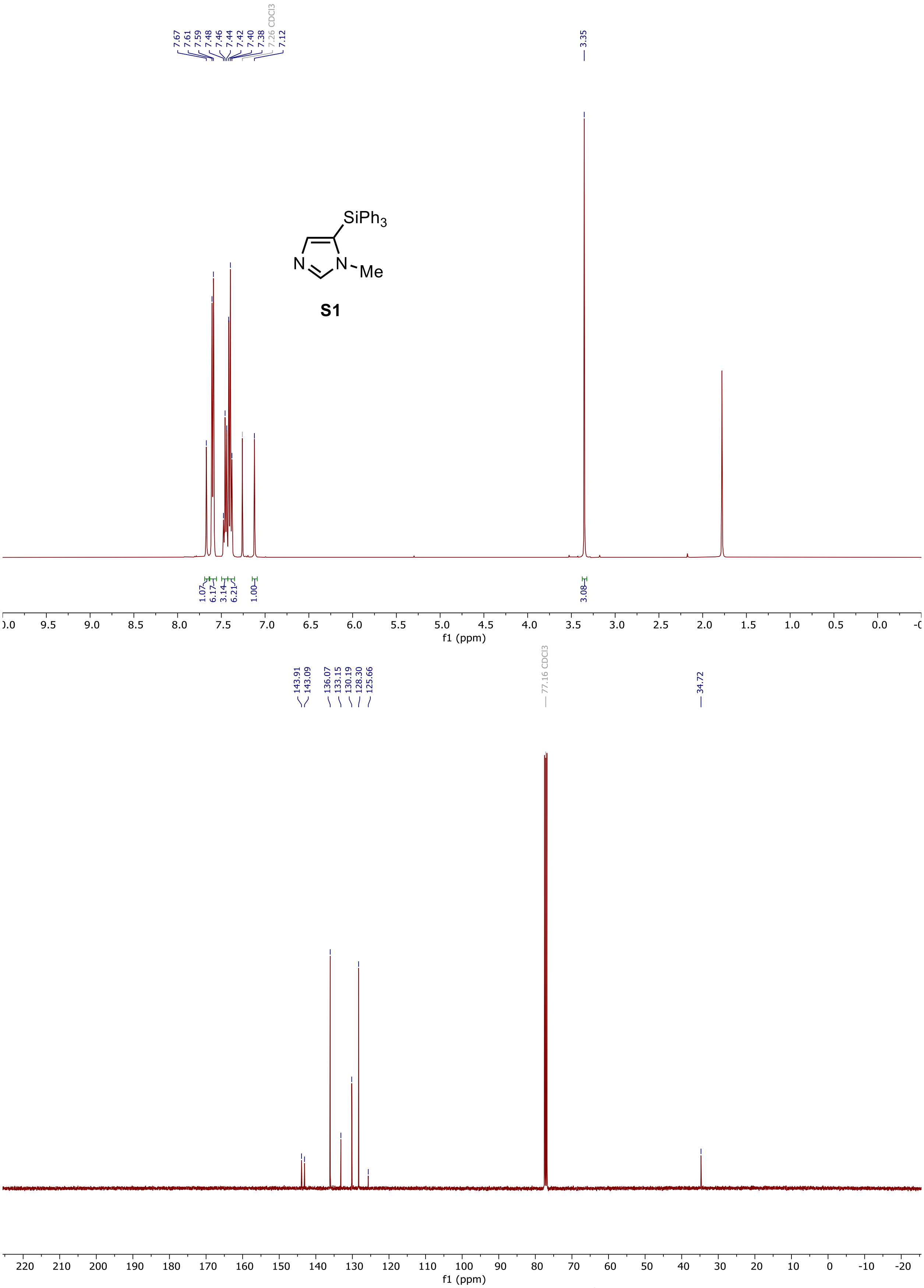


**Figure S15.** Top: $^{1}H$ NMR spectrum (400 MHz), and bottom: $^{13}C$ NMR spectrum (101 MHz) of **S1** in Chloroform-*d*.

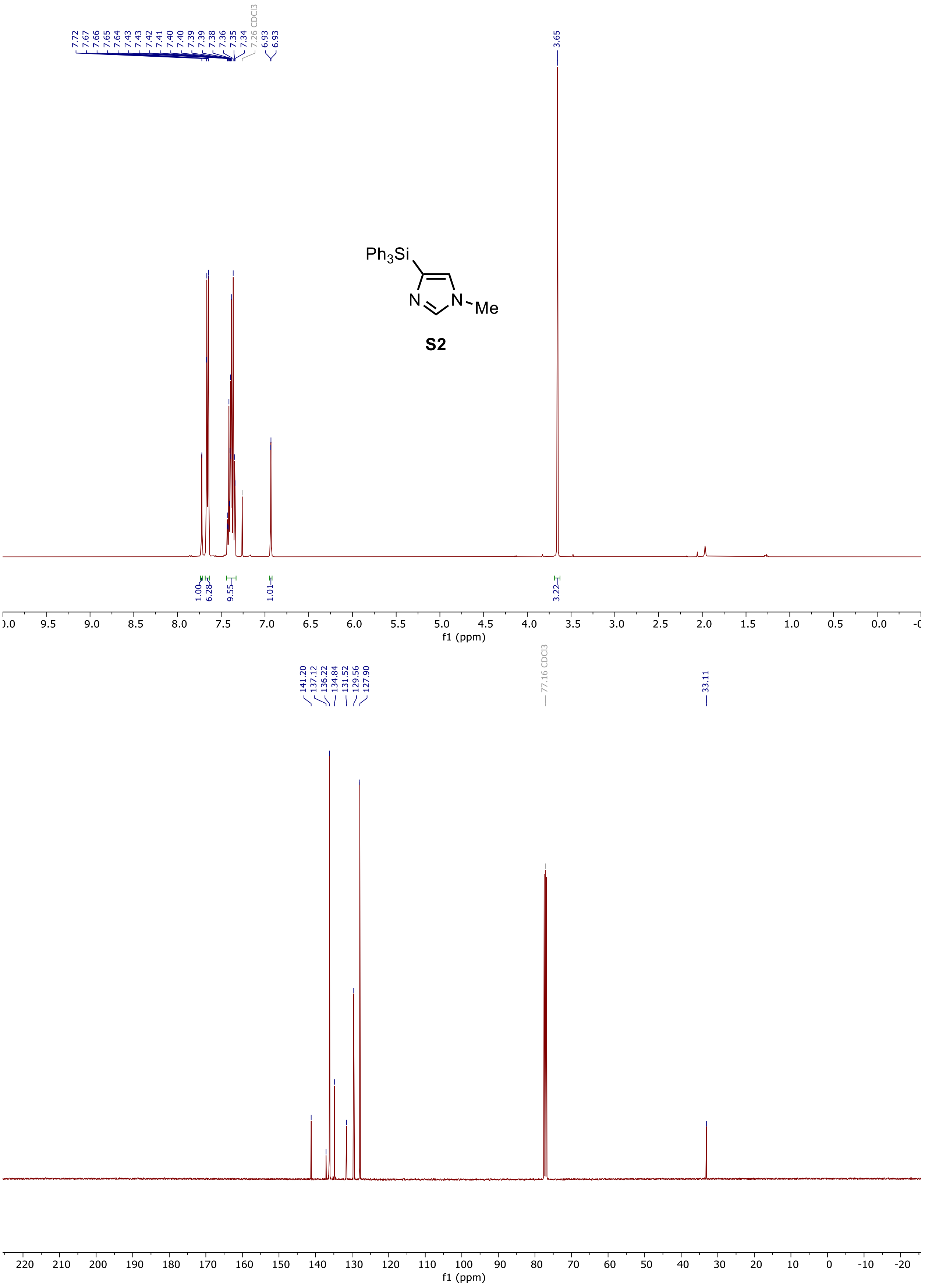


**Figure S16.** Top: $^{1}H$ NMR spectrum (400 MHz), and bottom: $^{13}C$ NMR spectrum (101 MHz) of **S2** in Chloroform-*d*.

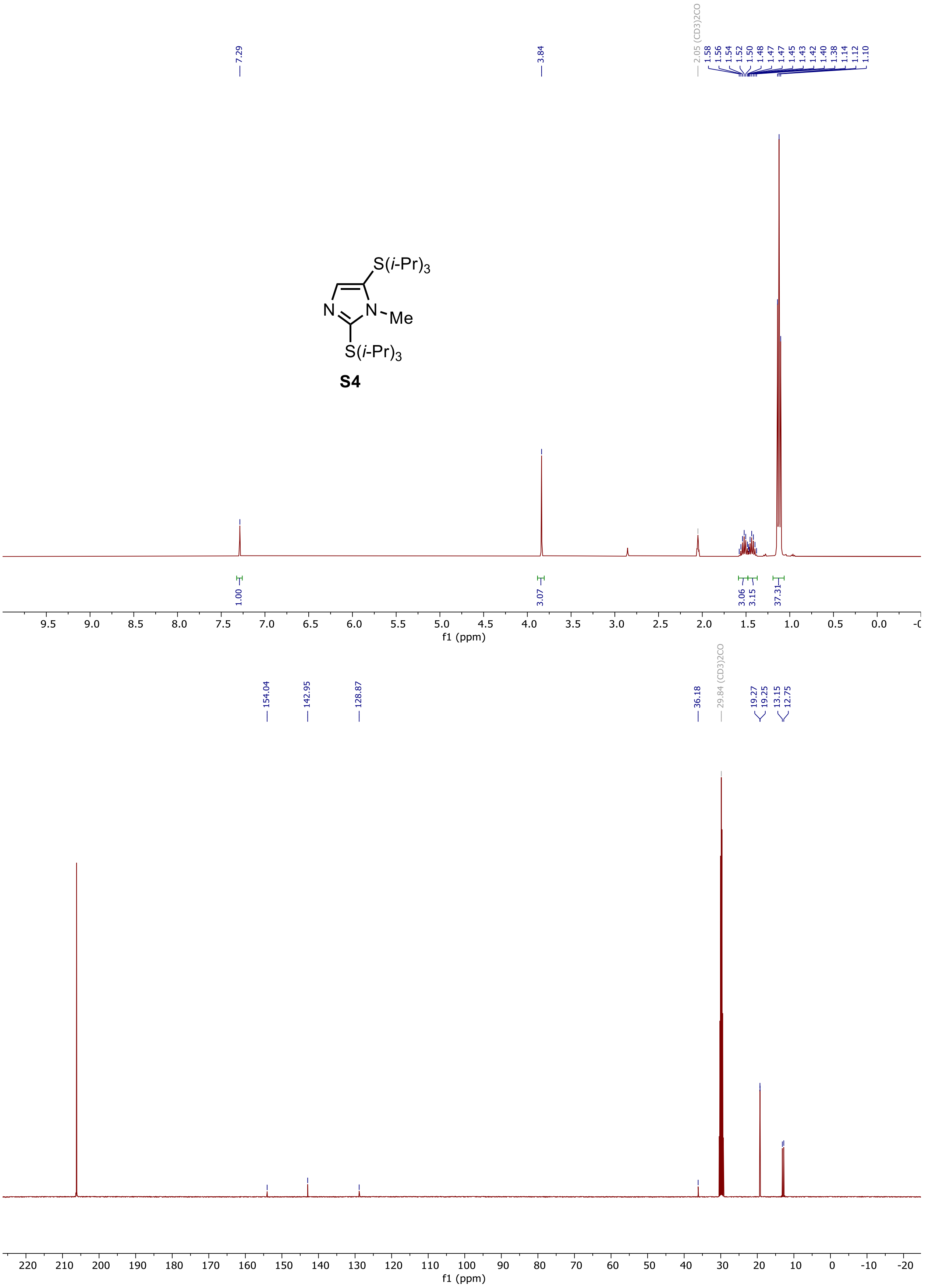


**Figure S17.** Top: $^{1}H$ NMR spectrum (400 MHz), and bottom: $^{13}C$ NMR spectrum (101 MHz) of **S4** in Acetone-$d_6$.

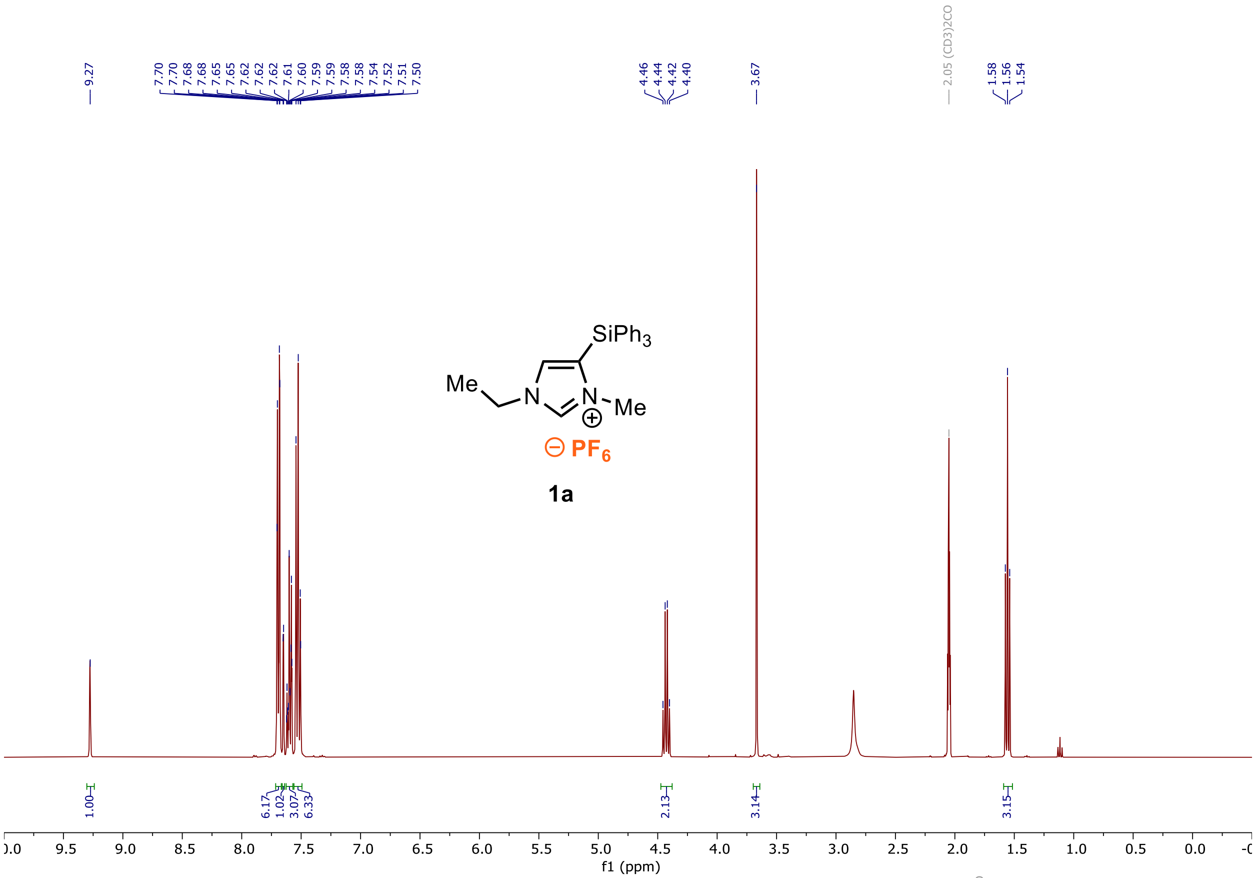


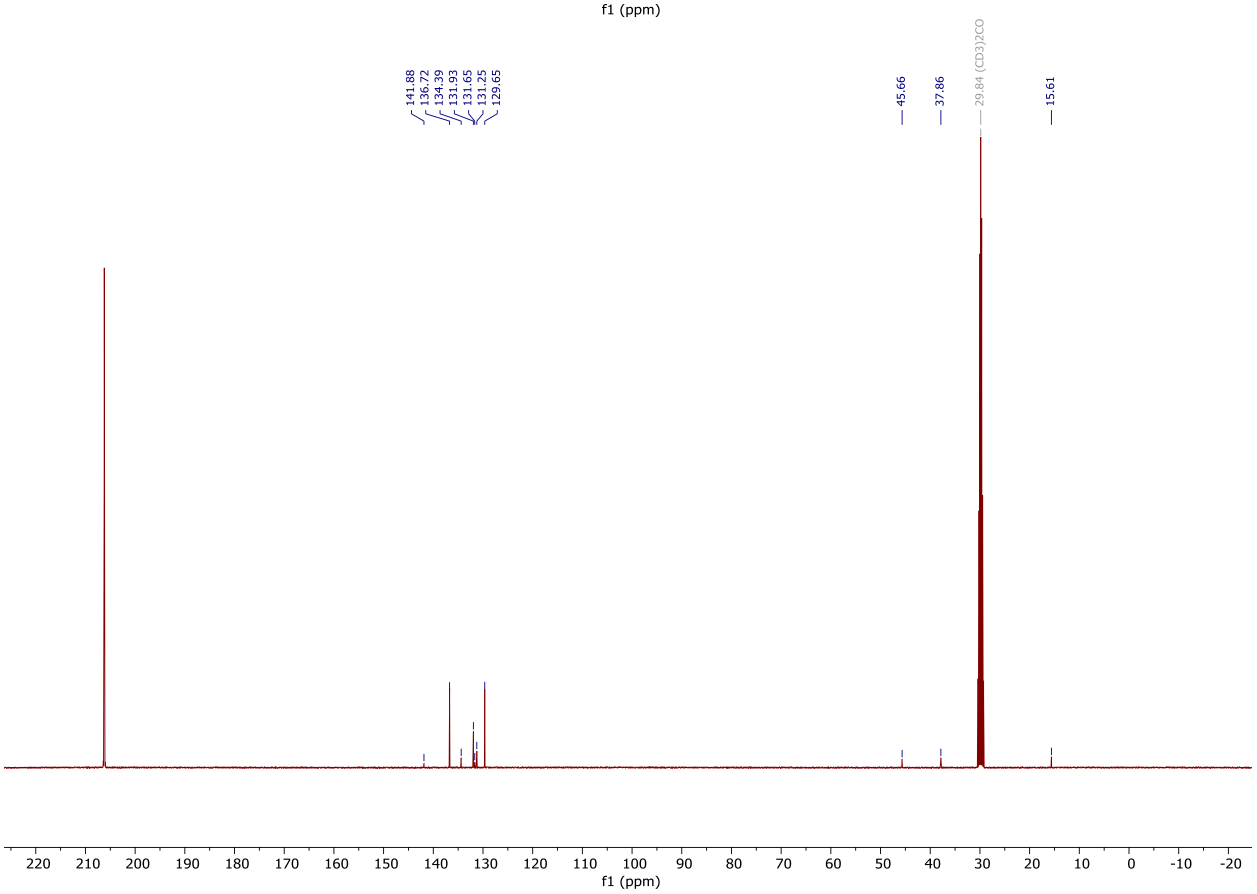


**Figure S18**. Top: $^{1}H$ NMR spectrum (400 MHz), and bottom: $^{13}C$ NMR spectrum (101 MHz) of **1a** in Acetone-$d_6$.

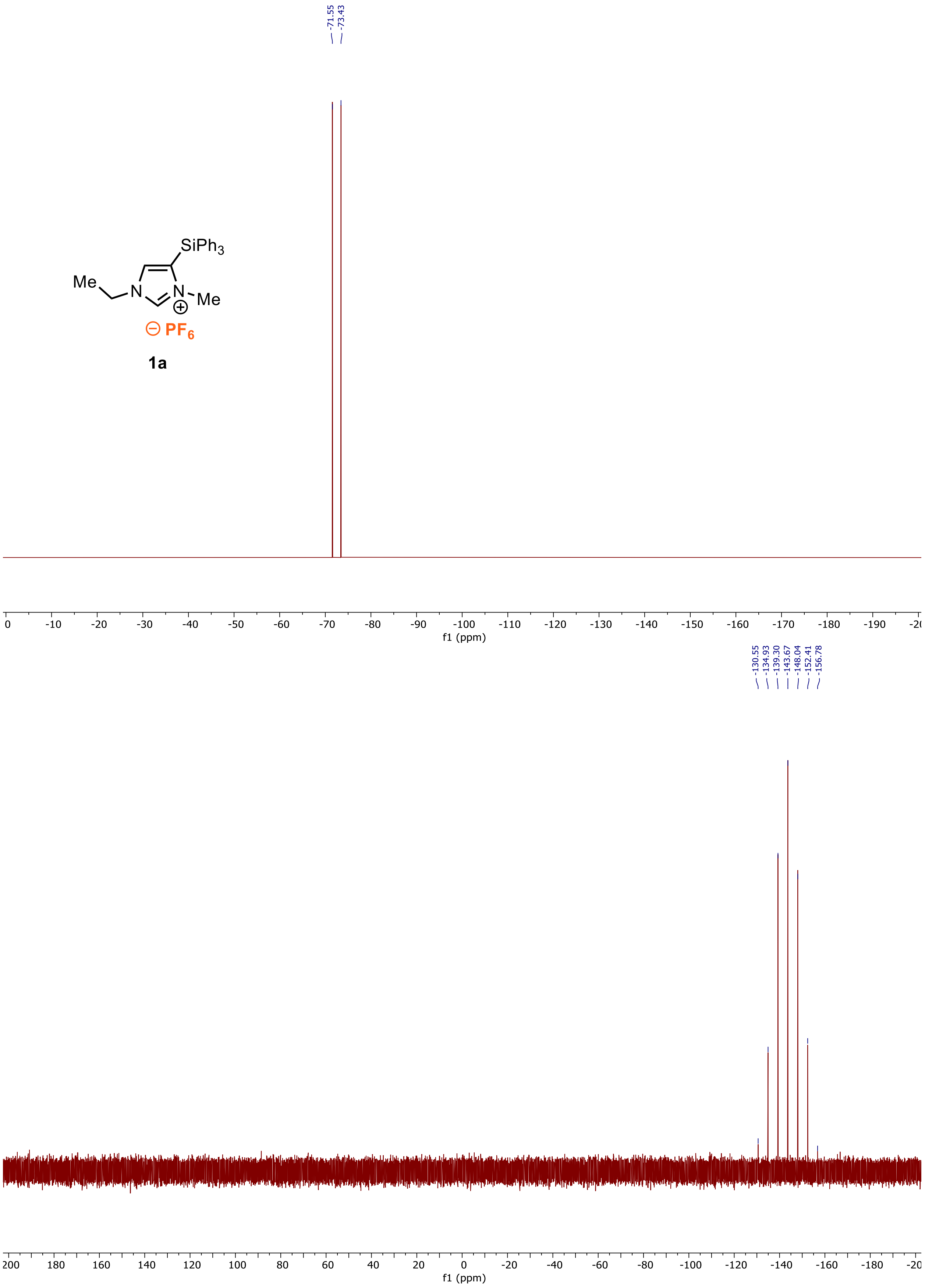


**Figure S19**. Top: $^{19}$F NMR spectrum (376 MHz), and bottom: $^{31}$P NMR spectrum (162 MHz) of **1a** in Acetone-$d_6$.

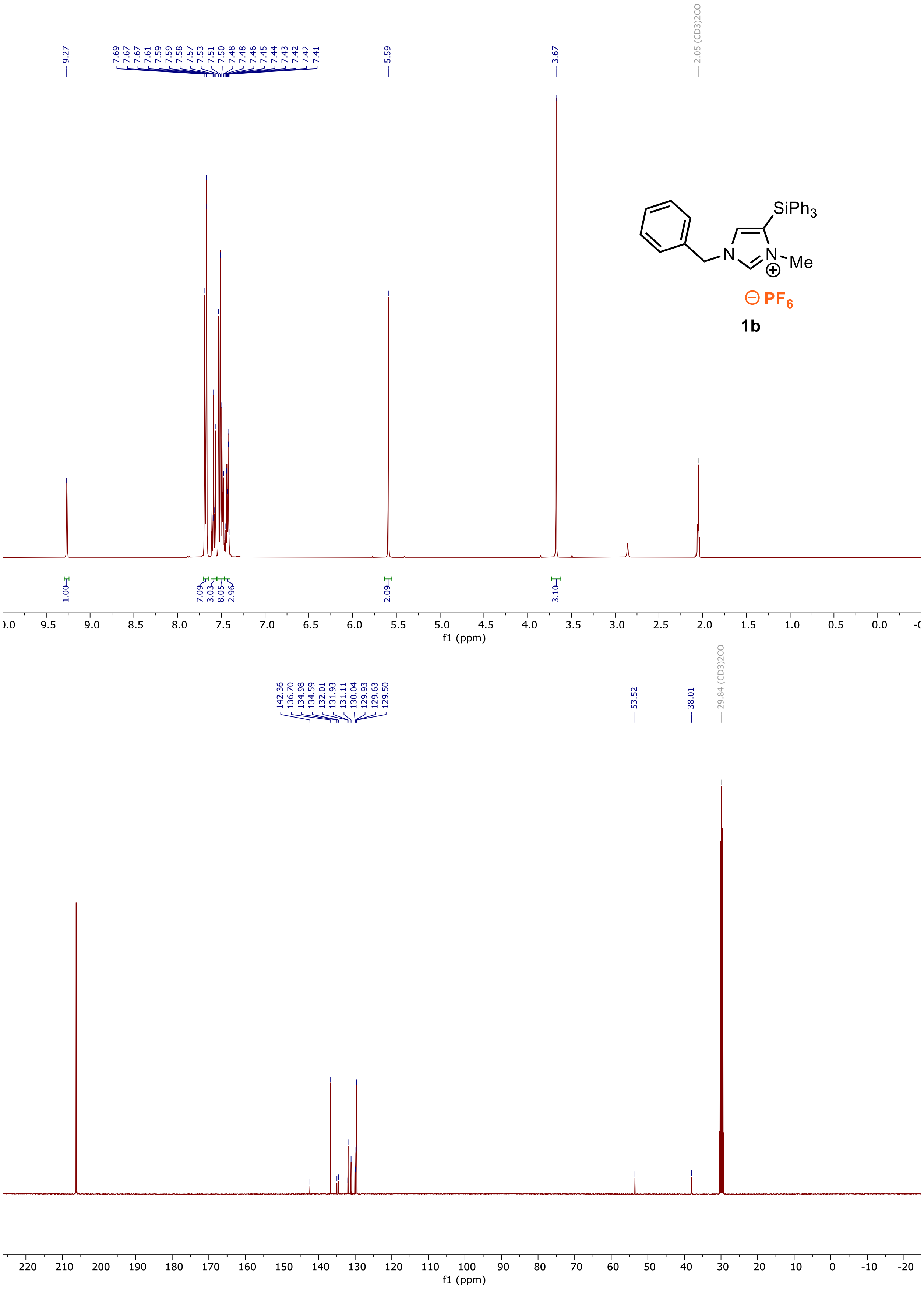


**Figure S20**. Top: $^{1}H$ NMR spectrum (400 MHz), and bottom: $^{13}C$ NMR spectrum (101 MHz) of **1b** in Acetone-$d_6$.

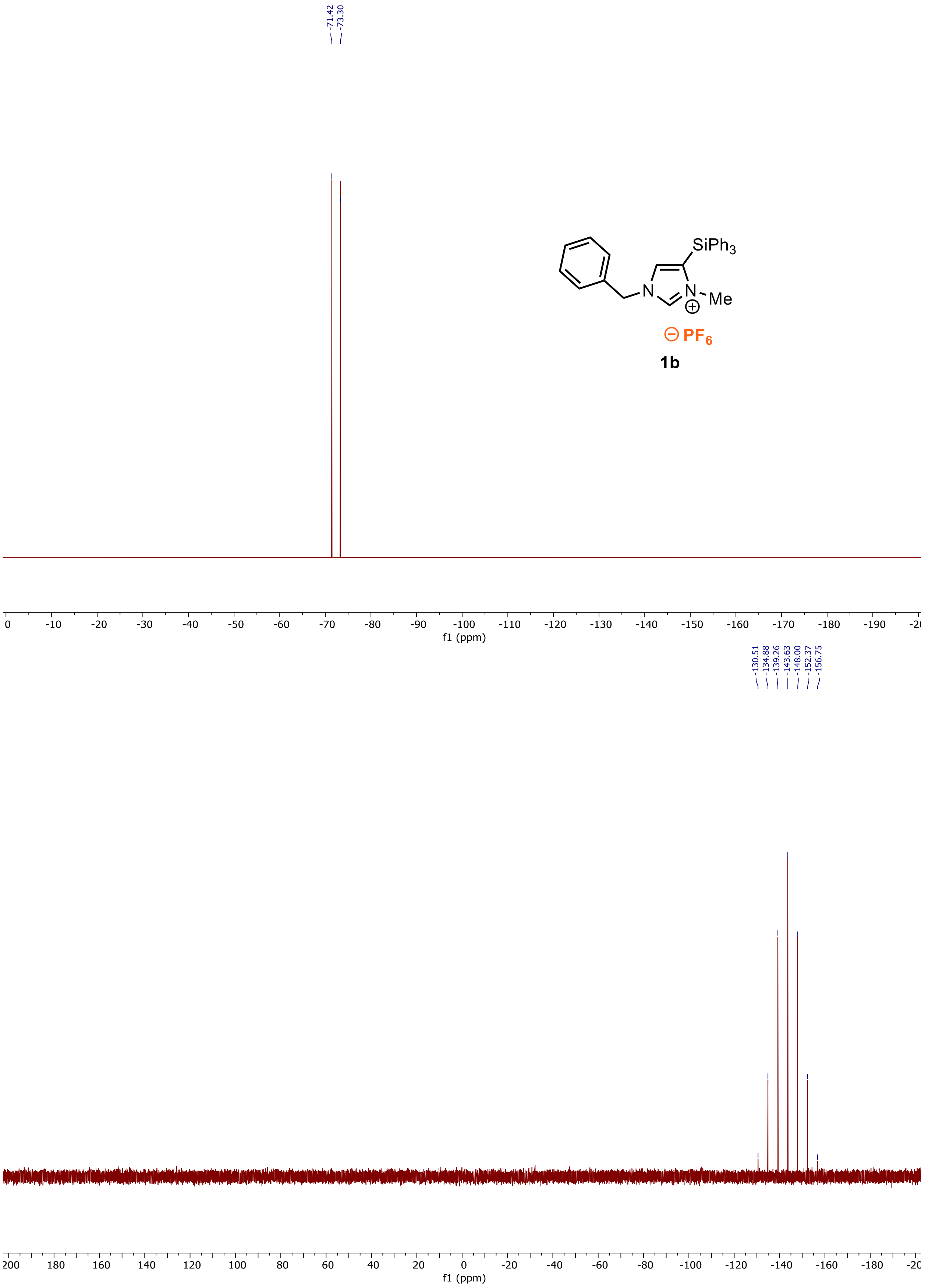


**Figure S21**. Top: $^{19}F$ NMR spectrum (376 MHz), and bottom: $^{31}P$ NMR spectrum (162 MHz) of **1b** in Acetone-$d_6$.

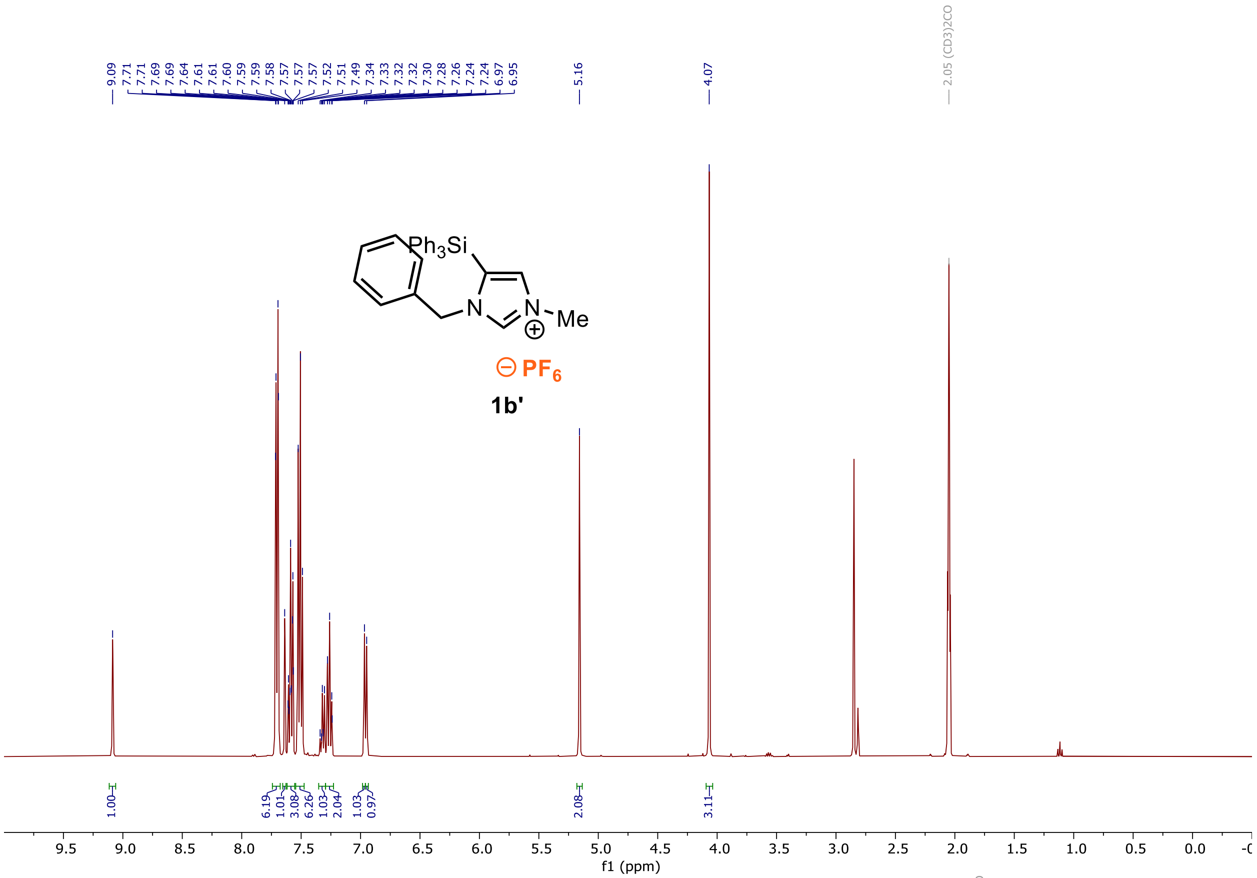


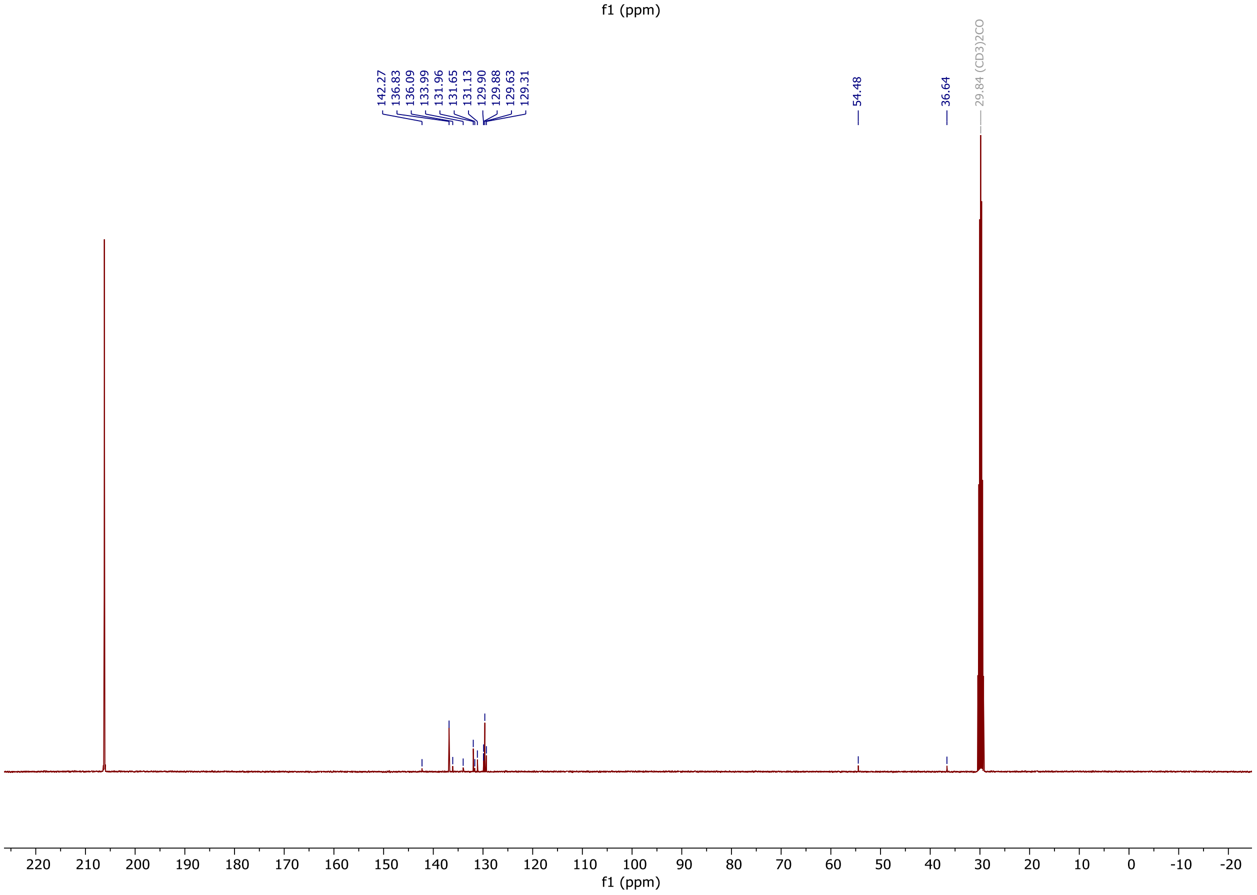


**Figure S22**. Top: $^{1}$H NMR spectrum (400 MHz), and bottom: $^{13}$C NMR spectrum (101 MHz) of **1b’** in Acetone-*d*6.

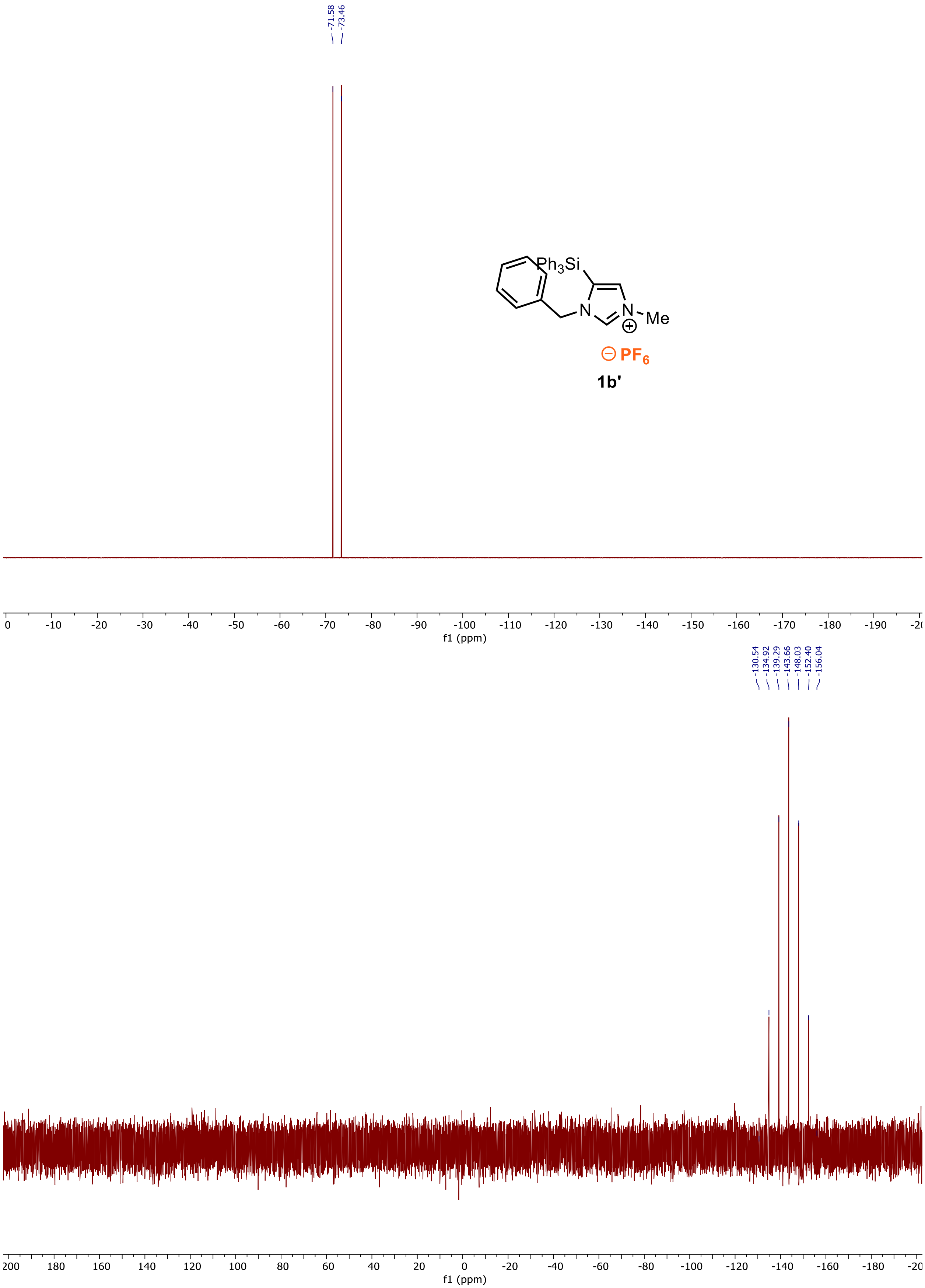


**Figure S23**. Top: $^{19}$F NMR spectrum (376 MHz), and bottom: $^{31}$P NMR spectrum (162 MHz) of **1b’** in Acetone-*d*6.

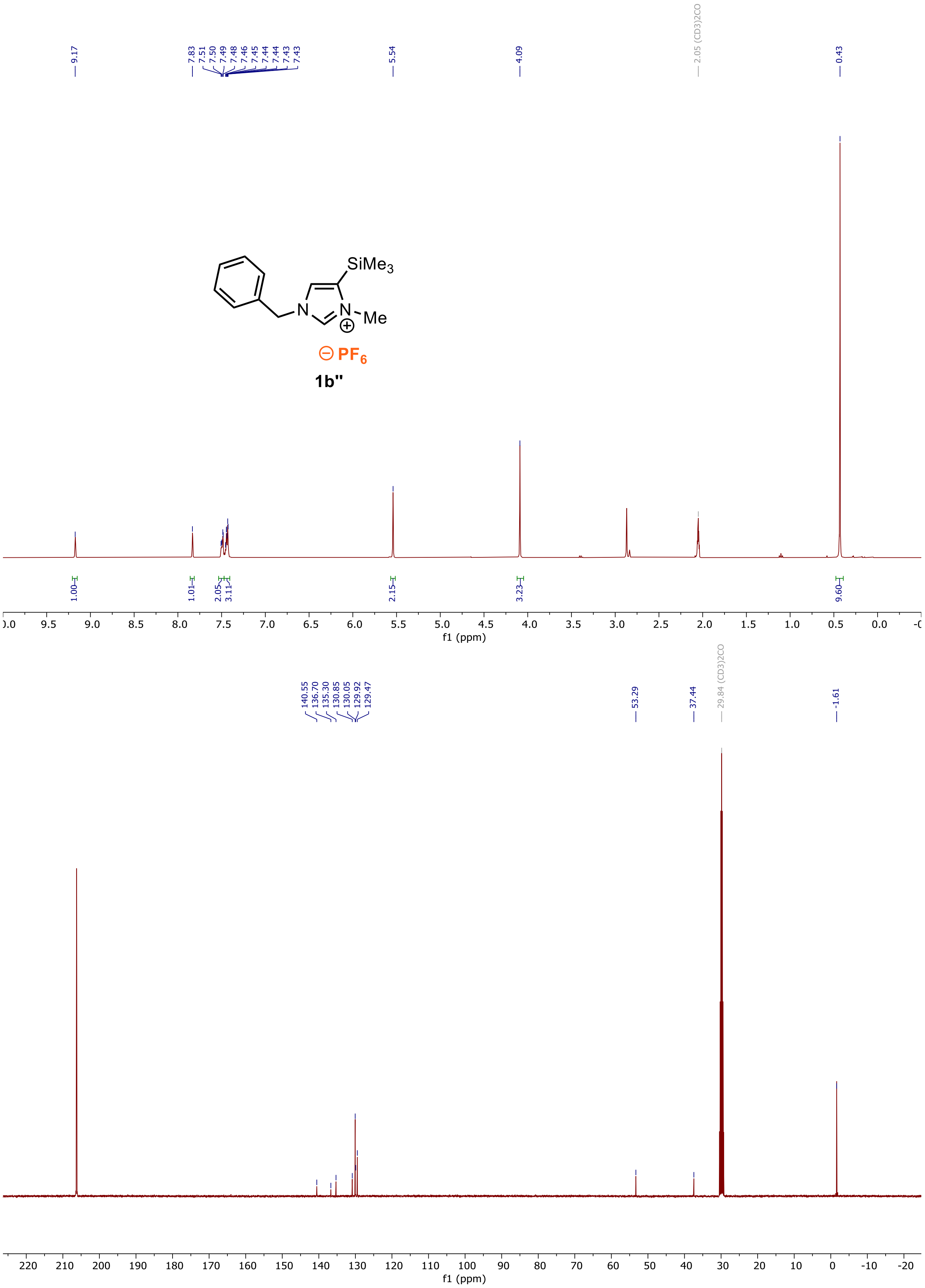


**Figure S24**. Top: $^{1}$H NMR spectrum (400 MHz), and bottom: $^{13}$C NMR spectrum (101 MHz) of **1b''** in Acetone-$d_6$.

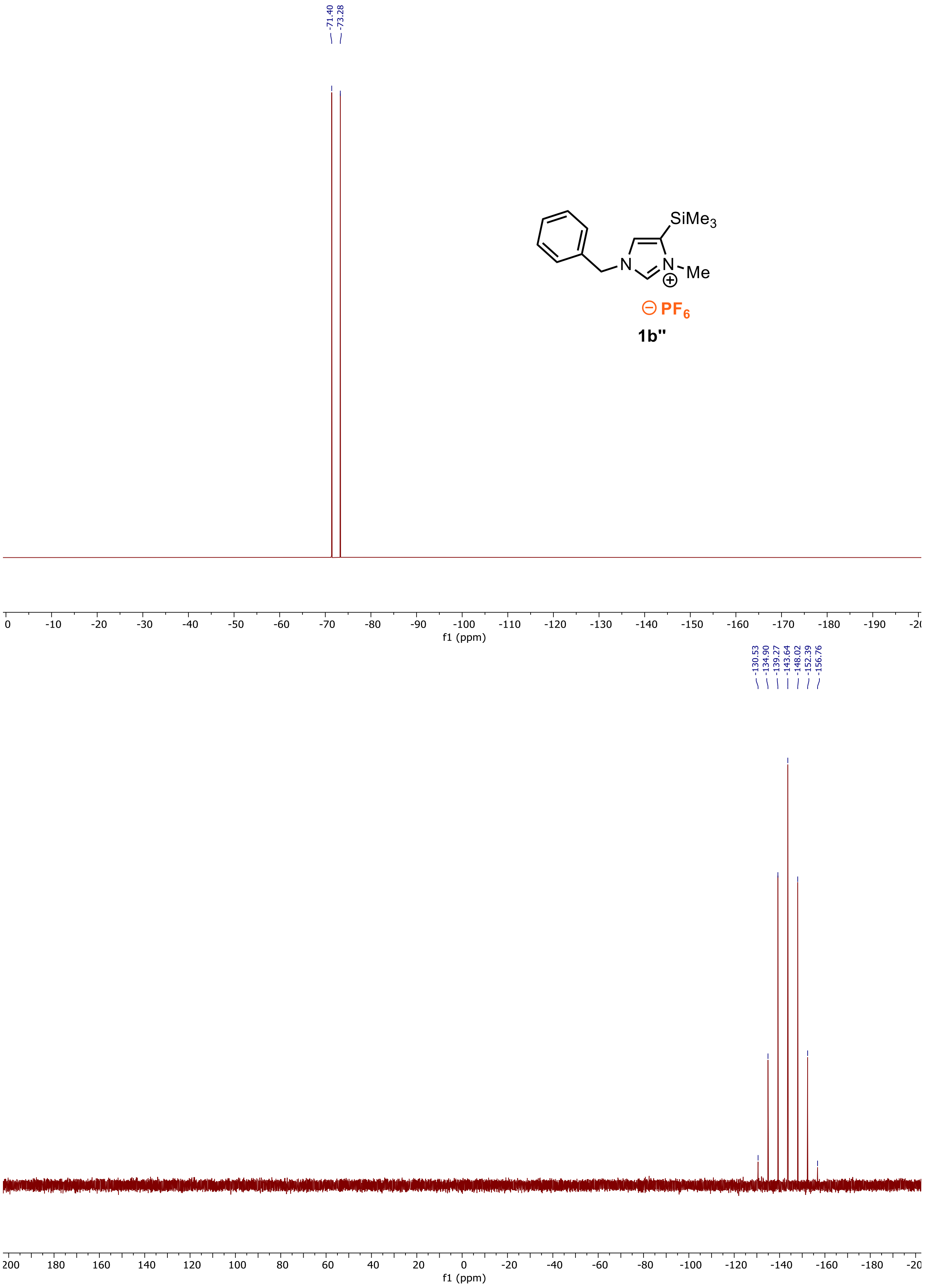


**Figure S25**. Top: $^{19}$F NMR spectrum (376 MHz), and bottom: $^{31}$P NMR spectrum (162 MHz) of **1b''** in Acetone-$d_6$.

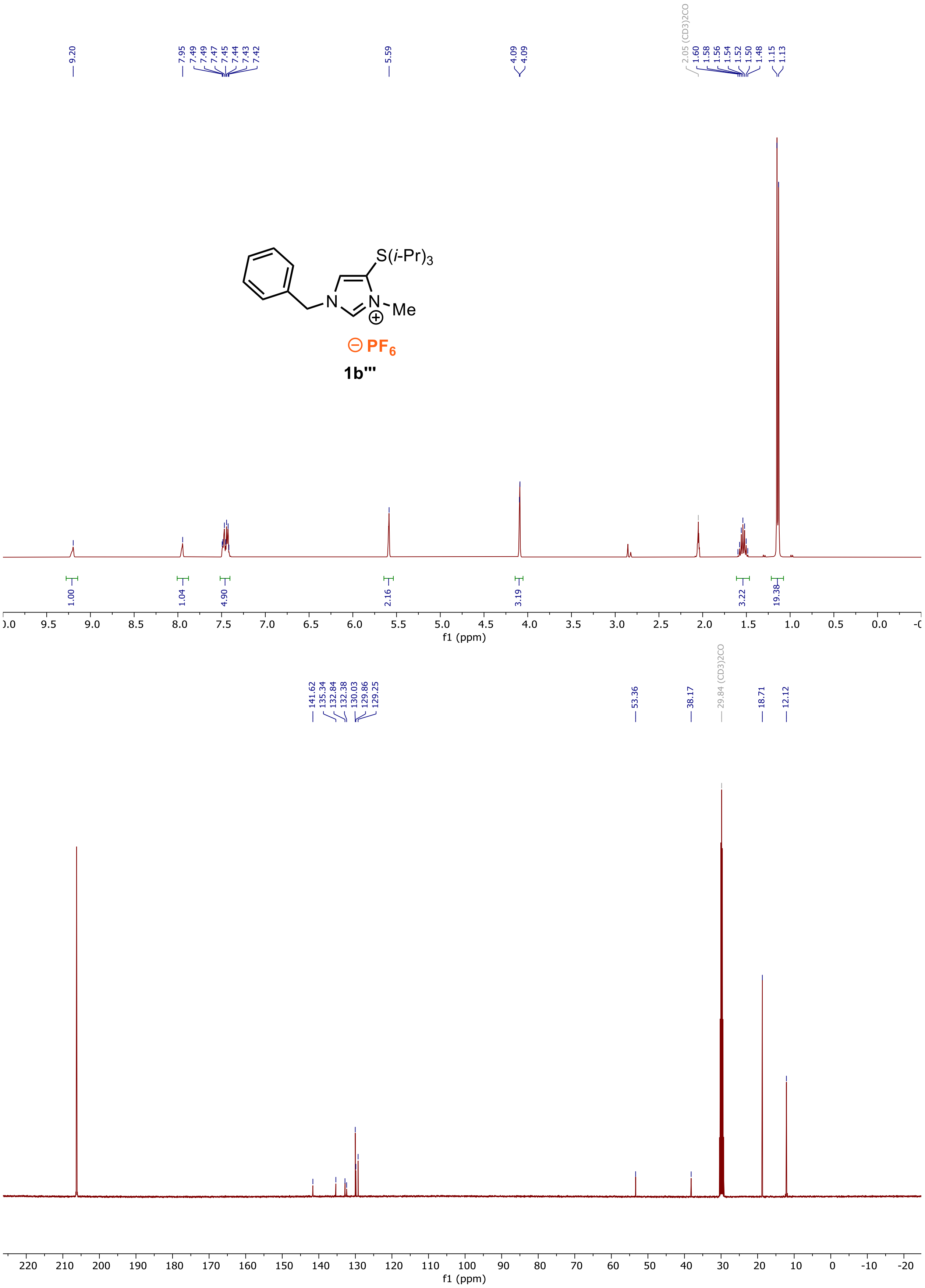


**Figure S26**. Top: $^{1}H$ NMR spectrum (400 MHz), and bottom: $^{13}C$ NMR spectrum (101 MHz) of **1b’’’** in Acetone-$d_6$.

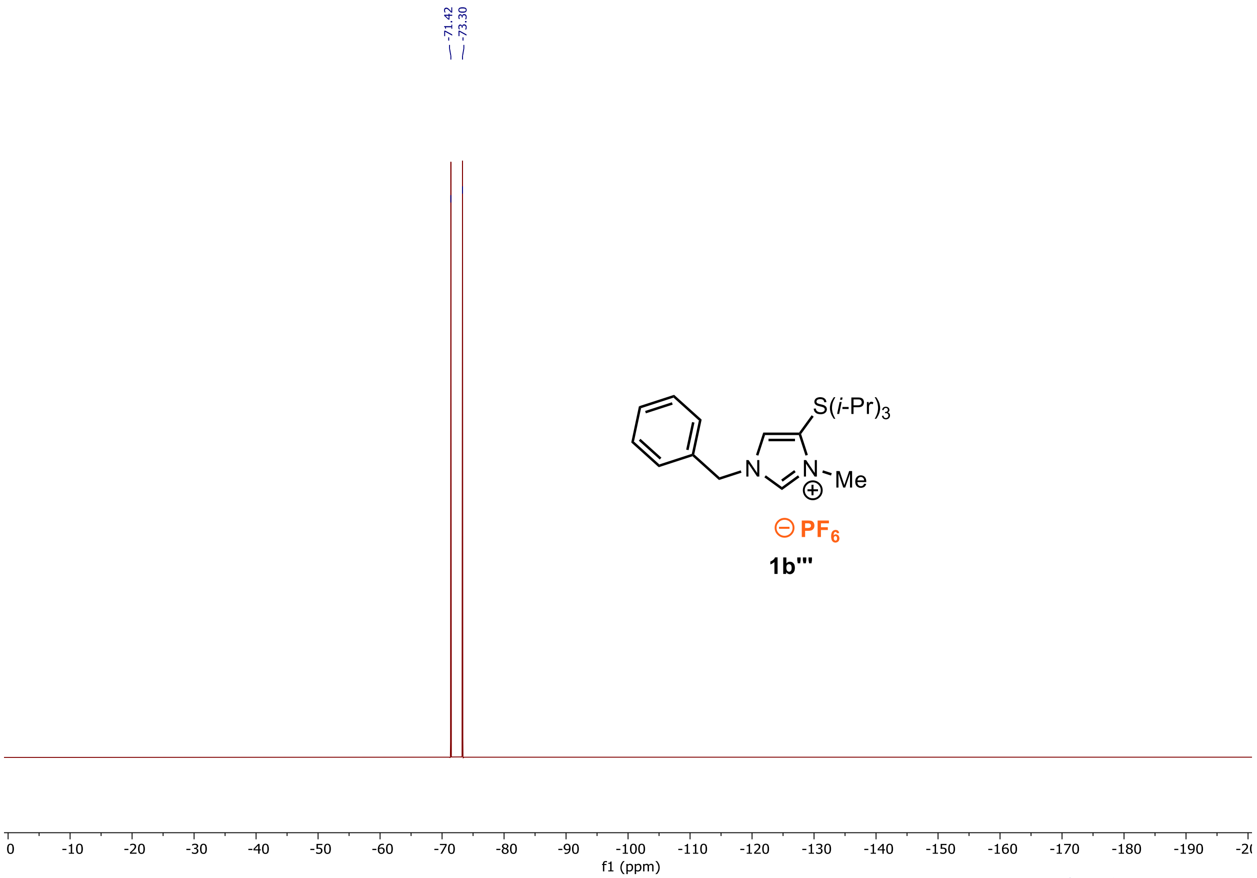


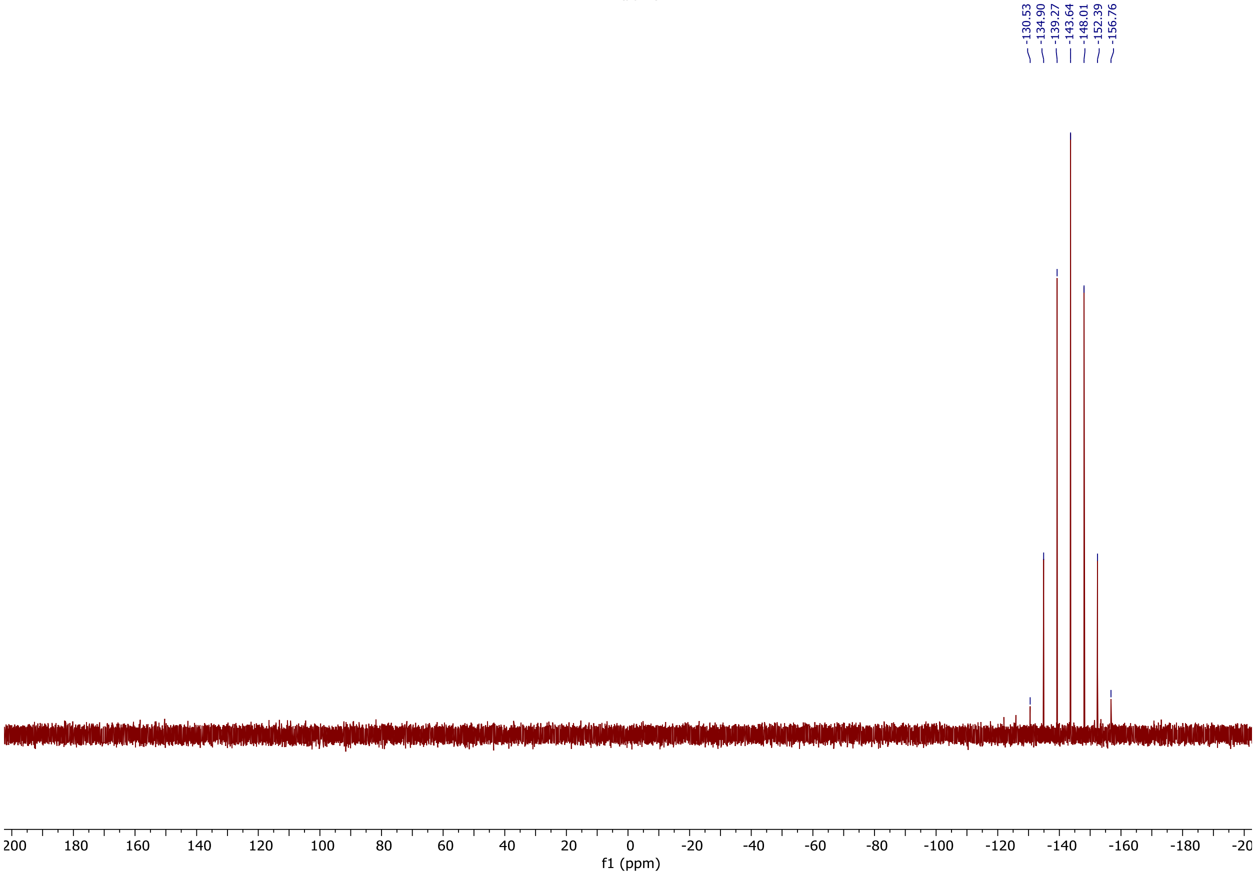


**Figure S27**. Top: $^{19}F$ NMR spectrum (376 MHz), and bottom: $^{31}P$ NMR spectrum (162 MHz) of **1b'''** in Acetone-$d_6$.

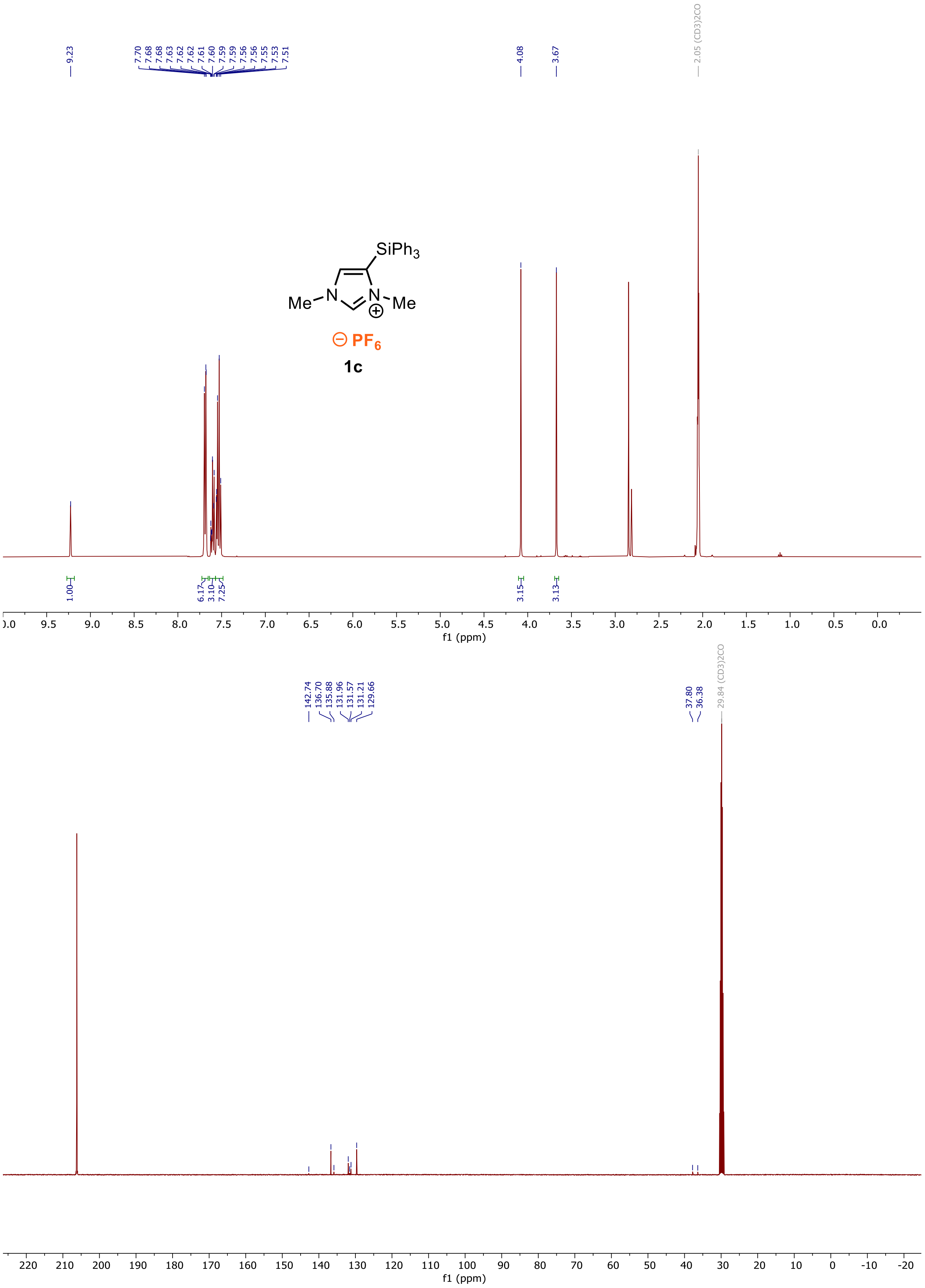


**Figure S28**. Top: $^{1}H$ NMR spectrum (400 MHz), and bottom: $^{13}C$ NMR spectrum (101 MHz) of **1c** in Acetone-$d_6$.

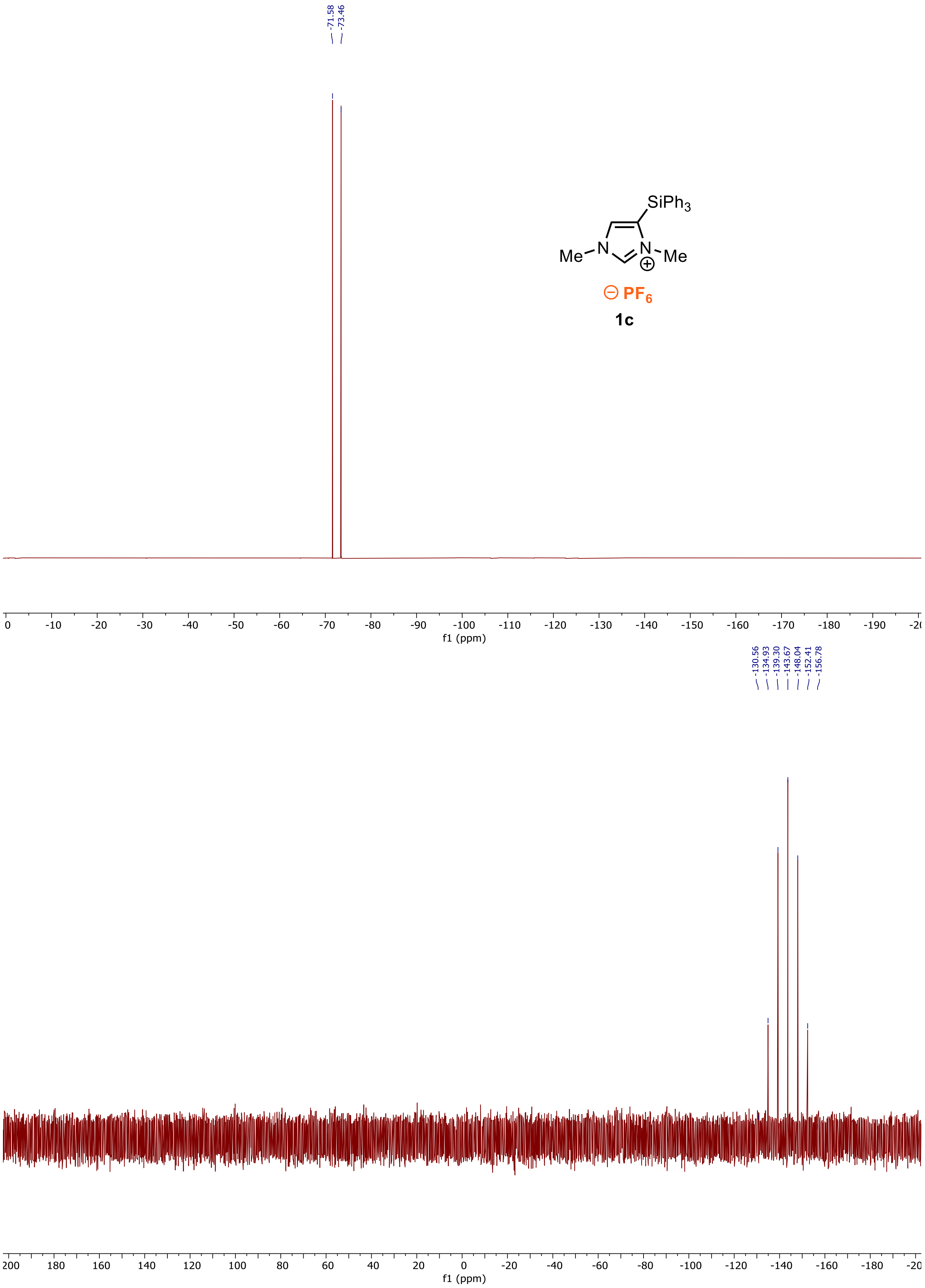


**Figure S29**. Top: $^{19}F$ NMR spectrum (376 MHz), and bottom: $^{31}P$ NMR spectrum (162 MHz) of **1c** in Acetone-$d_6$.

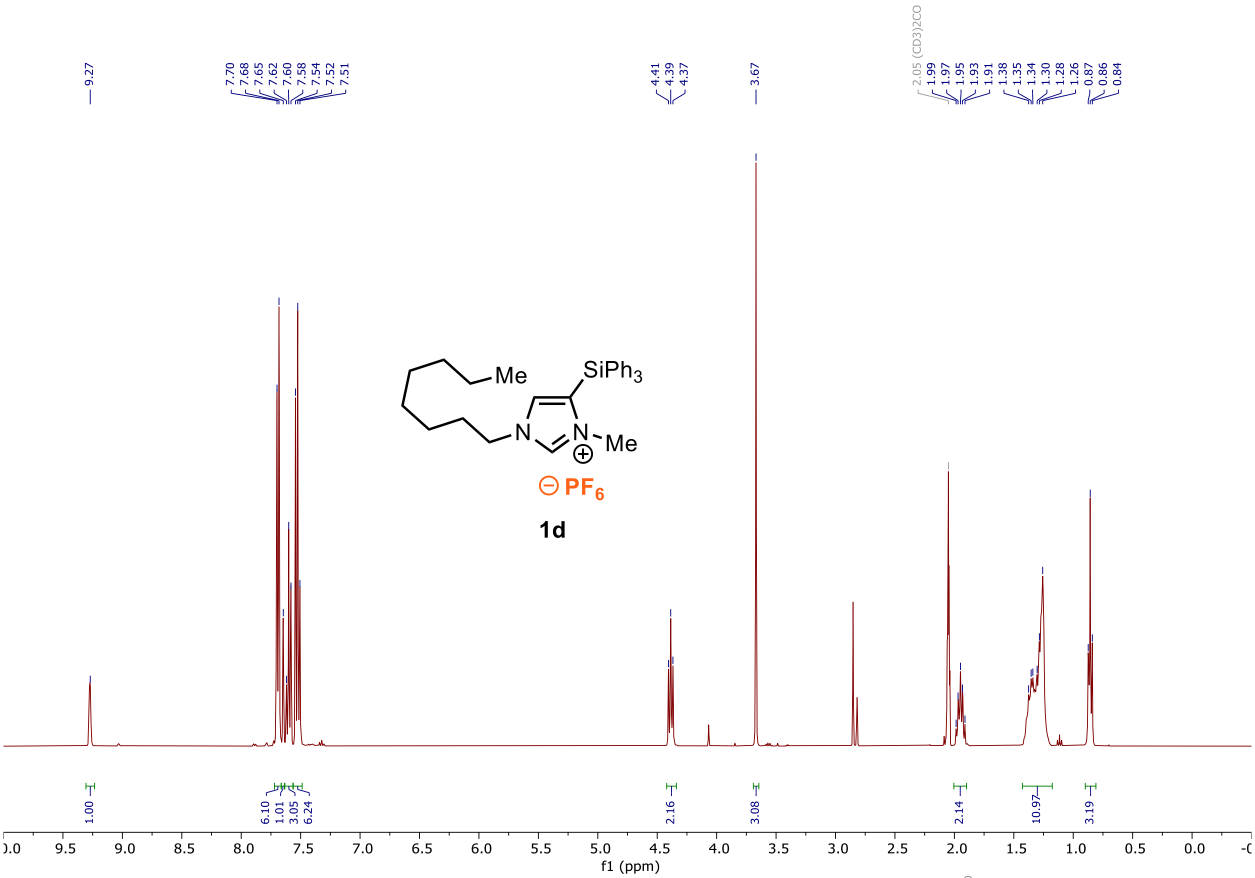


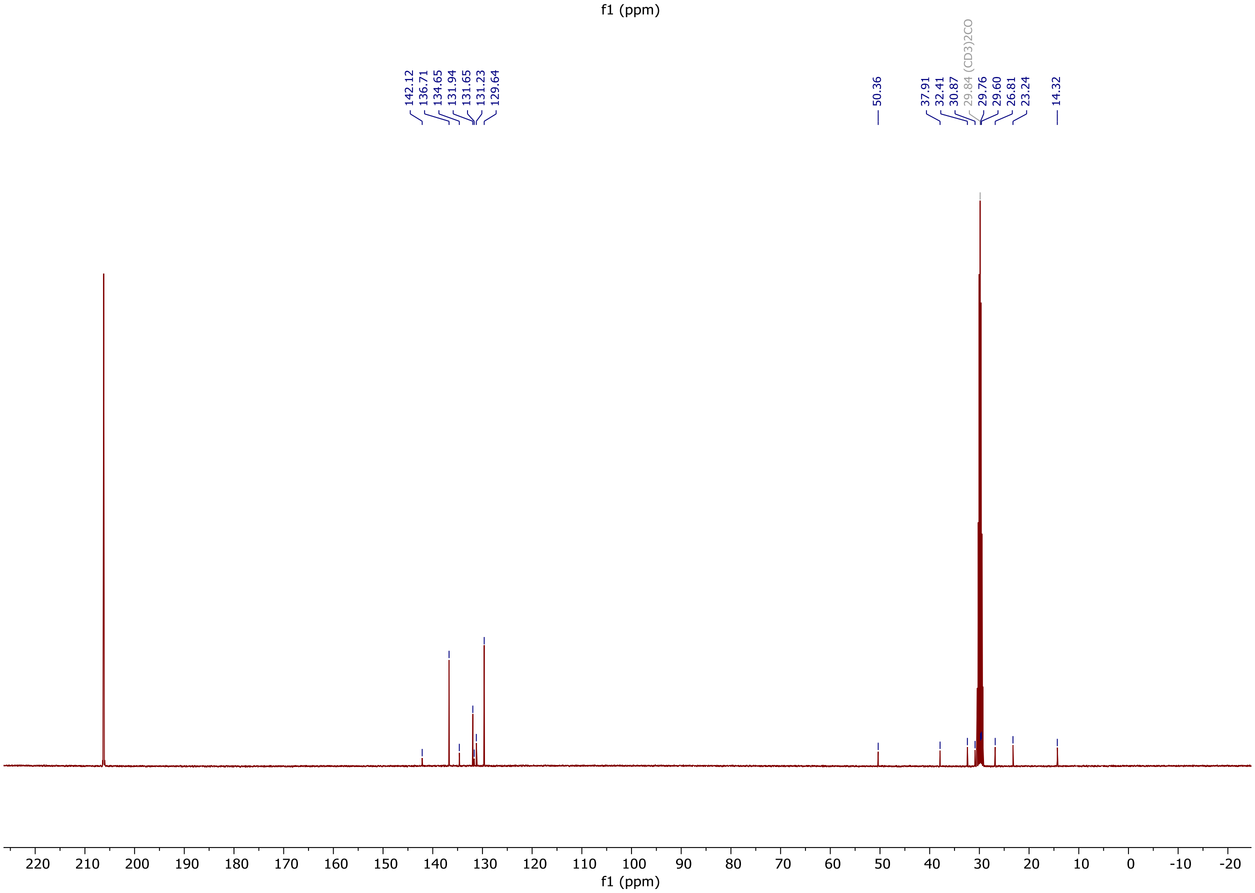


**Figure S30**. Top: $^{1}$H NMR spectrum (400 MHz), and bottom: $^{13}$C NMR spectrum (101 MHz) of **1d** in Acetone-$d_6$.

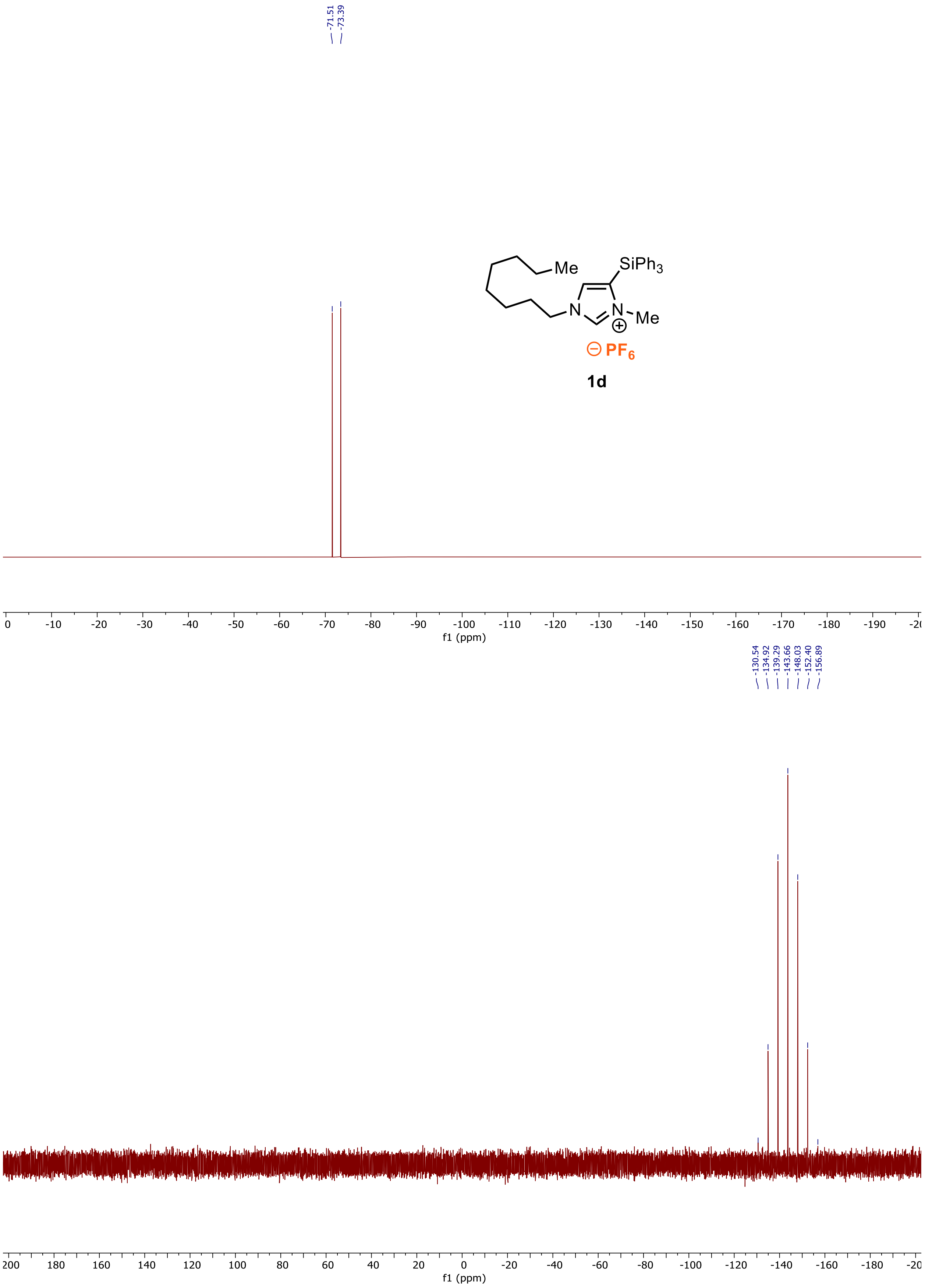


**Figure S31**. Top: $^{19}F$ NMR spectrum (376 MHz), and bottom: $^{31}P$ NMR spectrum (162 MHz) of **1d** in Acetone-$d_6$.

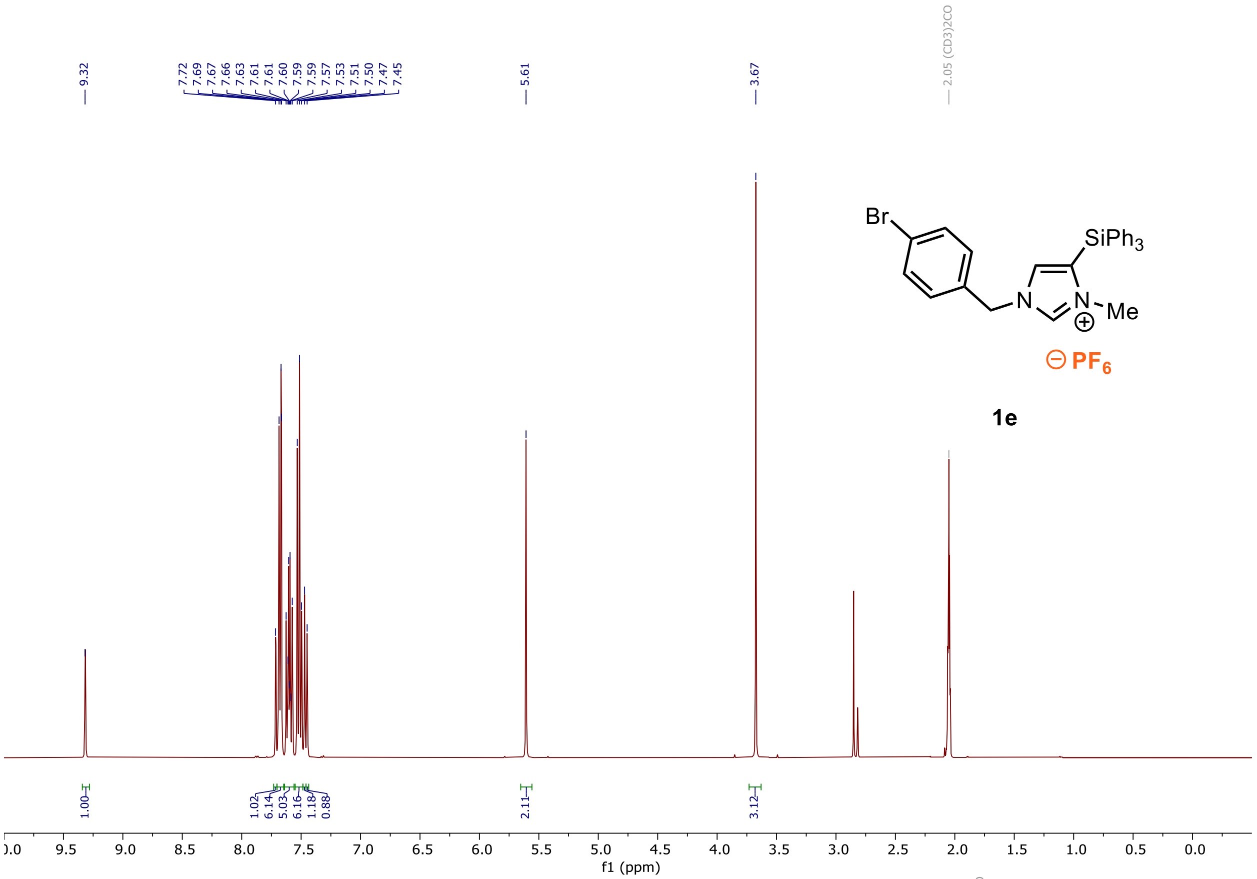


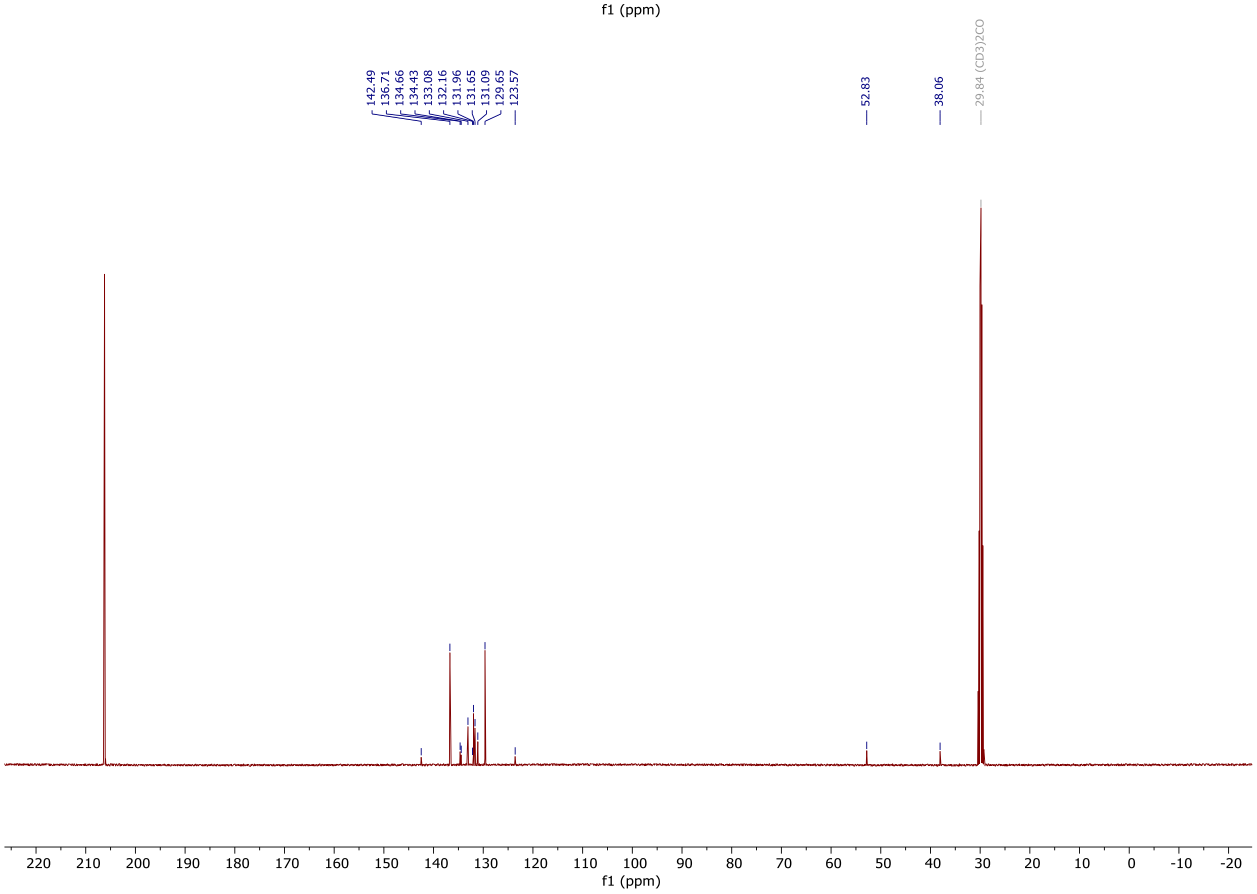


**Figure S32**. Top: $^{1}$H NMR spectrum (400 MHz), and bottom: $^{13}$C NMR spectrum (101 MHz) of **1e** in Acetone-$d_6$.

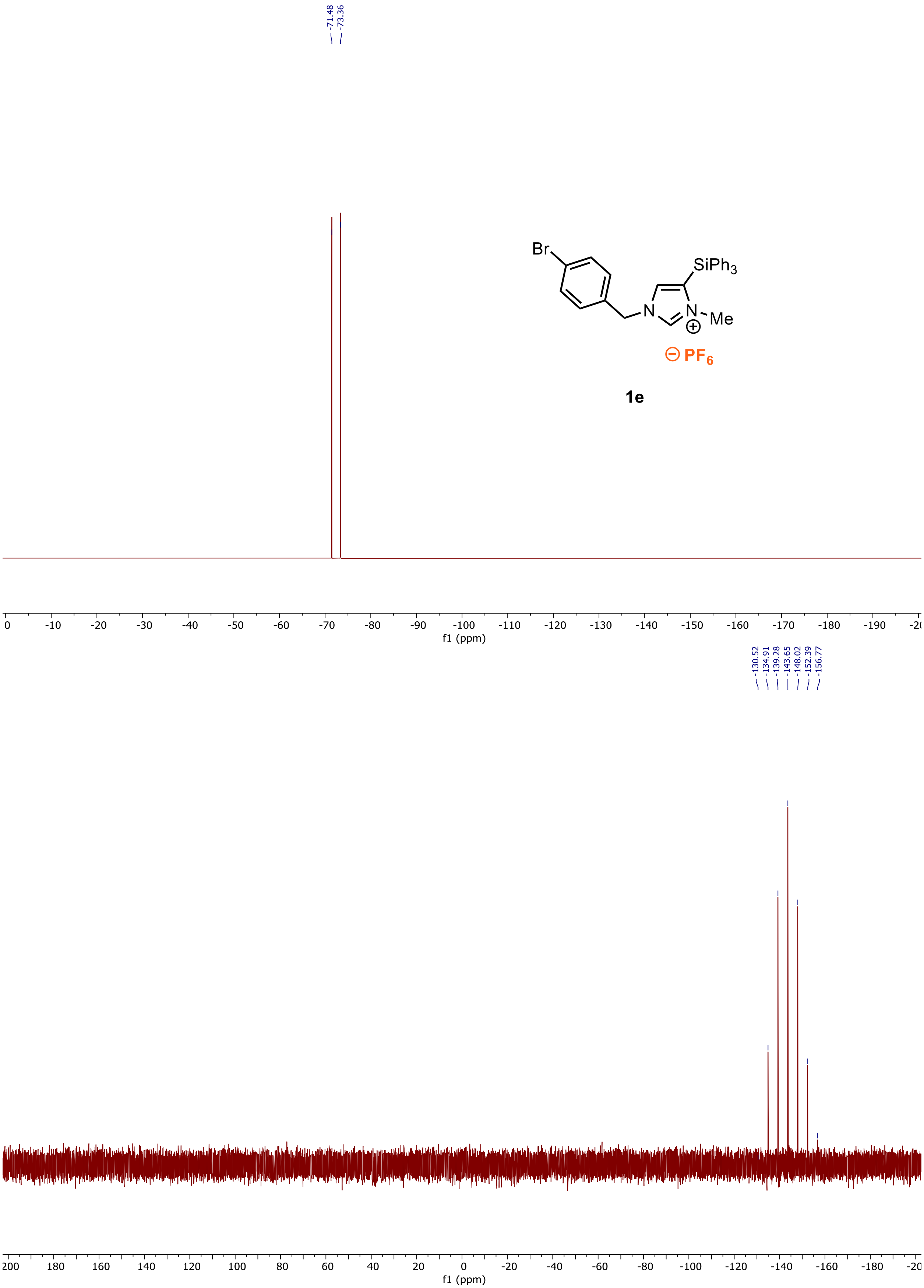


**Figure S33**. Top: $^{19}F$ NMR spectrum (376 MHz), and bottom: $^{31}P$ NMR spectrum (162 MHz) of **1e** in Acetone-$d_6$.

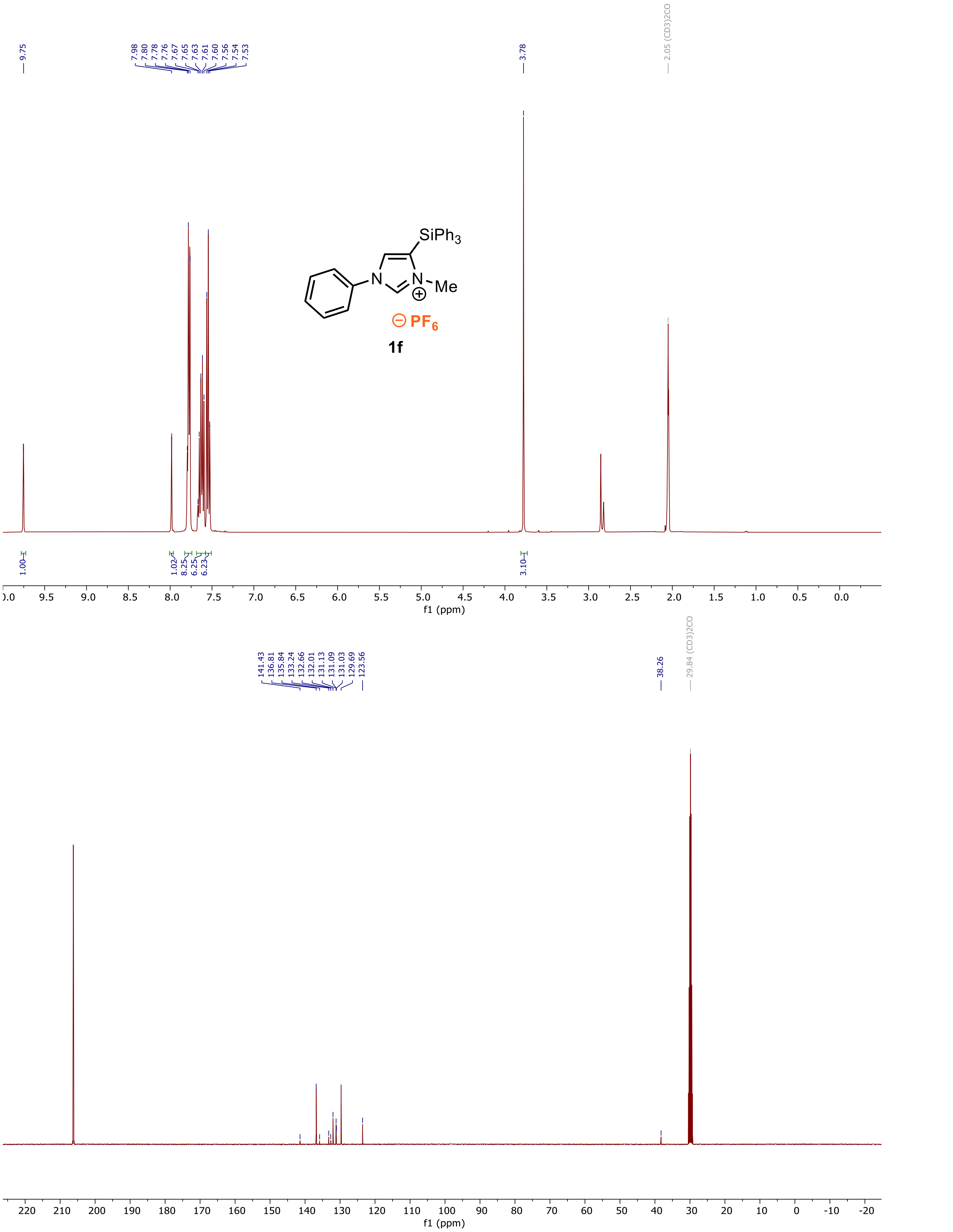


**Figure S34**. Top: $^{1}H$ NMR spectrum (400 MHz), and bottom: $^{13}C$ NMR spectrum (101 MHz) of **1f** in Acetone-$d_6$.

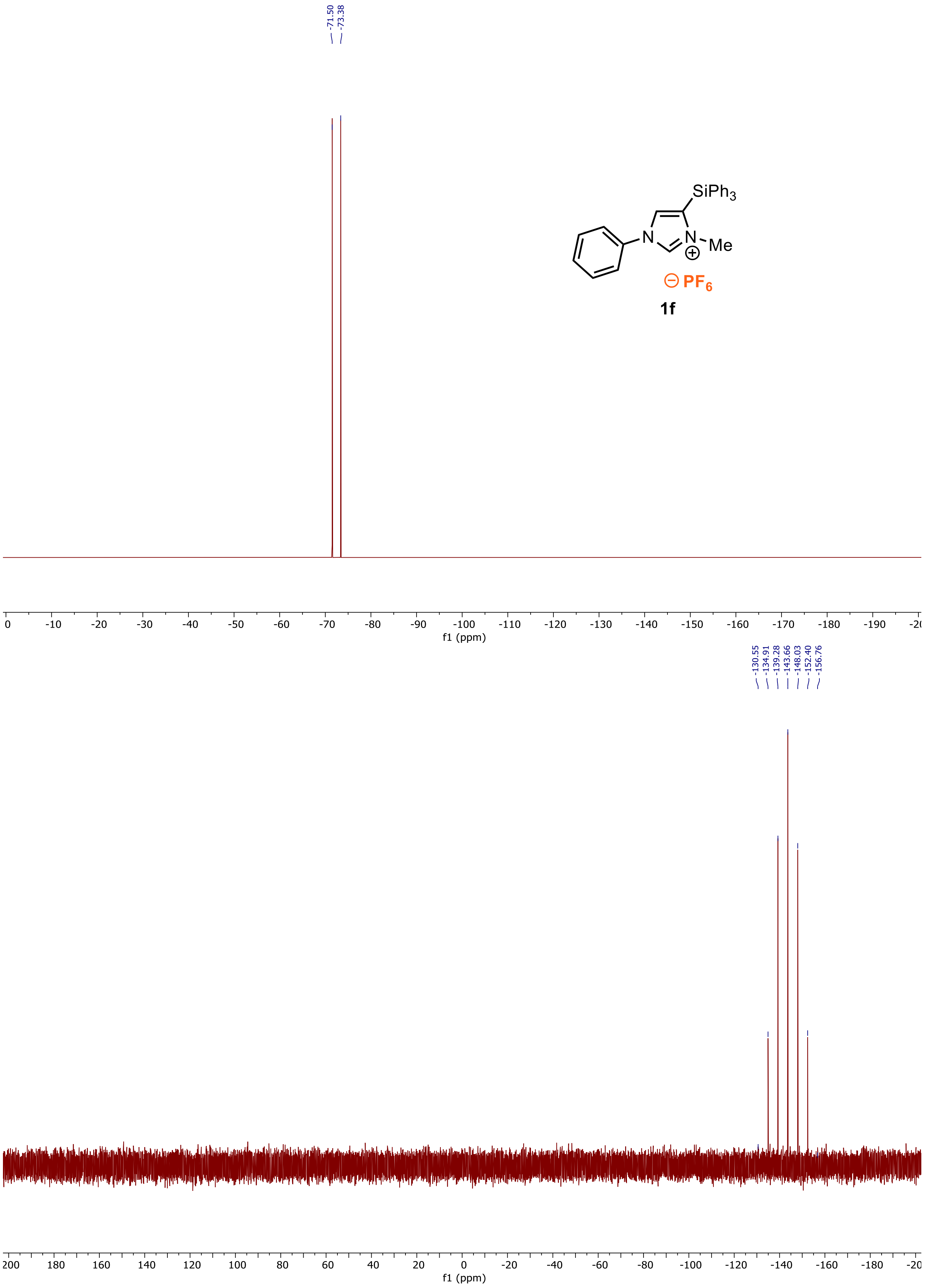


**Figure S35**. Top: $^{19}F$ NMR spectrum (376 MHz), and bottom: $^{31}P$ NMR spectrum (162 MHz) of **1f** in Acetone-$d_6$.

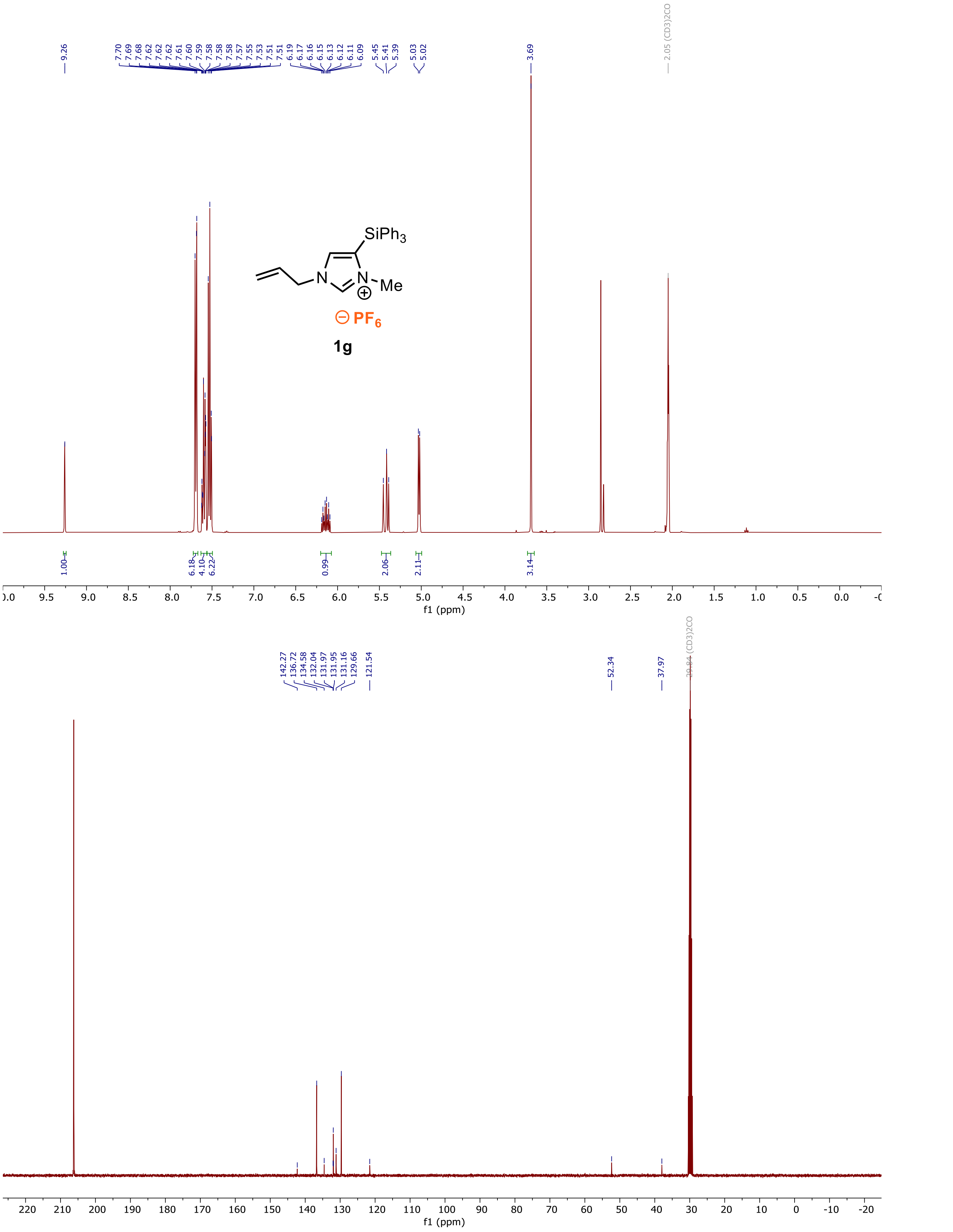


**Figure S36**. Top: $^{1}$H NMR spectrum (400 MHz), and bottom: $^{13}$C NMR spectrum (100 MHz) of **1g** in Acetone-$d_6$.

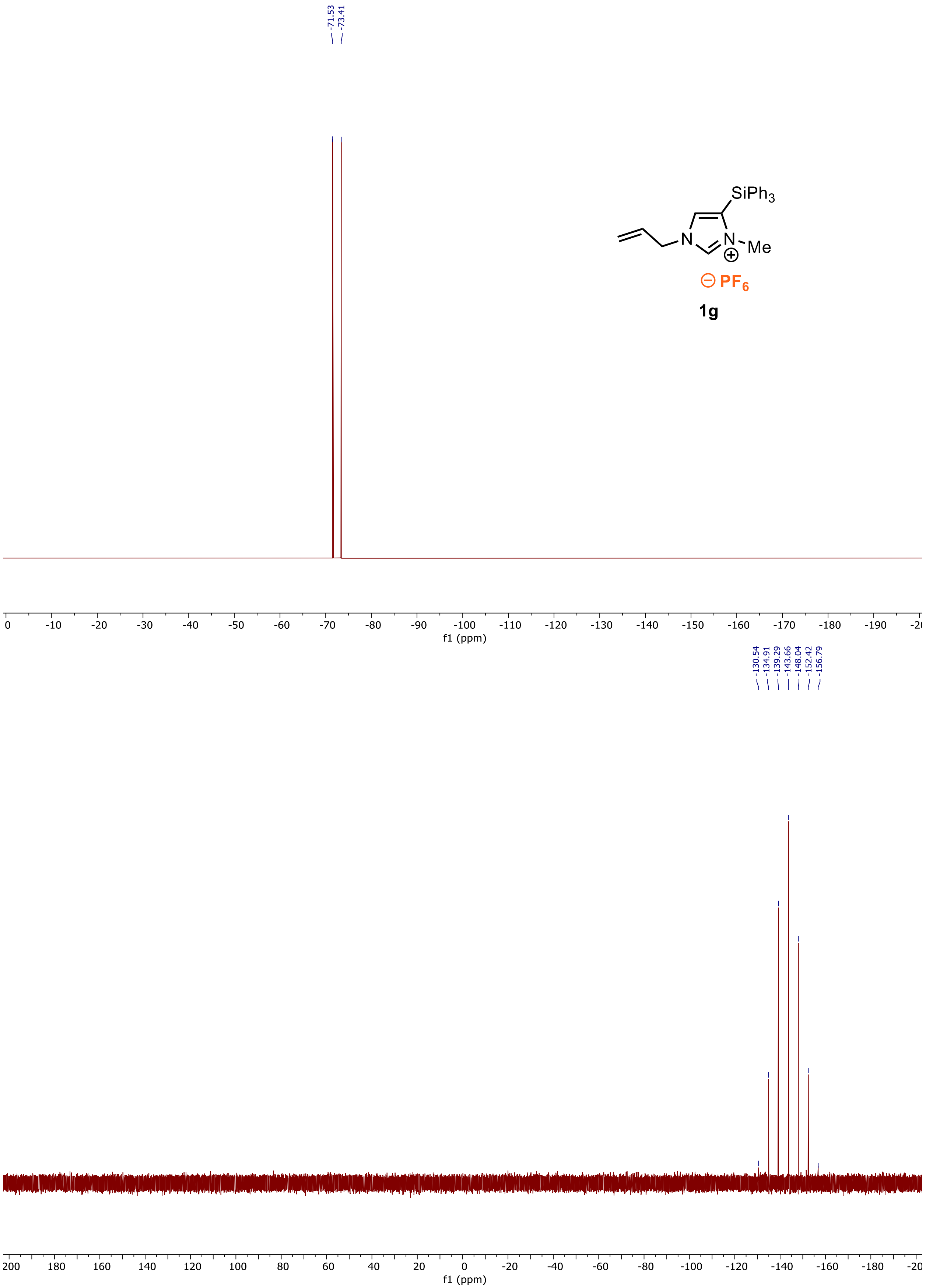


**Figure S37**. Top: $^{19}$F NMR spectrum (376 MHz), and bottom: $^{31}$P NMR spectrum (162 MHz) of **1g** in Acetone-$d_6$.

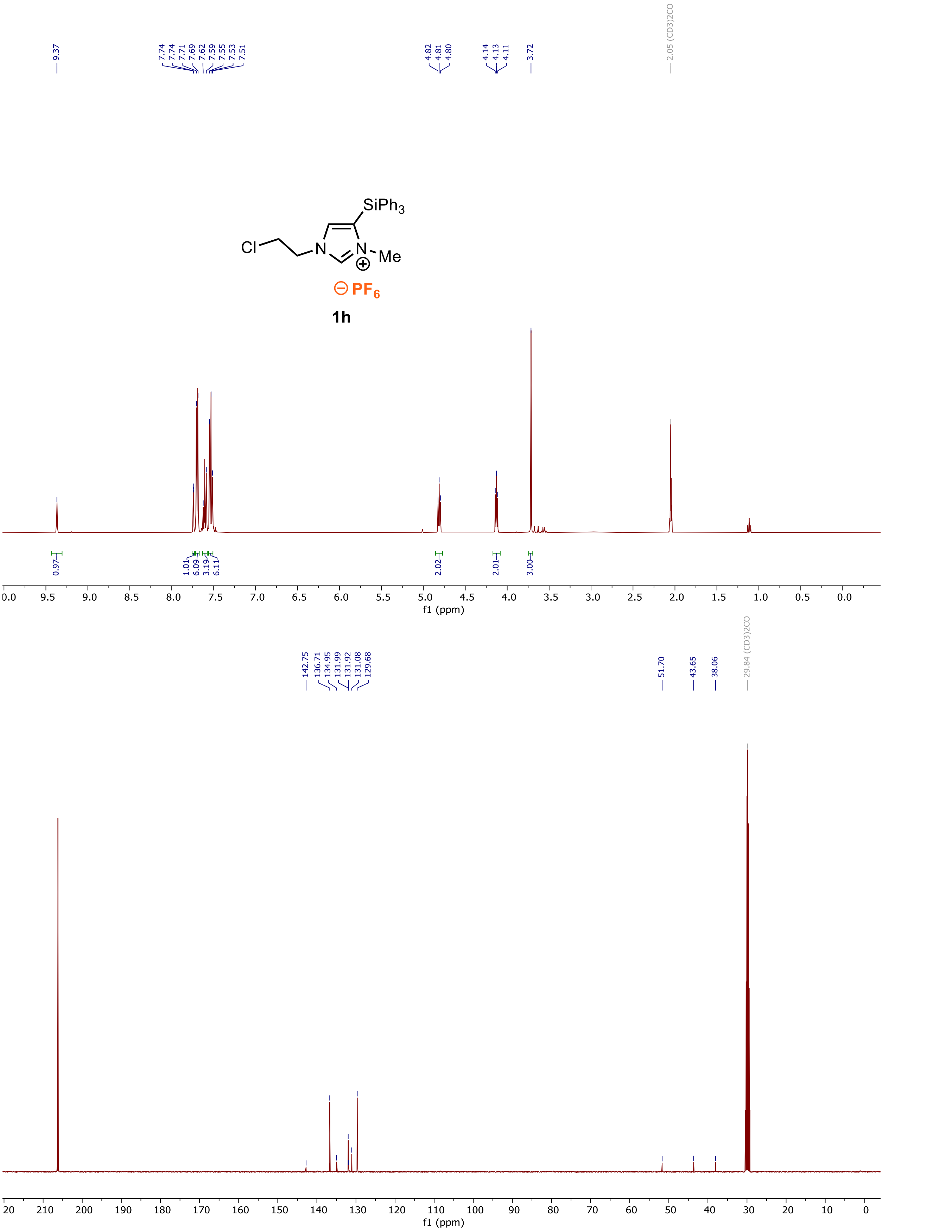


**Figure S38**. Top: $^{1}H$ NMR spectrum (400 MHz), and bottom: $^{13}C$ NMR spectrum (101 MHz) of **1h** in Acetone-$d_6$.

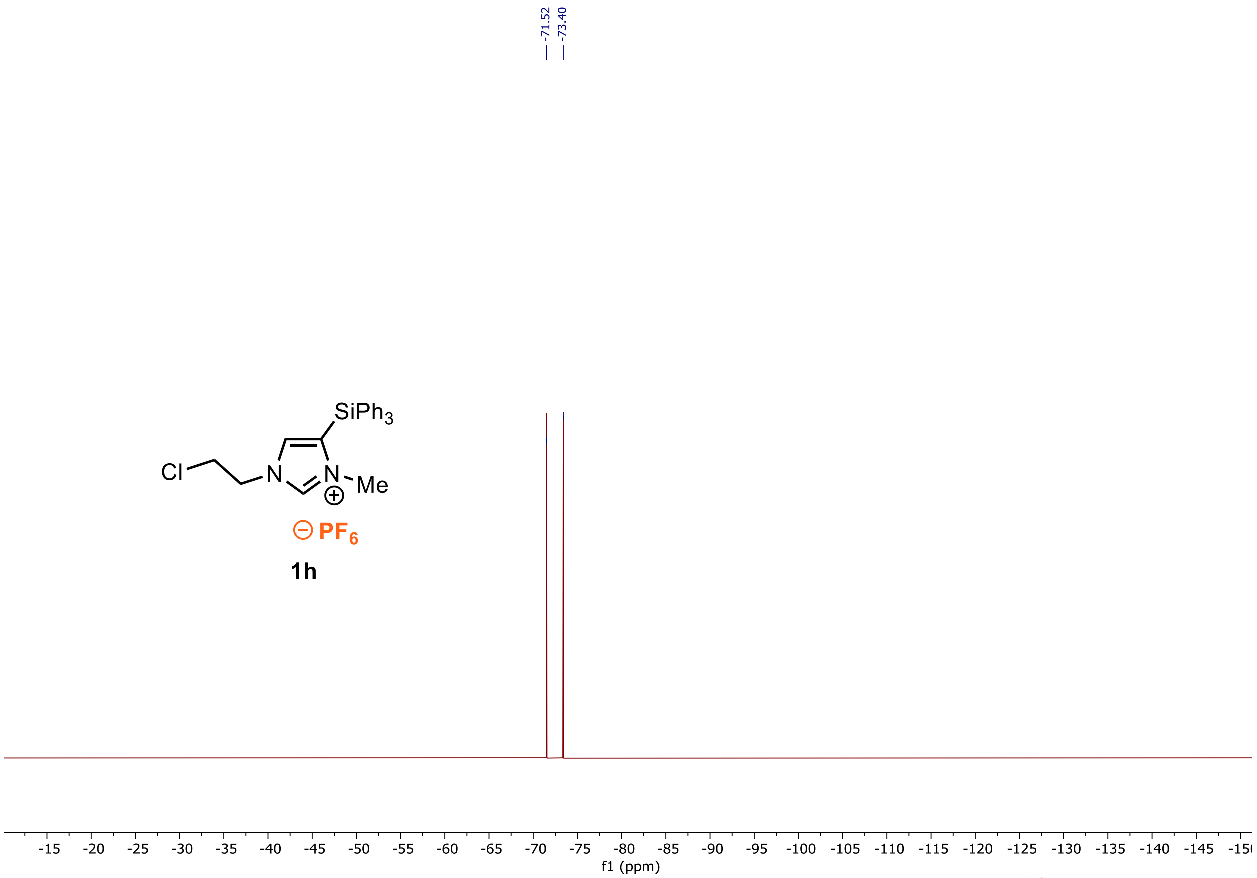


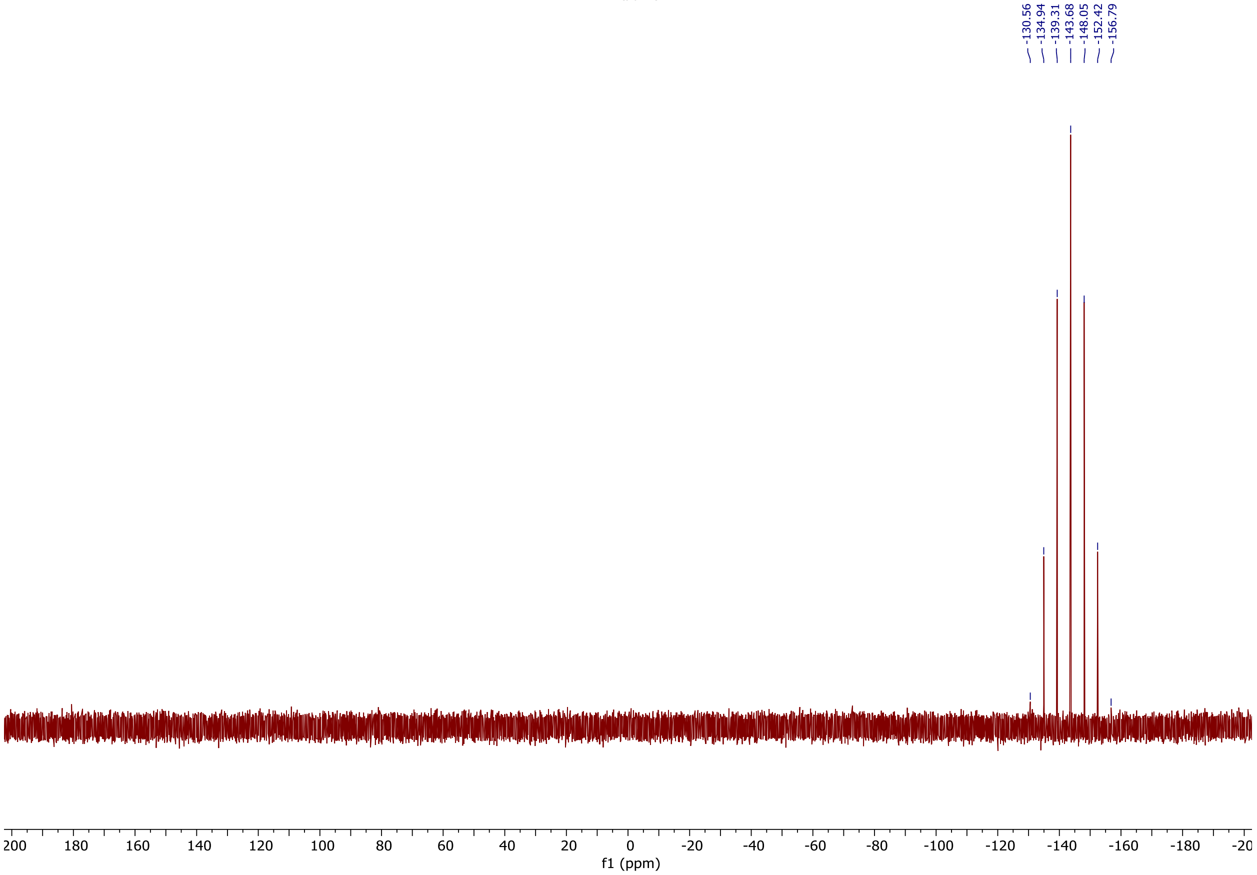


**Figure S39**. Top: $^{19}$F NMR spectrum (376 MHz), and bottom: $^{31}$P NMR spectrum (162 MHz) of **1h** in Acetone-$d_6$.

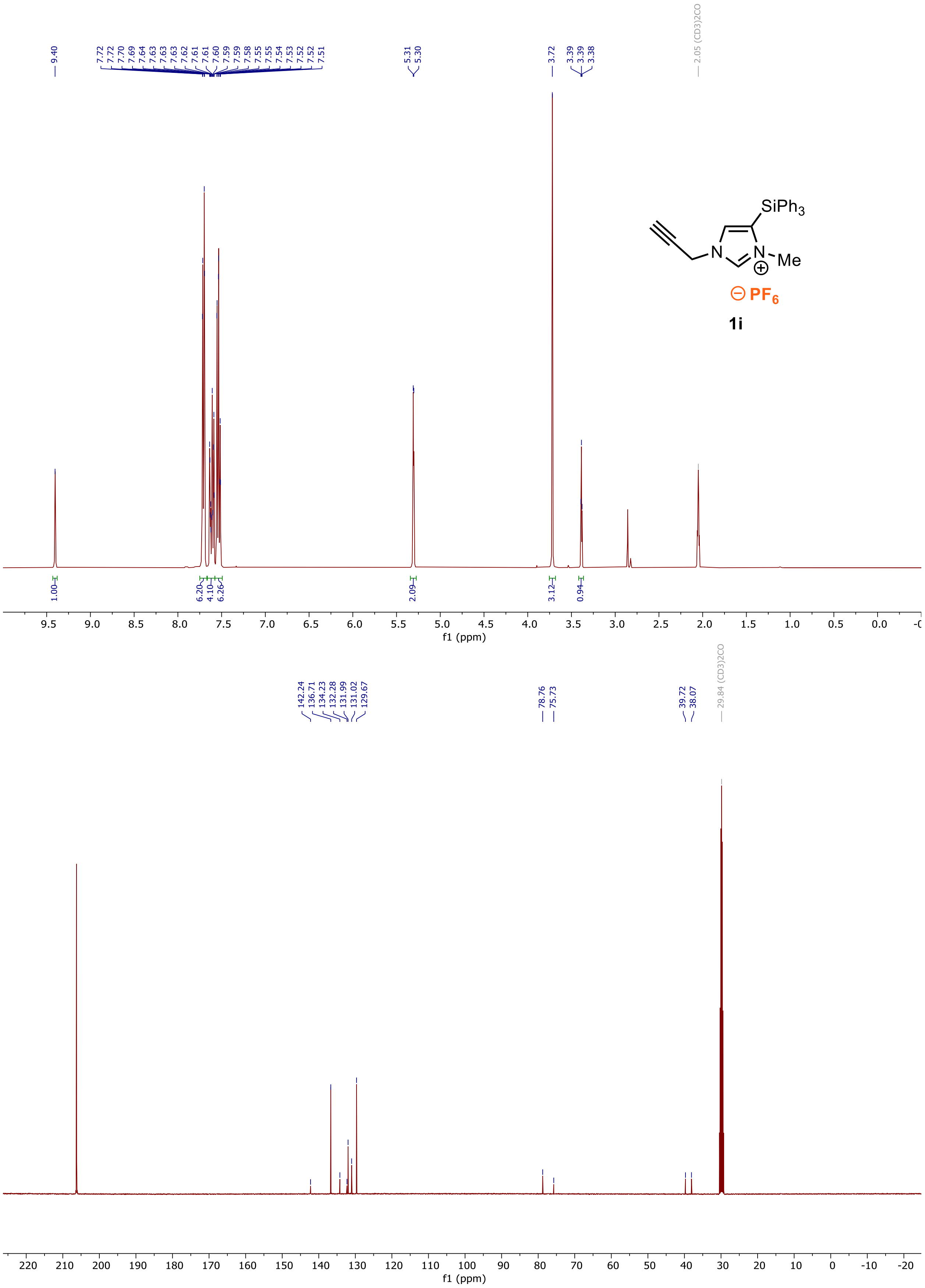


**Figure S40**. Top: $^{1}H$ NMR spectrum (400 MHz), and bottom: $^{13}C$ NMR spectrum (101 MHz) of **1i** in Acetone-$d_6$.

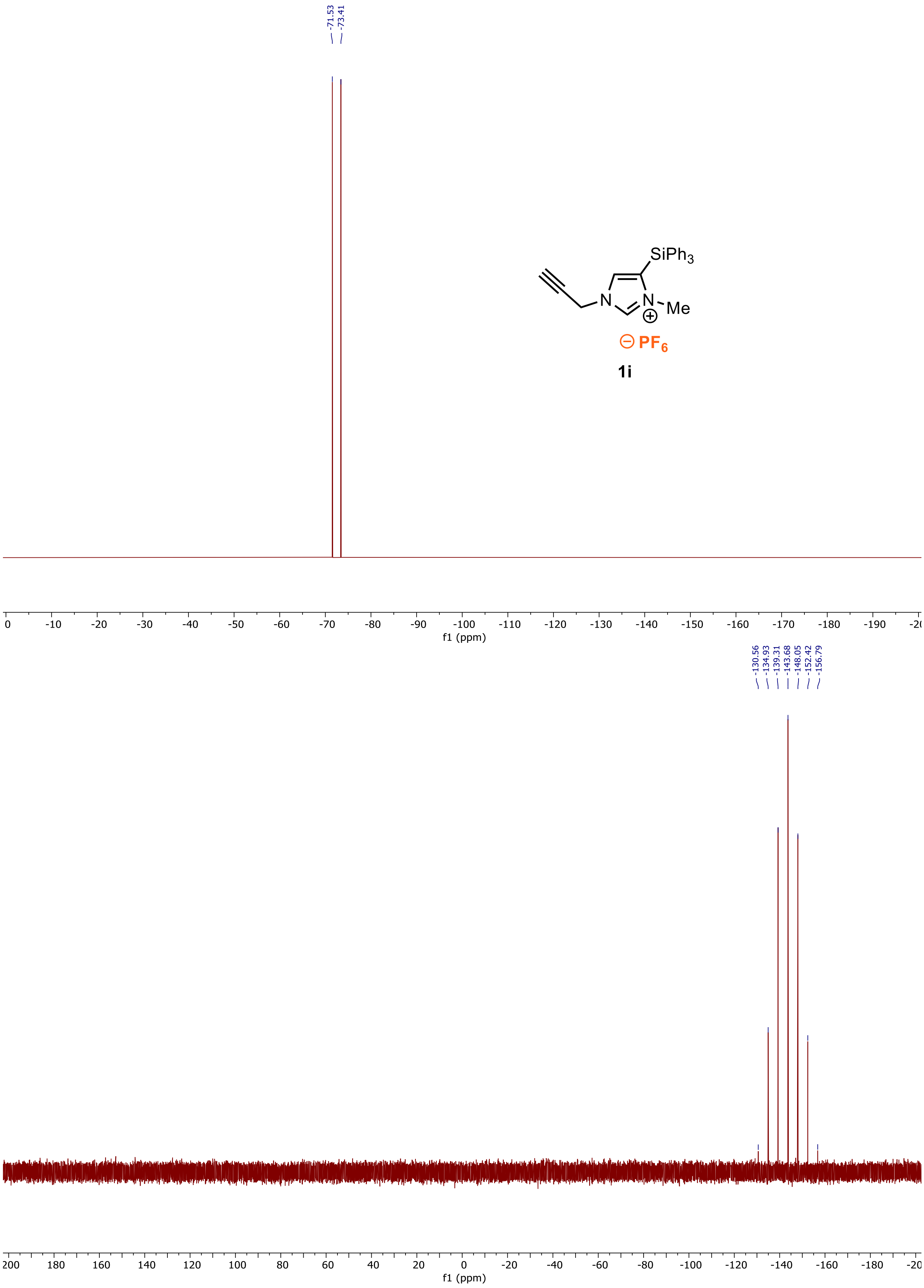


**Figure S41**. Top: $^{19}$F NMR spectrum (376 MHz), and bottom: $^{31}$P NMR spectrum (162 MHz) of **1i** in Acetone-$d_6$.

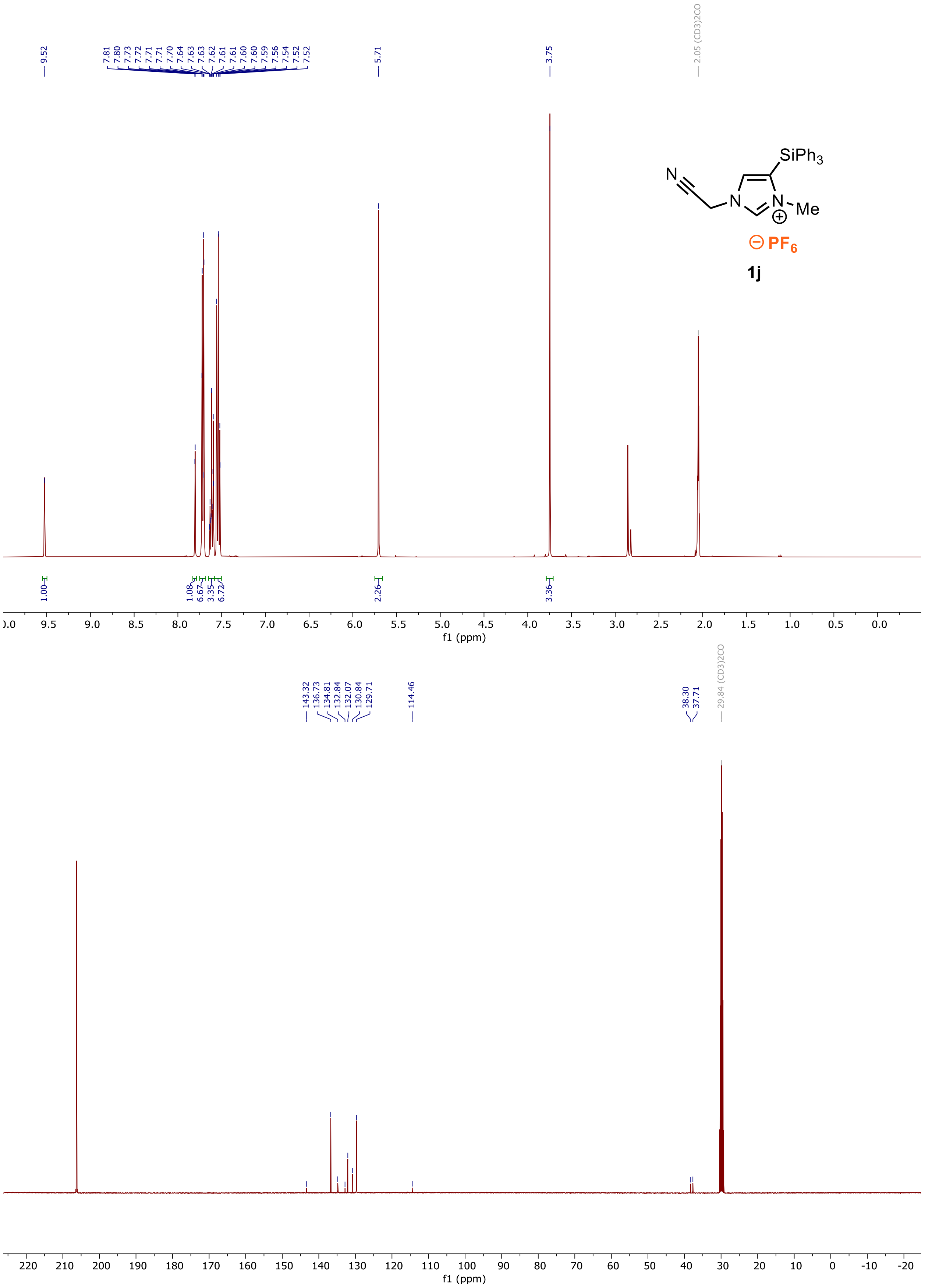


**Figure S42**. Top: $^{1}$H NMR spectrum (400 MHz), and bottom: $^{13}$C NMR spectrum (101 MHz) of **1j** in Acetone-$d_6$.

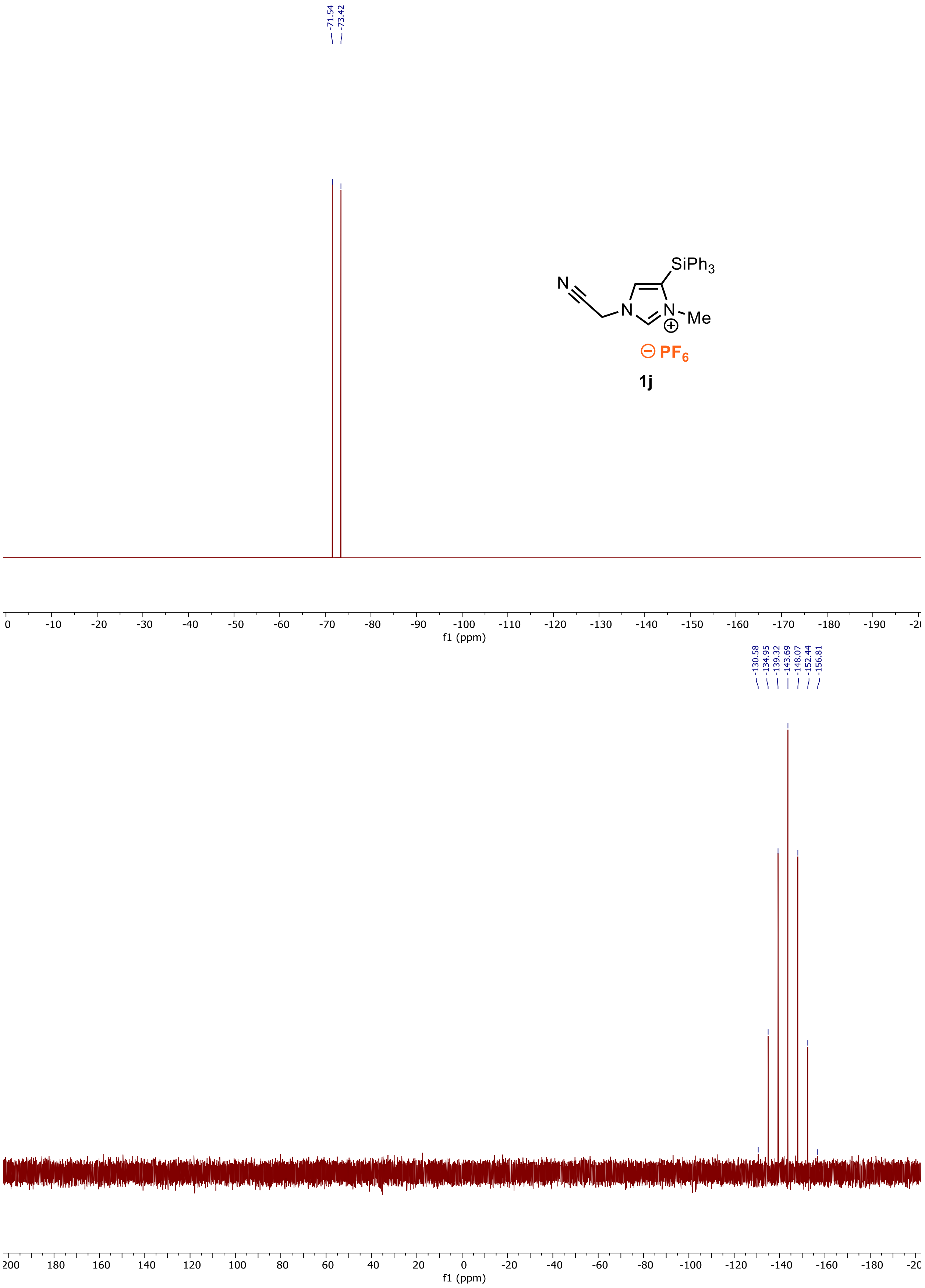


**Figure S43**. Top: $^{19}F$ NMR spectrum (376 MHz), and bottom: $^{31}P$ NMR spectrum (162 MHz) of **1j** in Acetone-$d_6$.

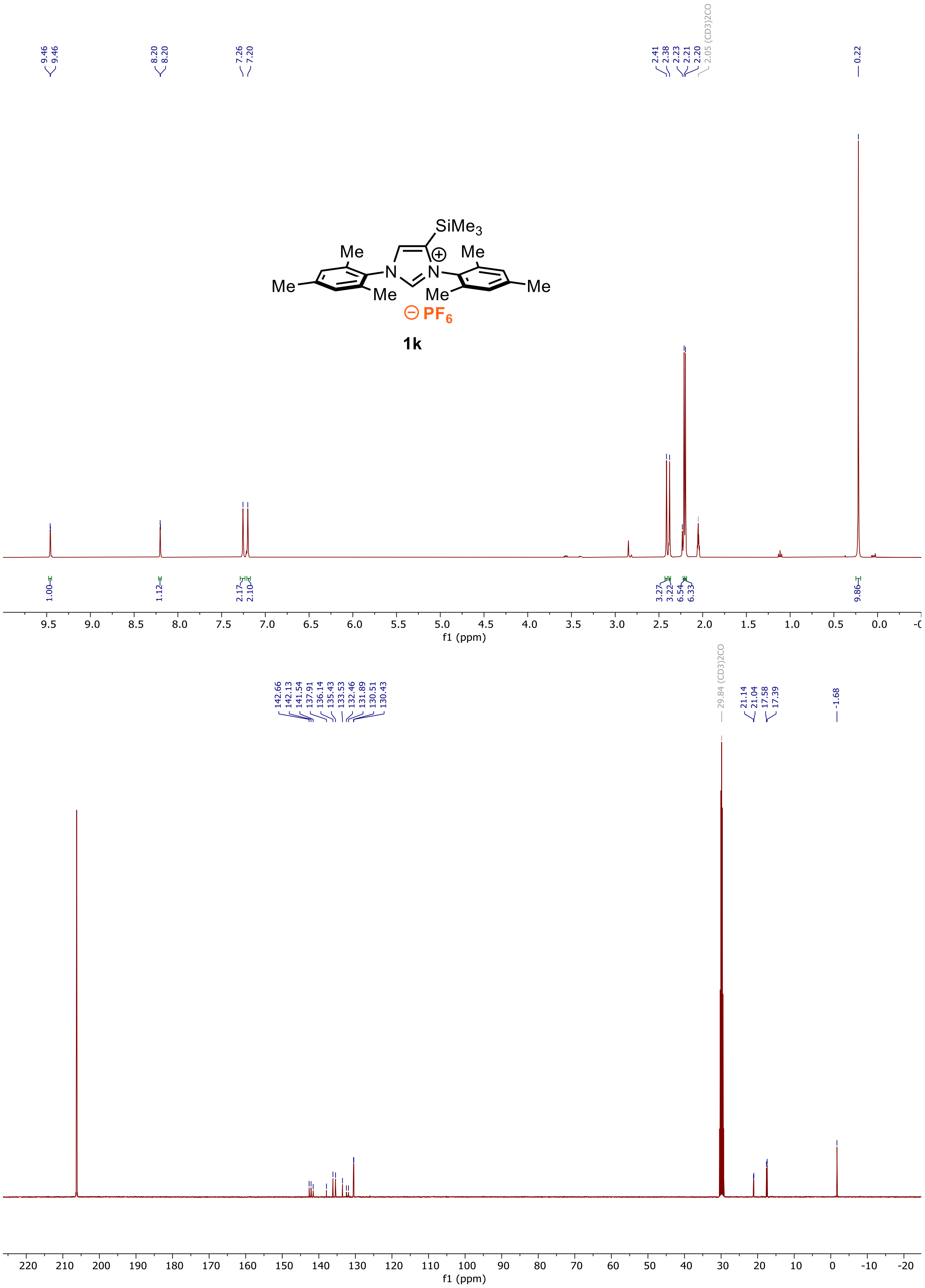


**Figure S44**. Top: $^{1}H$ NMR spectrum (400 MHz), and bottom: $^{13}C$ NMR spectrum (101 MHz) of **1k** in Acetone-$d_6$.

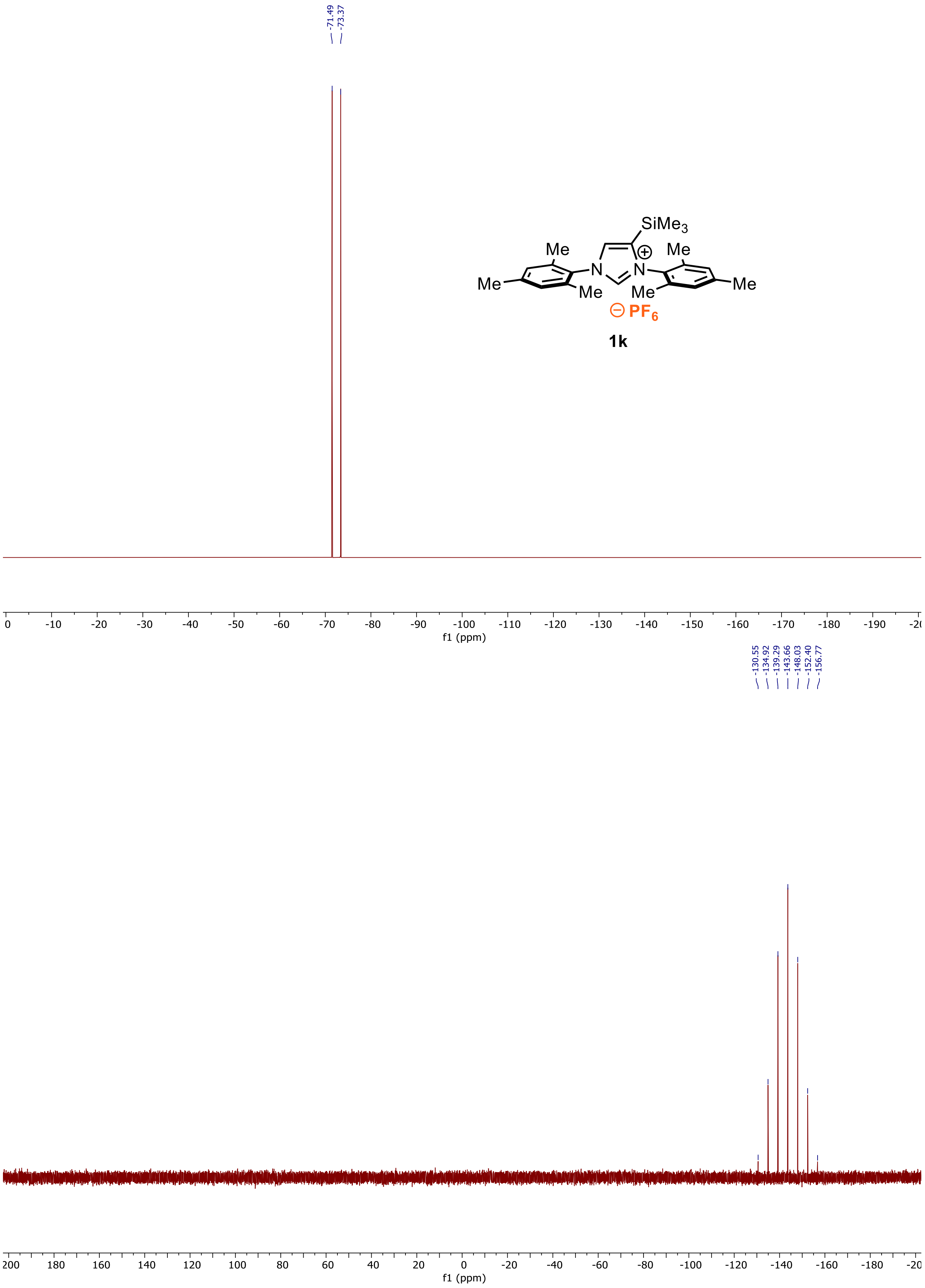


**Figure S45**. Top: $^{19}$F NMR spectrum (376 MHz), and bottom: $^{31}$P NMR spectrum (162 MHz) of **1k** in Acetone-$d_6$.

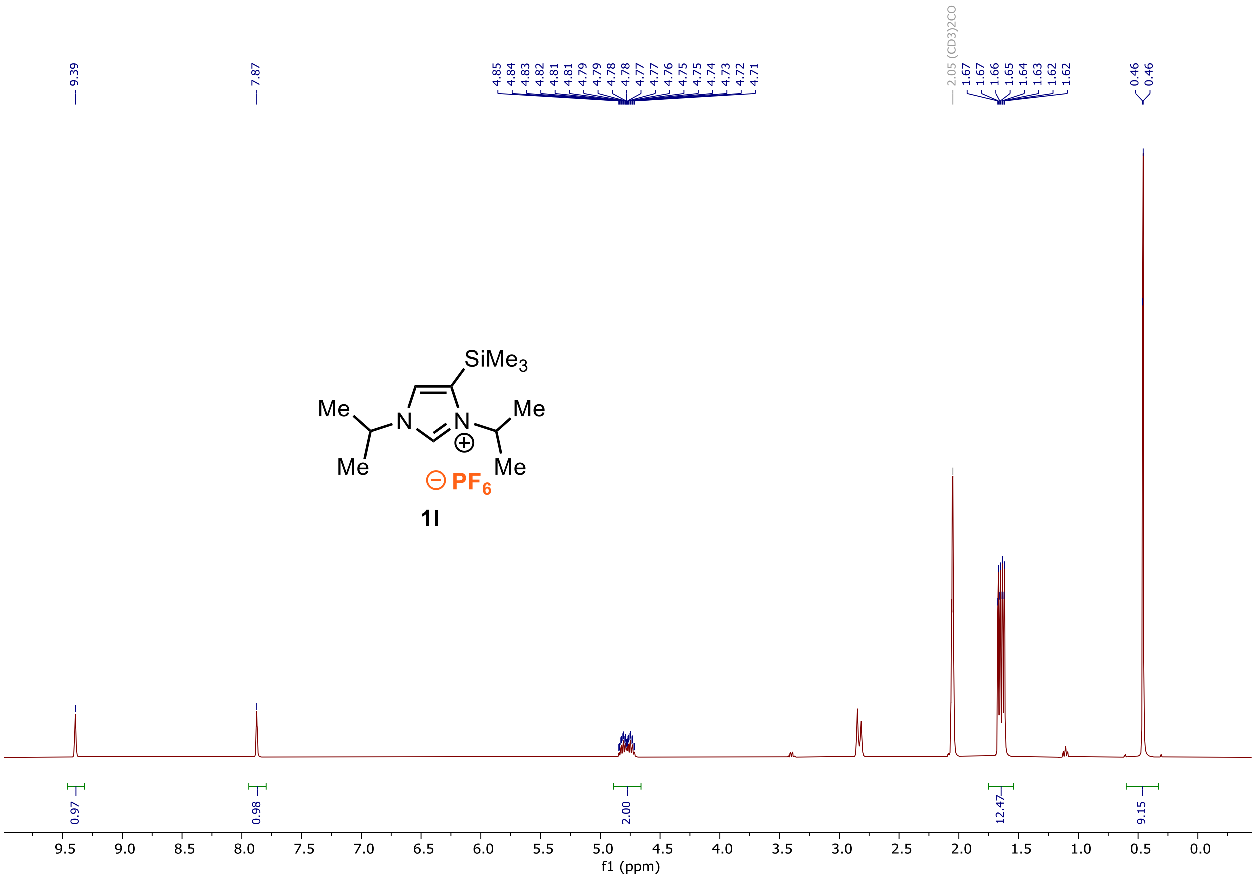


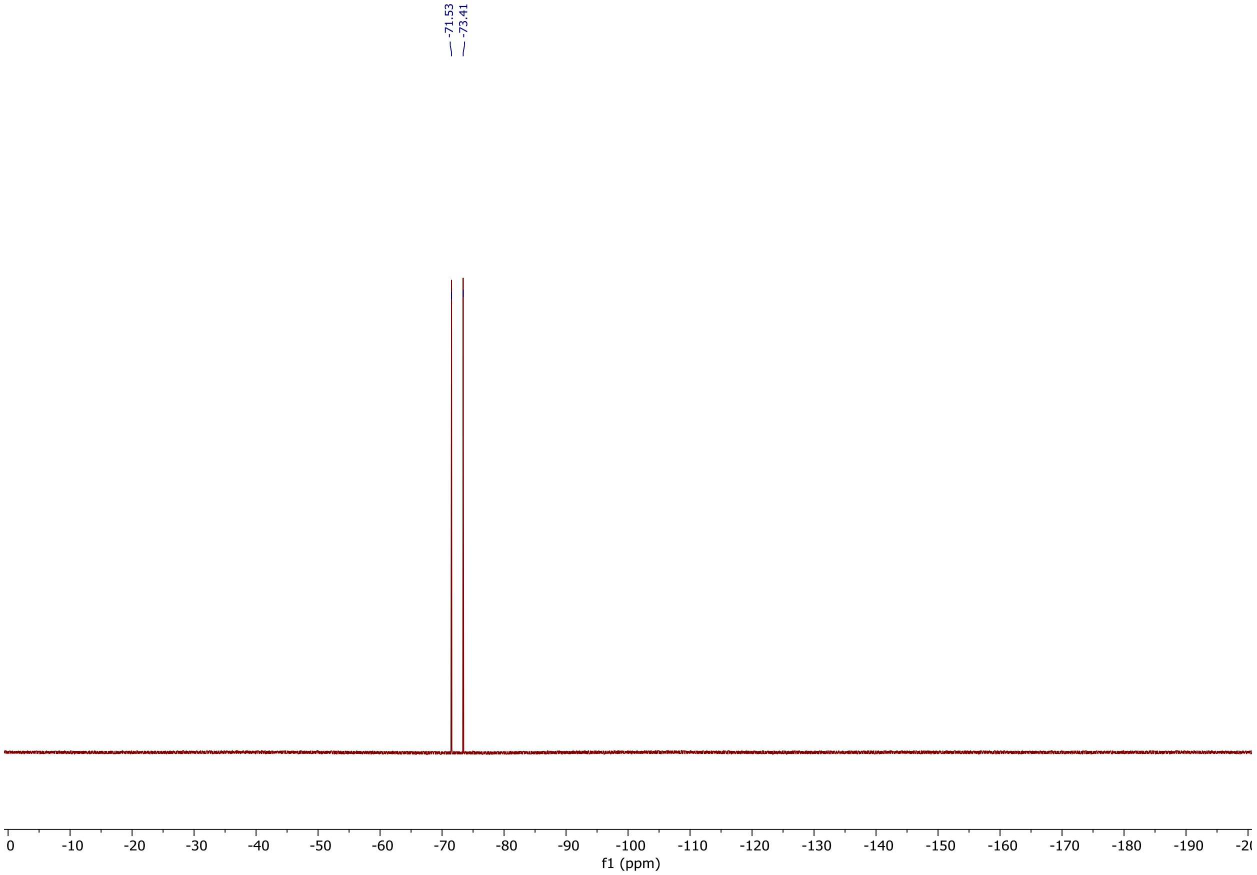


**Figure S46**. Top: $^{1}$H NMR spectrum (400 MHz), and bottom: $^{19}$F NMR spectrum (376 MHz) of **1l** in Acetone-$d_6$.

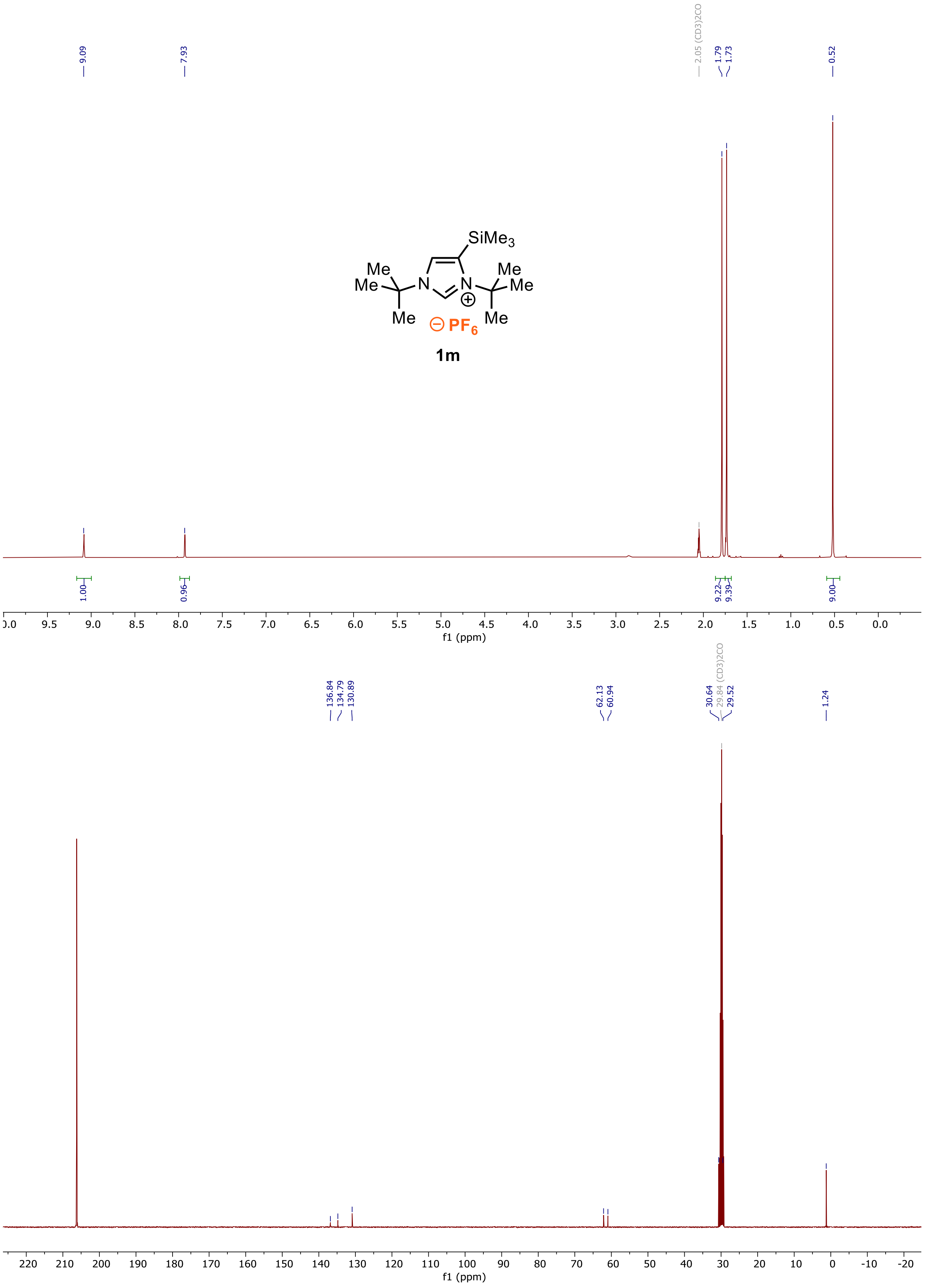


**Figure S47**. Top: $^{1}$H NMR spectrum (400 MHz), and bottom: $^{13}$C NMR spectrum (101 MHz) of **1m** in Acetone-$d_6$.

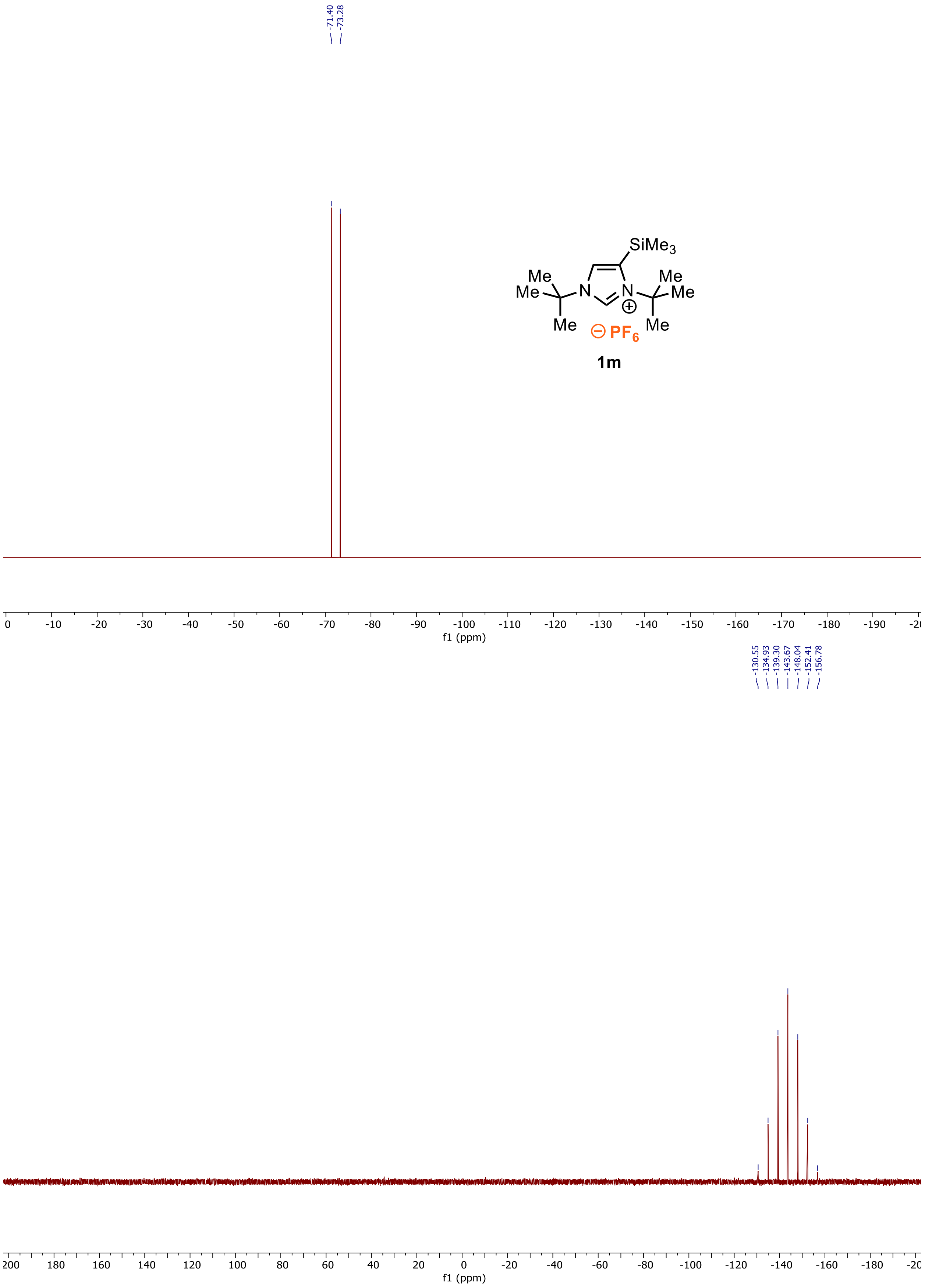


**Figure S48**. Top: $^{19}$F NMR spectrum (376 MHz), and bottom: $^{31}$P NMR spectrum (162 MHz) of **1m** in Acetone-$d_6$.

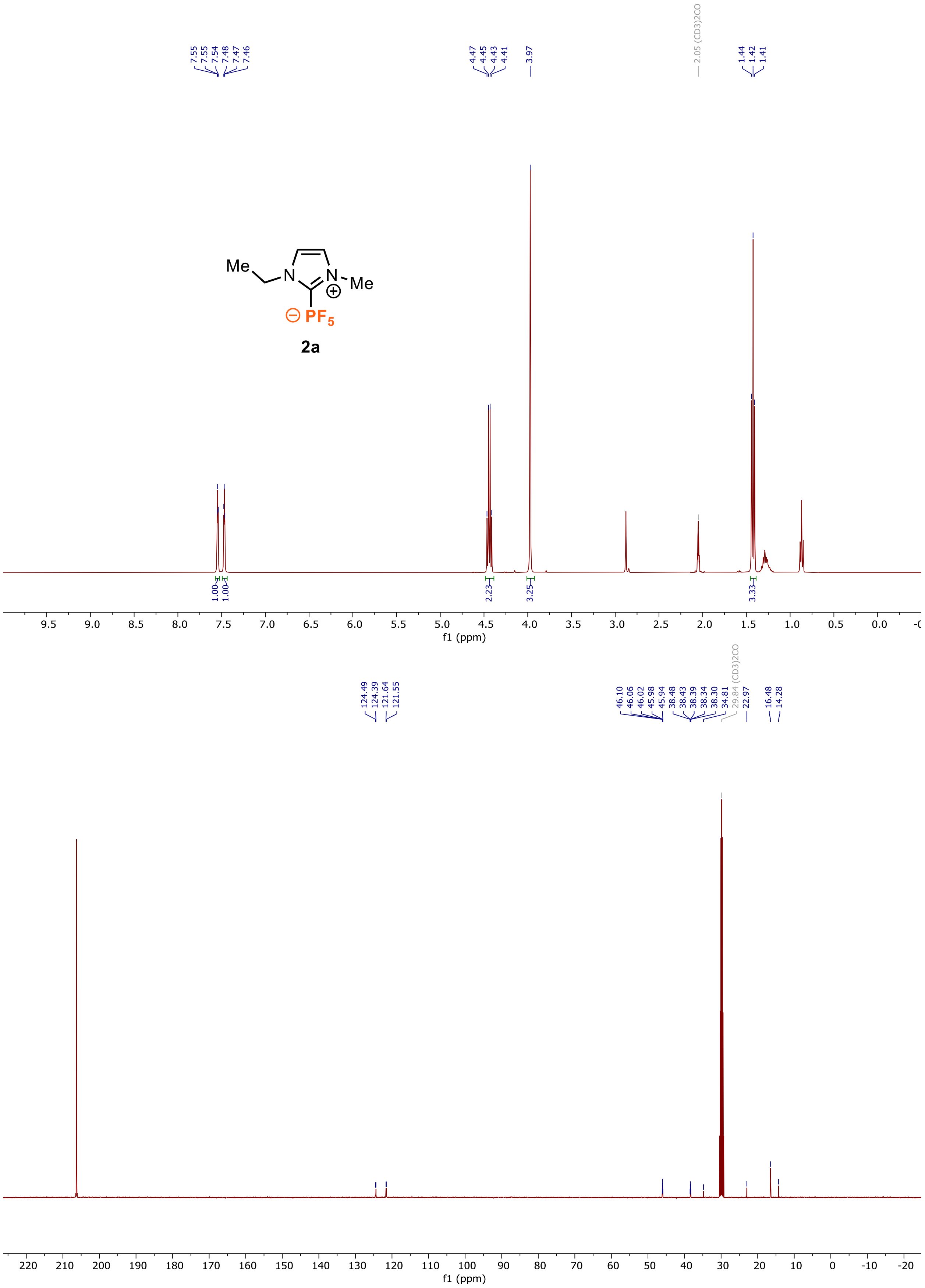


**Figure S49**. Top: $^{1}$H NMR spectrum (400 MHz), and bottom: $^{13}$C NMR spectrum (101 MHz) of **2a** in Acetone-$d_6$.

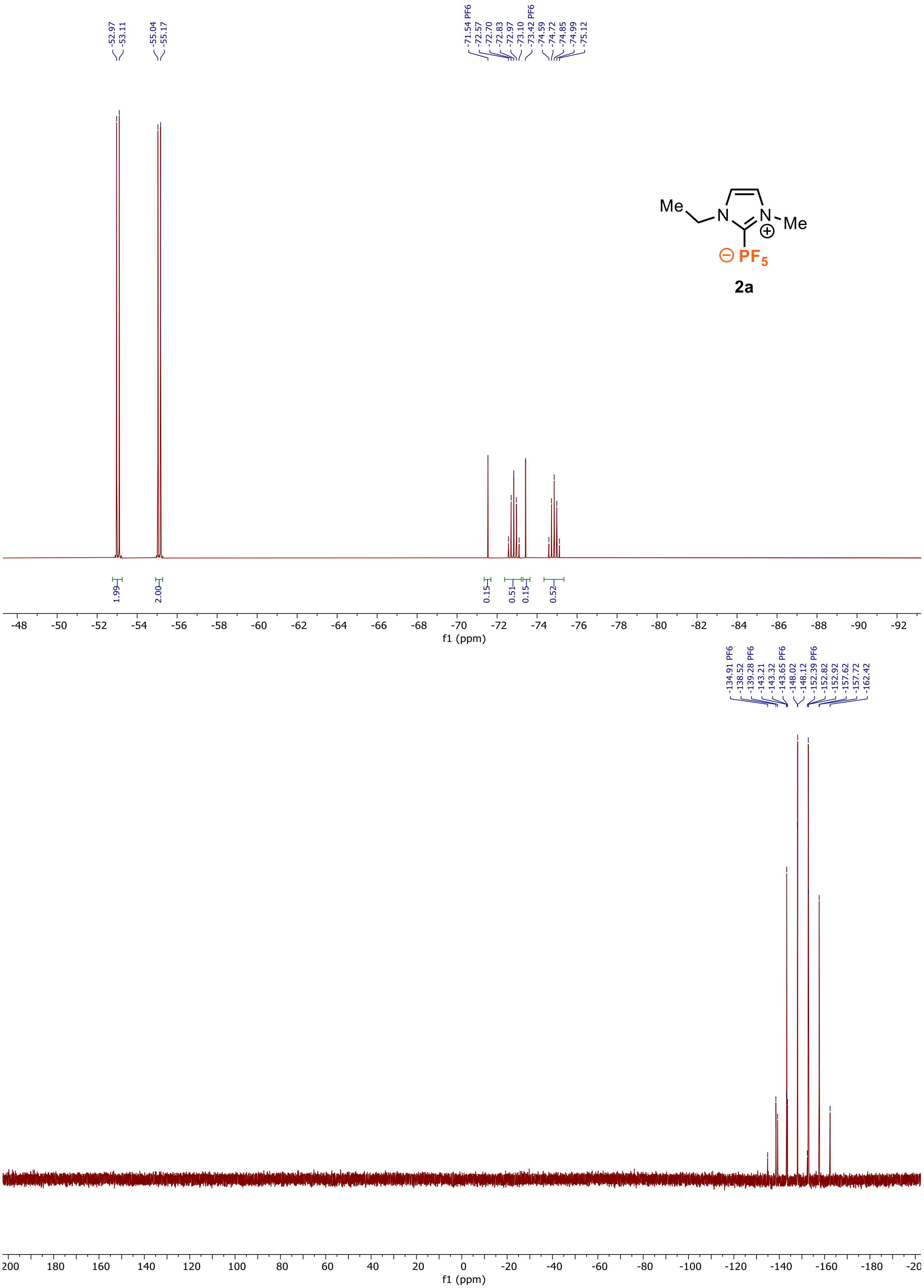


**Figure S50**. Top: $^{19}$F NMR spectrum (376 MHz), and bottom: $^{31}$P NMR spectrum (162 MHz) of **2a** in Acetone-$d_6$.

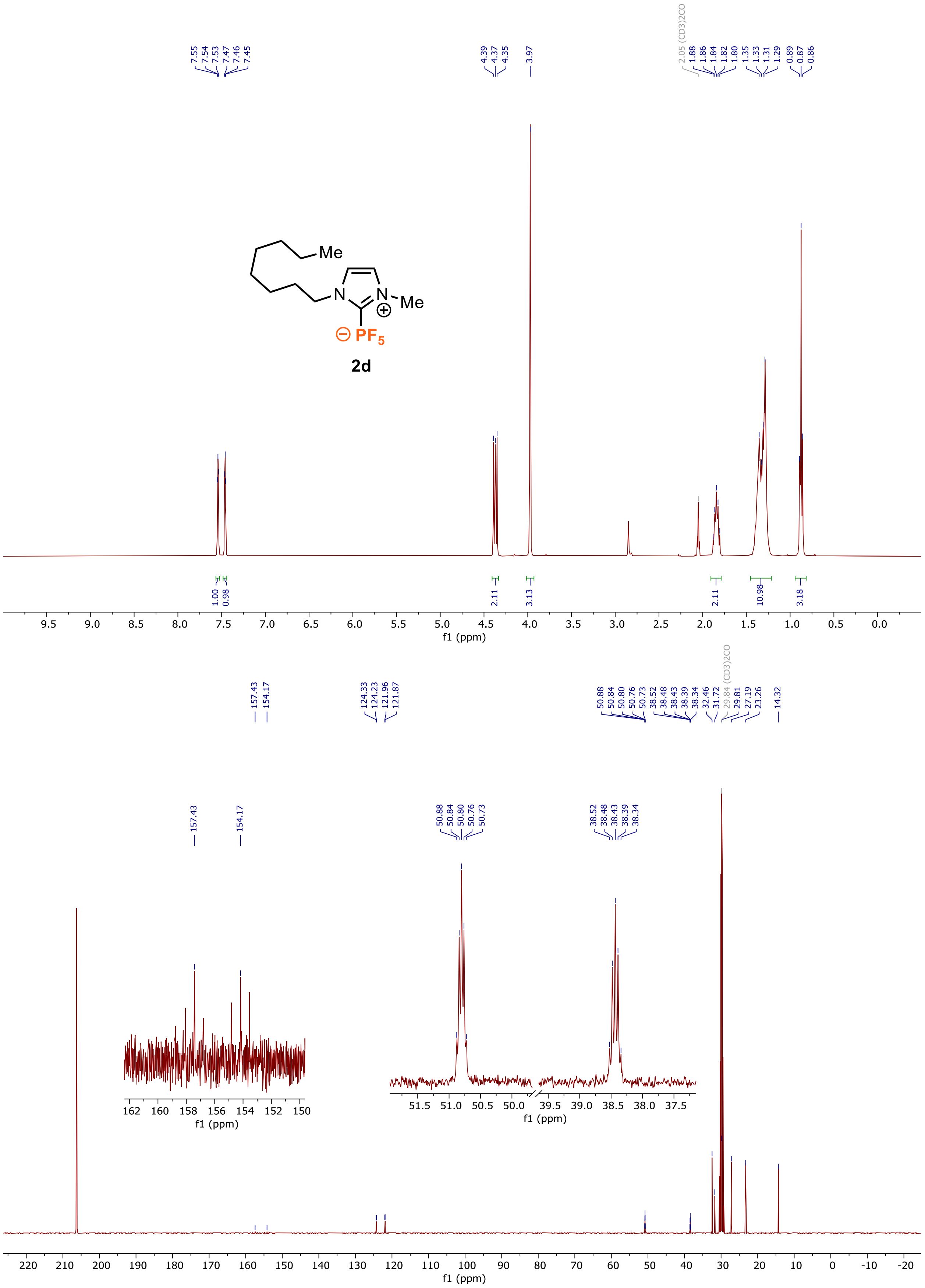


**Figure S51**. Top: $^{1}$H NMR spectrum (400 MHz), and bottom: $^{13}$C NMR spectrum (101 MHz) of **2b** in Acetone-$d_6$.

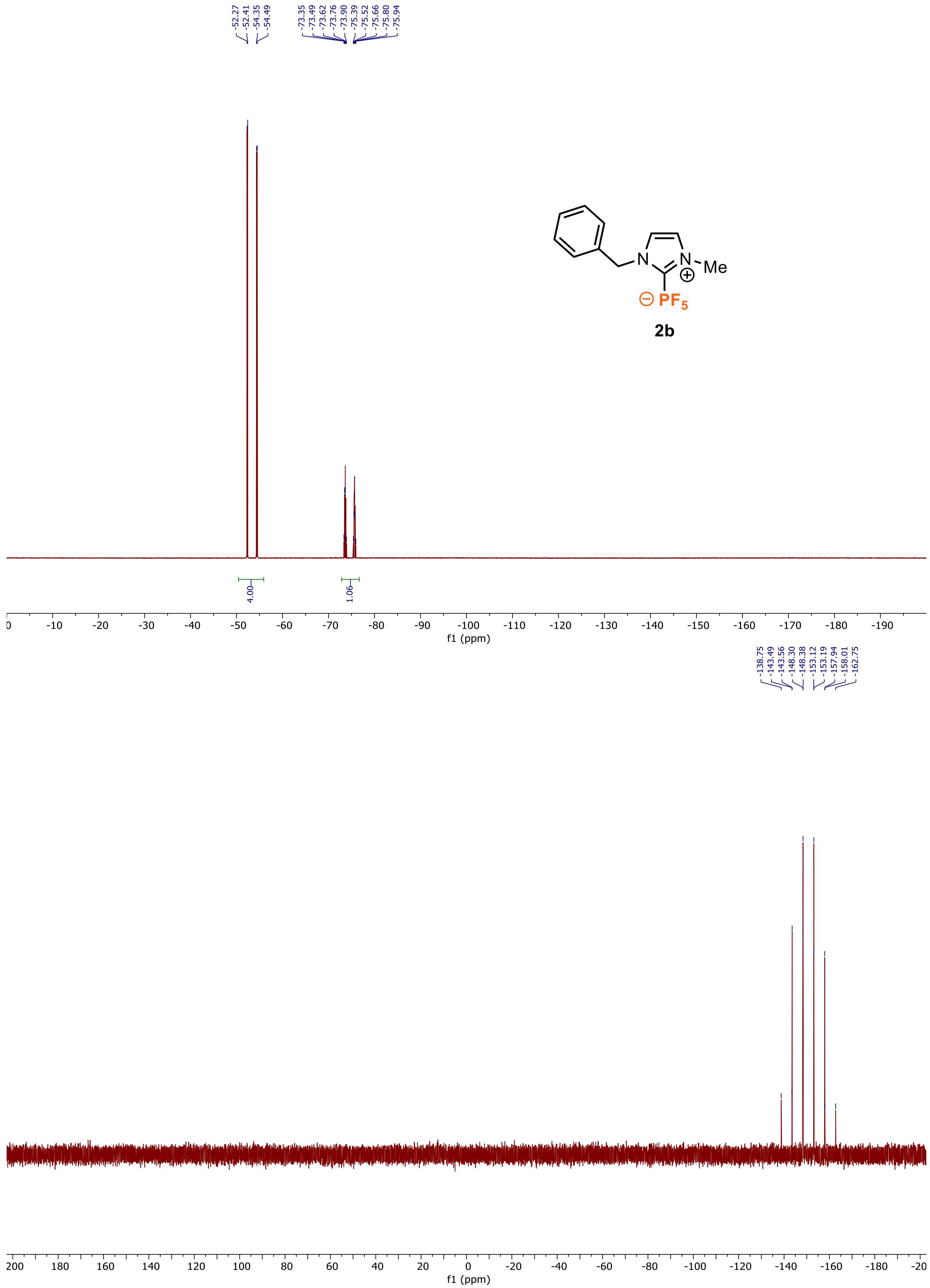


**Figure S52**. Top: $^{19}$F NMR spectrum (376 MHz), and bottom: $^{31}$P NMR spectrum (162 MHz) of **2b** in Acetone-$d_6$.

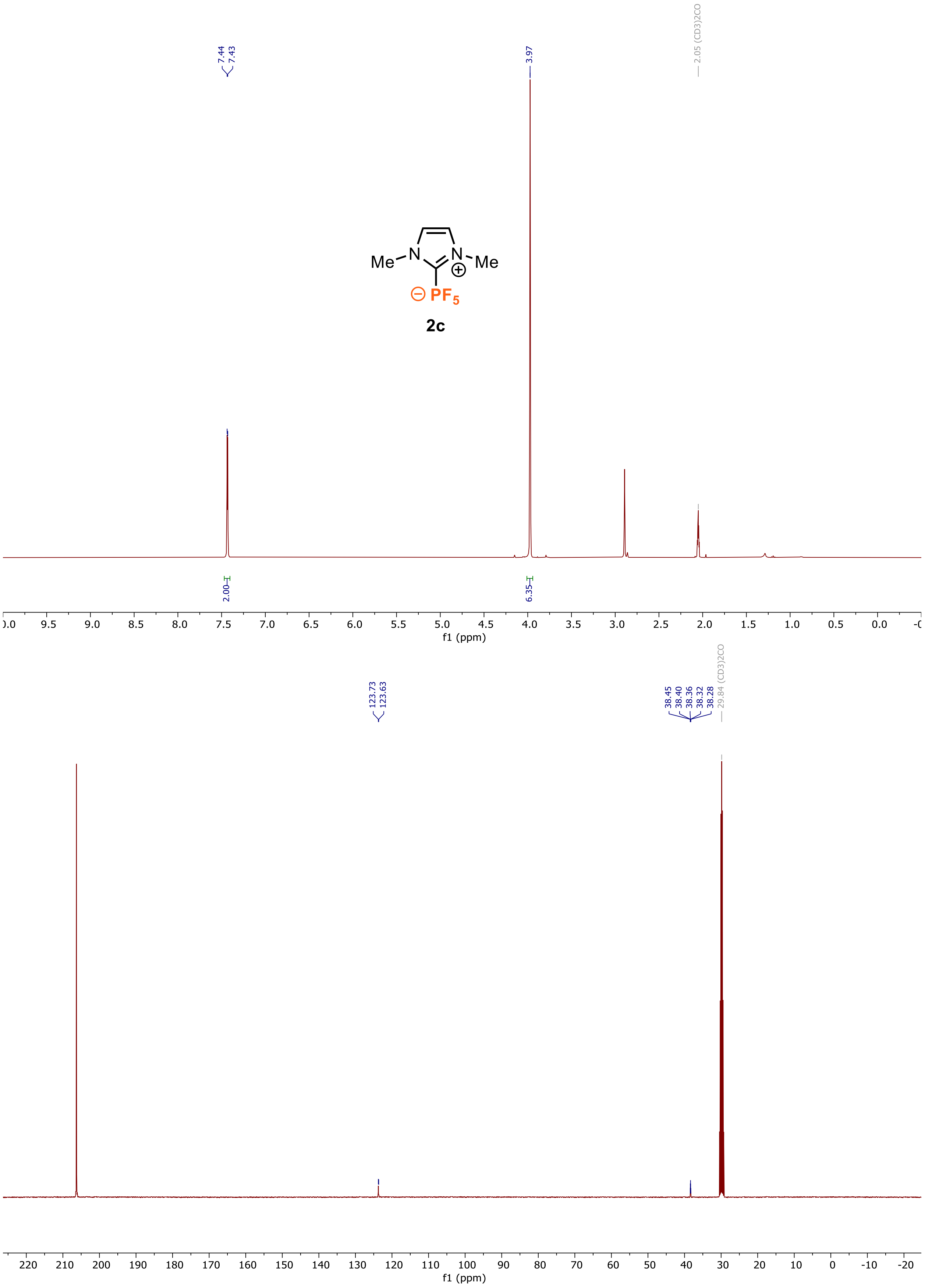


**Figure S53**. Top: $^{1}H$ NMR spectrum (400 MHz), and bottom: $^{13}C$ NMR spectrum (101 MHz) of **2c** in Acetone-$d_6$.

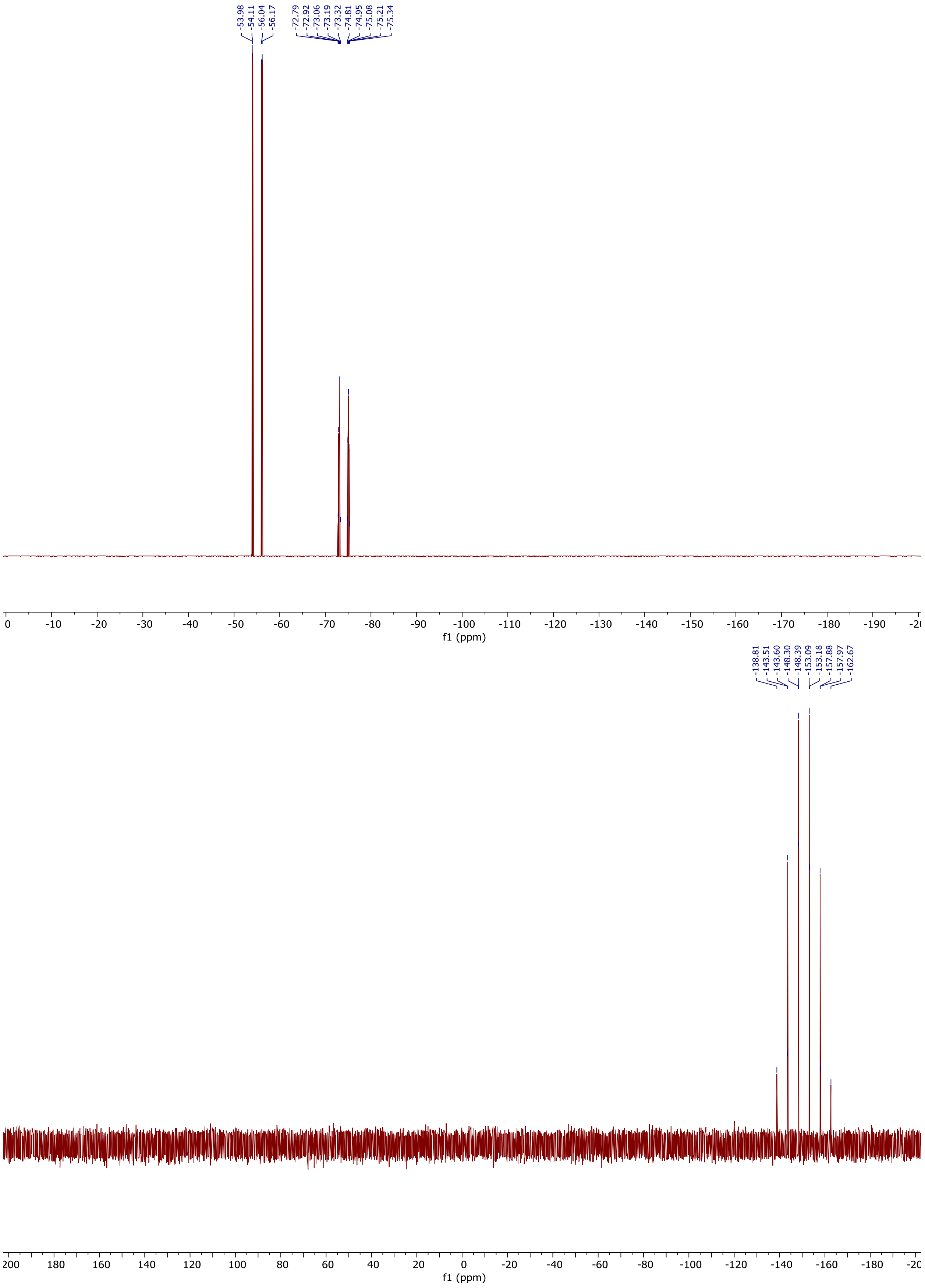


**Figure S54**. Top: $^{19}$F NMR spectrum (376 MHz), and bottom: $^{31}$P NMR spectrum (162 MHz) of **2c** in Acetone-$d_6$.

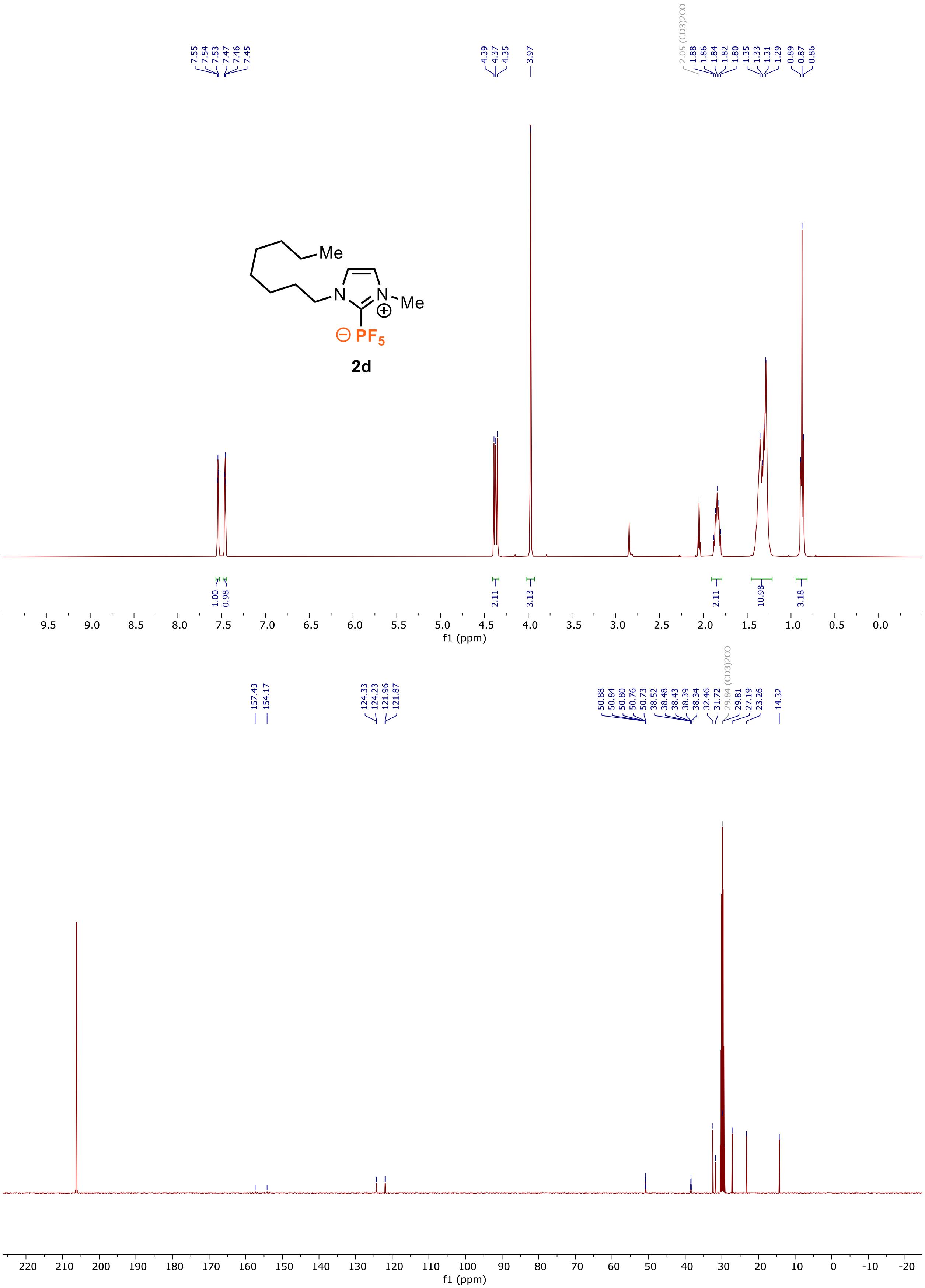


**Figure S55**. Top: $^{1}$H NMR spectrum (400 MHz), and bottom: $^{13}$C NMR spectrum (101 MHz) of **2d** in Acetone-$d_6$.

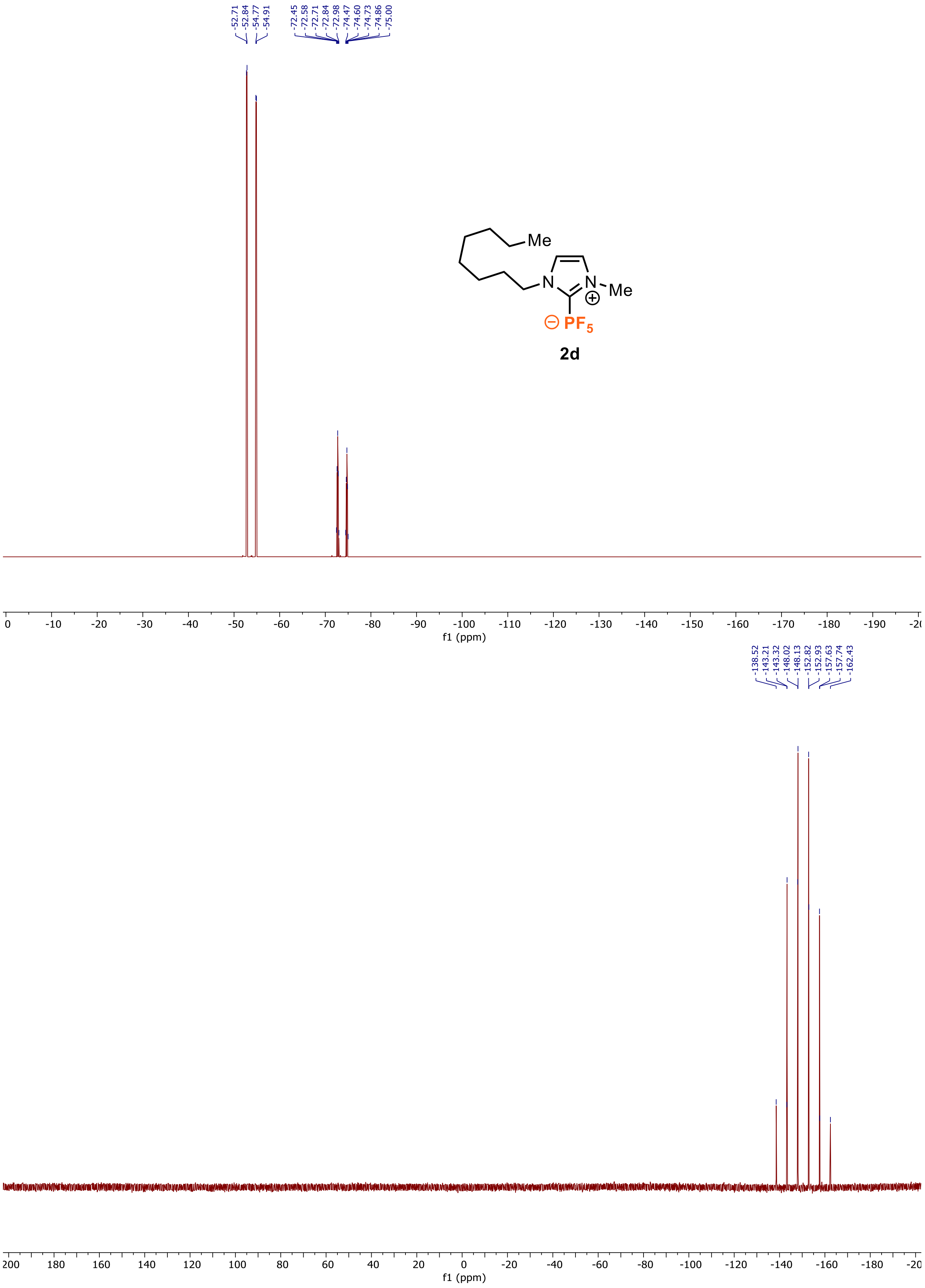


**Figure S56**. Top: $^{19}F$ NMR spectrum (376 MHz), and bottom: $^{31}P$ NMR spectrum (162 MHz) of **2d** in Acetone-$d_6$.

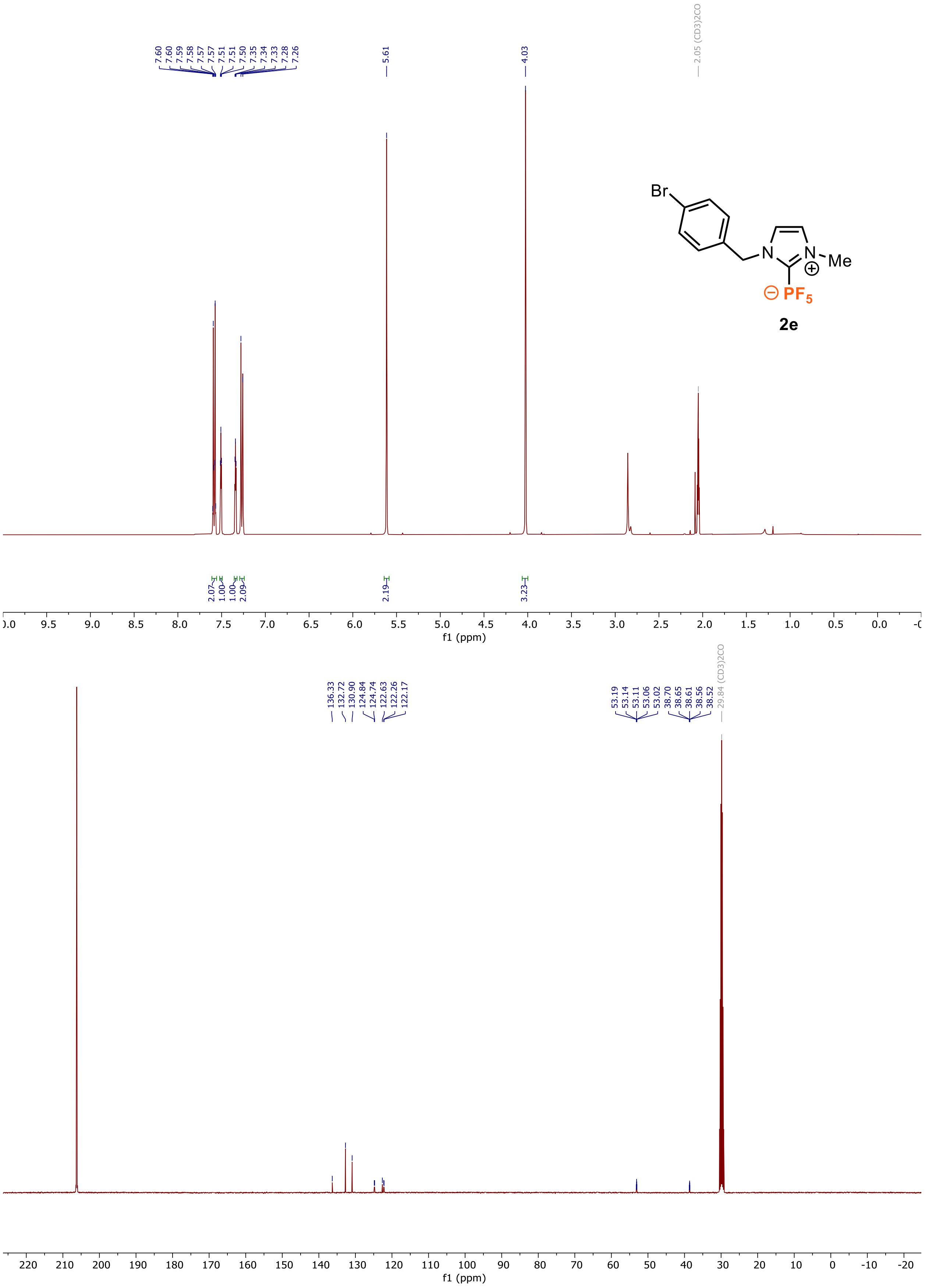


**Figure S57**. Top: $^{1}$H NMR spectrum (400 MHz), and bottom: $^{13}$C NMR spectrum (101 MHz) of **2e** in Acetone-$d_6$.

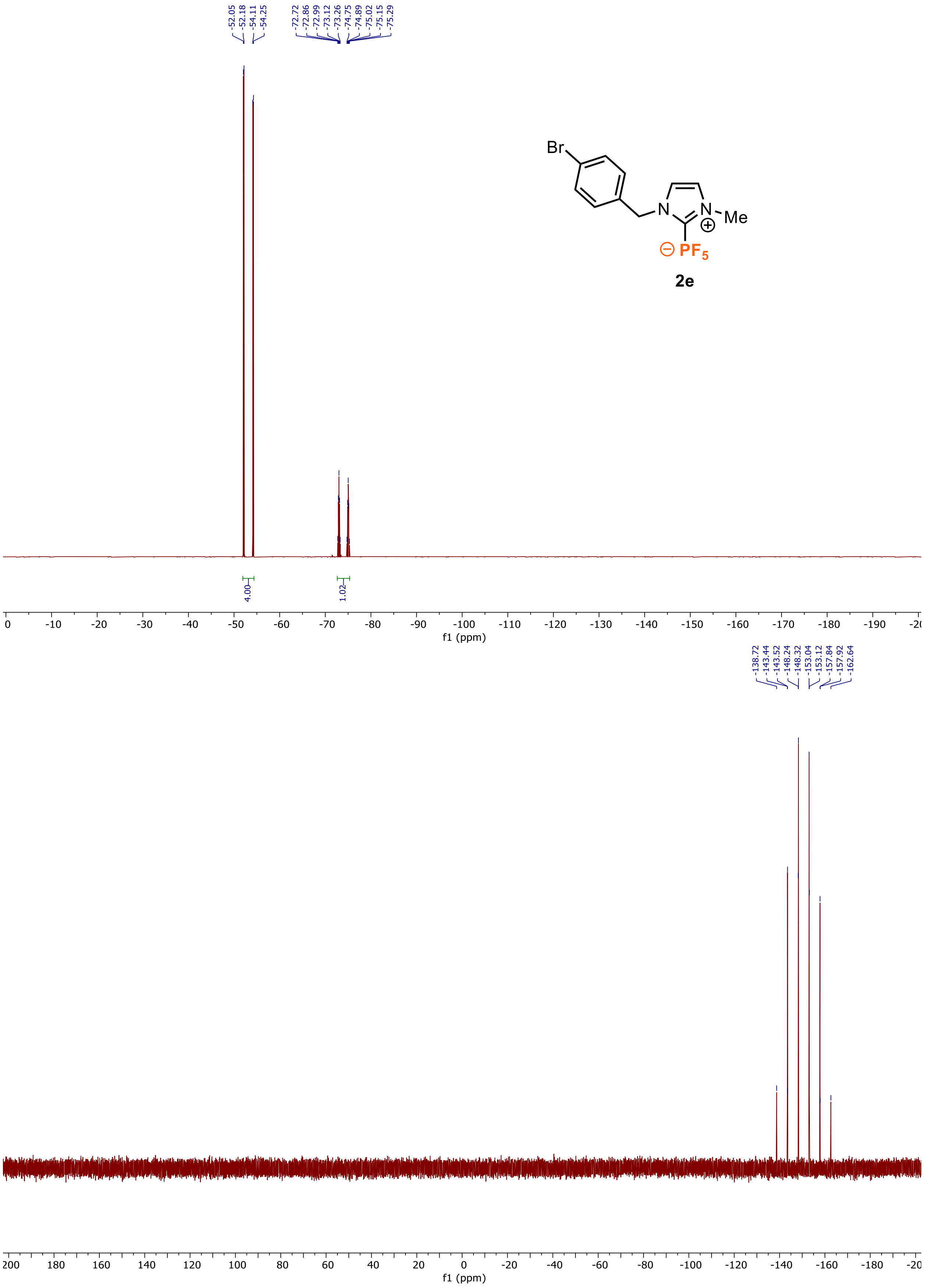


**Figure S58**. Top: $^{19}$F NMR spectrum (376 MHz), and bottom: $^{31}$P NMR spectrum (162 MHz) of **2e** in Acetone-$d_6$.

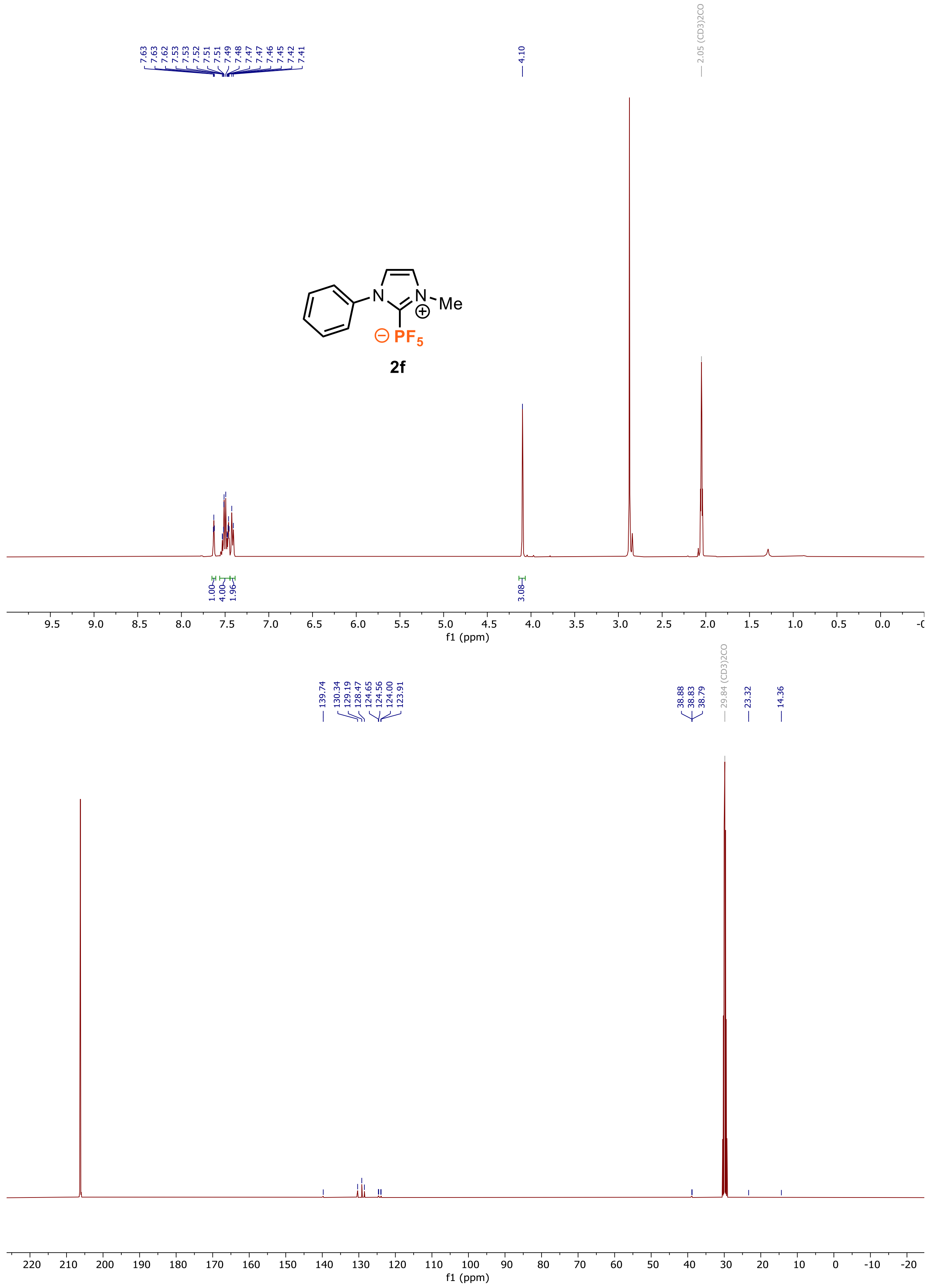


**Figure S59**. Top: $^{1}$H NMR spectrum (400 MHz), and bottom: $^{13}$C NMR spectrum (101 MHz) of **2f** in Acetone-$d_6$.

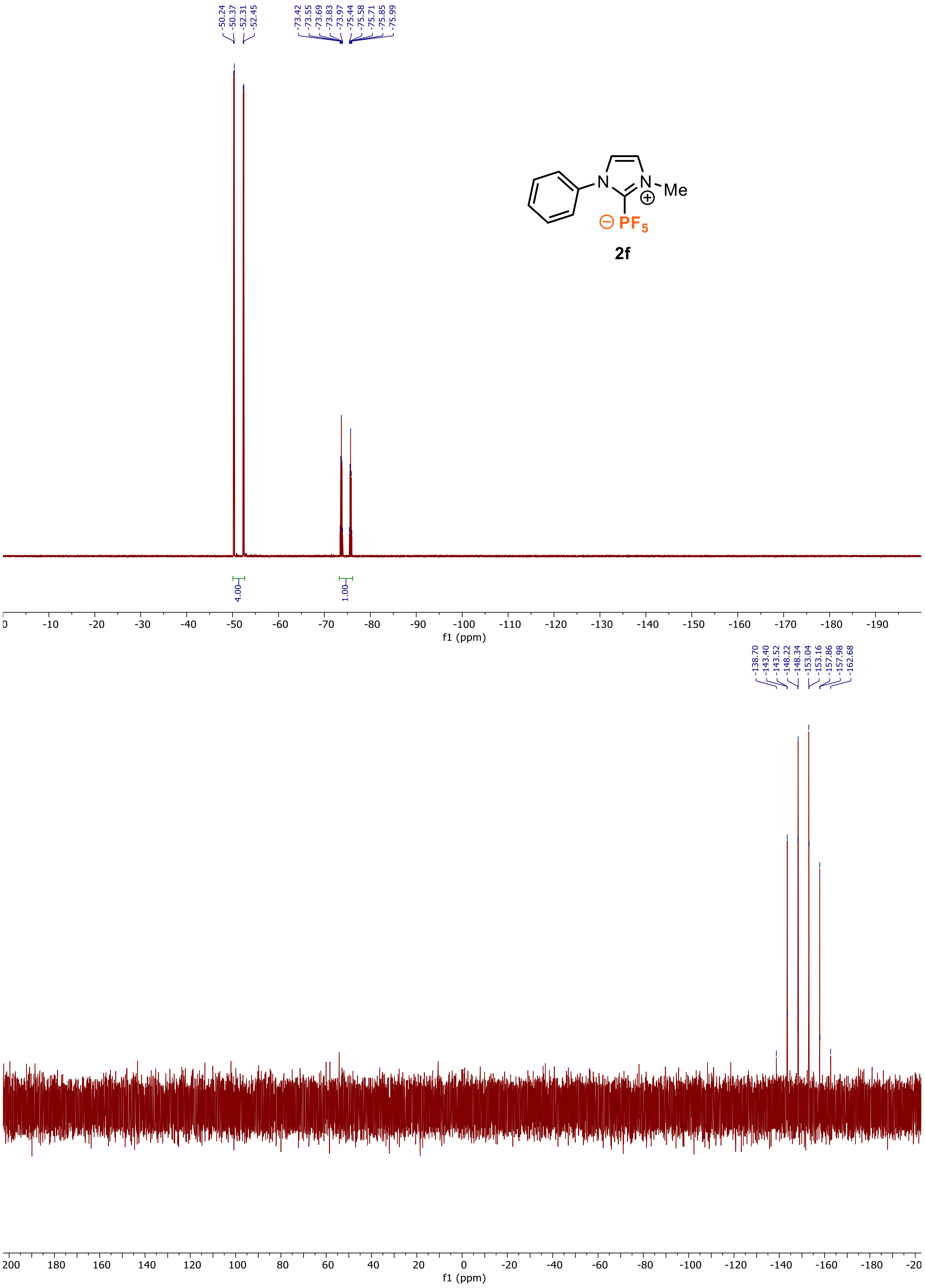


**Figure S60**. Top: $^{19}$F NMR spectrum (376 MHz), and bottom: $^{31}$P NMR spectrum (162 MHz) of **2f** in Acetone-$d_6$.

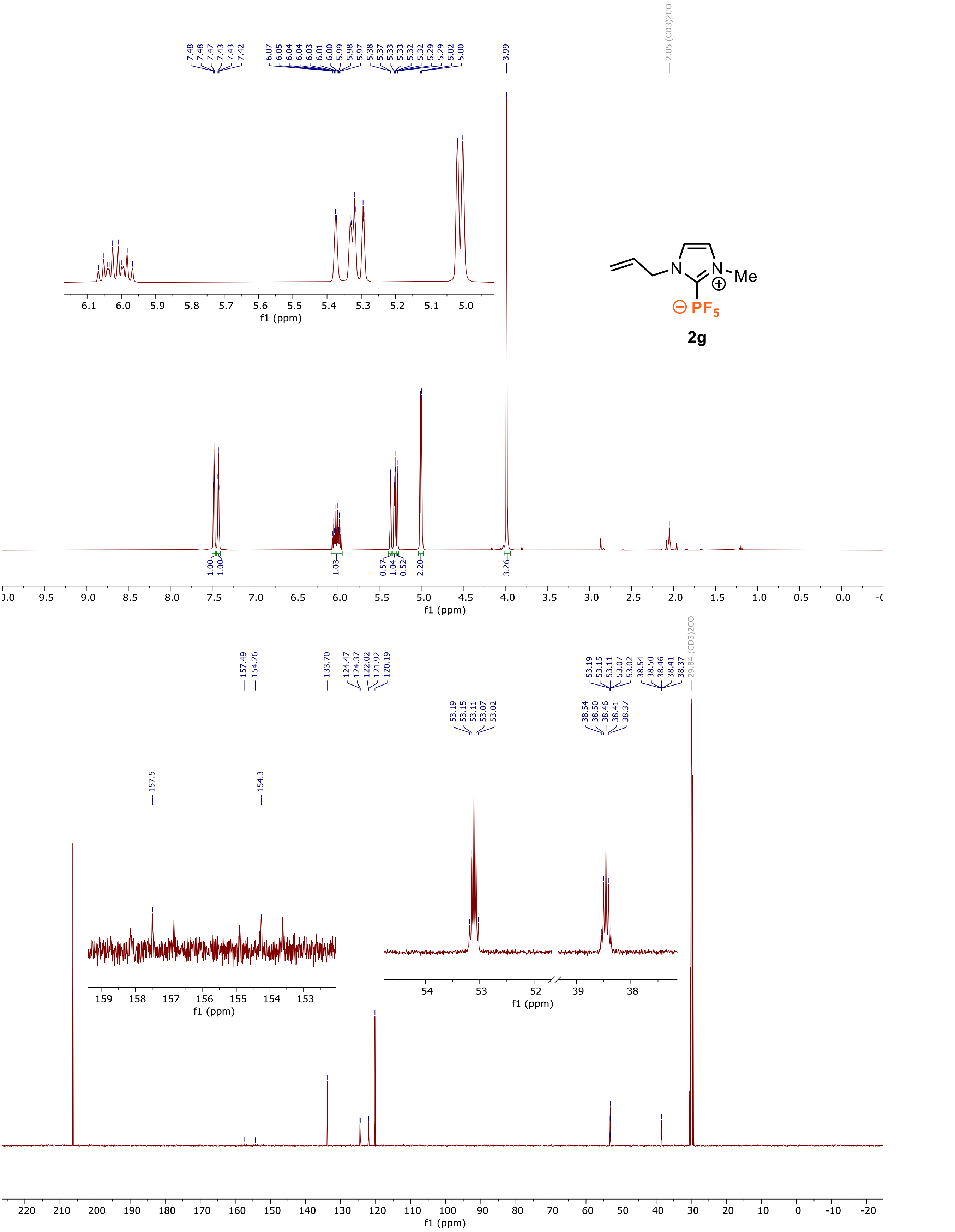


**Figure S61**. Top: $^{1}H$ NMR spectrum (400 MHz), and bottom: $^{13}C$ NMR spectrum (101 MHz) of **2g** in Acetone-$d_6$.

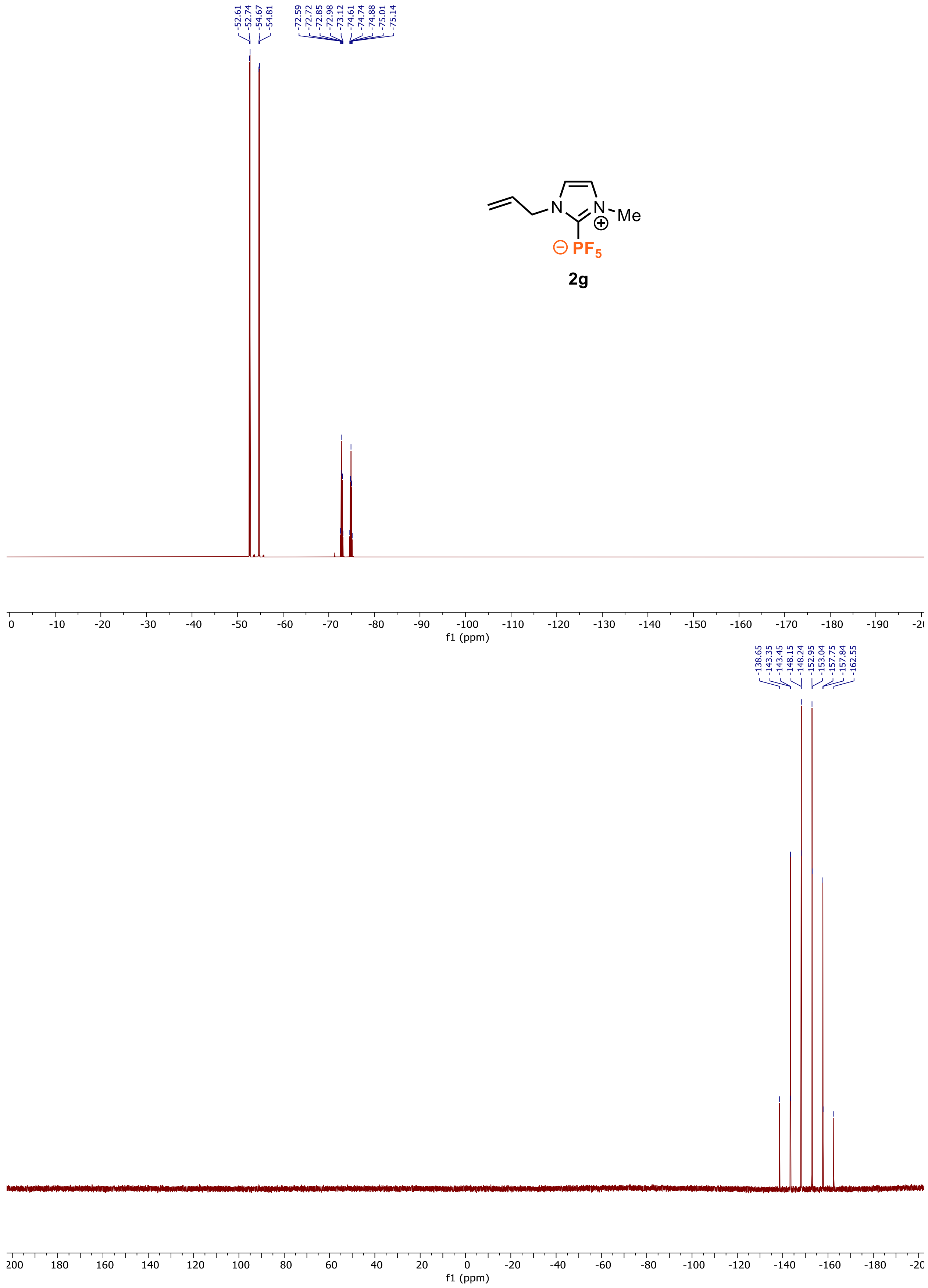


**Figure S62**. Top: $^{19}$F NMR spectrum (376 MHz), and bottom: $^{31}$P NMR spectrum (162 MHz) of **2g** in Acetone-$d_6$.

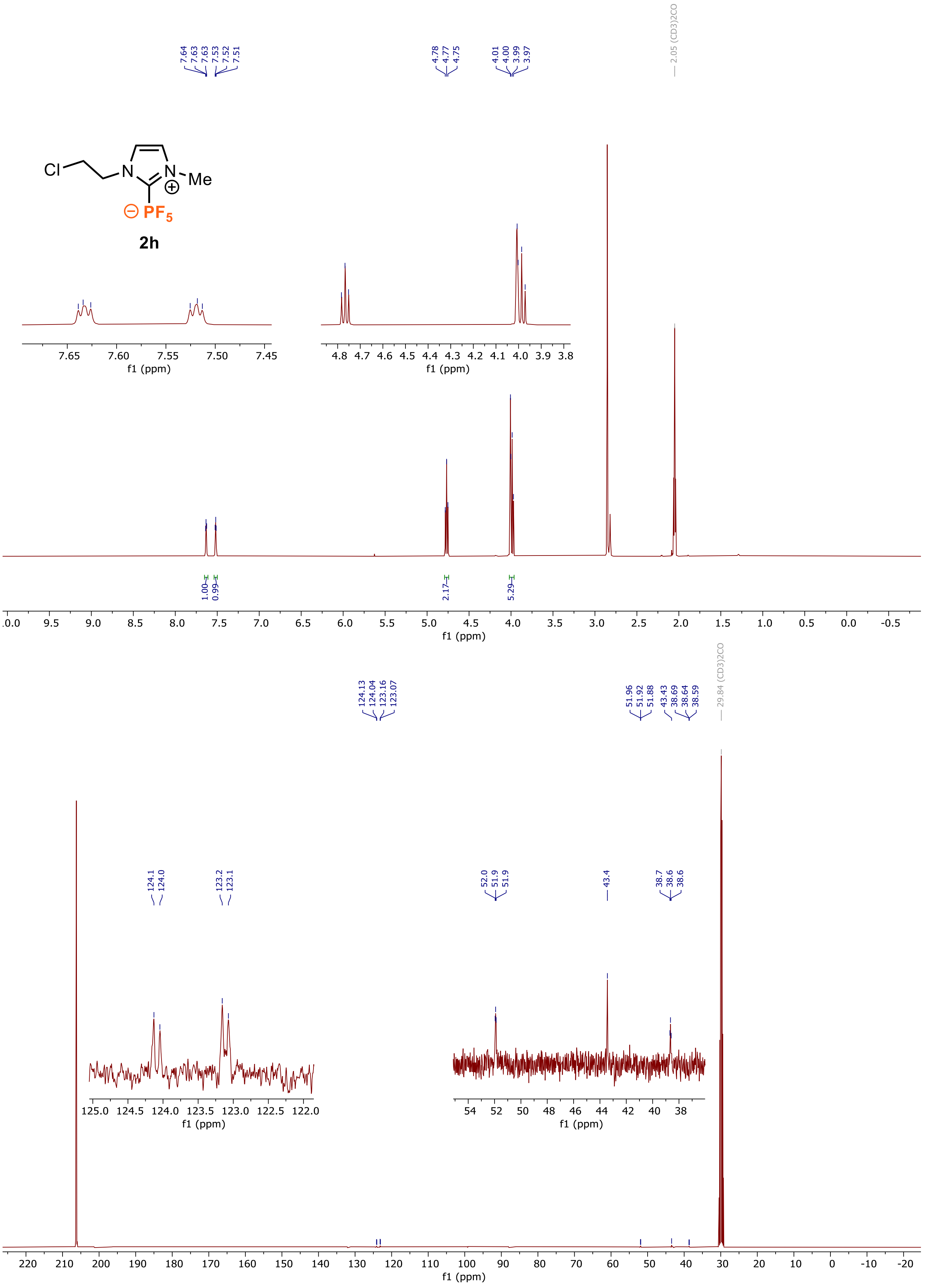


**Figure S63**. Top: $^{1}$H NMR spectrum (400 MHz), and bottom: $^{13}$C NMR spectrum (101 MHz) of **2h** in Acetone-$d_6$.

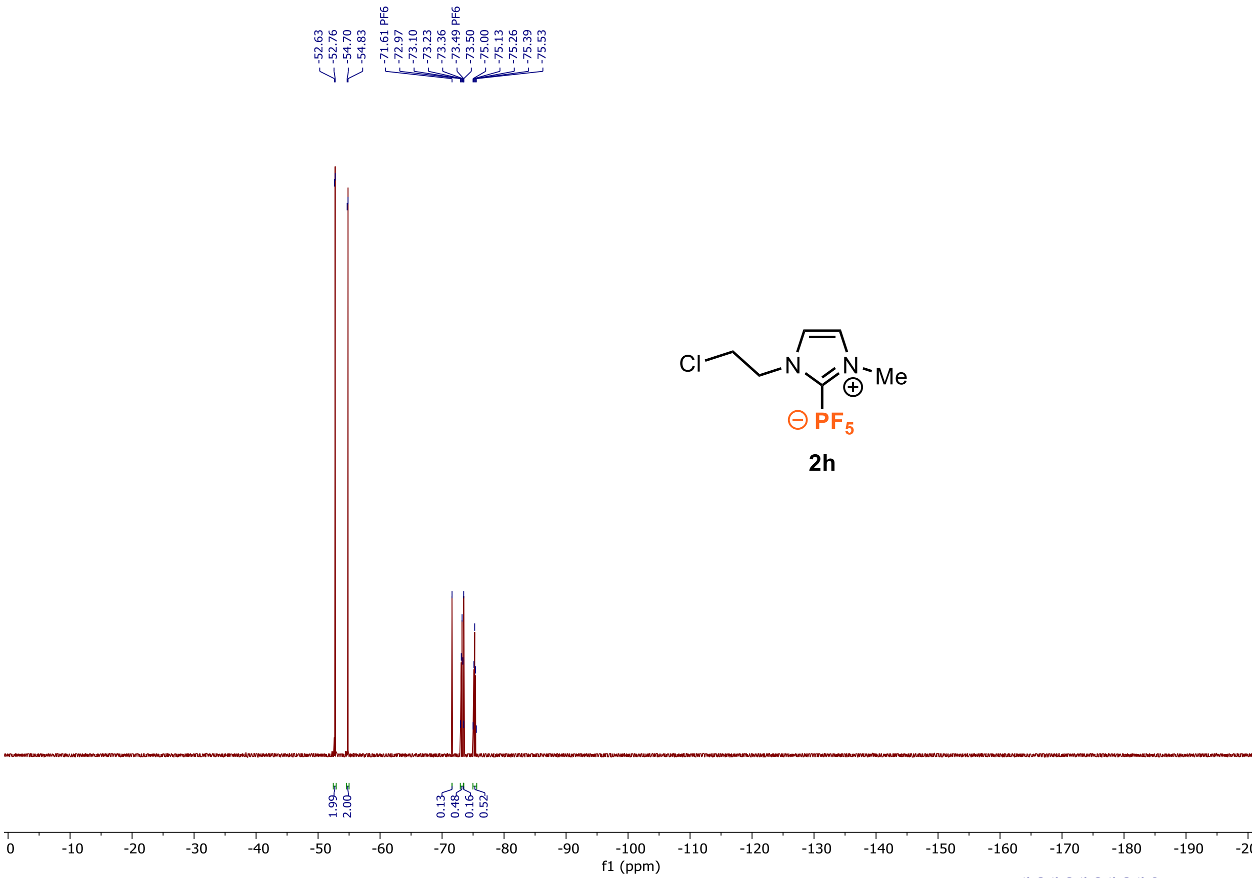

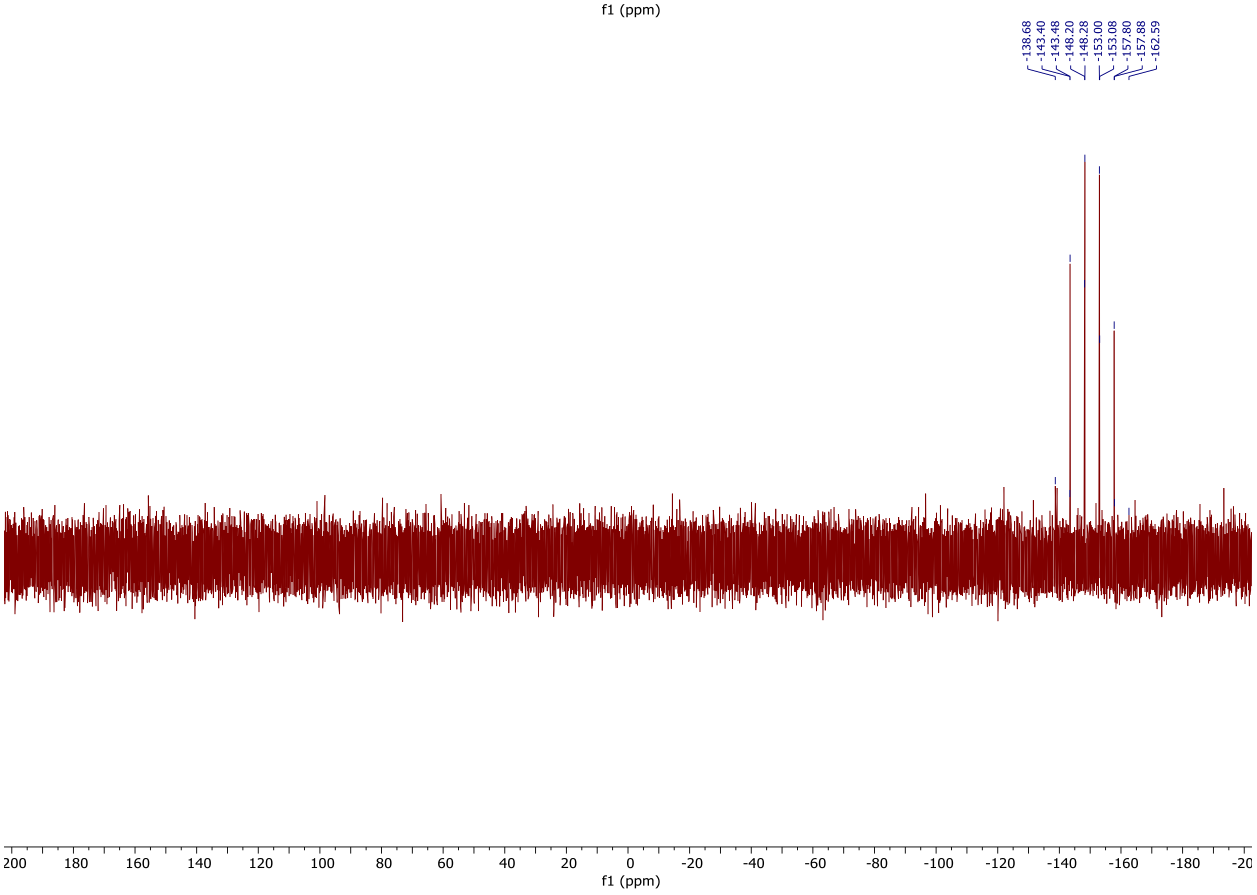

**Figure S64**. Top: $^{19}$F NMR spectrum (376 MHz), and bottom: $^{31}$P NMR spectrum (162 MHz) of **2h** in Acetone-$d_6$.

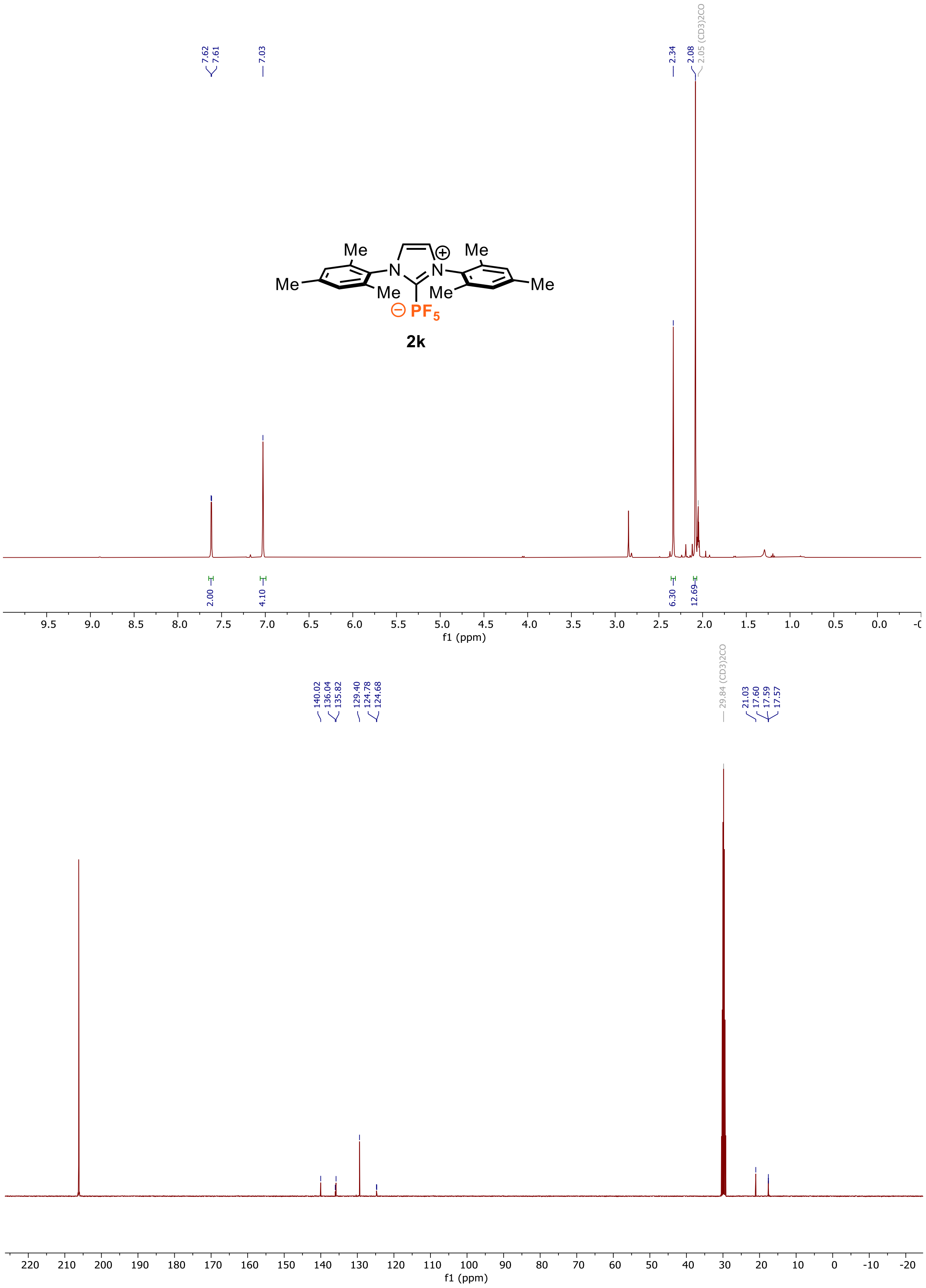


**Figure S65**. Top: $^{1}$H NMR spectrum (400 MHz), and bottom: $^{13}$C NMR spectrum (101 MHz) of **2k** in Acetone-$d_6$.

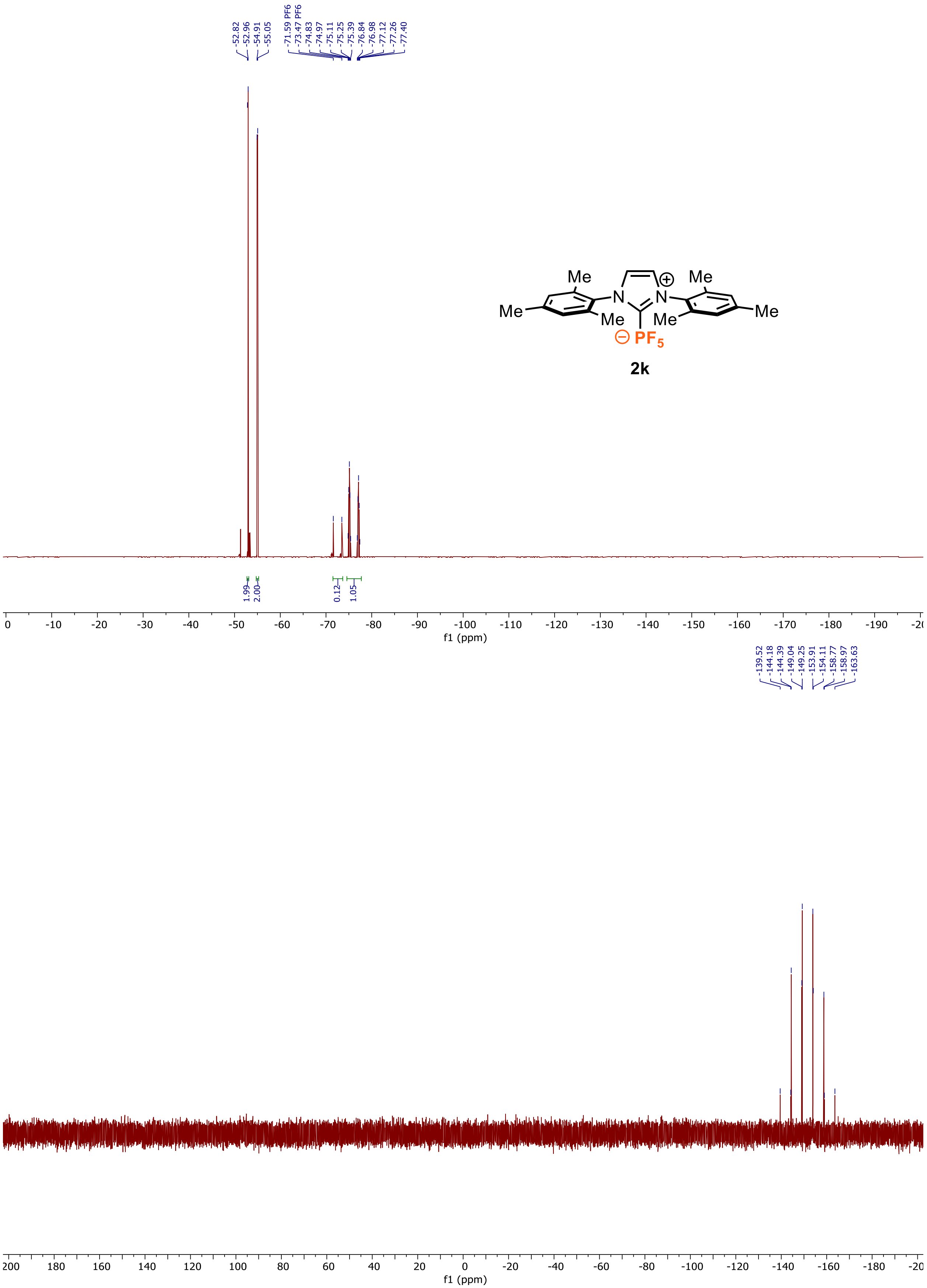


**Figure S66**. Top: $^{19}$F NMR spectrum (376 MHz), and bottom: $^{31}$P NMR spectrum (162 MHz) of **2k** in Acetone-$d_6$.

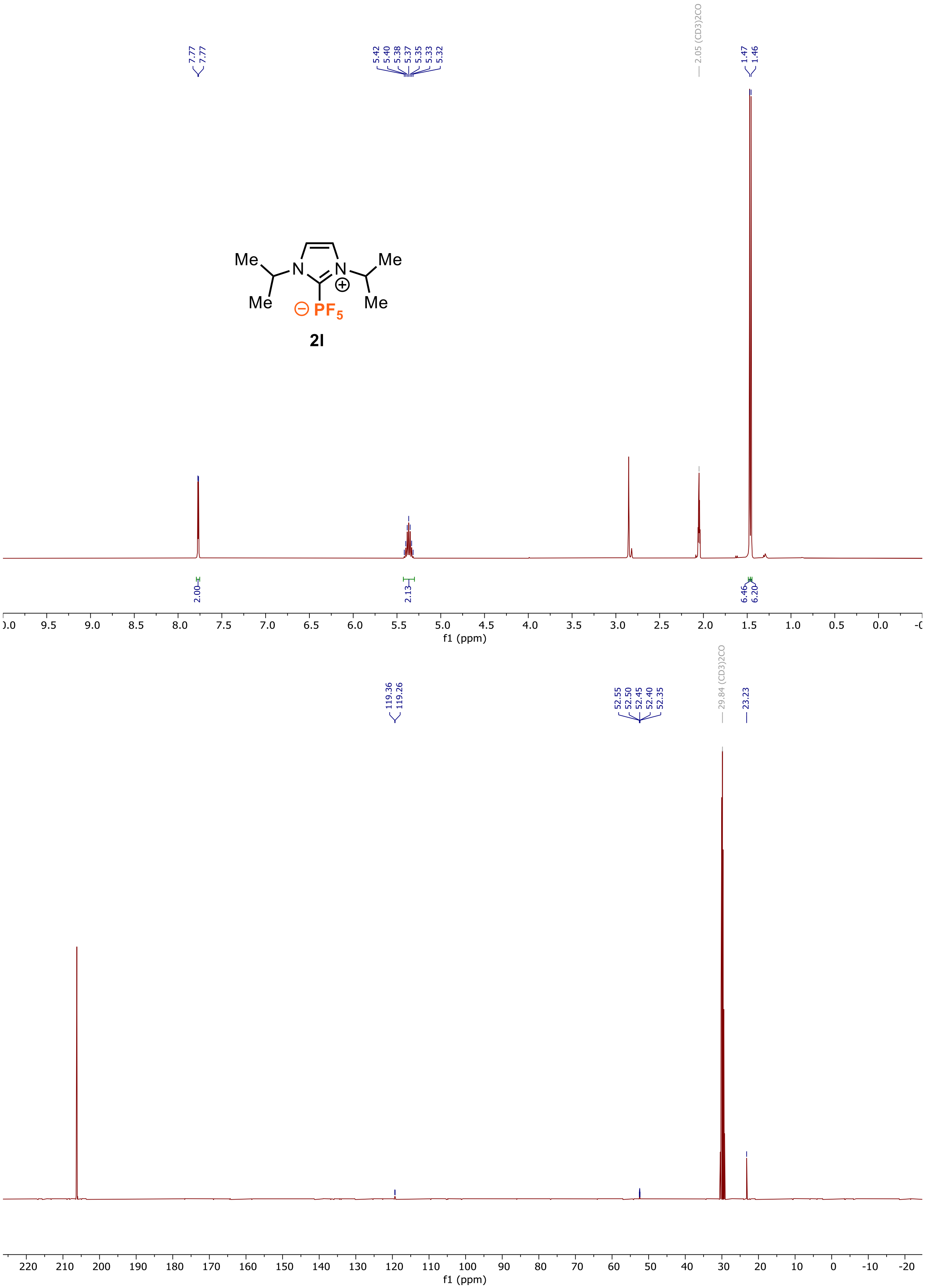


**Figure S67**. Top: $^{1}H$ NMR spectrum (400 MHz), and bottom: $^{13}C$ NMR spectrum (101 MHz) of **2l** in Acetone-$d_6$.

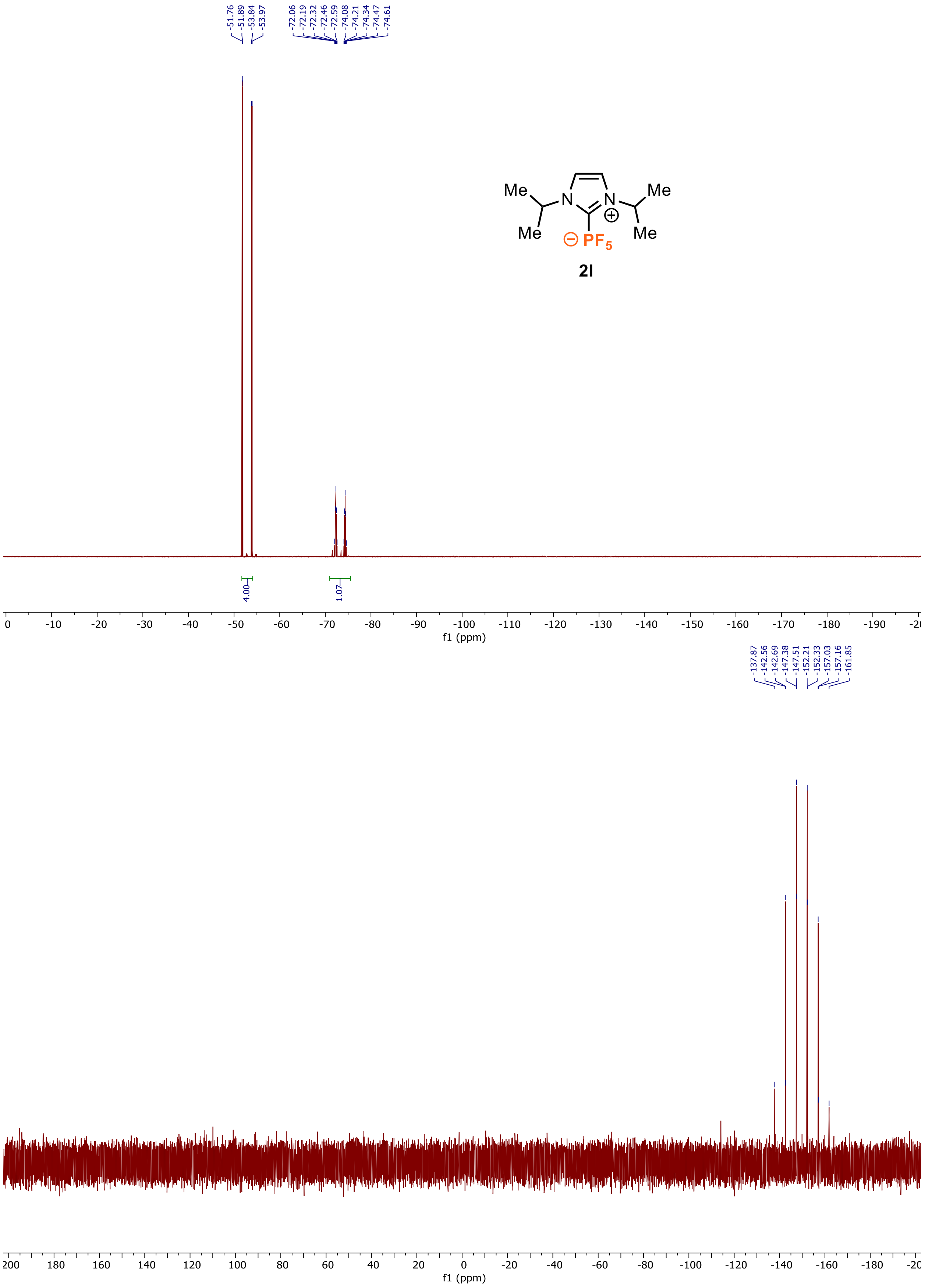


**Figure S68**. Top: $^{19}$F NMR spectrum (376 MHz), and bottom: $^{31}$P NMR spectrum (162 MHz) of **2l** in Acetone-$d_6$.

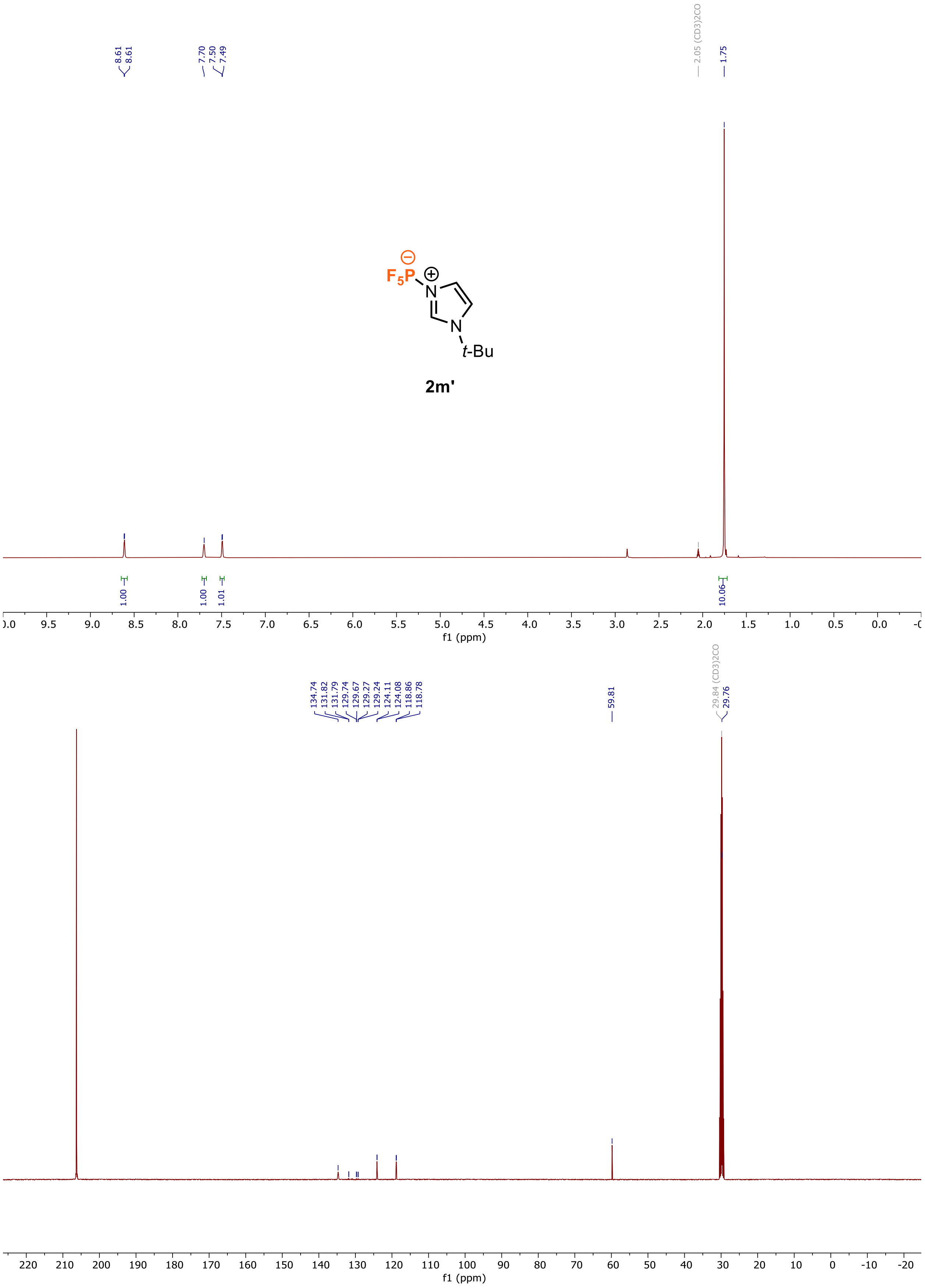


**Figure S69**. Top: $^{1}H$ NMR spectrum (400 MHz), and bottom: $^{13}C$ NMR spectrum (101 MHz) of **2m** in Acetone-$d_6$.

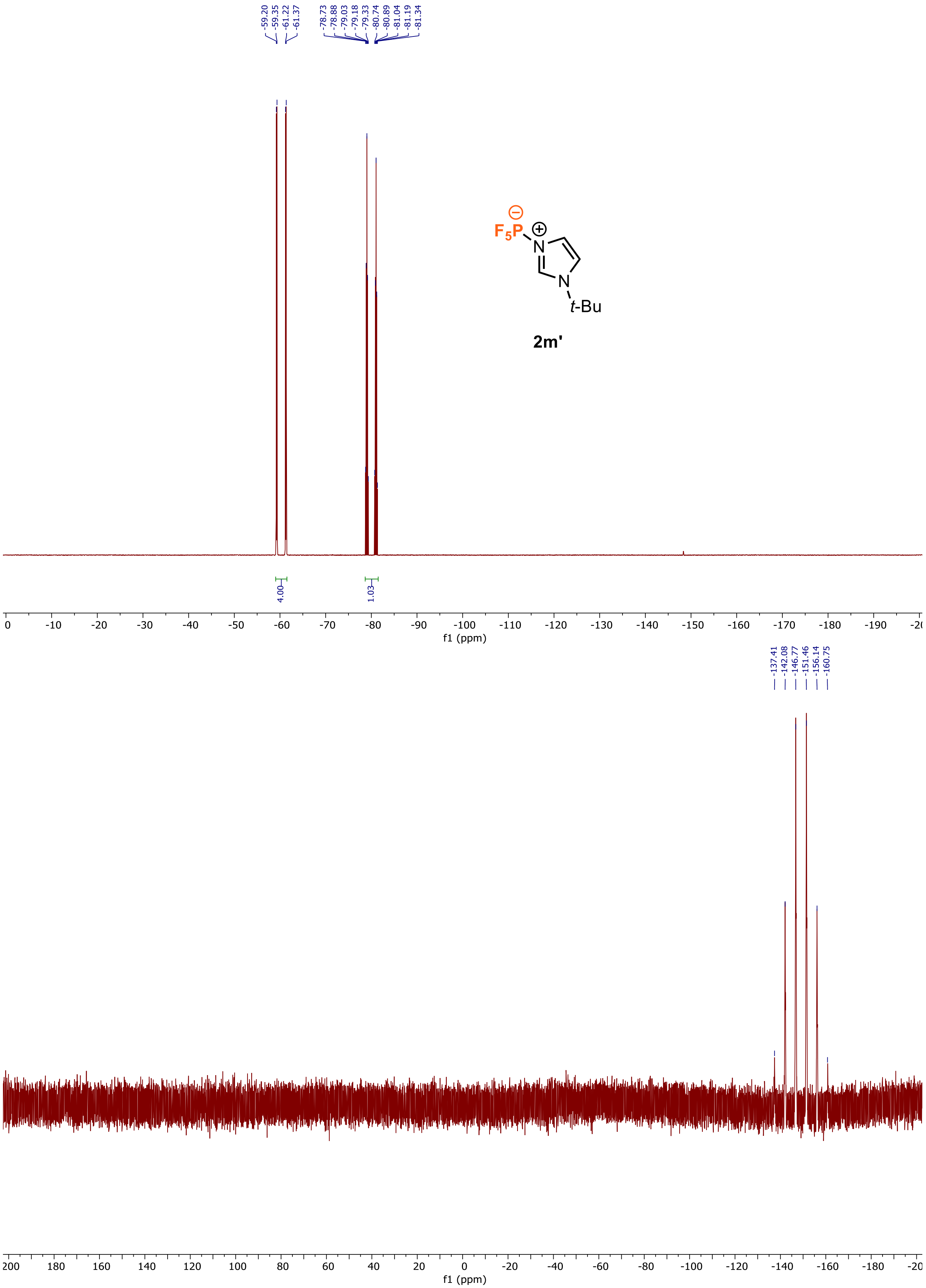


**Figure S70**. Top: $^{19}F$ NMR spectrum (376 MHz), and bottom: $^{31}P$ NMR spectrum (162 MHz) of **2m** in Acetone-$d_6$.

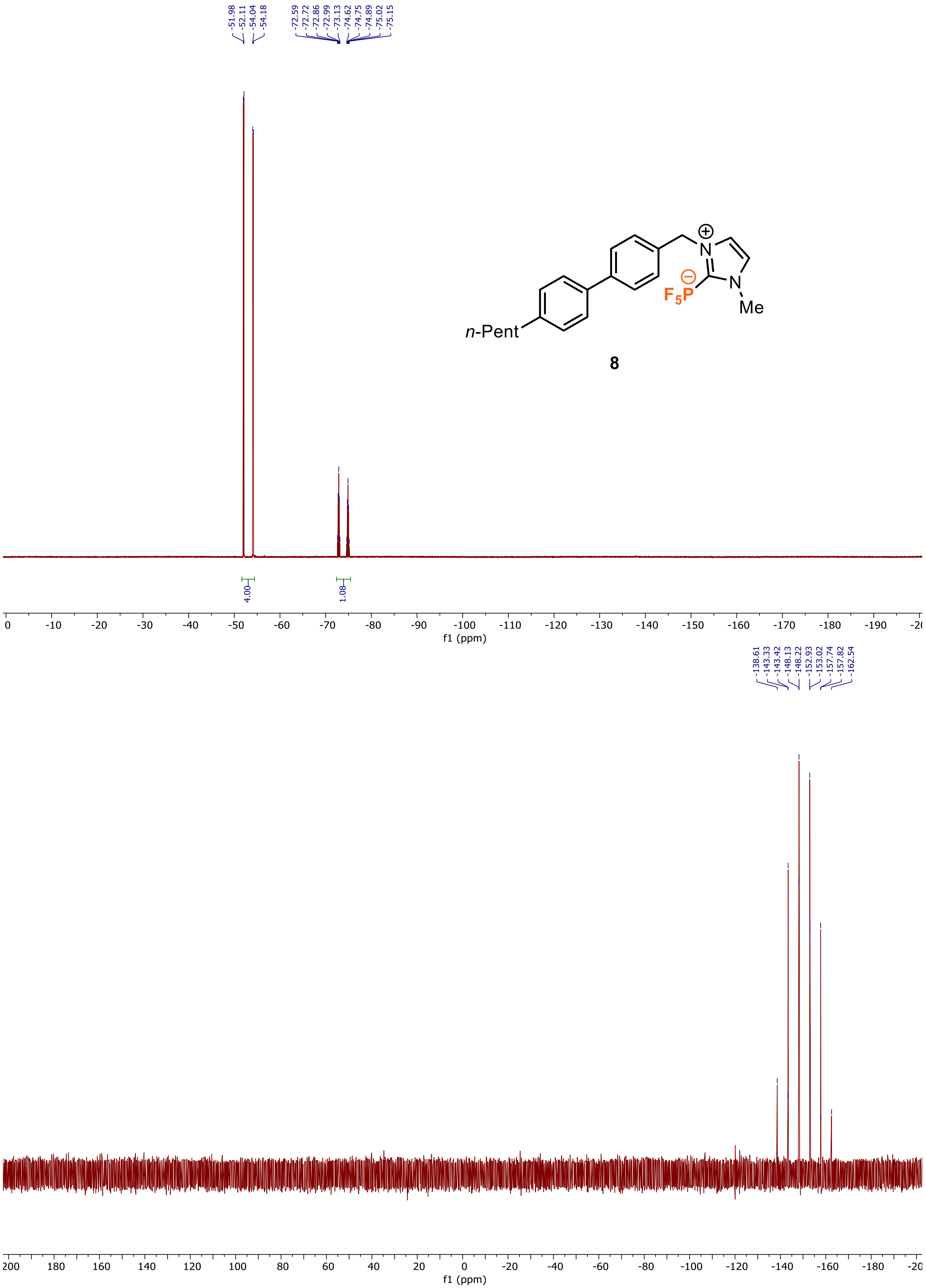


**Figure S71**. Top: $^{1}H$ NMR spectrum (400 MHz), and bottom: $^{13}C$ NMR spectrum (101 MHz) of **8** in Acetone-$d_6$.

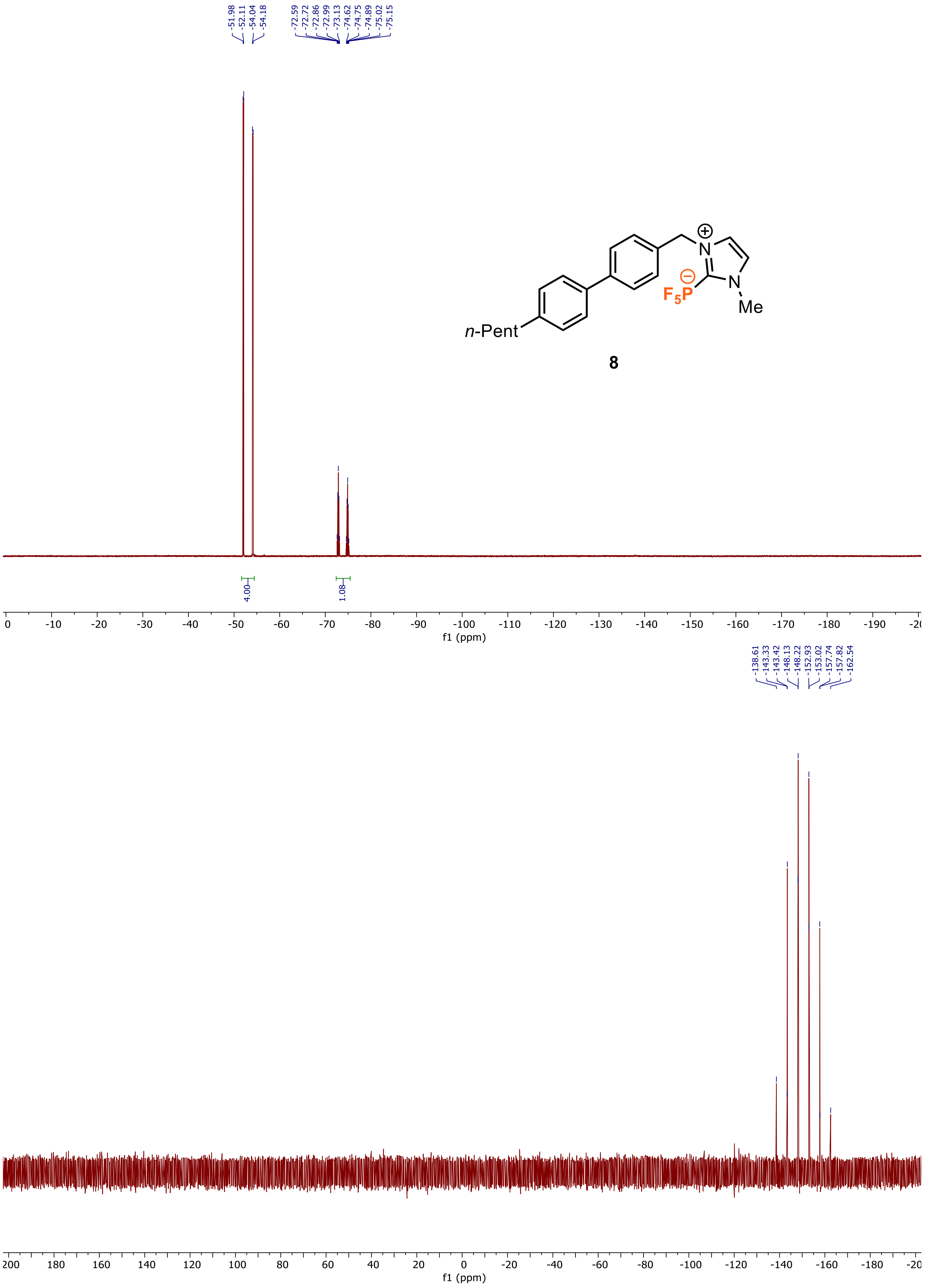


**Figure S72**. Top: $^{19}$F NMR spectrum (376 MHz), and bottom: $^{31}$P NMR spectrum (162 MHz) of **8** in Acetone-$d_6$.

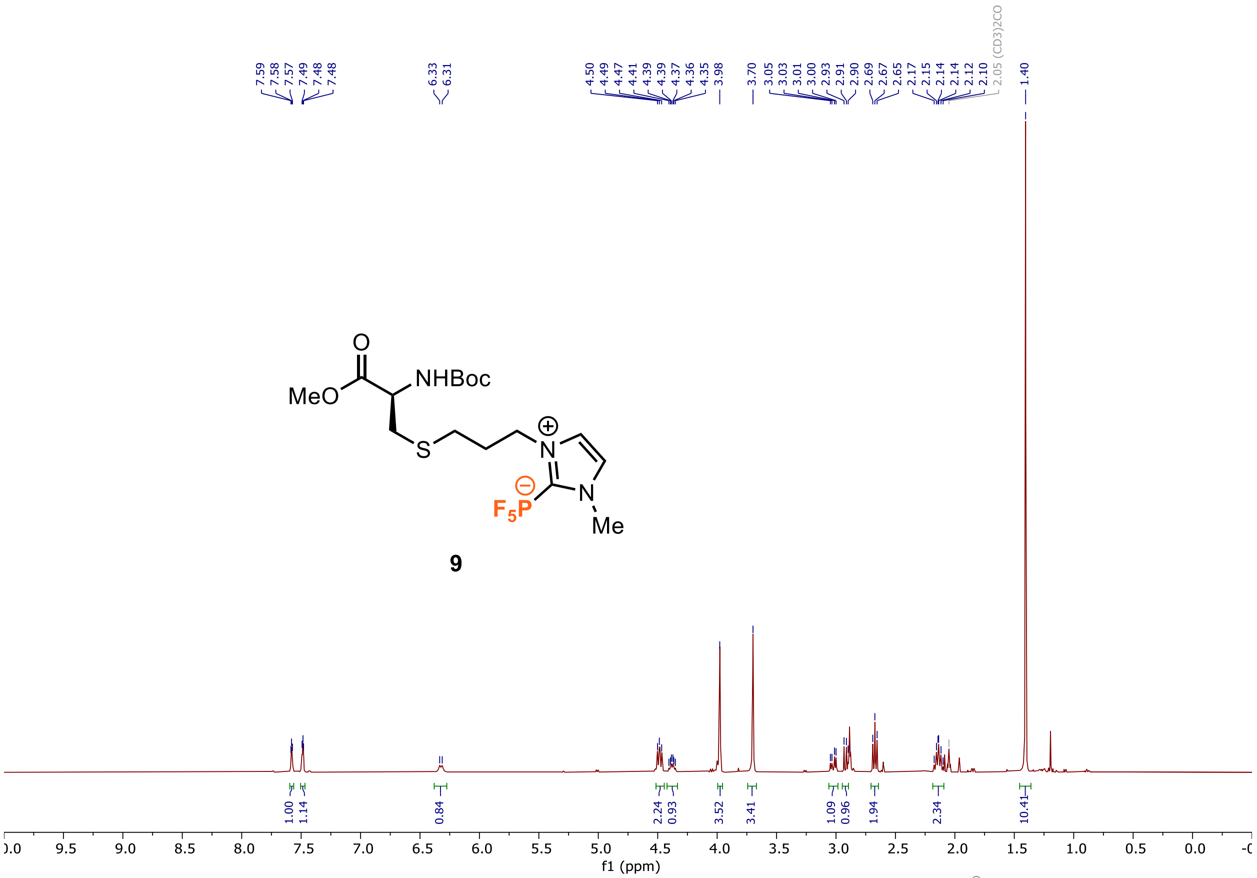


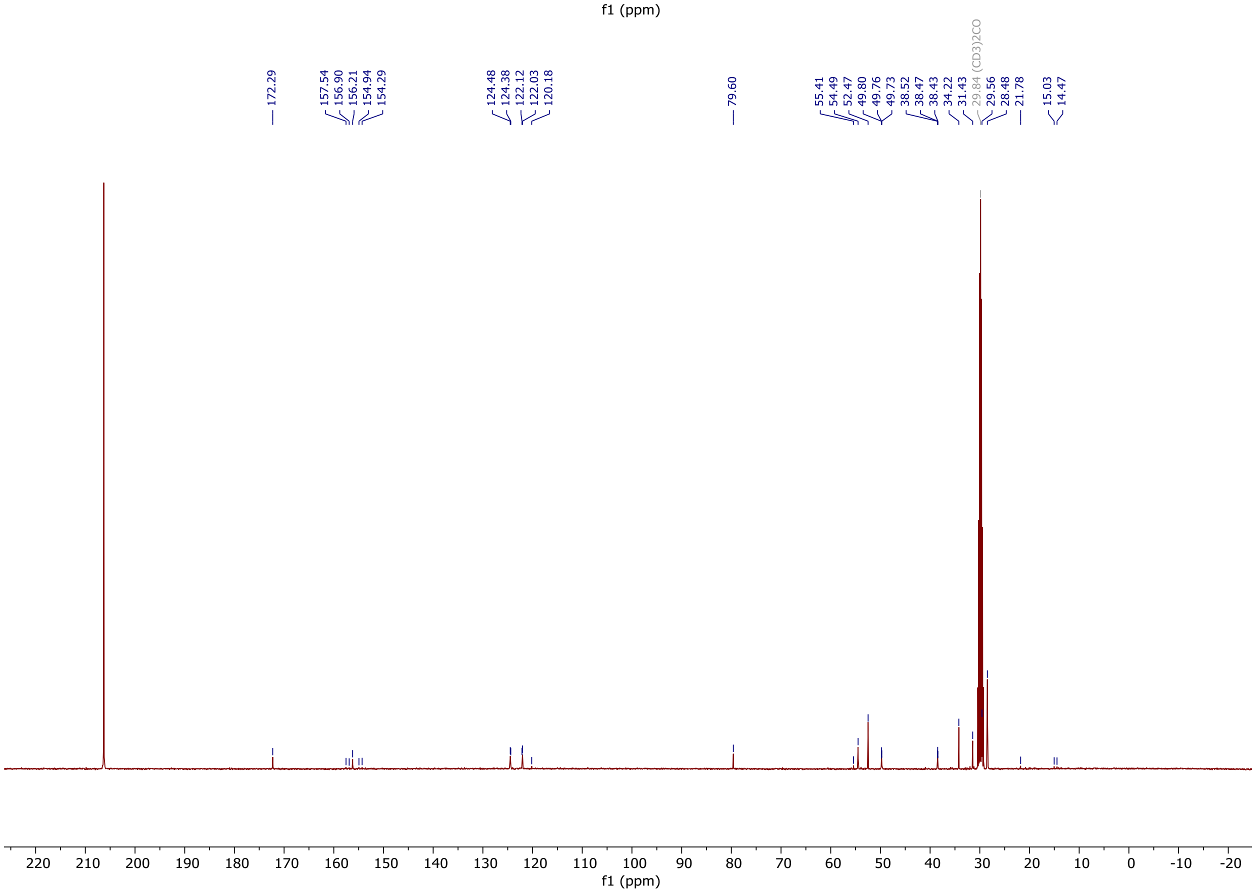


**Figure S73**. Top: $^{1}H$ NMR spectrum (400 MHz), and bottom: $^{13}C$ NMR spectrum (101 MHz) of **9** in Acetone-$d_6$.

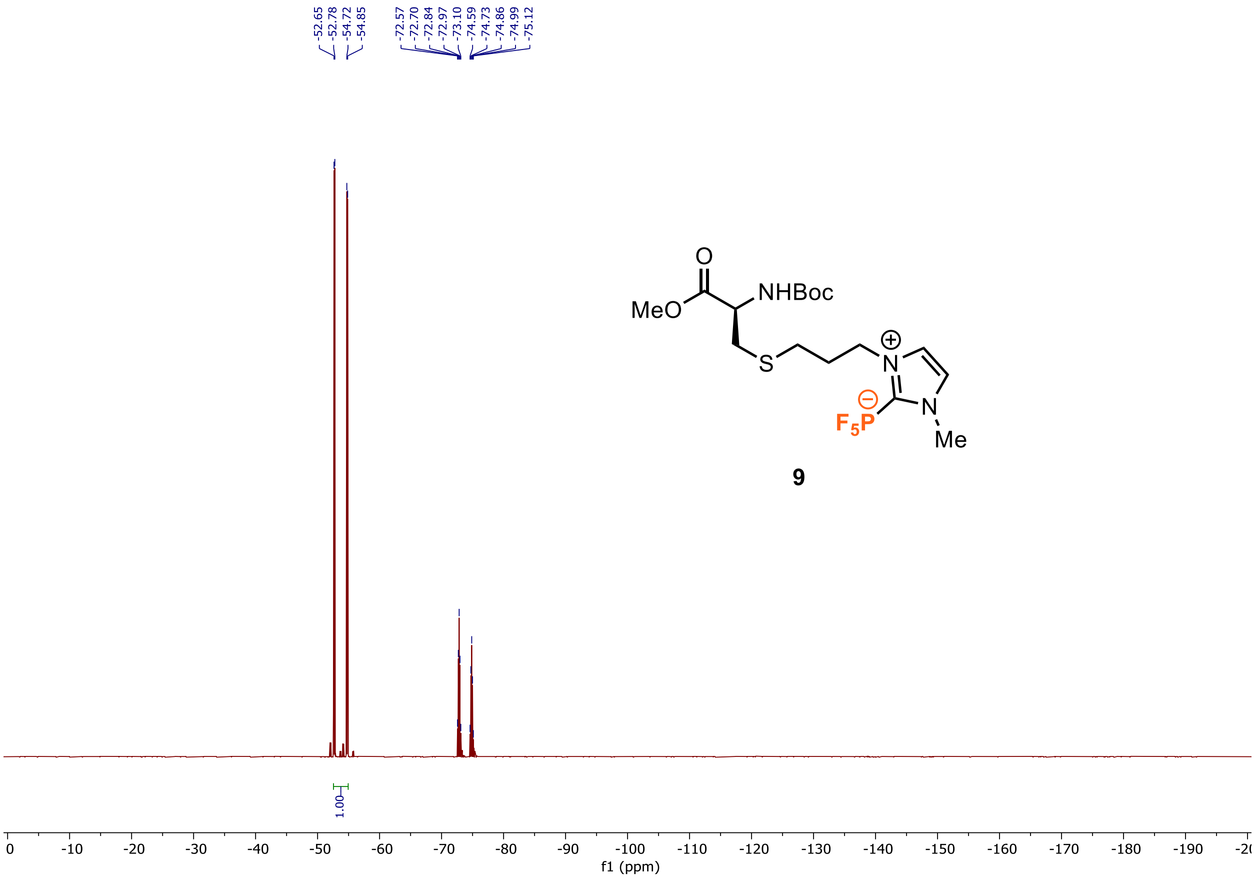


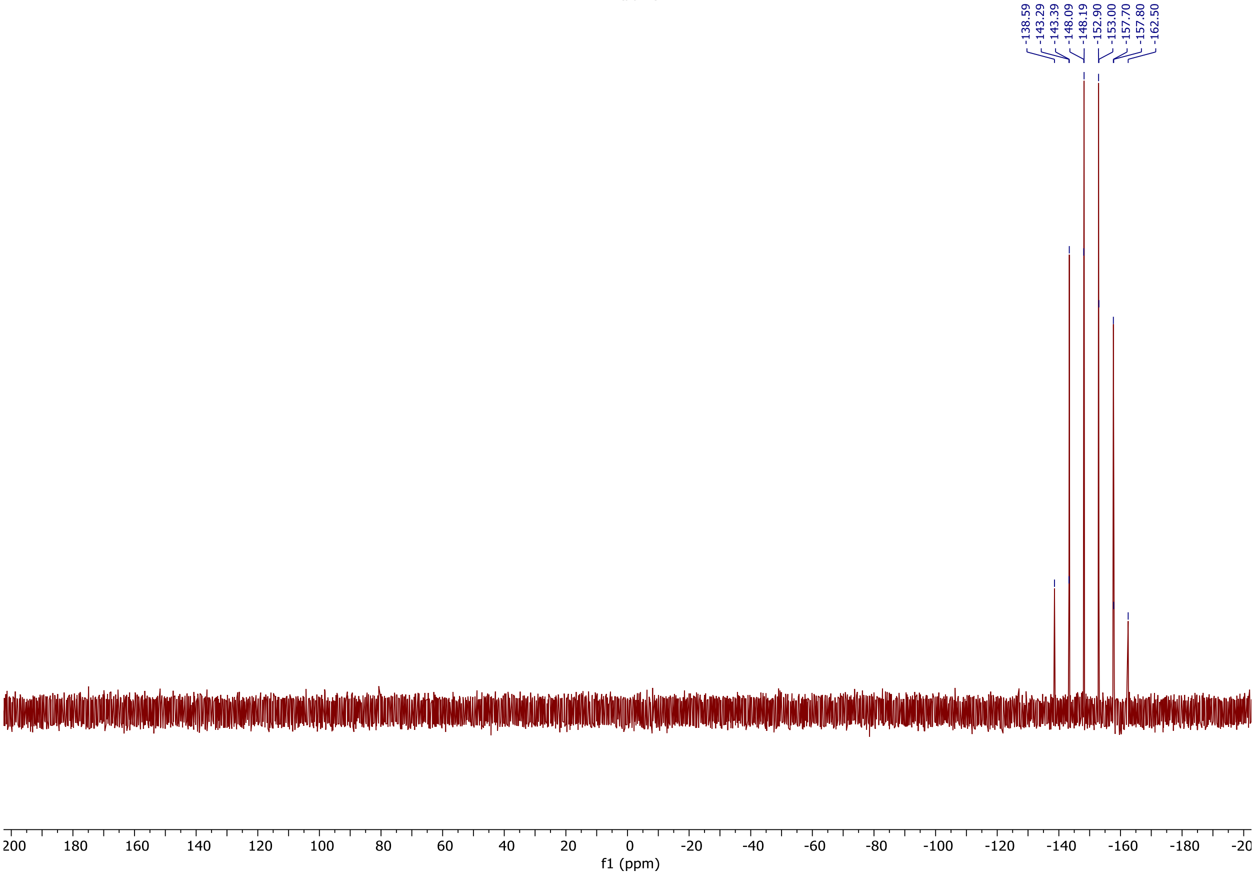


**Figure S74**. Top: $^{19}$F NMR spectrum (376 MHz), and bottom: $^{31}$P NMR spectrum (162 MHz) of **9** in Acetone-$d_6$.

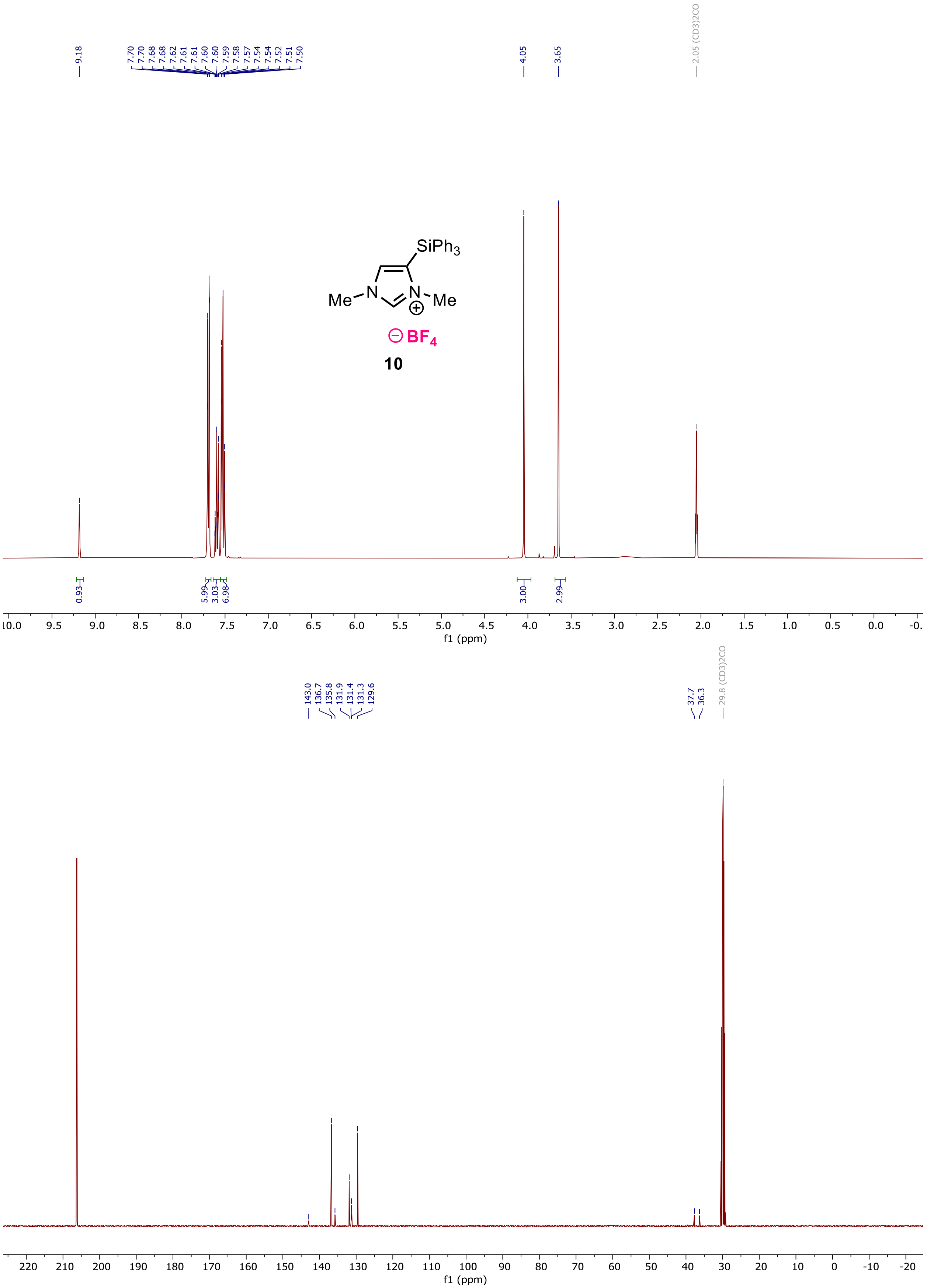


**Figure S75**. Top: $^1$H NMR spectrum (400 MHz), and bottom: $^{13}$C NMR spectrum (101 MHz) of **10** in Acetone-$d_6$.

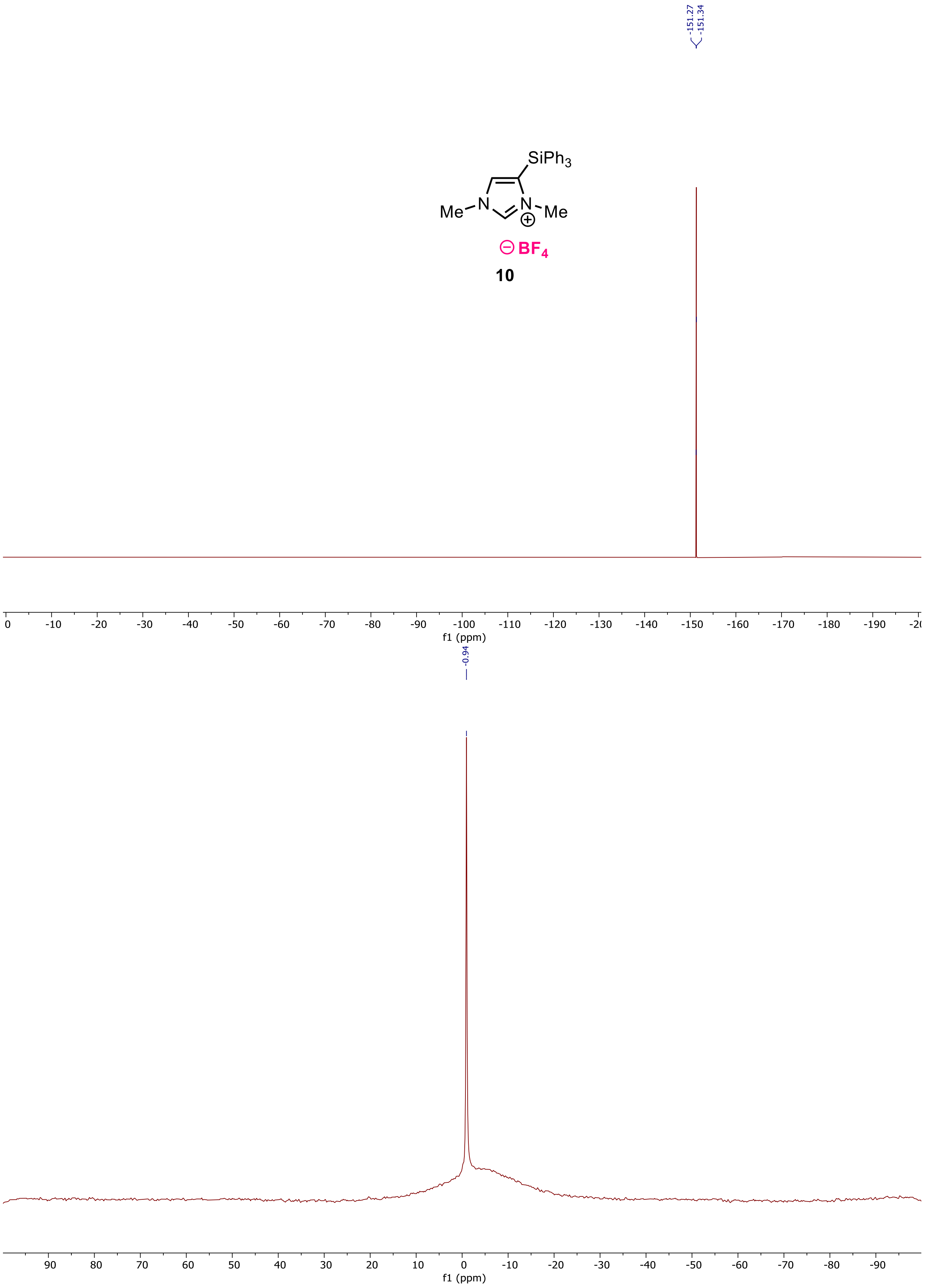


**Figure S76**. Top: $^{19}$F NMR spectrum (376 MHz), and bottom: $^{11}$B NMR spectrum (96 MHz) of **10** in Acetone-$d_6$.

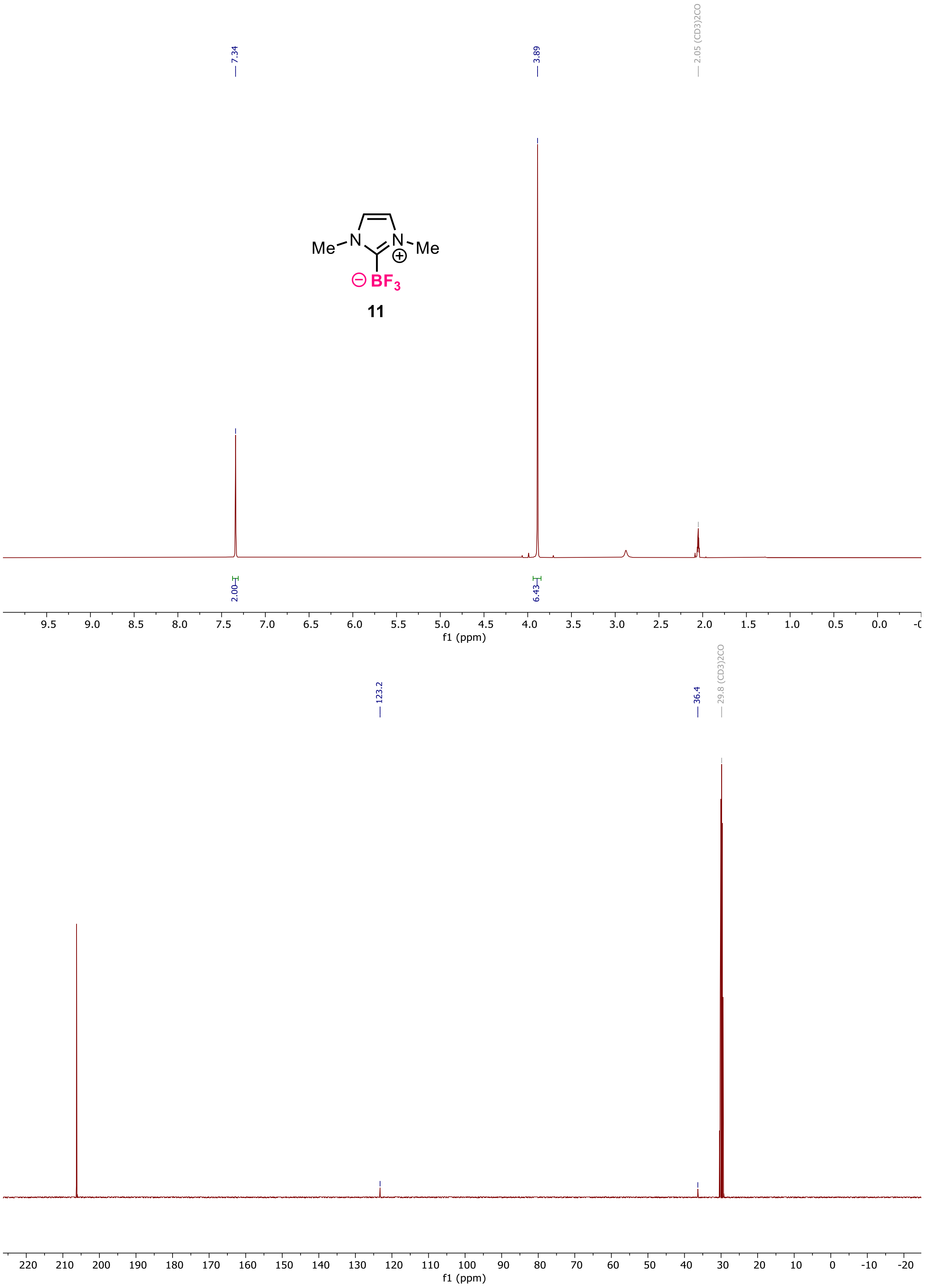


**Figure S77**. Top: $^{1}$H NMR spectrum (400 MHz), and bottom: $^{13}$C NMR spectrum (101 MHz) of **11** in Acetone-$d_6$.

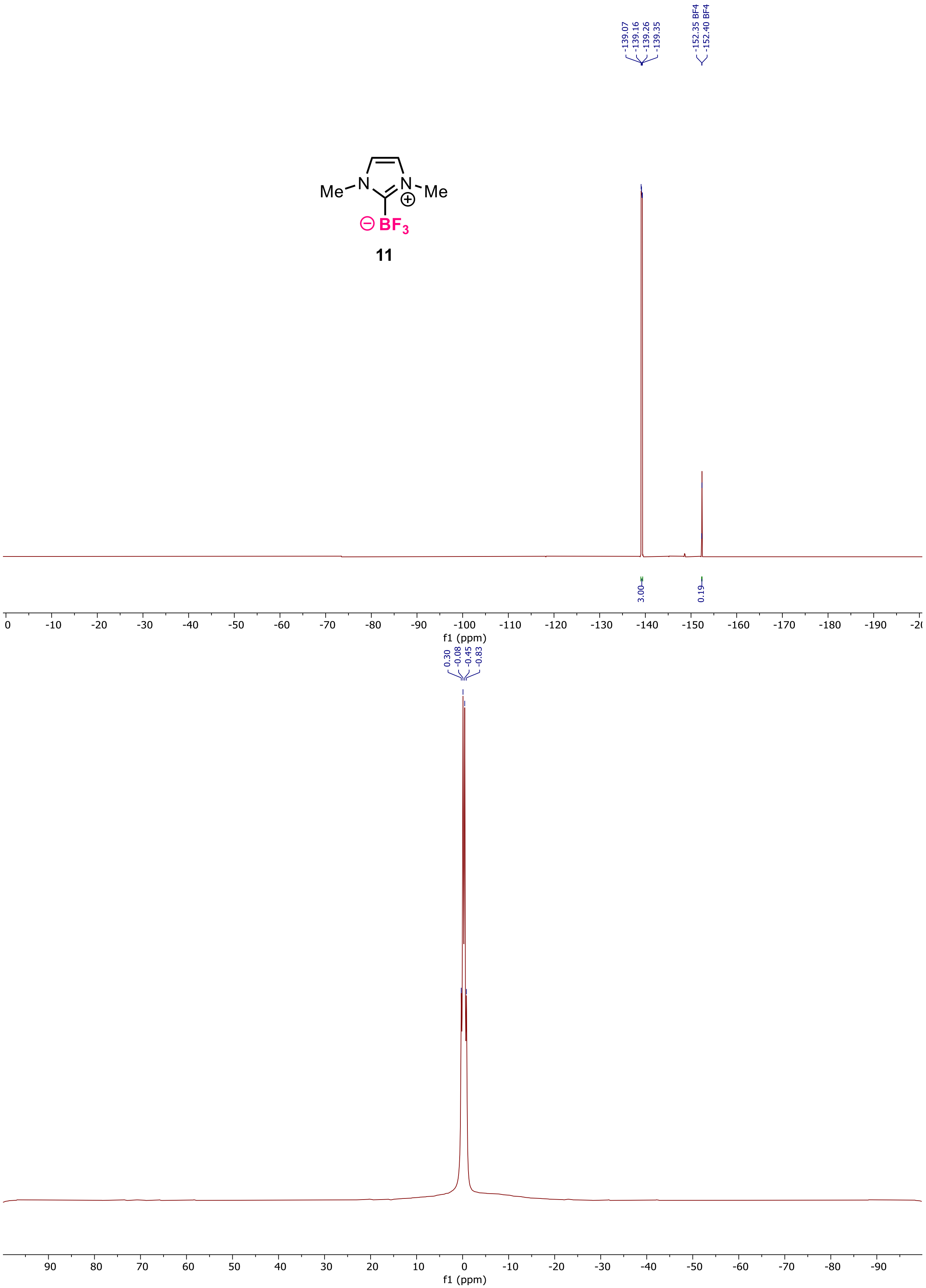


**Figure S78**. Top: $^{19}F$ NMR spectrum (376 MHz), and bottom: $^{11}B$ NMR spectrum (96 MHz) of **11** in Acetone-$d_6$.

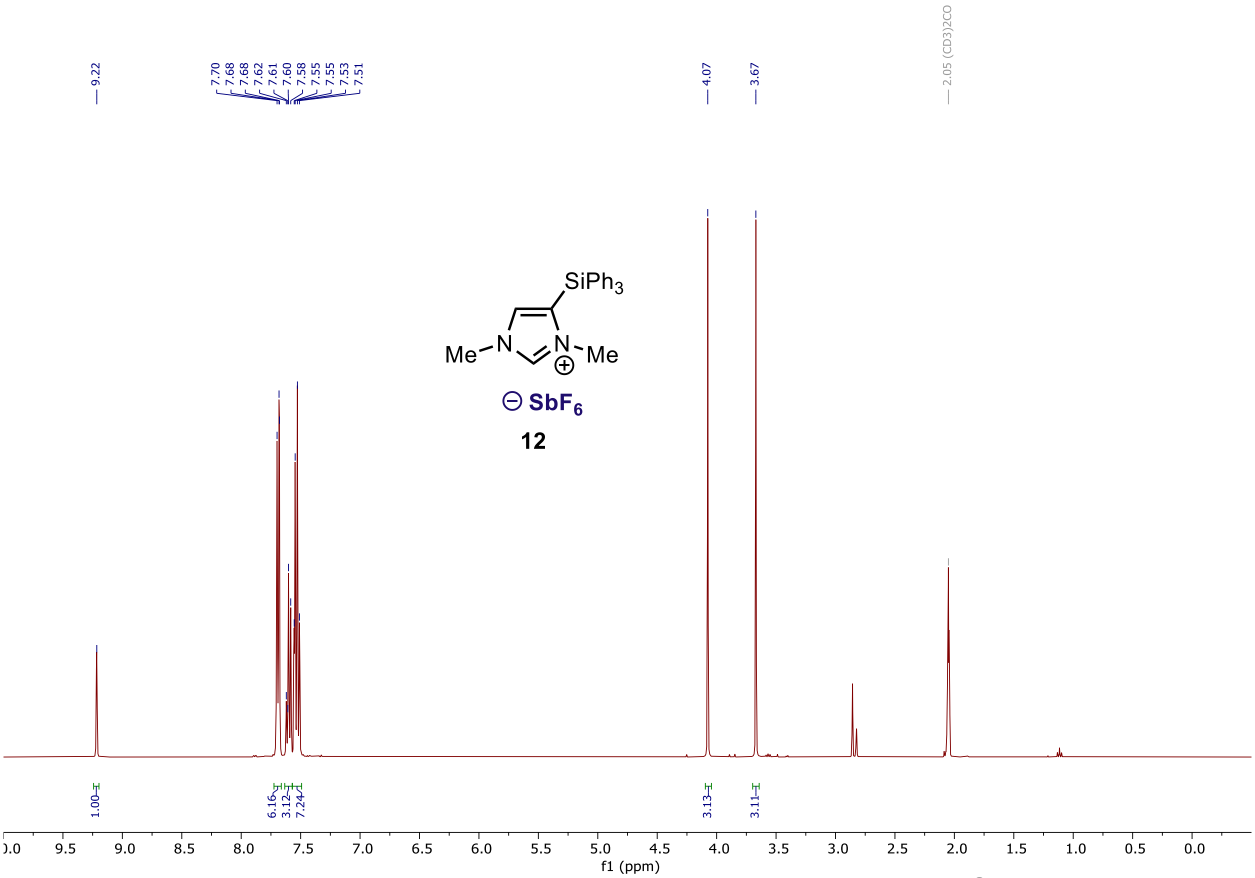


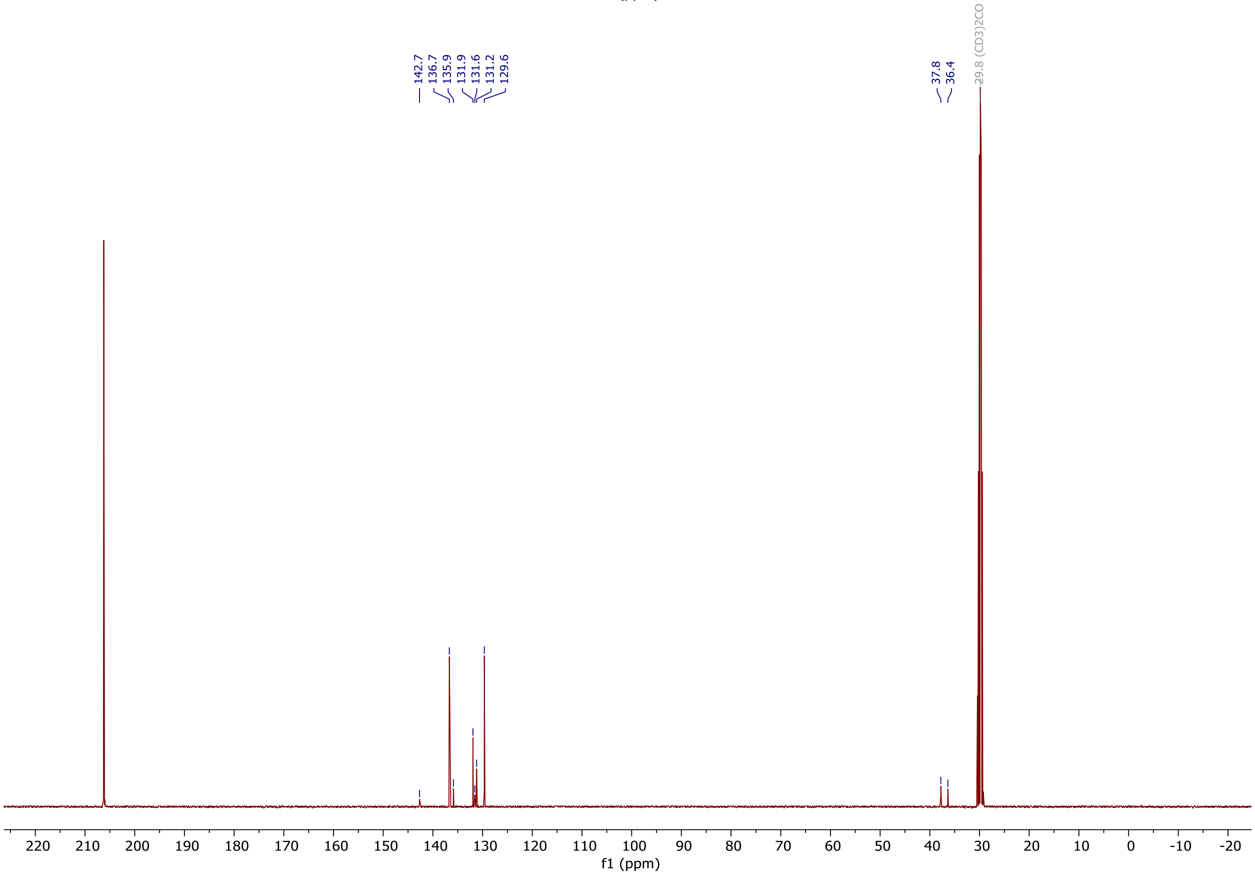


**Figure S79**. Top: $^{1}$H NMR spectrum (400 MHz), and bottom: $^{13}$C NMR spectrum (101 MHz) of **12** in Acetone-$d_6$.

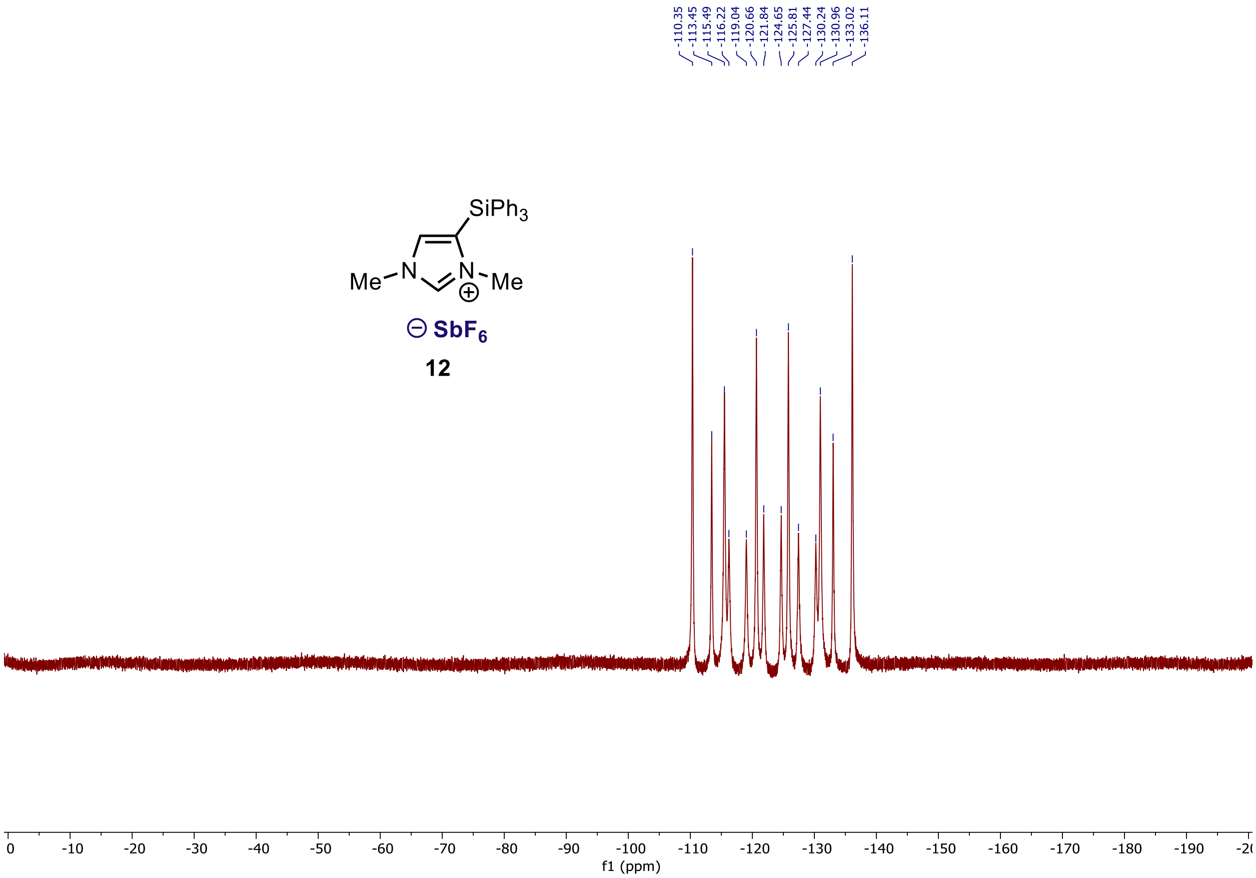


**Figure S80**. $^{19}$F NMR spectrum (376 MHz) of **12** in Acetone-$d_6$.

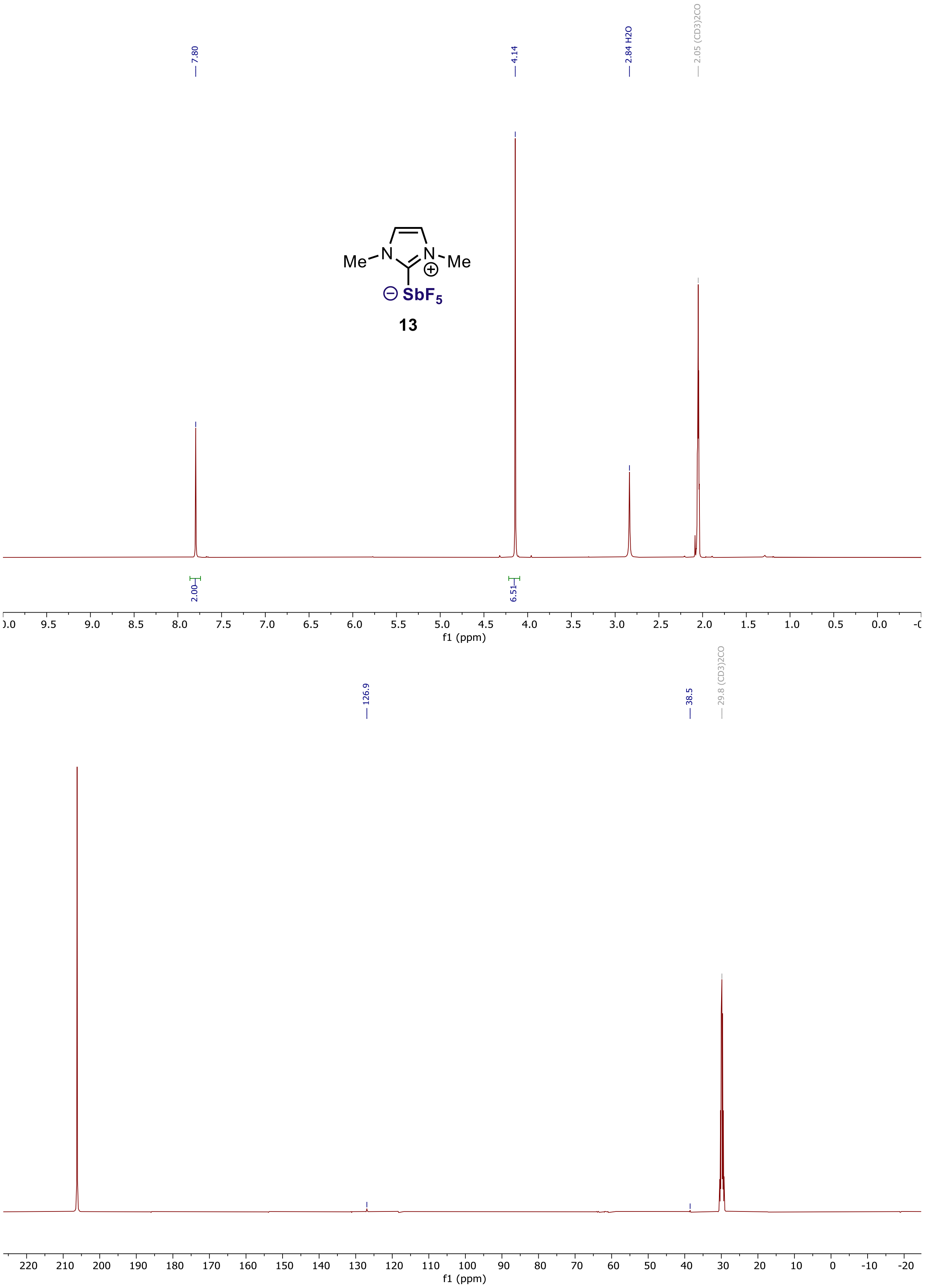


**Figure S81**. Top: $^{1}$H NMR spectrum (400 MHz), and bottom: $^{13}$C NMR spectrum (101 MHz) of **13** in Acetone-$d_6$.

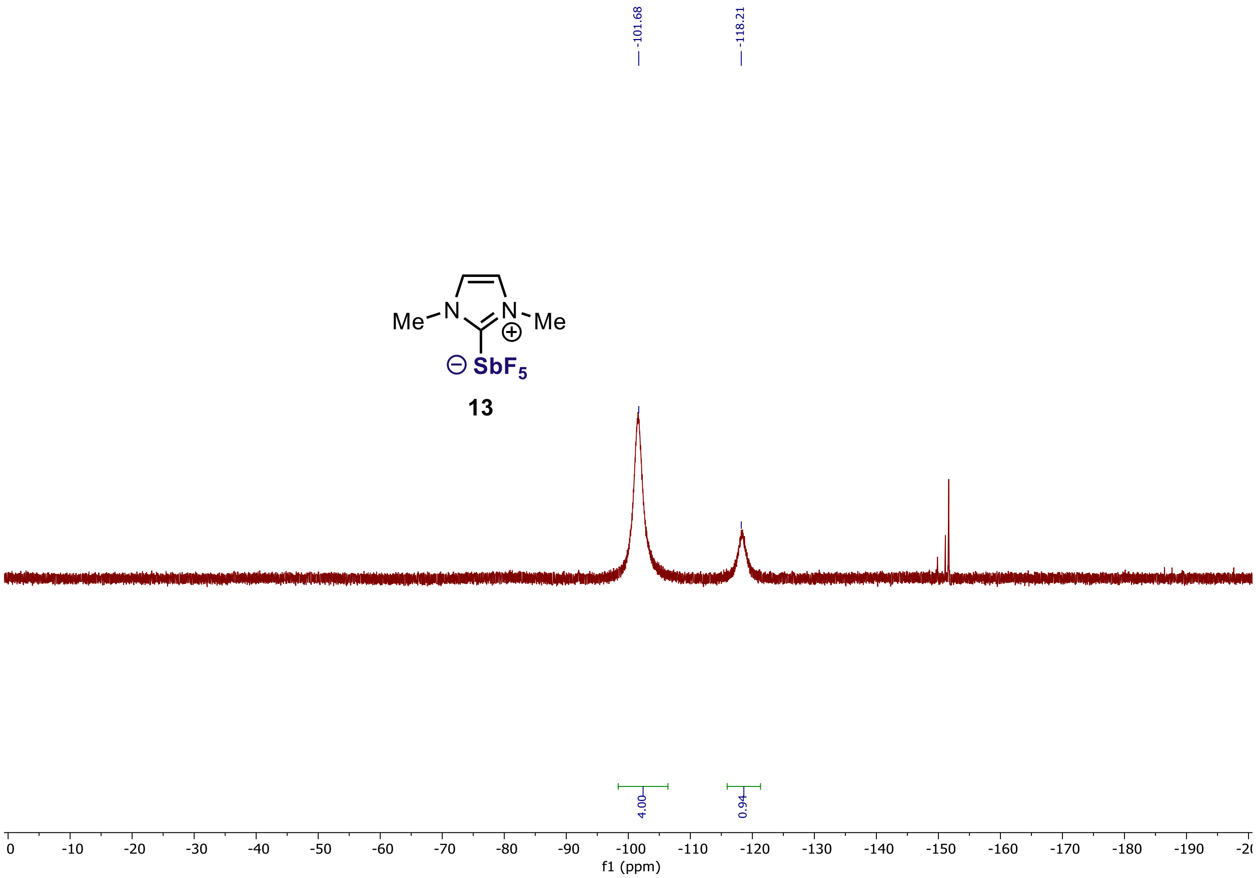


**Figure S82**. $^{19}$F NMR spectrum (376 MHz) of **13** in Acetone-$d_6$.

## 8. References


(1) Fulmer, G. R.; Miller, A. J. M.; Sherden, N. H.; Gottlieb, H. E.; Nudelman, A.; Stoltz, B. M.; Bercaw, J. E.; Goldberg, K. I. NMR Chemical Shifts of Trace Impurities: Common Laboratory Solvents, Organics, and Gases in Deuterated Solvents Relevant to the Organometallic Chemist. *Organometallics* **2010**, *29* (9), 2176–2179. https://doi.org/10.1021/om100106e.

(2) Shapiro, G.; Marzi, M. Synthesis of 2,5-Dilithio-1-Methylimidazole. *Tetrahedron Lett.* **1993**, *34* (21), 3401–3404. https://doi.org/10.1016/S0040-4039(00)79166-0.

(3) Effenberger, F.; Roos, M.; Ahmad, R.; Krebs, A. Carbodesilylierung von (Trimethylsilyl)Imidazolen Und -Pyrazolen. *Chem. Ber.* **1991**, *124* (7), 1639–1650. https://doi.org/10.1002/cber.19911240727.

(4) Tian, C.; Nie, W.; Chen, Q.; Sun, G.; Hu, J.; Borzov, M. V. C- and N-Adducts of N-Alkenyl Substituted Arduengo Carbene and N-Alkyl Substituted Imidazole with PF5: Synthesis and Structural Investigation. *Russ. Chem. Bull.* **2014**, *63* (12), 2668–2674. https://doi.org/10.1007/s11172-014-0796-z.

(5) Aldeco-Perez, E.; Rosenthal, A. J.; Donnadieu, B.; Parameswaran, P.; Frenking, G.; Bertrand, G. Isolation of a C5-Deprotonated Imidazolium, a Crystalline "Abnormal" N-Heterocyclic Carbene. *Science* **2009**, *326* (5952), 556–559. https://doi.org/10.1126/science.1178206.

(6) Sheldrick, G. M. SADABS, Program for Area Detector Adsorption Correction. *Inst. Inorg. Chem. Univ. Gött. Ger.* **1996**, *33*.

(7) Sheldrick, G. M. *SHELXT* – Integrated Space-Group and Crystal-Structure Determination. *Acta Crystallogr. Sect. Found. Adv.* **2015**, *71* (1), 3–8. https://doi.org/10.1107/S2053273314026370.

(8) Sheldrick, G. M. Crystal Structure Refinement with *SHELXL*. *Acta Crystallogr. Sect. C Struct. Chem.* **2015**, *71* (1), 3–8. https://doi.org/10.1107/S2053229614024218.

(9) Dolomanov, O. V.; Bourhis, L. J.; Gildea, R. J.; Howard, J. a. K.; Puschmann, H. OLEX2: A Complete Structure Solution, Refinement and Analysis Program. *J. Appl. Crystallogr.* **2009**, *42* (2), 339–341. https://doi.org/10.1107/S0021889808042726.

(10) Van Der Sluis, P.; Spek, A. L. BYPASS: An Effective Method for the Refinement of Crystal Structures Containing Disordered Solvent Regions. *Acta Crystallogr. A* **1990**, *46* (3), 194–201. https://doi.org/10.1107/S0108767389011189.
